# Selfish Spermatogonial Selection: Evidence from an Immunohistochemical Screen in Testes of Elderly Men

**DOI:** 10.1371/journal.pone.0042382

**Published:** 2012-08-06

**Authors:** Jasmine Lim, Geoffrey J. Maher, Gareth D. H. Turner, Wioleta Dudka-Ruszkowska, Stephen Taylor, Ewa Rajpert-De Meyts, Anne Goriely, Andrew O. M. Wilkie

**Affiliations:** 1 Clinical Genetics Group, Weatherall Institute of Molecular Medicine, University of Oxford, Oxford, United Kingdom; 2 Department of Cellular Pathology, NIHR Biomedical Research Centre, Oxford University Hospitals NHS Trust, Oxford, United Kingdom; 3 Computational Biology Research Group, Weatherall Institute of Molecular Medicine, University of Oxford, Oxford, United Kingdom; 4 University Department of Growth and Reproduction, Copenhagen University Hospital (Rigshospitalet), Copenhagen, Denmark; The Institute of Cancer Research, United Kingdom

## Abstract

The dominant congenital disorders Apert syndrome, achondroplasia and multiple endocrine neoplasia–caused by specific missense mutations in the FGFR2, FGFR3 and RET proteins respectively–represent classical examples of paternal age-effect mutation, a class that arises at particularly high frequencies in the sperm of older men. Previous analyses of DNA from randomly selected cadaveric testes showed that the levels of the corresponding *FGFR2*, *FGFR3* and *RET* mutations exhibit very uneven spatial distributions, with localised hotspots surrounded by large mutation-negative areas. These studies imply that normal testes are mosaic for clusters of mutant cells: these clusters are predicted to have altered growth and signalling properties leading to their clonal expansion (selfish spermatogonial selection), but DNA extraction eliminates the possibility to study such processes at a tissue level. Using a panel of antibodies optimised for the detection of spermatocytic seminoma, a rare tumour of spermatogonial origin, we demonstrate that putative clonal events are frequent within normal testes of elderly men (mean age: 73.3 yrs) and can be classed into two broad categories. We found numerous small (less than 200 cells) cellular aggregations with distinct immunohistochemical characteristics, localised to a portion of the seminiferous tubule, which are of uncertain significance. However more infrequently we identified additional regions where entire seminiferous tubules had a circumferentially altered immunohistochemical appearance that extended through multiple serial sections that were physically contiguous (up to 1 mm in length), and exhibited enhanced staining for antibodies both to FGFR3 and a marker of downstream signal activation, pAKT. These findings support the concept that populations of spermatogonia in individual seminiferous tubules in the testes of older men are clonal mosaics with regard to their signalling properties and activation, thus fulfilling one of the specific predictions of selfish spermatogonial selection.

## Introduction

Amongst the diverse factors shaping the frequency of different mutations in the genome, selfish spermatogonial selection is an important new concept emerging from human genetic studies. Evidence for this process is strongest for human congenital disorders caused by spontaneous mutations in the genes *FGFR2*, *FGFR3*, *HRAS, PTPN11* and *RET* [1], [2], [3], [4], [5]. These so-called paternal age-effect (PAE) mutations encode proteins with gain-of-function biochemical mechanisms of action, and exhibit shared properties of a substantially elevated apparent point mutation rate, near-exclusive paternal origin, and an increased age at paternity of the unaffected fathers compared to the average for the population (reviewed in [6]). Experimental studies of sperm in the case of *FGFR2* [7], [8], [9], *FGFR3* [10], [11] and *HRAS* [12] have directly demonstrated increased levels of relevant mutations, that are positively correlated with the age of the donor and account for the elevated birth prevalence associated with these spontaneous disorders.

The identical nature of many germline PAE mutations to somatic mutations identified in certain cancers [6] led to a search for the corresponding mutations in testicular tumours. Indeed, functionally related mutations of *FGFR3* and *HRAS* were identified in spermatocytic seminoma [11], a rare testicular tumour distinct from more common seminomatous and non-seminomatous neoplasms [13]. Spermatocytic seminomas affect an older age group of men and are believed to originate from spermatogonia, the self-renewing spermatogenic stem cells of the adult testis [14], [15], [16], [17]. The PAE genes *FGFR2*, *FGFR3* and *RET* encode receptor tyrosine kinase proteins essential for binding trophic signalling factors on the surface of spermatogonia [18]; *PTPN11* and *HRAS* encode intracellular components of the signalling network downstream of these receptors, and a critical role for HRAS activation has been demonstrated in mouse spermatogonial proliferation [19]. These observations have led to the proposal that activation by rare gain-of-function mutations affecting growth factor receptor-RAS signal transduction within spermatogonia (likely through the MEK-ERK and/or phosphoinositide-3 kinase [PI3K]-AKT pathways) [19], leads to progressive clonal expansion of the mutation with age, explaining the paternal age effect [6], [11]. This process, which we term selfish spermatogonial selection, is likely to affect all men as they age.

Of several experimental approaches to test the selfish selection model, one is to look directly for evidence of clonal expansion events within human testes. This has previously been undertaken by examining whole cadaveric testes or testis biopsies for regional variation in the levels of specific PAE mutations. The most detailed studies have been undertaken for two different mutations of *FGFR2* (c.755C>G encoding p.Ser252Trp and c.758C>G encoding p.Pro253Arg, both causing Apert syndrome) and a mutation in *RET* (c.2943T>C, encoding p.Met918Thr and causing multiple endocrine neoplasia type 2B [Men2B]) [20], [21], [22]. Dissection of a total of 16 testes from men aged 19–80 years into 192 pieces each, followed by DNA extraction and mutation quantification by a sensitive and specific PCR-based assay, showed that most testes exhibited a small number of spatially localised hotspots of mutation (0–8 hotspots per testis, with maximum mutation levels as high as 6% per haploid genome in individual testis pieces), with large surrounding “deserts” in which the mutation was undetectable (<25×10^−6^) [20], [21], [22]. Mathematical modelling of the numerical data obtained on these three specific mutations and the earlier data on sperm [7], [8], showed that if the effect of rare originating mutations was to promote occasional (in the order of ∼1 every 100 divisions) symmetrical self-renewal of the mutant cell, then over the male lifespan a handful of expanding clones containing the mutations would be expected in the entire testis, closely matching the experimental findings [20], [21], [22], [23]. A conceptually similar study of the *FGFR3* c.1138G>A achondroplasia mutation (encoding p.Gly380Arg) obtained broadly comparable results, with maximum mutation levels up to 2.3% in testis biopsies [24]. Since multiple mutations in different genes are expected to confer selective advantage to the mutant spermatogonial cell, such clonal cellular phenomena are expected to be universal and should be identified more readily in the testes of elderly men. The obvious question arises whether such events can be directly visualised within testicular tissue, but the process of DNA extraction used in the studies above [20], [21], [22], [24] prevents localisation of the process at a tissue level.

**Figure 1:**
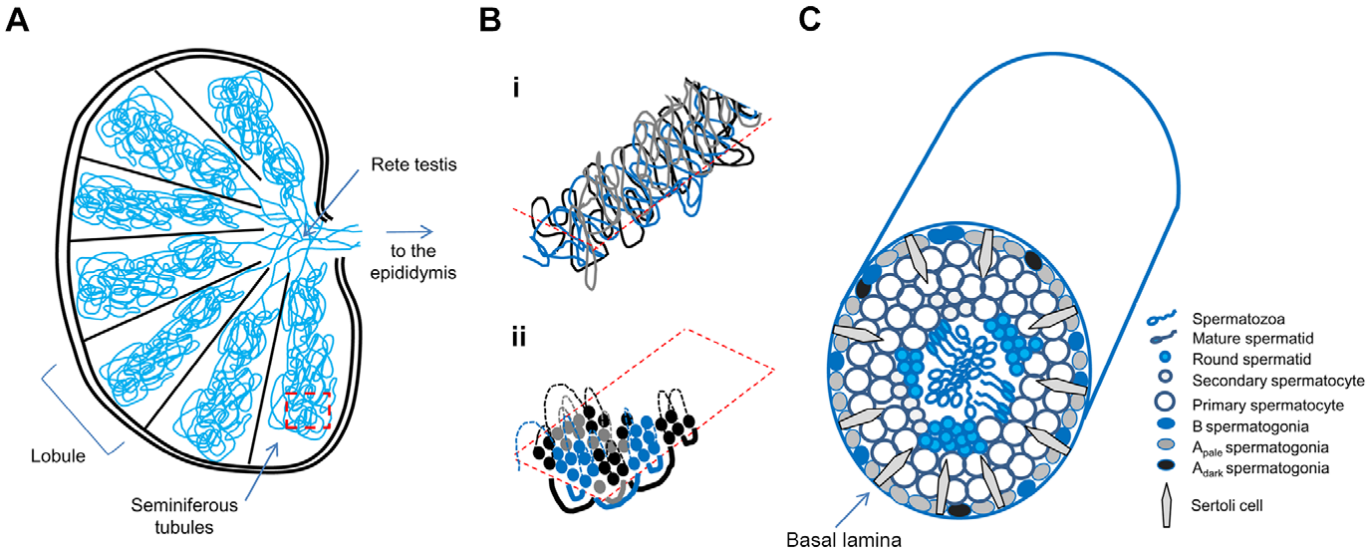
Structure and organisation of the human testis and seminiferous tubules. **A.** Macroscopic appearance. The testis is divided into compartments (lobules) separated by connective tissue. There are an estimated 250 lobules per testis, which vary in size; for clarity only 7 lobules are illustrated here. **B.** (**i**) Diagrammatic representation of the highly convoluted organisation of 3 seminiferous tubules (blue, black, grey) within a lobule. (**ii**) A magnified cross section through the lobule in **i** reveals how the tubules would appear in thin microscopic sections. Dashed lines (above section) and solid lines (below section) join the contiguous tubules. **C.** Cross section through an individual seminiferous tubule. Spermatogonia, located at the basal lamina of the seminiferous tubules, comprise a heterogeneous population of diploid germ cells. These can be classified according to their morphologies and correspond to three main maturation stages: A_dark_ spermatogonia, which are considered to represent the reserve stem cell population; highly proliferating A_pale_ spermatogonia; and more mature B spermatogonia that give rise to primary spermatocytes. Primary spermatocytes undergo meiosis to form secondary spermatocytes that differentiate to form spermatids, which when fully formed are released into the central lumen as spermatozoa that progress to the rete testis. Spermatogenesis is supported by the presence of the somatic Sertoli cells. The vascular and connective tissue network lie external to the wall of the seminiferous tubule.

In approaching the challenge of how to visualise putative clones within the whole testis, it is important to appreciate the structural organisation of the seminiferous epithelium, which is organised into highly convoluted tubules (Figure 1A). Each testis comprises an average of 500 seminiferous tubules that are ∼70–80 cm in length and ∼120–300 µm in diameter [25]. All tubules start and end at the rete testis, where the sperm are collected, and are organised into tightly packed testicular lobules comprising a small number (1–4) of tubules bound together by connective tissue and surrounded by interstitial cells and vascular structures. As a consequence, cross-sections through testicular tissue show several distinct sections of the length of the same tubule (Figure 1B). At higher magnification, tubular cross-sections reveal the architecture of the seminiferous epithelium (Figure 1C), in which spermatogenesis occurs in a highly regulated manner [26], [27]. Spermatogonia, the proliferating diploid germline stem cells, form a single layer on the basal lamina of the tubule; differentiation proceeds in an organised centripetal sequence from type A and B spermatogonia, to spermatocytes, which undergo two meiotic divisions to form haploid spermatids that, after maturation, are released into the lumen of the tubule (Figure 1C).

To visualise selfish spermatogonial clones within the seminiferous tubules, several strategies might be envisaged. First, ultrastructural features of groups of cells compared to their neighbours (based on either light or electron microscopic visualisation) might suggest altered growth/differentiation characteristics; second, altered patterns of immunohistochemical phenotype, either for spermatogonial antigens or for markers of signalling pathway activation, might indicate altered antigenic and/or signalling properties in situations where cellular appearances were unchanged; third, variant characteristics identified by one of the first two methods could be correlated directly with the presence of cellular mutation or clonality, for example using laser capture microdissection followed by mutation analysis, or *in situ* mutation detection techniques.

At present, relatively weak evidence for mosaicism in the testis is available using only the first of the three approaches outlined above. Localised alterations in the appearance of the seminiferous tubules were originally described using electron microscopy by Holstein et al. in 1984 [28]. These authors examined the testes of three men aged 68–72 years, who had been treated for prostate cancer by orchidectomy prior to chemotherapy. Cells with the characteristic ultrastructural appearances of type A spermatogonia (which normally occur strictly adjacent to the basal lamina of the tubule (Figure 1C)), were observed at various ectopic positions both within the tubule and in the tubular lumen. In some cases the presence of so-called “clusters” or “plaques” of spermatogonial cells was apparent, and in others the basal spermatogonia appeared as a double layer. These appearances were not seen in men under 65 years of age, except in the case of established seminoma. Holstein et al. proposed [28] that the ectopic spermatogonia were proliferating but had lost their ability to differentiate. Similar appearances have also been described in infertile men [29], and close to the rete testis in a young healthy man [30]. Pop et al. [31] analysed variations in the thickness of the basal lamina that had previously been associated with reduction in spermatogenesis efficiency and advancing age [32], and described a ‘mosaic’ focal distribution of increased lamina thickness in the testes of elderly men. These observations [28], [29], [31], [32] describing localised/mosaic cellular appearances in the testes are consistent with a clonal proliferation mechanism. Another relevant consideration is that spermatocytic seminoma, the tumour proposed to arise from adult spermatogonia (see above), is believed to commence as an intratubular lesion, in which abnormal cells accumulate within seminiferous tubules [33]. However identification of such intratubular spermatocytic seminoma (ISS) alone (without neighbouring overt tumour) is extremely rare [14], because ISS is asymptomatic and is not associated with infertility or testicular dysgenesis.

Although collectively the above observations hint that clonal proliferation events may occur in macroscopically normal testes, the issue has not been explored systematically [34], [35], [36], nor have modern immunohistochemical knowledge and techniques – potentially much more powerful than ultrastructural analysis – been exploited. Our aim therefore, was to develop a robust immunohistochemical methodology to search for potential mosaic/clonal events that could correspond to selfish clones in normal testes. We have addressed this problem in four stages. First, we optimised a panel of relevant antibodies to use in an immunohistochemical screen, based on the published antigenic characteristics of type A spermatogonia [16], [37] and spermatocytic seminoma [15], [16], and the insight that elevated activity through pathways involving receptor tyrosine kinase-RAS signalling is predicted in selfish spermatogonial mutations [6], [11]. Second, we developed objective criteria to classify unusual staining appearances of cellular clusters viewed on light microscopy of individual testicular sections. Third, we analysed adjacent sections for the same, or different, antigens using standardised criteria, and only annotated flagged clusters as potential clones when these clusters had been independently identified on two or more different sections. Finally, we examined these potential clones for evidence of abnormal signal activation downstream of RAS with a marker of phosphorylated AKT (pAKT). Using this approach we demonstrate that given a reasonable quality of tissue preservation, localised abnormalities of immunohistochemical staining can be visualised in most testes from elderly men.

## Materials and Methods

### Testis Samples

Formalin-fixed, paraffin-embedded testis blocks from anonymised patients who had given informed written consent for research use were obtained from the tissue bank of the Department of Cellular Pathology, John Radcliffe Hospital, Oxford, according the protocols of the Oxford Centre for Histopathology Research (OCHRe). Ethical approval was given by the Oxfordshire Research Ethics Committee A (C03.076: Receptor tyrosine kinases and germ cell development: detection of mutations in normal testis, testicular tumours and sperm). Information on the indication for orchidectomy, use of pre-operative chemotherapy (where relevant) and age of the donor was obtained. The criteria for normal testes used in this study were that the majority of seminiferous tubules exhibited an acceptable degree of spermatogenesis, measured using Johnsen’s criteria [38]. Testes for which the Johnsen score [38] of spermatogenesis was 6 or less, or in which the cellular morphology or results of initial immunohistochemistry were unsatisfactory, were not included. Information on the testis samples from six individuals used in this work is provided in Table 1.

**Table 1:** Testis samples used in this work.

Samplenumber	Johnsenscore [38]	Donor age (years)	Indication for orchidectomy	Previous treatment
1	9	71	Prostate cancer	Deep X-rays and 3 months of goserilin acetate, 5 years prior to orchidectomy
2	9	75	Infected haematoma after excision of a benign epididymal cyst	Chemotherapy for oesophageal adenocarcinoma, 6 years prior to orchidectomy
3	9	78	Incarcerated right inguinal hernia	None, concurrent hydrocoele at orchidectomy
4	8	75	Incarcerated left inguinal hernia	None
5	7	72	Simple epithelial cyst, and cystic rete testis	None
6	9	69	Prostate cancer	None

### Antibodies and Immunohistochemistry

The primary antibodies used have all been validated and published previously as detailed in Table 2. Formalin-fixed, paraffin-embedded tissue sections (5 µm) were dried at 55°C for 1 hour, deparaffinised twice in Histoclear (National Diagnostics) and rehydrated with graded ethanol incubations (100%, 95%, 80% and 70%) for 5 min each. For antigen retrieval, slides were immersed in citrate buffer (10 mM, pH 6) and heated in a microwave on high mode for 1 min, followed by simmer mode for 15 min. Slides were cooled to room temperature and incubated with Dako REAL™ Peroxidase-Blocking Solution (code S2023, Dako) for 10 min to quench endogenous peroxidase activity. Subsequently, sections were incubated overnight at 4°C with primary antibodies at the optimal concentration (Table 2). Primary antibodies were detected using the EnVision+Dual Link System, Peroxidase (DAB+) kit (code K5007, Dako). After binding with Dako Envision™/Horse Radish Peroxidase, Rabbit/Mouse polymer, sections were incubated with 3,3′-diaminobenzidine (DAB+) substrate-chromogen for 10 min before counterstaining with haematoxylin, and mounted with Aquatex® (MERCK). During incubations, slides were placed in a humidity chamber to prevent evaporation and drying of the tissue sections. Washing steps using TBS/Tween-20 buffer were conducted between each step of antibody incubation and detection. Positive and negative control slides were included in each study. After completing the staining, sections were scanned with a dotSlide-digital virtual microscope (Olympus) and the digital images (“virtual slides”) were analysed using the viewer software OlyVIA-Viewer (Olympus).

**Table 2:** Primary antibodies used for immunohistochemistry.

Antibody	Staining pattern^1^	Type^2^	Clone	Epitope	Dilution	Provider	Reference
FGFR3	A and B spermatogonia (pm)	Rabbit pAb	C-15	IgG	1∶1000–1∶4000	Santa Cruz Biotechnology Inc.	[55], [56], [57]
Ki67	Subset of spermatogonia (B > A) (n)	Mouse mAb	MIB-1	IgG	Undiluted	Dako	[58], [59]
MAGEA4	A and B spermatogonia (n, c). Occasional weak staining of spermatocytes (c)	Mouse mAb	57B	IgG	1∶250–1∶1000	Prof. Giulio C. Spagnoli	[54], [60], [61]
OCT2	A_dark_ spermatogonia (n)	Mouse mAb	OCT-207	IgG_2b_	1∶25	Novocastra Laboratories Ltd	[15], [37]
Phospho-Akt (Ser473)	Spermatogonia (c, pm). Occasional weak stainingof spermatocytes (c)	Rabbit mAb	736E11	IgG	1∶25	Cell Signalling Technology	[62], [63], [64]
SAGE1	B spermatogonia (n)	Rabbit pAb	HPA003033	IgG	1∶350–1∶700	Sigma-Aldrich Inc.	[15], [37]
SSX	A_pale_ and B spermatogonia (n)	Mouse mAb	E3AS	IgG	1∶100	Prof. Ad Geurts van Kessel	[15], [65], [66]

1 pm  =  plasma membrane; n =  nuclear; c =  cytoplasm.

2 pAb, polyclonal antibody; mAb, monoclonal antibody.

### Staining Protocols

In preliminary experiments we found that MAGEA4 was both the most robust marker for staining normal spermatogonia, and the antigen most commonly observed in unusual cellular clusters. Therefore to optimise the detection of clusters and enable equivalent tubules in consecutive sections to be accurately followed, we stained every third to fifth serial 5 µm section with anti-MAGEA4. Intervening serial sections were stained with a variety of different antibodies (FGFR3, Ki67, OCT2, pAKT, SAGE1, SSX), in a sequence that differed between testis blocks; the staining order for each testis block is detailed in Dataset S1. For each antibody, spermatogonia and other stages of normal seminiferous tubules were examined to confirm satisfactory staining, as all antigens are normally reactive in a subset of spermatogenic cells (Table 2); sections not showing satisfactory staining were excluded from further analysis. For haematoxylin and eosin (H&E) staining, rehydrated sections were incubated in Ehrlich’s haematoxylin for 8 min and washed with running tap water for 5 min. Subsequently, the sections were dipped in acid alcohol (1% v/v hydrochloric acid in 70% ethanol) 7 times, rinsed with distilled H_2_O, and incubated in 0.1% eosin for 3.5 min. Finally, the sections were dehydrated through 70% ethanol for 2 dips, 100% ethanol for 1 min and Histoclear for 5 min before mounting with Histomount (National Diagnostics).

### Scoring for Cellular Clusters, Microclones, and Immunopositive Tubules

Sections were analysed at two standard magnifications, 20× and 100×; the higher magnification was used to identify microclonal events within individual seminiferous tubules, whereas the lower magnification was more effective to scan entire tubular cross-sections for altered staining. We standardised several definitions to increase the objectivity of our findings. We defined a *cellular cluster* as comprising 3 or more adjacent cells within the seminiferous tubule with positive staining (Figure S1A, B i); by setting this low threshold for a cluster, we expected to maximise the sensitivity of detection, at the expense of a large number of false positive signals. Within or adjacent to the lumen of the seminiferous tubule, these cells could be present in either clumps or chains (Figure S1A i, ii), whereas adjacent to the basement membrane the cells had to be present as a clump (Figure S1A iii, iv), to distinguish them from normal spermatogonia. Clusters were flagged using OlyVIA-Viewer software for later retrieval.

Putative *microclones* were defined by the presence of independently flagged clusters in two or more sections (not necessarily serial, and using either the same or a different antibody), which showed clear physical contiguity based on the registration of the seminiferous tubules (Figure S1B ii, iii). Once a putative microclone had been identified, the corresponding cellular cluster was located in intermediate sections that had scored negative at the screening stage, in order to determine whether the cells were genuinely negative for the antigen (true negative), or were in retrospect positive but had been missed in the screen (false negative); we term this stage *post hoc analysis* (Figure S1B iii). Estimation was made of the minimum length and the minimum number of cells in each microclone. The minimum length was determined from the total number of 5 µm thick sections studied that contained the particular cluster; quantitation did not extend beyond the first and last positive sections. The minimum number of cells was determined as the number of cells expressing MAGEA4 (calculated where necessary by linear interpolation of the cell counts between MAGEA4-positive slides and assuming a cell diameter of 5 µm, equal to the slide thickness), including other markers only where these represented the last positive section in one or both directions.

Separately, a distinctly different class of microscopic lesions were defined as *immunopositive tubules*. These were identified at low magnification and were characterised by tubules having an overall darker MAGEA4 staining compared to their neighbours; this was independently confirmed by the identification of similar appearances for the contiguous tubules in nearby sections, stained either with MAGEA4, or a subset of the other antibodies. These immunopositive tubules were then visualised at higher magnifications to determine whether the darker appearance was due to stronger staining and/or the presence of additional cells.

The number of cross-sectioned tubules per section and the volume of tissue analysed was quantified manually. The tubular cross sections on MAGEA4-stained tissue were counted by flagging each tubule using the OlyVIA-Viewer software. The counts presented represent the average of three or more sections (Dataset S1). The volume of testis analysed was obtained by estimating the area per section (the same three sections for which tubules were counted) and multiplying this by the number of sections and thickness of each section (5 µm) (Dataset S1). To extrapolate the frequency per testis, a testis volume of 18 cm^3^ was assumed [39].

### Tracking of Tubules Using Three-dimensional (3D) Reconstruction

Further 5 µm sections of sample 1–1 were cut, and slides at intervals of 20 or 30 µm were stained with MAGEA4, digitally scanned and analysed as described above. A contiguous region containing a cluster of strongly immunopositive tubules was identified in 13 sections covering a distance of 300 µm and the selected region was imported into the 3D content creation suite Blender (http://www.blender.org/) as a series of sequential images. For each section image Blender “metaballs” were manually added to fill each tubule to the cell border and grouped to be associated with each image. When these section images were stacked on the *z*-axis, the metaballs coalesced forming a continuous structure. The metaballs were then converted to a mesh, and this was painted and rendered to highlight important structures.

## Results

Our approach to develop an immunohistochemical screen to visualise spermatogonial selection in human testes was informed by our recent study of spermatocytic seminoma [11], because this is hypothesised to represent the extreme consequence of mutation in adult spermatogonial cells resulting in an overt tumour [11], [15], [16], [17]. Based on these results, we chose five categories of antibody to screen for unusual cellular characteristics within the seminiferous tubules (see Table 2). These markers were (1) MAGEA4 as a robust indicator of all spermatogonial stages; (2) OCT2, SAGE1 and SSX for different stages of spermatogonial differentiation; (3) FGFR3 as a marker of spermatogonia, also known to be directly related to the process of selfish selection [11]; (4) Ki67, a marker of cellular proliferation; and (5) pAKT to explore downstream signal activation in positive tubules.

### Identification of Numerous Putative Microclones in the Testes of Elderly Men

**Figure 2:**
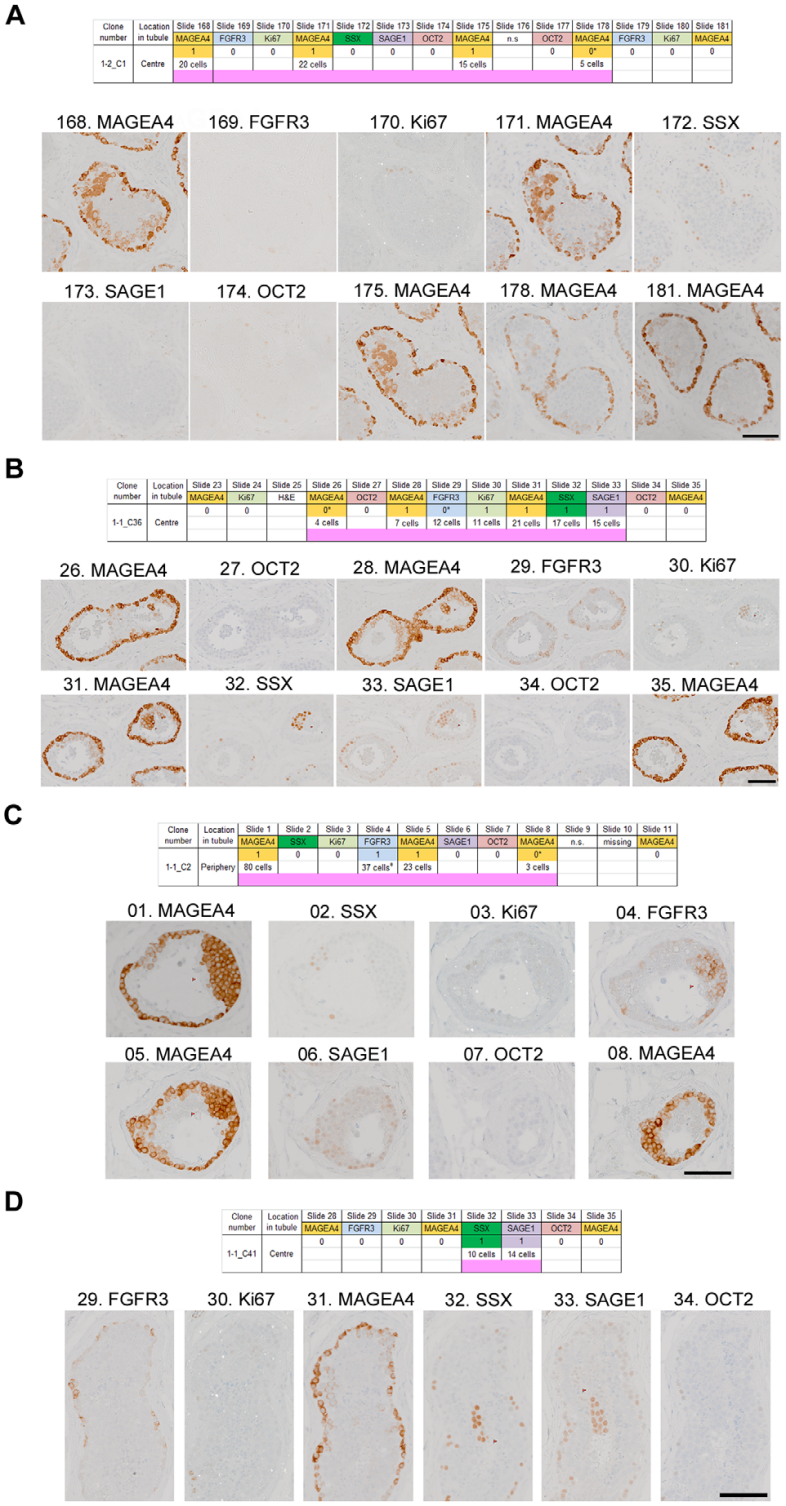
Examples of putative microclones with different antigenic profiles in samples 1–1 and 1–2. **A.** MAGEA4 positive only (microclone no: 1–2_C1): nuclear immunoreactivity to MAGEA4 was identified independently (tagged with flag) in sections no.168, 171 and 175. This cellular cluster is negative for five additional markers as shown. Weaker, cytoplasmic MAGEA4 staining of primary spermatocytes is also present. **B.** MAGEA4, SSX, SAGE1, FGFR3 and Ki67 positive (microclone 1–1_C36). Additional FGFR3 positivity was determined *post hoc*. **C.** MAGEA4 and FGFR3 (microclone 1–1_C2): a large cluster of cells occupying the periphery of the tubule expresses MAGEA4 and FGFR3 on adjacent serial sections, but is negative for SSX, Ki67, SAGE1 and OCT2. Further screening was not possible because section 01 was the first section of the tissue block. **D.** SSX and SAGE1 (microclone 1–1_C41): one of the few examples where a microclone was negative for MAGEA4 expression. In this case, the cluster of cells is positive for SSX and SAGE1 only. The specificity of all markers including MAGEA4 is confirmed by their expression in spermatogonia situated at the periphery of the tubule (internal positive control). Scale bars: 100 µm. Tables above each figure display the antigenic profile (positive  =  coloured box with 1 (independent identification), or 0* (*post hoc* identification); negative  =  white box with 0) and length (pink bar) of the microclone. n.s: not stained. Cell counts for each positive section are also detailed.

Initially we developed and refined our immunohistochemical screen based on the analysis of two separate testis blocks from a 71 year old man (sample 1, Table 1). We flagged *cellular clusters* (see Materials and Methods and Figure S1 for definitions) by independently screening different sections with six antibodies (MAGEA4, FGFR3, Ki67, OCT2, SAGE1 and SSX); then, by comparing the positions of flagged cells on adjacent sections, we asked whether any of these flagged clusters showed physical contiguity between the sections. We found, indeed, that we were regularly able to identify such contiguous clusters of cells: we considered that the independent identification of such clusters in different sections made it less likely that these were simple staining artefacts, and more likely that they represented examples of groups of cells with localised alteration in antigen expression, compatible with their identification as putative *microclones*. In two separate blocks (samples 1–1 and 1–2) from one testis (of total thickness 215 µm), each comprising ∼3000 seminiferous tubule cross-sections, we identified 84 microclones; 62 (74%) were positive for MAGEA4 alone, 20 (24%) expressed MAGEA4 and other combinations of antigens, and 2 (2%) expressed SAGE1 and SSX only (Table 3). Selected examples of each of these antigen combinations are shown in Figure 2A–D, which also illustrates the varying patterns of size and cellular morphology that we encountered in microclones. A comprehensive illustration of the 84 microclones identified in samples 1–1 and 1–2 is presented in Dataset S2.

**Table 3:** Immunohistochemical screening for microclones in testes of 6 men.

Sample-block number	No. of sections/approximate volume screened	Number of putative microclones	Number (%) MAGEA4 only	Number (%) MAGEA4+ other^a^	FGFR3 positive (primary/*post hoc*)	Microclone density (microclones per mm^3^)	Immunopositive tubular cross-sections (%)
1–1	27/59.3 mm^3^	45	27 (60%)	16 (+2^b^) (40%)	5/5	0.76	1.1
1–2	18/34.2 mm^3^	39	35 (90%)	4 (10%)	0/0	1.14	0
2–1	24/28.0 mm^3^	61	14 (23%)	46 (+1^b^) (77%)	34/1	2.17	1.9
2–2	21/13.7 mm^3^	NA	NA	NA	NA	NA	1.0
3–1	23/13.0 mm^3^	89	6 (7%)	83 (93%)	73/6	6.83	5.4
3–2	21/21.4 mm^3^	NA	NA	NA	NA	NA	1.7
4–1	19/16.6 mm^3^	6	4 (67%)	2 (33%)	0/1	0.36	0
5–1	18/7.9 mm^3^	4	1 (25%)	3 (75%)	2/1	0.51	0
6–1	19/11.4 mm^3^	7	5 (71%)	2 (29%)	0/1	0.61	0

a Testes were stained with different combinations of antibodies; see Dataset S1.

b Microclones negative for MAGEA4 but positive for 2 or more other antigens. NA =  Samples 2–2 and 3–2 were not analysed for the presence of cellular clusters/microclones.

The number of cellular clusters identified with each antibody, and the proportion of clusters that were found to represent microclones, varied considerably: MAGEA4 showed both the highest number of clusters per section (1176 in 18 sections) and the highest proportion of clusters involved in forming microclones (190/1176 = 16%) (Dataset S1). Most microclones were very small, all except two comprising fewer than 200 cells (Figure S2); smaller size was significantly associated with staining for MAGEA4 only (Mann-Whitney U test, *P = *0.029). We were particularly interested in the identification of microclones positive for FGFR3 staining, because of the direct functional link with the hypothesis of selfish spermatogonial selection [11]; 10 microclones were FGFR3 positive (5 of these by *post hoc* analysis only, see Materials and Methods), these included the two largest events comprising an estimated 287 (Figure 2C) and 356 cells, truncated in both cases because the end of the tissue block was reached.

**Figure 3:**
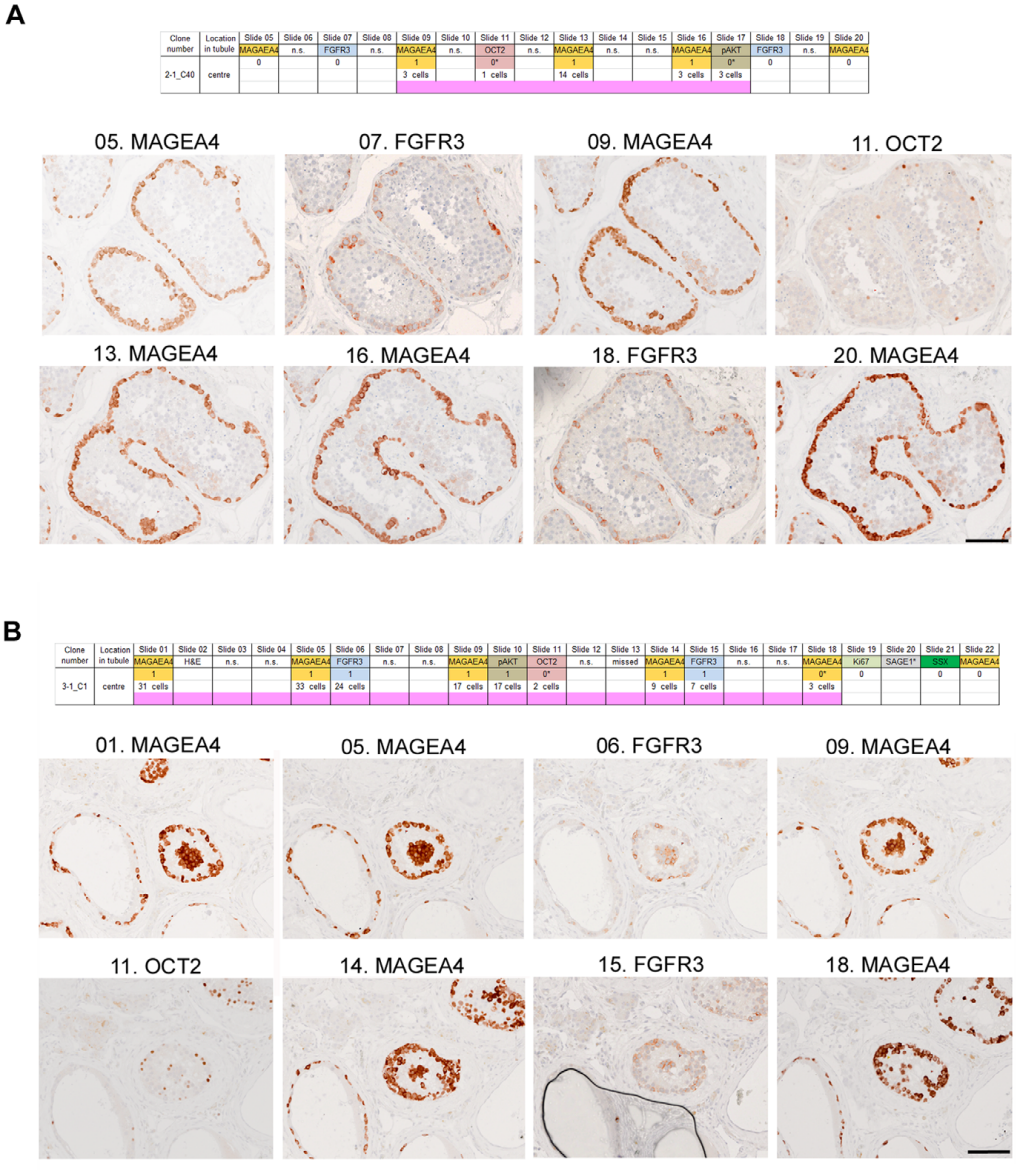
Examples of microclones in samples 2–1 and 3–1. **A.** Microclone 2–1_C40, located away from the basal lamina, is positive for MAGEA4 and OCT2 (single cell identified *post hoc*) but not for FGFR3. **B.** Microclone 3–1_C1, located within the lumen of the tubule is positive for MAGEA4, FGFR3 and OCT2 (2 cells identified *post hoc*). Panels above the set of images show the markers used to stain the sections and the analysis of the results, using the same scheme as in legend to Figure 2. Scale bars: 100 µm.

Having established a robust methodology to survey testes for microclones and identified many such events within two different regions of a single testis, we then screened portions of testes from 5 other men aged 69 to 78 years (Table 1) to determine whether these findings were peculiar to the original sample, or whether similar features could be identified more generally in testes of elderly men. Figures 3A and B illustrate further examples of microclones from two different testes, and Table 3 summarises the findings. Although the apparent prevalence of these microclones varied at least 10-fold between testes (Table 3), their occurrence in those aged over 69 years seems to be a widespread and general phenomenon.

### Tubules Showing Generally Enhanced MAGEA4 Immunoreactivity (Immunopositive Tubules)

**Figure 4:**
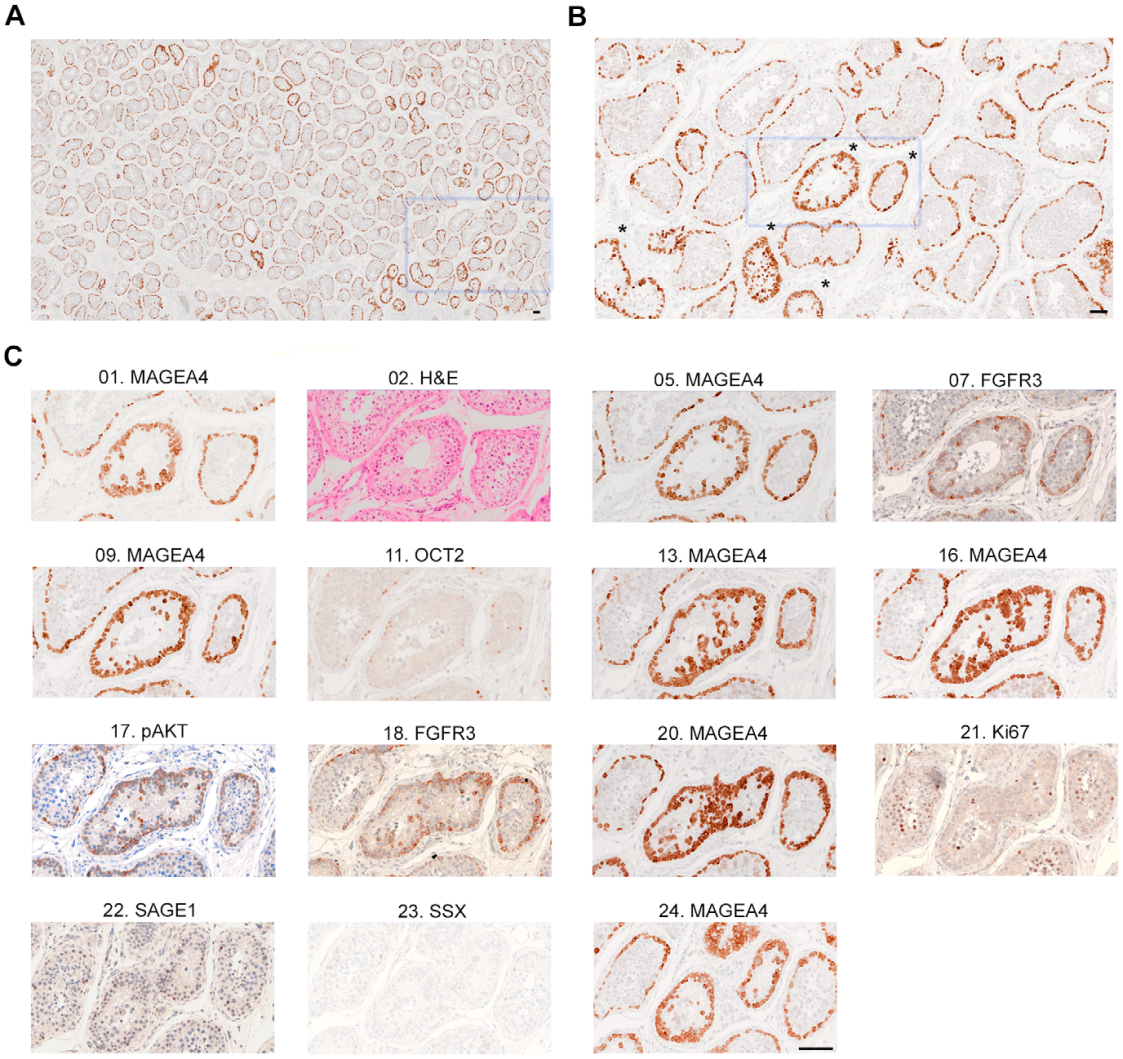
Immunopositive tubules in sample 2–1. **A.** Under low power magnification of section 05, several clusters of two or more tubules with strong MAGEA4 staining are visible. **B.** Higher power magnification of the boxed region in **A.** Five tubules with stronger MAGEA4 staining (*) are distinguishable from their neighbouring tubules with normal levels of MAGEA4 staining. In normal tubules, MAGEA4 stains the nucleus and cytoplasm of the spermatogonia on the basal lamina. In the immunopositive tubules additional MAGEA4-positive cells are present, forming a double row. **C.** Serial sections spanning 115 µm display consistently stronger staining for MAGEA4, FGFR3 and pAKT. Clusters of MAGEA4 positive cells are present in the lumen of this immunopositive tubule. No differences in staining for OCT2, Ki67, SAGE1 or SSX are observed. H&E staining (section 02) shows that the tubule in the centre contains few spermatocytes and no spermatids, whereas the tubule on the right hand side contains both spermatocytes and spermatids. A higher resolution image of the H&E staining is shown in Figure S4A. Scale bar: 100 µm.

**Figure 5:**
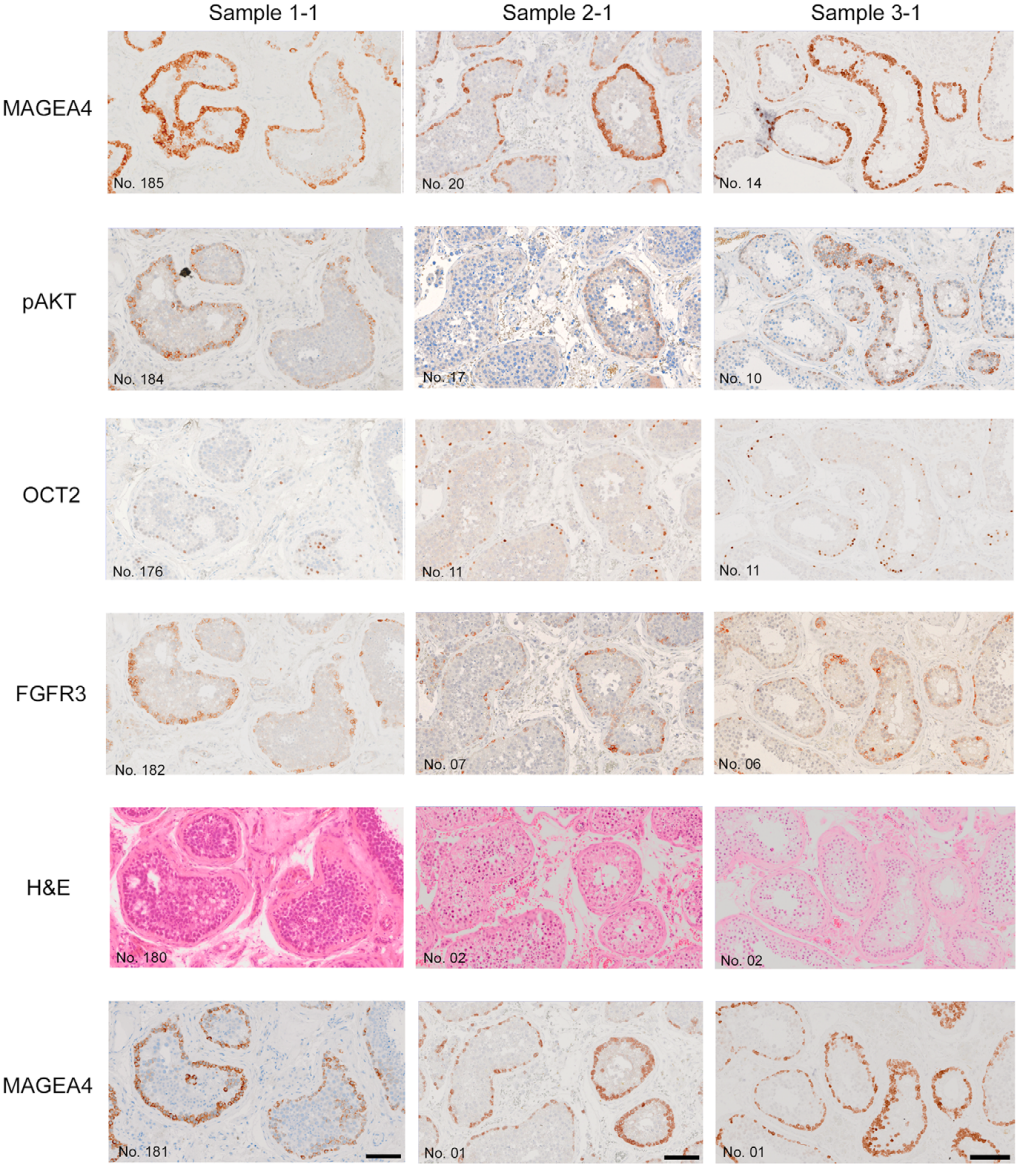
Examples of immunopositive tubules in samples 1–1, 2–1 and 3–1. Immunopositive tubules in three testis samples identified by staining with MAGEA4 also show enhanced staining for FGFR3 and pAKT. There is no difference in intensity of OCT2 staining, although some of the additional cells in sample 3–1 are OCT2 positive. H&E staining reveals that spermatocytes and spermatids are present in samples 1–1 and 2–1, but in sample 3–1 only spermatogonia and Sertoli cells are present (higher resolution images of the H&E panels are shown in Figure S4B). Scale bars: 100 µm. Note that sections are not all shown in sequential order.

**Figure 6:**
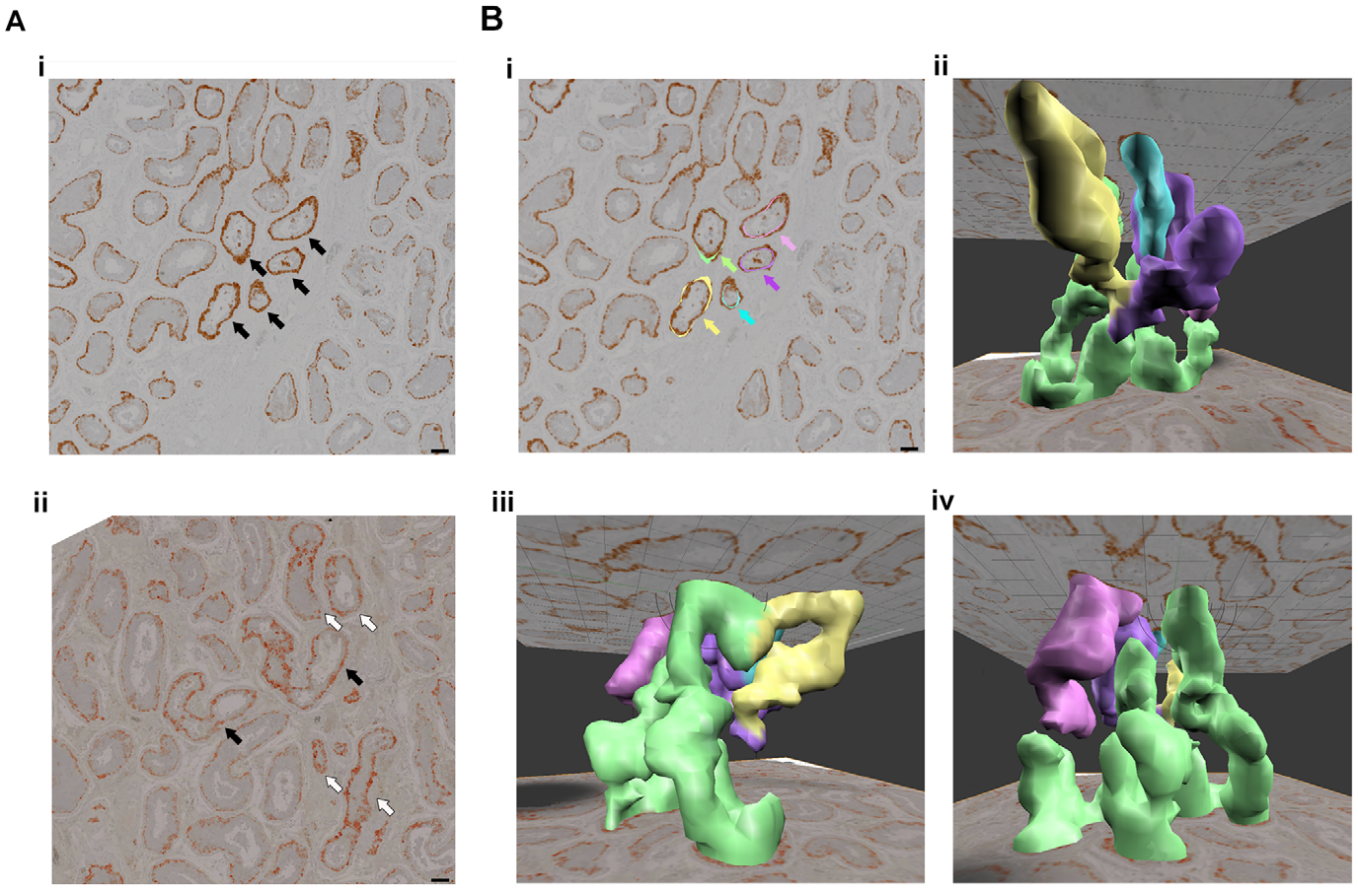
3D reconstruction of immunopositive tubules in sample 1–1. **A.** (**i**) Five immunopositive tubular cross-sections (black arrows) in MAGEA4-stained section 51. The immunopositive tubules were followed by staining further sections at intervals of 4 or 6 slides (20 and 30 µm, respectively), until section 111. (**ii**). Although other immunopositive tubular cross-sections are present in section 111 (white arrows) and the intermediate sections, only those which could be traced back to section 51 (black arrows) were included in the reconstruction. Scale bars: 100 µm. **B.** 3D reconstruction of the immunopositive tubule. (**i**) The five immunopositive tubular cross-sections in section 51 were colour coded (green, pink, purple, blue and yellow arrows). The resolved 3D structure (with section 51 at top and section 111 at bottom) reveals that 4 of the 5 immunopositive cross-sections in section 51 (blue, purple, yellow and green) are part of the same tubule (joining where the colour margins blur together)(**ii**, **iii**, **iv**). Although they are in close proximity, it could not be demonstrated that the pink and green tubules are contiguous (**iv**). As the structure is highly convoluted, for clarity the scale of the *z*-axis has been increased 3-fold. A movie displaying the rotating 3D structure (shown to scale) is available as Video S1 (for description see Text S1).

Whilst examining sections under low power magnification, we noticed that occasional tubules showed a distinct staining pattern in which the entire circumference exhibited enhanced immunopositivity for MAGEA4; moreover, several such tubules (which we term *immunopositive tubules*) often clustered in a localised part of the section and/or lay adjacent to each other (Figure 4 A, B). At higher power, we established that the increased MAGEA4 staining appeared attributable to the presence of additional immunopositive cells. These cells were mainly adjacent to the spermatogonia lying on the basal lamina, although in some cases additional cells were located higher in the germinal epithelium in a more lumenal position. In three of the testes examined (samples 1–1, 2–1 and 3–1) these immunopositive tubules were readily apparent (Figures 4, 5), but no such tubules were identified in the remainder (samples 4–1, 5–1, 6–1). By staining further sections with MAGEA4, we found that individual immunopositive tubules could be followed across all sections analysed in samples 2–1 and 3–1 (corresponding to block thicknesses of 115 and 110 µm, respectively) and up to 295 µm of tissue depth in sample 1–1. Despite the convoluted nature of the seminiferous epithelium, we could in some cases follow the U-turns taken by tubules using three-dimensional reconstruction; this analysis showed that neighbouring tubular cross-sections were part of the same unit and that these strong staining appearances were likely to encompass long contiguous segments of individual tubules, corresponding up to at least a millimetre of the tubular length (Figure 6, Text S1and Video S1; see also Figure S3).

We next examined the patterns of reactivity of these MAGEA4-immunopositive tubules with the other antibodies in our survey. We found that the tubules showing increased reactivity for MAGEA4 also showed enhanced staining for FGFR3, but there was no difference in the staining intensity of SSX, SAGE1, Ki67 or OCT2 (Figures 4C, 5, S3). To determine whether this was associated with signal activation, we performed immunohistochemistry with an antibody to pAKT on selected sections. We found that tubules reacting strongly with MAGEA4 and FGFR3 also showed enhanced pAKT immunoreactivity (Figures 4C, 5, S3). Haematoxylin and eosin (H&E) staining of the sections showed that a proportion of the cells within the immunopositive tubules still exhibited active spermatogenesis, and differentiation at least to the spermatocyte stage was noted in tubules of samples 1–1 and 2–1, allowing us to exclude the possibility that we had inadvertently detected ISS in these samples (Figures 4C, 5, S4). In sample 3–1, the majority of the immunopositive tubules had arrested spermatogenesis at the spermatogonial stage. A subset of the additional cells in the immunopositive tubules were positive for OCT2 (Figure 5), a marker of A_dark_ spermatogonia [15], suggesting that the population of staining cells includes both A_dark_ and A_pale_ spermatogonia. No differences in cellular proliferation (inferred from Ki67 expression) between normal and immunopositive tubules were noted (Figures 4, S3). The majority of tubules were visualised as cross-sections but occasionally longitudinal tubular sections measuring up to 1.4 mm were apparent (Figure S3).

The immunopositive tubules represented 1.1%, 1.9% and 5.4% of the total number of tubular cross-sections in samples 1–1, 2–1 and 3–1, respectively (Table 3). To establish whether this phenomenon is a general feature in certain individuals, we screened additional sections taken from different blocks (samples 1–2, 2–2 and 3–2) with MAGEA4. No immunopositive tubules were noted in sample 1–2 and in each of the other two additional samples, the frequency of immunopositive tubules was lower (1.0% and 1.7% in samples 2–2 and 3–2, respectively), than in the block initially examined. This indicates that there are marked regional variations in the density of the immunopositive tubules within individual testes (Table 3).

## Discussion

Our primary objective was to seek the immunohistochemical correlates of selfish spermatogonial selection, which we have proposed underlies the phenomenon of paternal age-effect mutations [6]. We concentrated on testes from elderly men, as we reasoned that mutational events would be more abundant, and therefore more readily detected, in this age group. Although the histopathology of testes has been intensively studied owing to its importance in infertility and tumourigenesis [13], [40], we are unaware of any previous attempt to demonstrate immunohistochemical evidence for clonal expansion in normal testes. However, as described in the Introduction, there is strong molecular genetic evidence in the case of four particular mutations in *FGFR2*, *FGFR3* and *RET* that such clones must occur [20], [21], [22], [24], and the histopathological literature supplies three observations that might support the occurrence of such mosaics or clones in the testis [14], [15], [28], [31].

We identified two main phenomena: (1) putative *microclones* of spermatogonial cells within isolated tubular cross-sections and (2) a more general abnormal staining pattern within a subset of adjacent tubular cross-sections (MAGEA4-*immunopositive tubules*). We found that clusters of cells with distinctive antigenic characteristics could readily be identified in all 6 testes examined and, in the case of MAGEA4-positive clusters, nearly one fifth of these could be independently located as part of a contiguous cluster on a nearby section. Greater confidence could be placed in the interpretation of these appearances as representing genuine microclonal events when the clusters had been independently identified using more than one antibody; this requirement was fulfilled in 159 of the 251 cases (63%) (Table 3, Dataset S1), several of which are illustrated (Figures 2B–D, 3, Dataset S2).

Overall, these microclones tended to be small (93% comprised fewer than 200 cells) but frequent (by extrapolation, 6,500–123,000 events per testis), and may be similar to the “plaques” previously described in electron microscopic images [28]. However, these size and frequency characteristics do not match the expectation of mutational clones previously described by DNA studies of Apert, Men2B and achondroplasia mutations, which, based on the number of mutant molecules quantified in each clustered set of testis pieces, could involve anything from ∼100 (the lower limit of detection) to over 1 million cells [20], [21], [22], [24]. Hence, although a proportion of these microscopically identified events may indeed represent mutational microclones, their interpretation in terms of current data on selfish spermatogonial selection remains uncertain. It is recognised that formalin fixation (although suitable for immunohistochemistry) is not ideal for detailed examination of cellular morphology in seminiferous tubules [41], [42], although the use of maturation stage-specific markers (e.g. OCT2) partly obviates this problem. A re-analysis of this issue using a preferred fixative (for example Bouin’s) would be advantageous to elucidate the cellular nature of these phenomena.

In 3 of the 6 testes analysed, we noted distinctly stained tubules with additional positively staining cells adjacent to the spermatogonia on the basal lamina, forming a double layer. Rather consistently, this increased staining was also apparent using a functional marker, pAKT, suggesting that this was associated with activation of the phosphoinosidide-3-kinase/AKT signalling pathway downstream of RAS activation, which has been directly implicated in spermatogonial self-renewal and may confer a proliferation and/or survival advantage [6], [19], [43]. Owing to the lobular organisation of the testis and the convoluted architecture of the tubules (Figure 1), it might be anticipated that the close proximity of the immunopositive tubules within a section was indicative of their contiguous nature. In several instances, it could be established by following U-turns in their course, that closely spaced immunopositive cross-sections were indeed contiguous and belonged to the same tubule (Figure 6 and Video S1). These immunopositive tubules could also be tracked over long distances and were observed as far as 59 slides apart (corresponding to 295 µm of block thickness), suggesting that the total extent of these immunopositive events could cover distances up to a few mm of tubular length.

**Figure 7:**
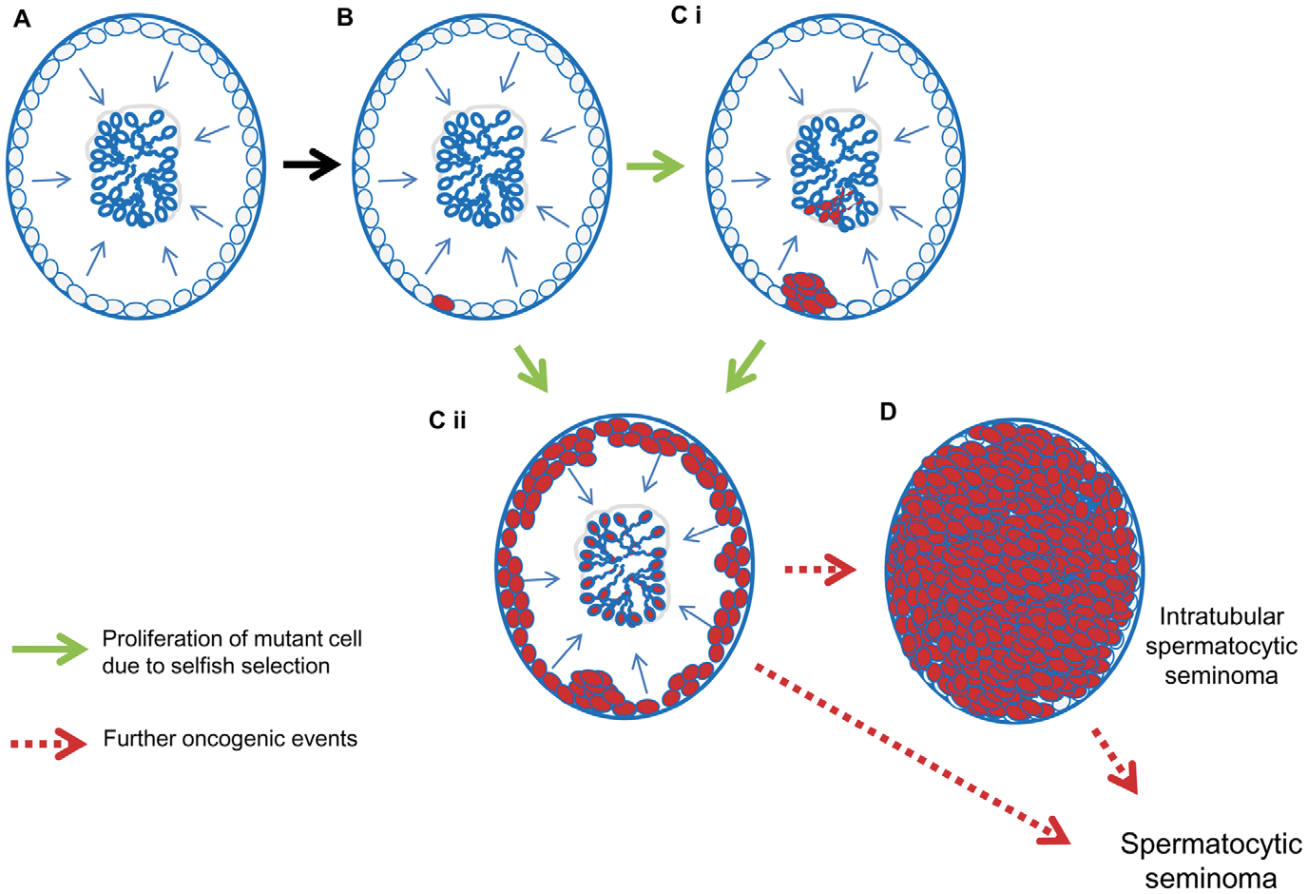
Proposed model linking the immunohistochemical observations to clonal expansion of PAE mutations in seminiferous tubules. **A.** Tubule undergoing normal spermatogenesis: spermatogonia (grey) adjacent to basal lamina (blue line) proliferate and differentiate (arrows) to spermatozoa in the lumen. **B.** An activating PAE mutation arises in a single spermatogonial cell (red cell). **C.** Altered cellular signalling associated with the mutation confers a proliferative or survival advantage to the mutant cell, leading to clonal expansion of spermatogonia and relative enrichment of mutant sperm. The clonally expanded cells retain the immunohistochemical features of the originating cell (such as MAGEA4 and FGFR3) and may also have altered markers of signal activation (pAKT) - forming localised microclones (**i**) or expanding around the circumference and along the tubule (**ii**). Process (ii), compatible with the appearance of the immunopositive tubules identified in this study, would account for the distribution and number of mutant cells containing PAE mutations previously determined by DNA studies [20], [21], [22], [24]. **D.** In rare cases, additional mutational events may arise and lead to the formation of spermatocytic seminoma, possibly via an ISS intermediate state.

Although the appearance of a ‘double layer’ of spermatogonia has been observed by electron microscopy previously in testes of elderly men, this was assoociated with spermatogenesis arrest and was proposed to result from the failure of type A spermatogonia to differentiate [28]. In our study, H&E staining revealed the presence of spermatocytes and spermatids in immunopositive tubules of two of three testes, suggesting that these tubules were still capable of spermatogenesis (Figures 4C, 5, S4). The morphology of the immunopositive tubules was noticeably different from that described for ISS, in which the entire tubule, including the lumen, is replaced by round cells of varying sizes. However, the anatomical distribution of affected tubules within the tissue section is reminiscent of ISS and is consistent with the intratubular spread of a mutant cell population characterised by abnormal, enhanced signal activation (Figure 7). The likely clonal nature of the immunopositive tubules is supported by lineage tracing experiments of spermatogonial stem cells in the mouse testis, in which stochastically labelled cells expanded over time along the seminiferous tubules over distances up to 8.5 mm [44]. In the context of tumourigenesis, Lee et al. [19] observed that colonies resulting from transplantation of germ cells transfected with an activating *Hras* mutation also followed a tubular course. Moreover, immunohistochemical and H&E analysis of cross-sections of tubules populated by germ cells over-expressing activating Hras showed that these murine tubules were characterised by a double layer of spermatogonia, reminiscent of the phenomenon we describe in human testes [45].

Based on our current understanding of selfish spermatogonial selection, Figure 7 illustrates the predicted consequences of PAE mutations within tubular cross-sections. This model shows how the topographic staining patterns we observe within immunopositive tubules are compatible with the proposed effects of selfish selection. This raises the question as to how reliably the immunohistochemical phenotype of a group of cells may reflect an underlying clonal mutational origin. There are many instances in which this has proved to be the case. Selected examples include studies in which the cellular distribution of staining with an antibody to p53 in skin was shown to correlate with p53 mutational status [46], [47]; the correlation of change in histopathological characteristics and tumour mutational load revealed by deep sequencing [48]; and, directly relevant to this work, the demonstration in myelodysplastic syndrome that positivity for an FGFR3 antibody correlated with the presence of a t(4;14) translocation involving *FGFR3* on chromosome 4 [49]. On the other hand, there are also many instances where the correlation is less straightforward; this is illustrated by the role of FGFR3 in superficial urothelial carcinoma and spermatocytic seminoma, for both of which mutations and antibody positivity are described, but the correlation between the two is not absolute [11], [50]. Some apparently clonal protein/antigen expression patterns arise through epigenetic or post-translational modifications rather than genetic mutation [51], and in this regard it should be noted that MAGEA4, the best marker both of spermatogonia and of microclones, is proposed to suppress transcription induced by p53 [52], and is itself affected by promoter methylation [53]. The anti-MAGEA4 antibody (clone 57B) used in this study recognises primarily antigen A4 but also A3 [54], so it is possible that the increased immunopositivity of clonally proliferating spermatogonia may reflect changes in relative expression of these two antigens. Some of the appearances that we have described could be normal variations involving regular turnover of stochastic events in the testis [19], [44] or represent the elimination of displaced germ cell clumps by sloughing off, or might arise as preservation artefacts, for example, from squeezing of the testis during surgery.

In summary, although these concerns caution against over extrapolation of our results, we have clearly demonstrated that variation in the number and spatial localisation of spermatogonia within testicular seminiferous tubules of elderly men is likely much more widespread and prevalent than has hitherto been suspected. This provides a prelude to documenting these phenomena more broadly, for example asking how the frequency of microclones and immunopositive tubules varies with age or position within the testis, and to what extent it reflects stochastic processes. Most importantly, the reagents and techniques described here will help to pave the way for a more direct search, for example using microdissection of immunopositive tubules followed by genetic characterisation, for evidence of mutational clonality in individual seminiferous tubules identified by their distinctive immunohistochemical features.

## Supporting Information

Figure S1 **Criteria for identification of cellular clusters (A) and putative microclones (B).**
(TIF)

Figure S2 **Distribution of the minimum number of cells of each microclone in relation to the expression of markers for samples 1–1 and 1–2.**
(TIF)

Figure S3 **Longitudinal section of an immunopositive tubule in sample 3–1.**
(TIF)

Figure S4 **H&E staining of immunopositive tubules in** **Figures 4** **and** **5** **.**
(TIF)

Dataset S1 **Staining strategy and analysis of microclones and immunopositive tubules.**
(XLS)

Dataset S2 **84 microclones identified in samples 1–1 and 1–2.**
(PDF)

Text S1 **Legend for Video S1.**
(DOC)

Video S1 **Three-dimensional reconstruction of an immunopositive tubule.**
(MP4)
