# Supplementary figures and images for: Selfish Spermatogonial Selection: Evidence from an Immunohistochemical Screen in Testes of Elderly Men
Source: PLoS One. 2012 Aug 6;7(8):e42382. doi: 10.1371/journal.pone.0042382 (PMC3412839; doi:10.1371/journal.pone.0042382)

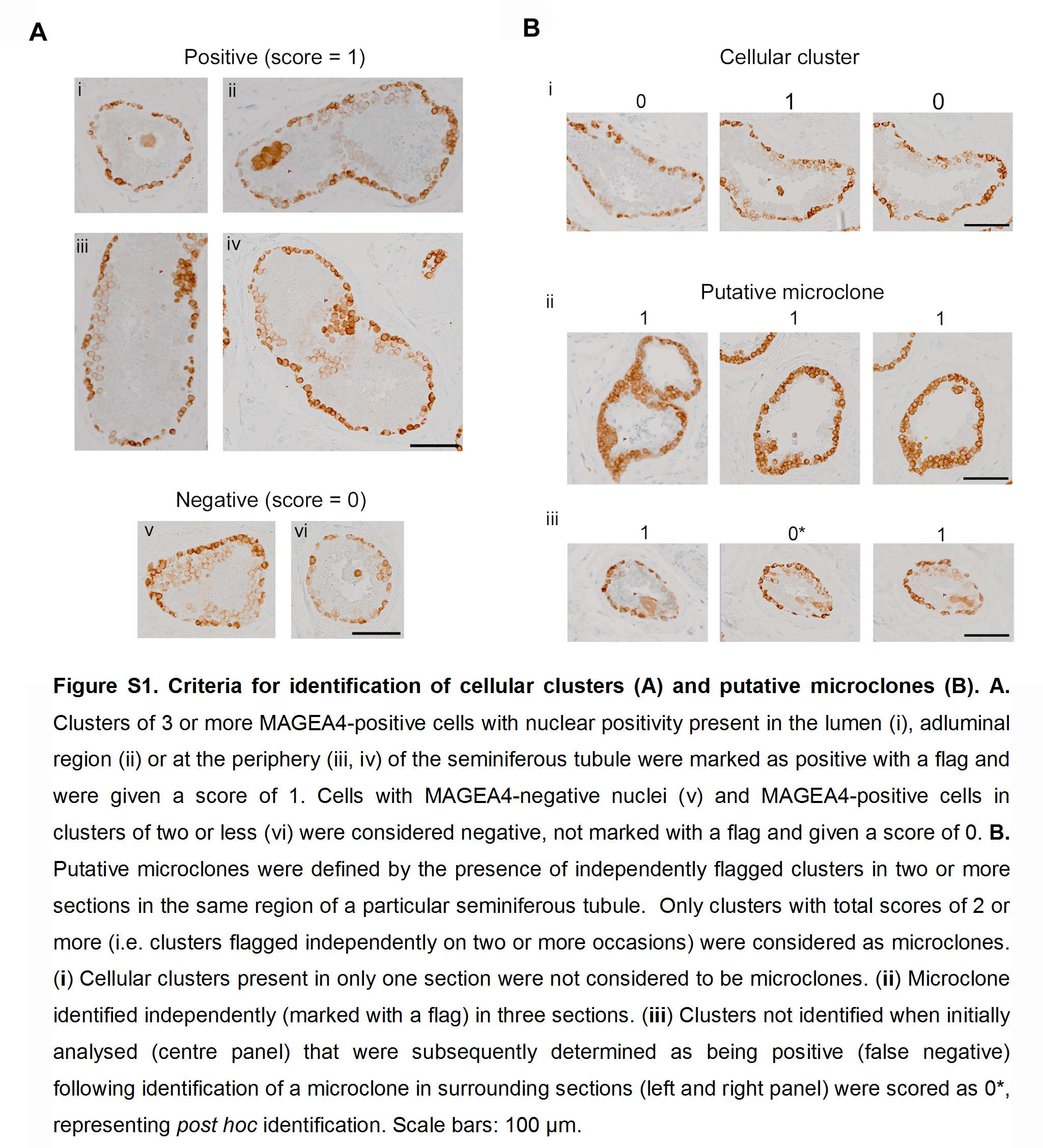

Supplement: Figure S1 — Criteria for identification of cellular clusters (A) and putative microclones (B). (TIF) [file pone.0042382.s001.tif]

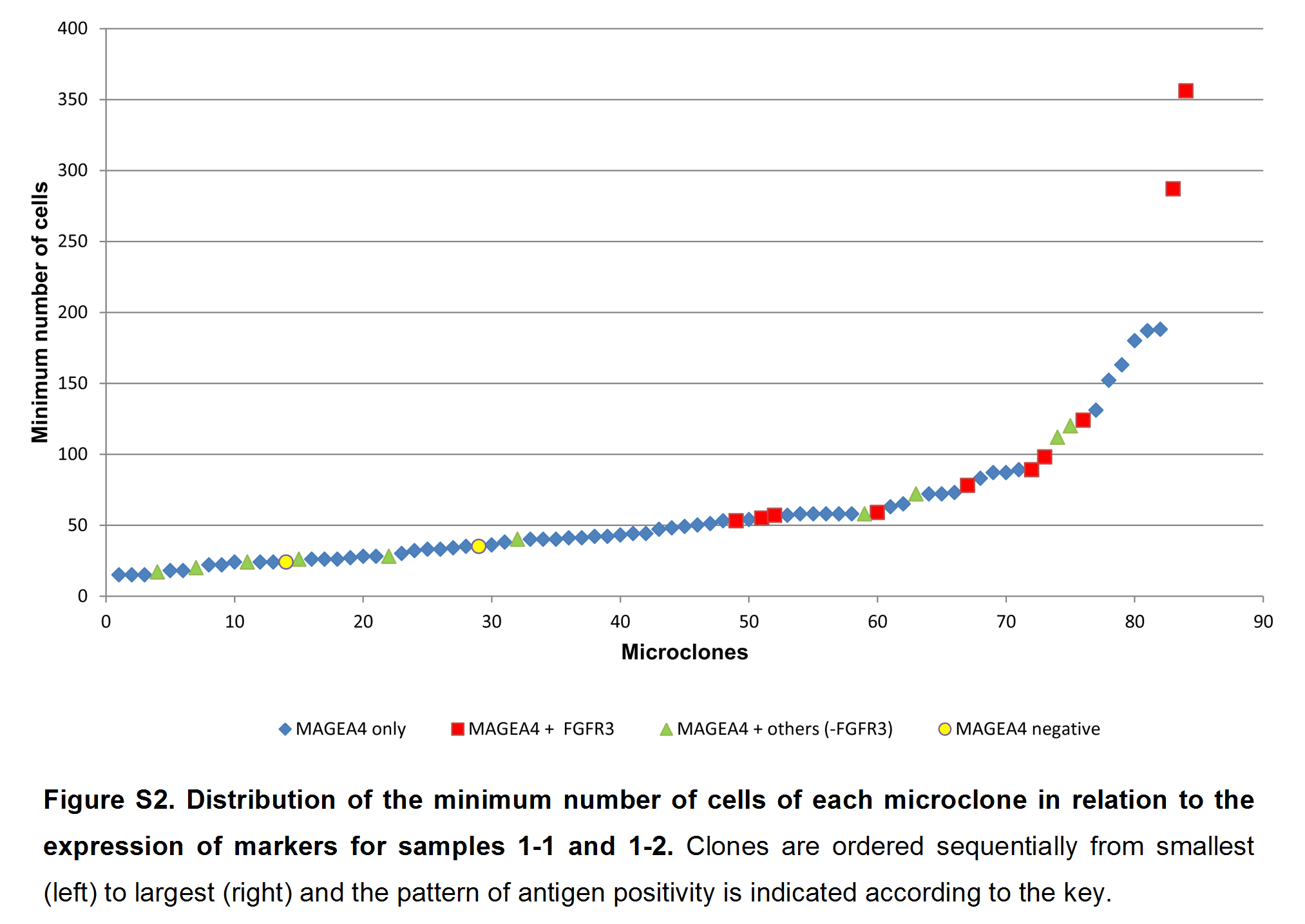

Supplement: Figure S2 — Distribution of the minimum number of cells of each microclone in relation to the expression of markers for samples 1–1 and 1–2. (TIF) [file pone.0042382.s002.tif]

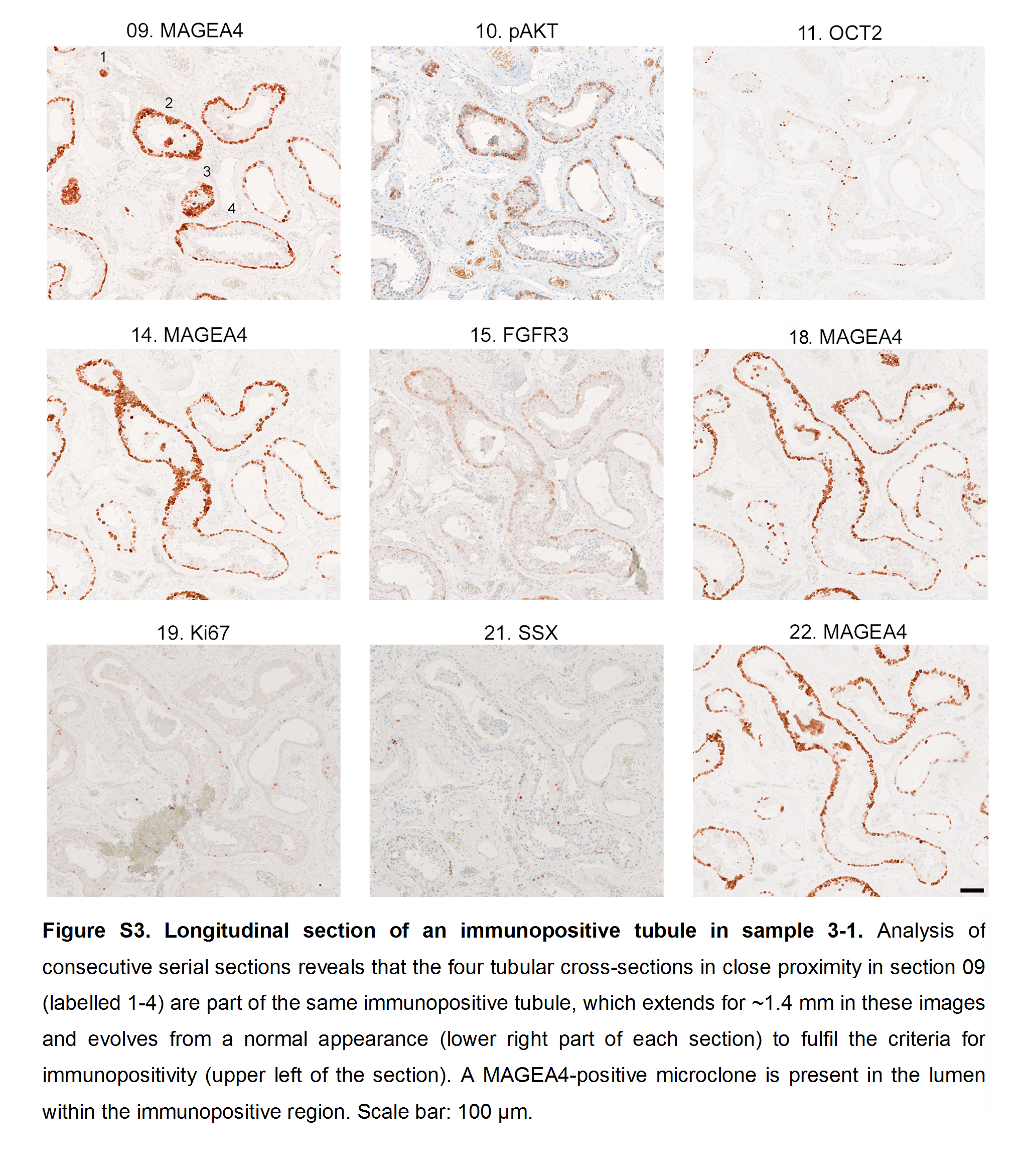

Supplement: Figure S3 — Longitudinal section of an immunopositive tubule in sample 3–1. (TIF) [file pone.0042382.s003.tif]

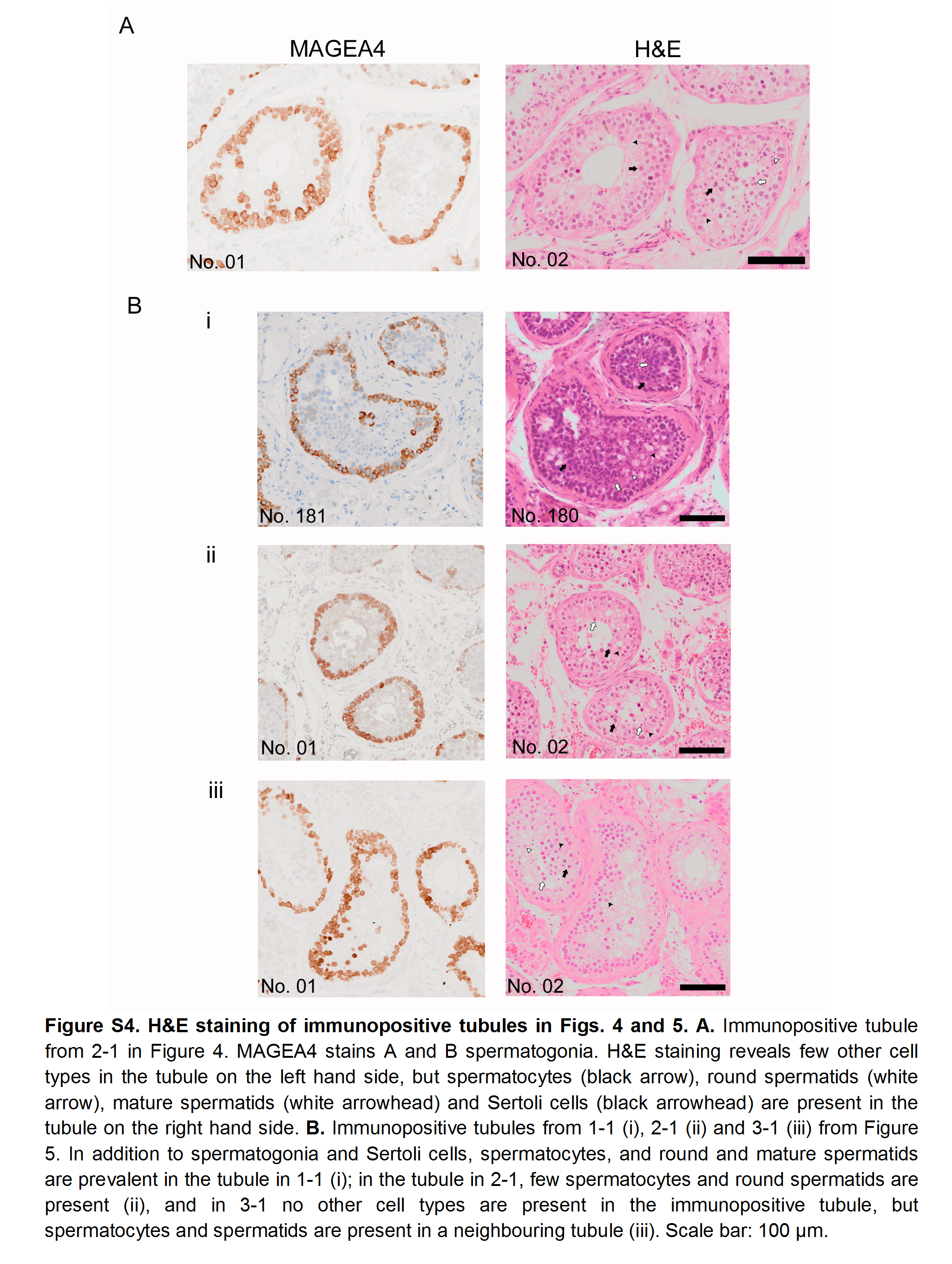

Supplement: Figure S4 — H&E staining of immunopositive tubules in Figures 4 and 5 . (TIF) [file pone.0042382.s004.tif]
