# Supplementary material for: Selfish Spermatogonial Selection: Evidence from an Immunohistochemical Screen in Testes of Elderly Men
Source: PLoS One. 2012 Aug 6;7(8):e42382. doi: 10.1371/journal.pone.0042382 (PMC3412839; doi:10.1371/journal.pone.0042382)

Dataset S2. Illustration of the 84 microclones identified in samples 1-1 and 1-2.
A. Tables displaying the antigenic profile of the microclone and cell counts for each positive section, using the same scheme as in legend to Figure 2. **B.** Panel of images displaying the staining of each microclone. Independently identified cellular clusters are tagged with a flag; absence of a flag indicates that no cluster is present (if allocated a score of 0 in the table above) or that clusters were identified in the *post hoc* analysis (if allocated a score of 0* in the table above). Scale bars: 100 µm.

Clone no. 1-1_ C1

A

Clone no.	Location	Slide 1	Slide 2	Slide 3	Slide 4	Slide 5	Slide 6	Slide 7	Slide 8	Slide 9	Slide 10	Slide 11	Minimum number of cells
		MAGEA4	SSX	Ki67	FGFR3	MAGEA4	SAGE1	OCT2	MAGEA4	n.s.	missing	MAGEA4	
1-1_C1	Centre	1	0	0	1	1	0*	0	1			NA	356
		38 cells			30 cells	62 cells	30 cells		22 cells				
		40 µm											

B

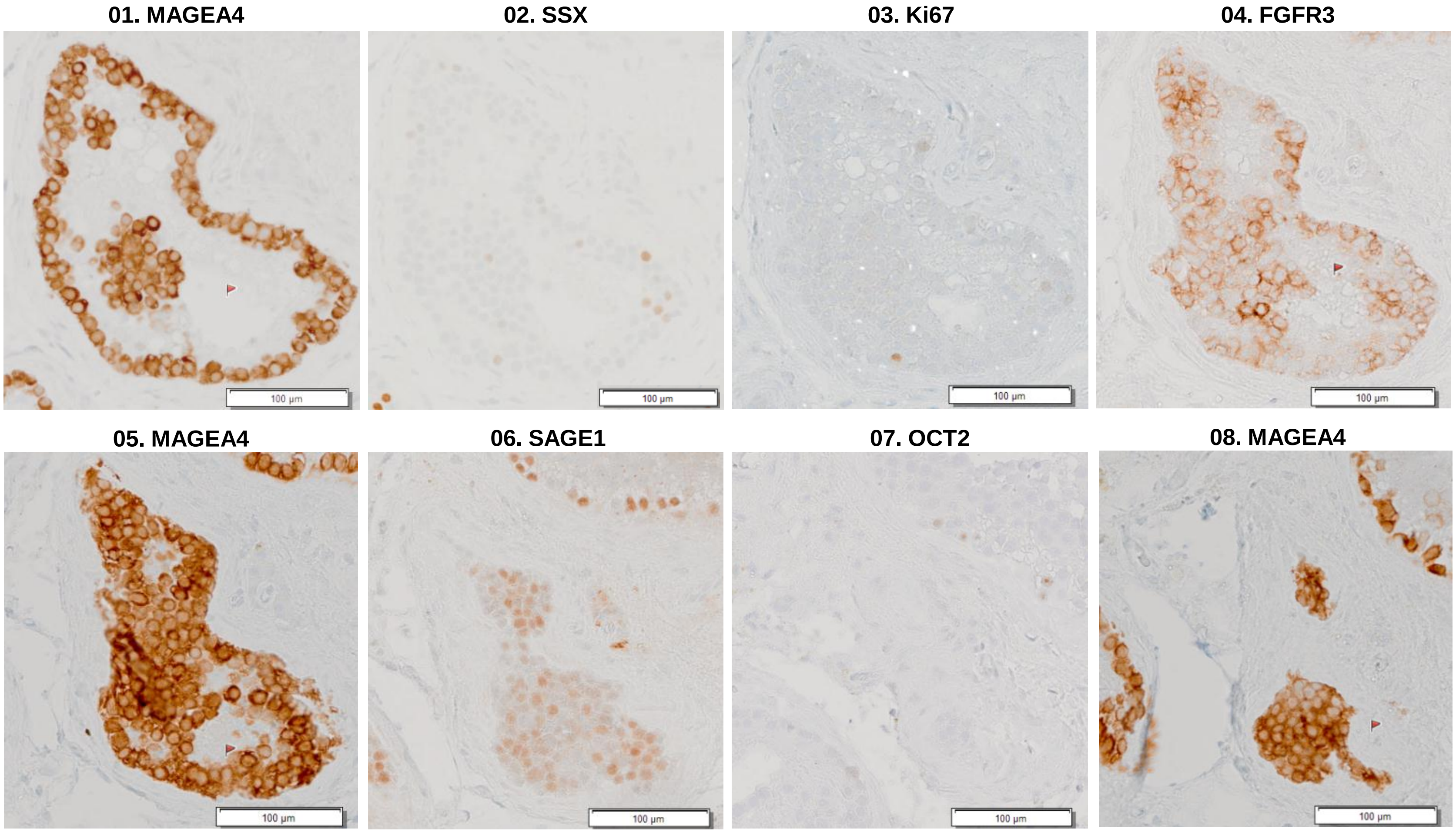

Clone no. 1-1_C2

A

Clone no.	Location	Slide 1	Slide 2	Slide 3	Slide 4	Slide 5	Slide 6	Slide 7	Slide 8	Minimum number of cells
		MAGEA4	SSX	Ki67	FGFR3	MAGEA4	SAGE1	OCT2	MAGEA4	
1-1_C2	Periphery	1	0	0	1	1	0	0	0*	287
		80 cells			10 cells	23 cells			3 cells	
		40 µm								

B

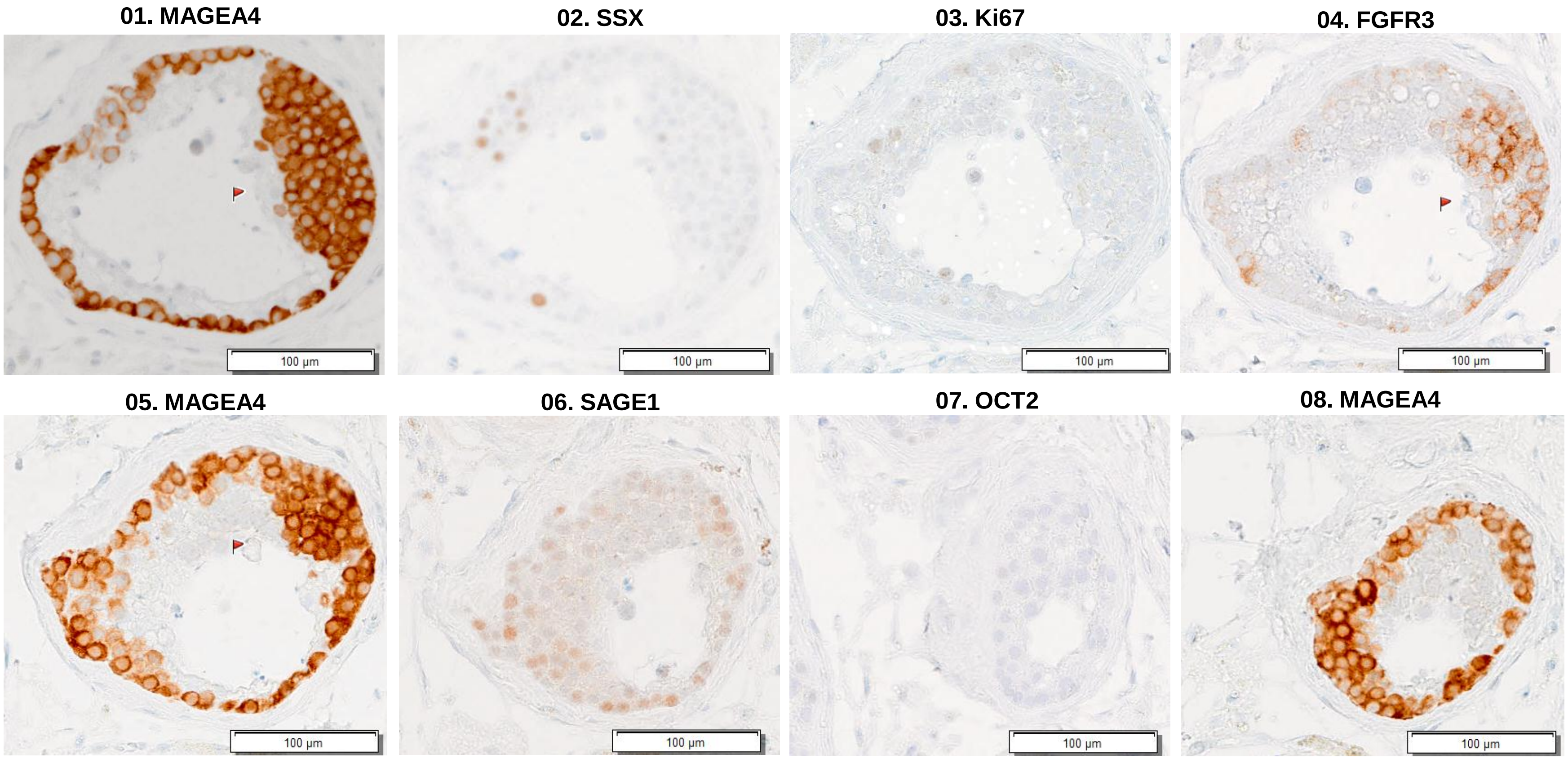

Clone no. 1-1_C3

A

Clone no.	Location	Slide 1	Slide 2	Slide 3	Slide 4	Slide 5	Slide 6	Slide 7	Slide 8	Minimum number of cells
		MAGEA4	SSX	Ki67	FGFR3	MAGEA4	SAGE1	OCT2	MAGEA4	
1-1_C3	Near Periphery	1	0	0	0	1	0	0	0*	58
		9 cells				9 cells			2 cells	
		40 µm								

B

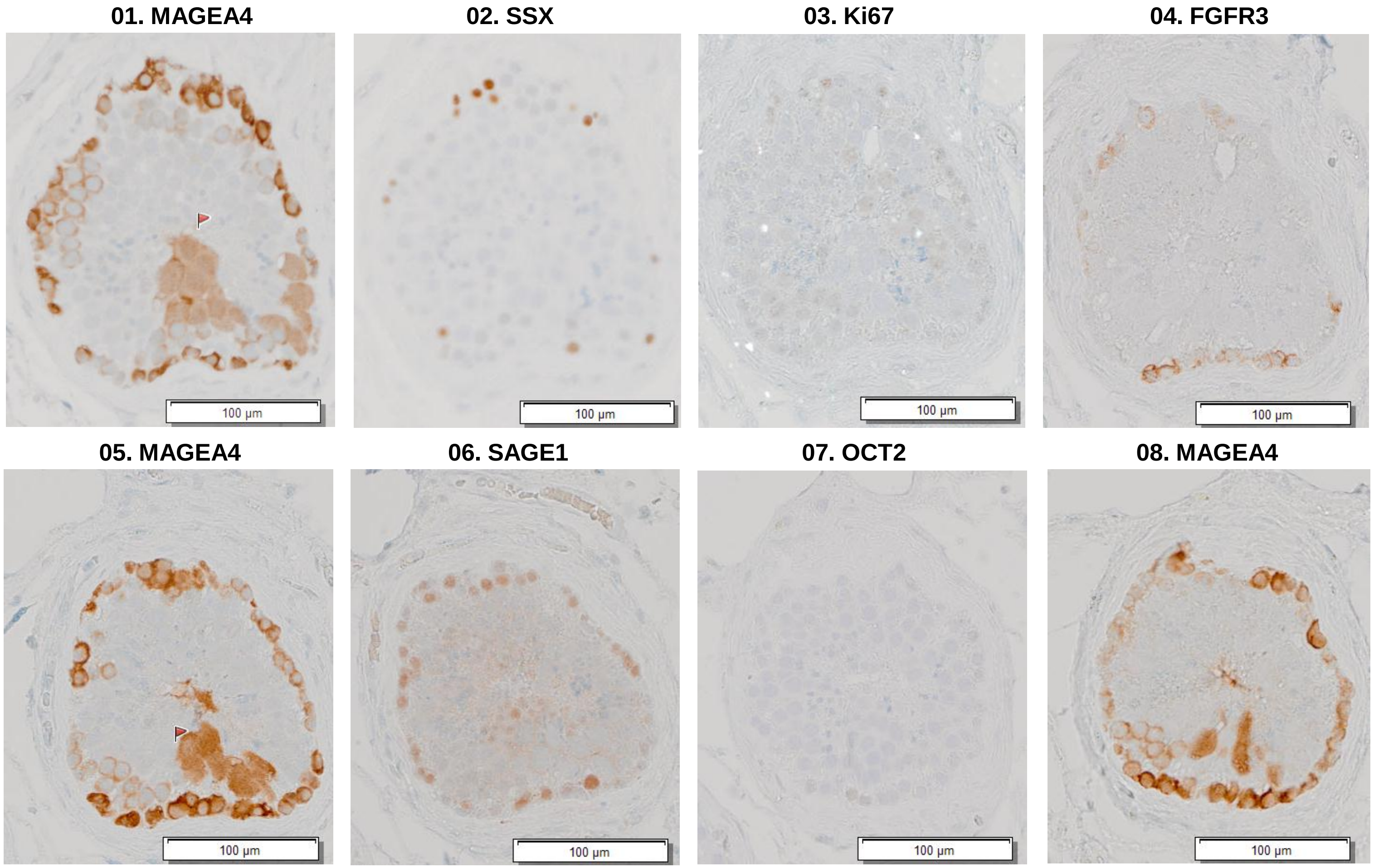

Clone no. 1-1_C4

A

Clone no.	Location	Slide 1	Slide 2	Slide 3	Slide 4	Slide 5	Slide 6	Slide 7	Slide 8	Slide 9	Slide 10	Slide 11	Minimum number of cells
		MAGEA4	SSX	Ki67	FGFR3	MAGEA4	SAGE1	OCT2	MAGEA4	n.s.	missing	MAGEA4	
1-1_C4	Centre	0*	1	0*	1	1	1	0	1			0	78
		6 cells	3 cells	2 cells	5 cells	14 cells	12 cells		7 cells				
		40 µm											

B

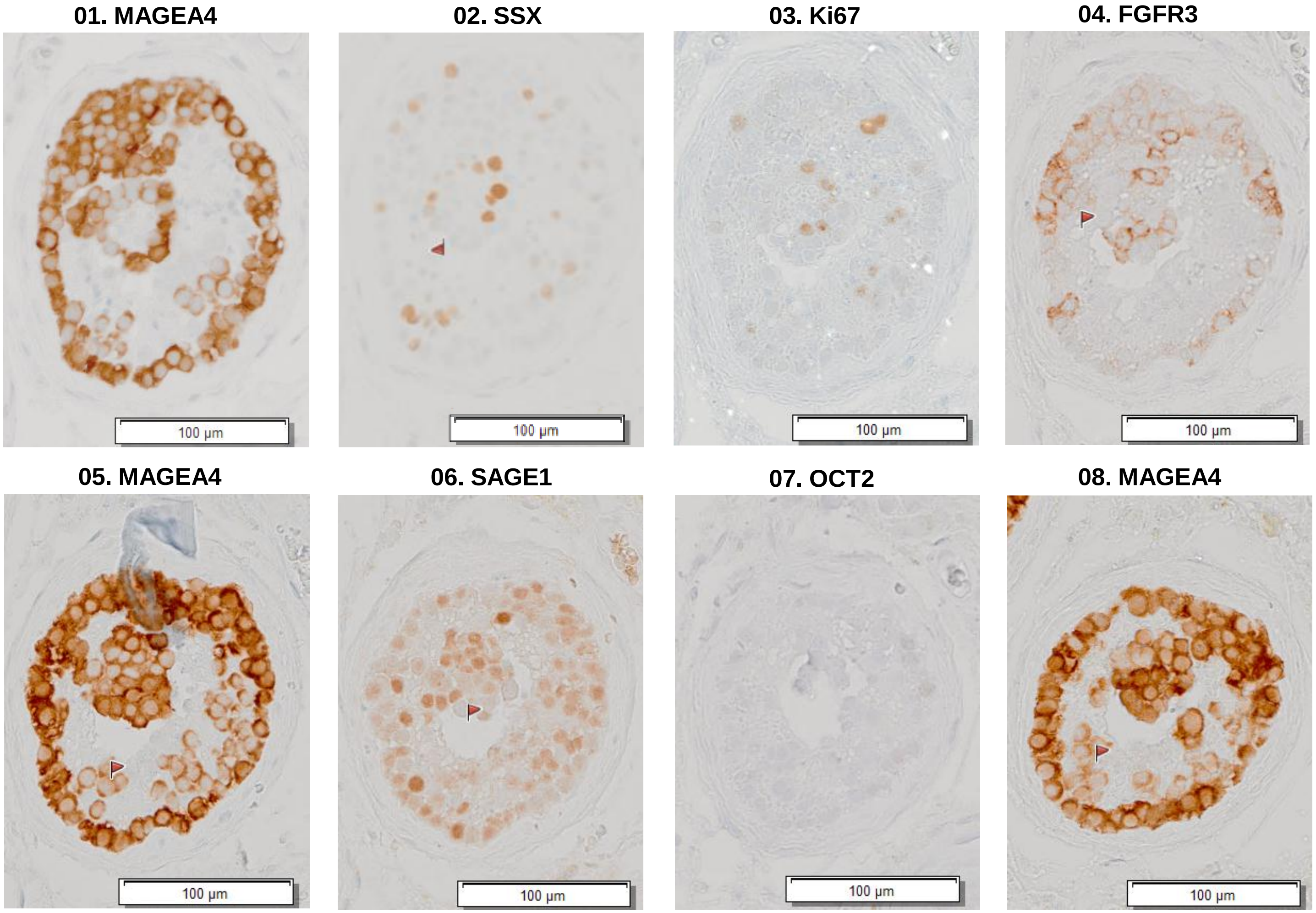

Clone no. 1-1_C5

A

Clone no.	Location	Slide 1	Slide 2	Slide 3	Slide 4	Slide 5	Slide 6	Slide 7	Slide 8	Slide 9	Slide 10	Slide 11	Minimum number of cells
		MAGEA4	SSX	Ki67	FGFR3	MAGEA4	SAGE1	OCT2	MAGEA4	n.s.	missing	MAGEA4	
1-1_C5	Periphery	NA	0	0	0	1	0*	0	1			1	120
						13 cells	4 cells		18 cells			20 cells	
						35 µm							

B

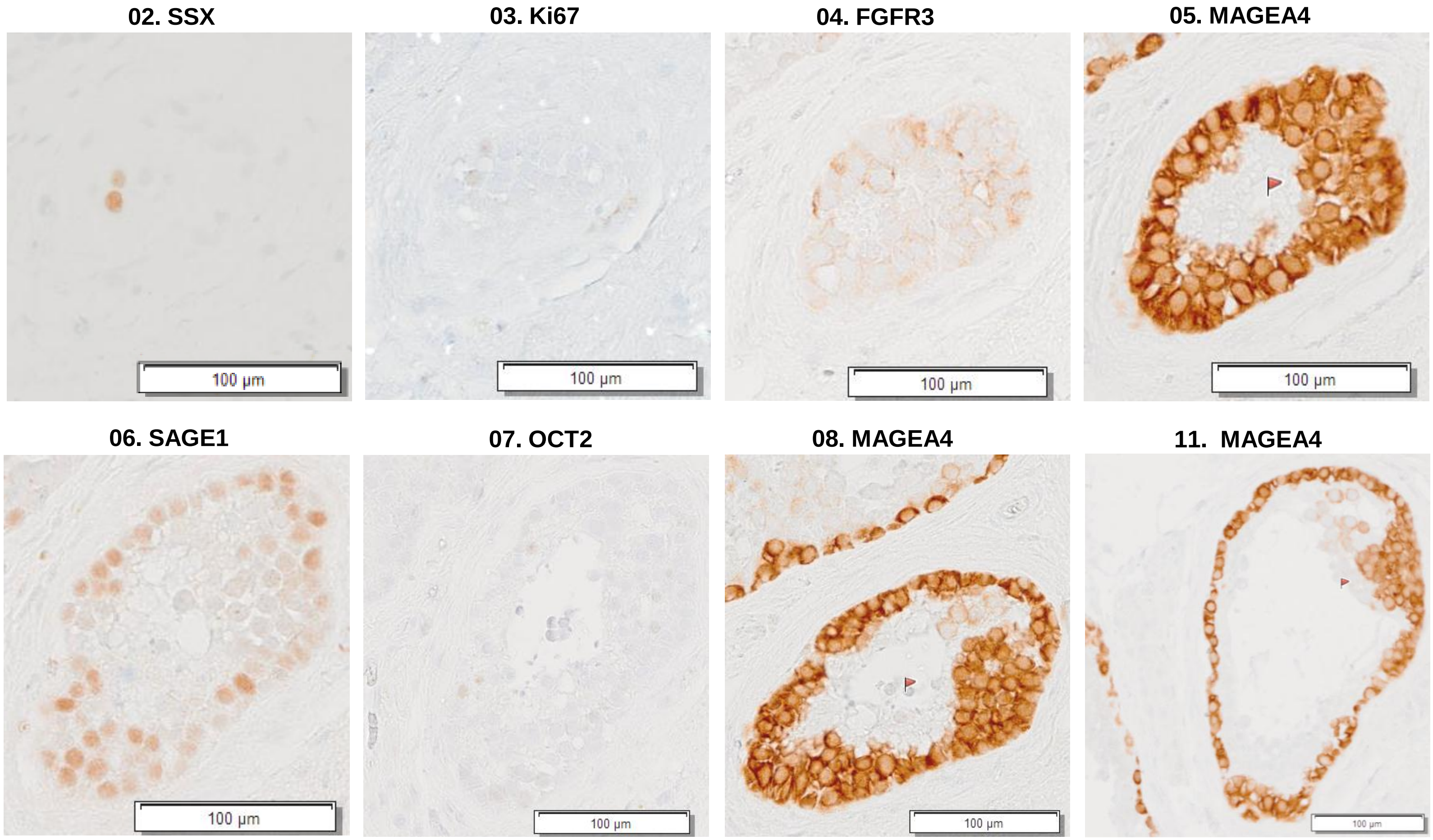

Clone no. 1-1_C6

A

Clone no.	Location	Slide 1	Slide 2	Slide 3	Slide 4	Slide 5	Slide 6	Slide 7	Slide 8	Slide 9	Slide 10	Slide 11	Minimum number of cells
		MAGEA4	SSX	Ki67	FGFR3	MAGEA4	SAGE1	OCT2	MAGEA4	n.s.	missing	MAGEA4	
1-1_C6	Centre	0*	0	0	0	1	1	0	1			0	40
		1 cell				9 cells	3 cells		3 cells				
		40 µm											

B

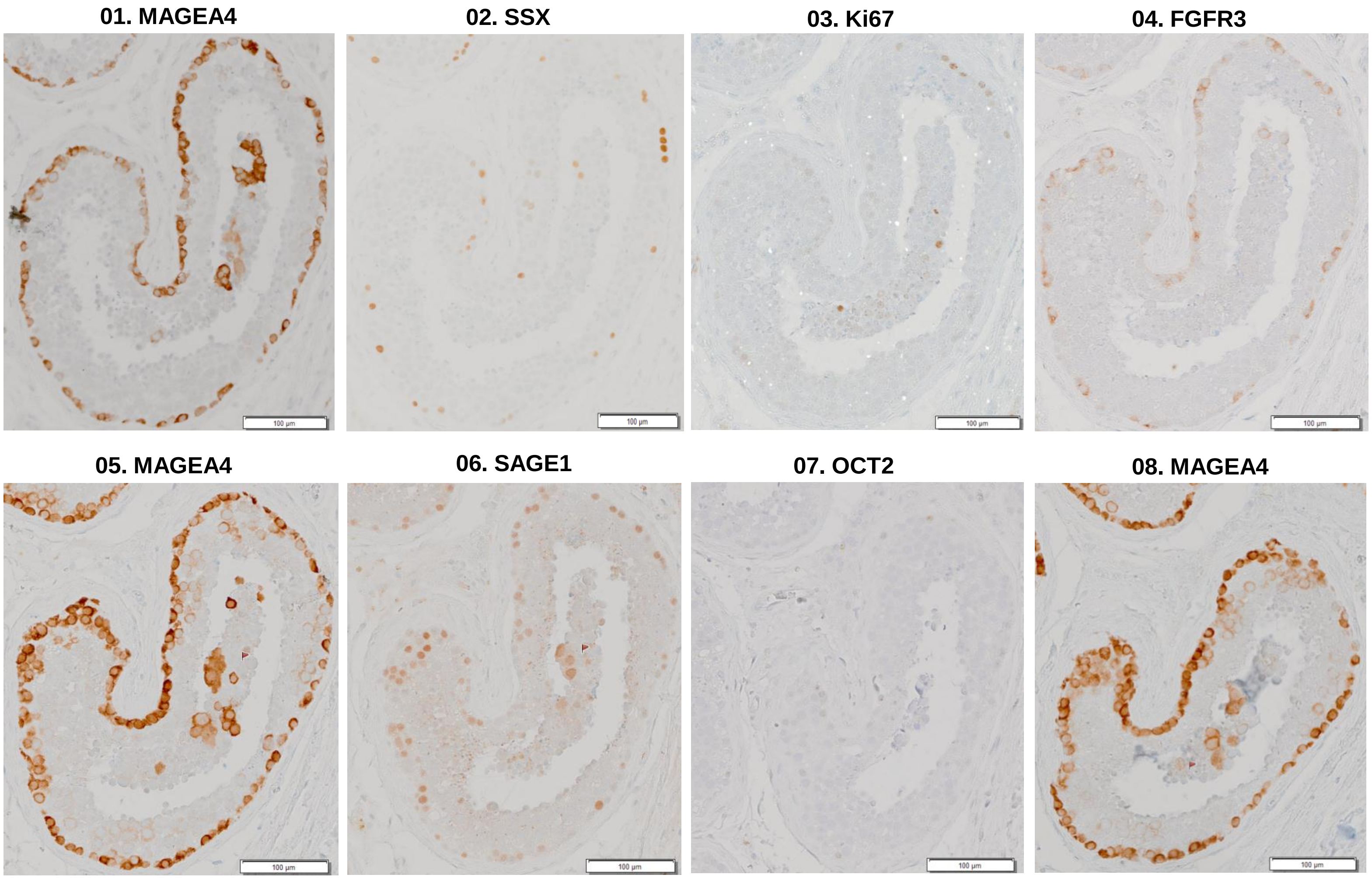

Clone no. 1-1_C7

A

Clone no.	Location	Slide 1	Slide 2	Slide 3	Slide 4	Slide 5	Slide 6	Slide 7	Slide 8	Slide 9	Slide 10	Slide 11	Minimum number of cells
		MAGEA4	SSX	Ki67	FGFR3	MAGEA4	SAGE1	OCT2	MAGEA4	n.s.	missing	MAGEA4	
1-1_C7	Periphery	0	0	0	0	1	0	0	1			0	36
						7 cells			11 cells				
						20µm							

B

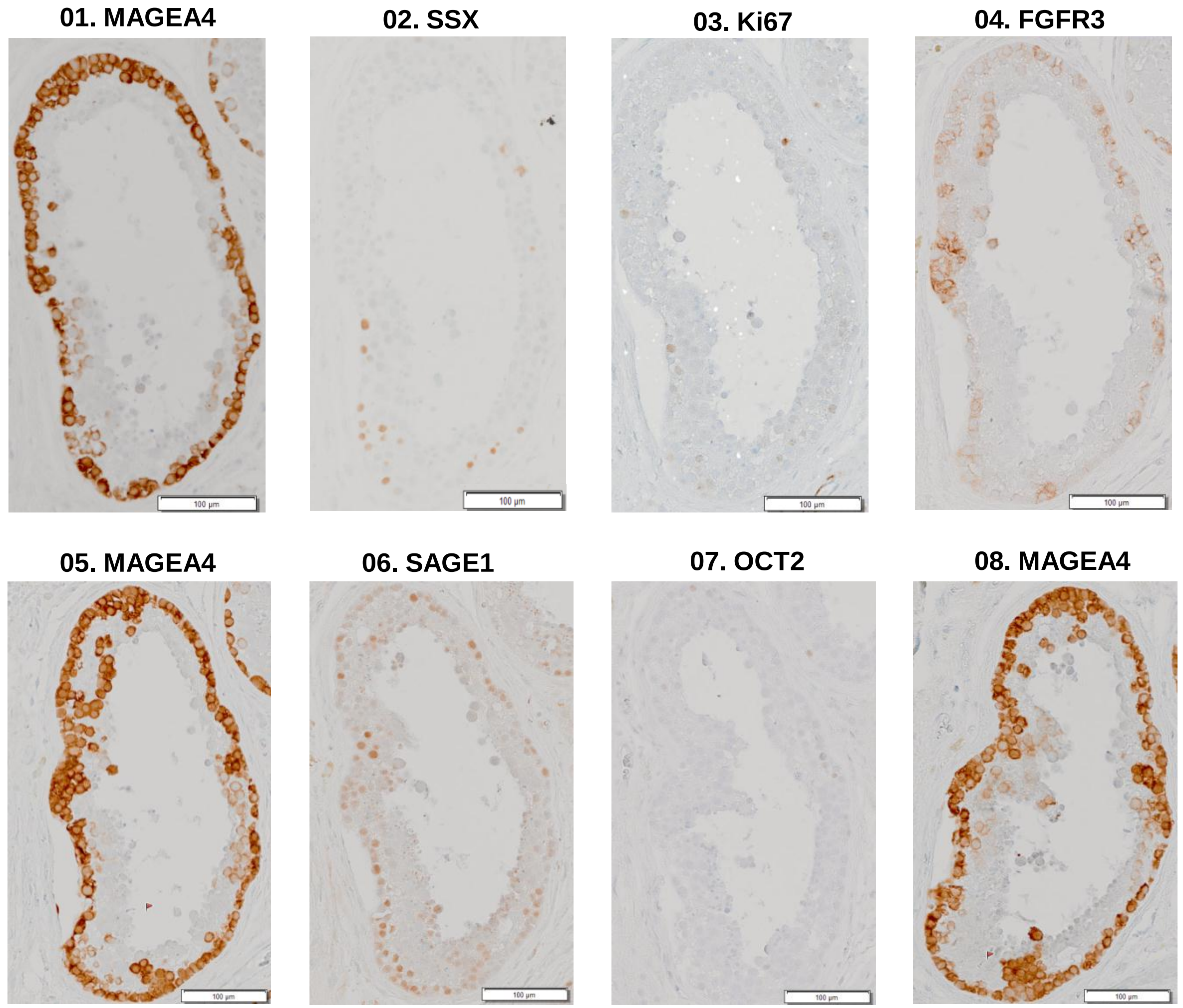

Clone no. 1-1_C8

A

Clone no.	Location	Slide 1	Slide 2	Slide 3	Slide 4	Slide 5	Slide 6	Slide 7	Slide 8	Slide 9	Slide 10	Slide 11	Minimum number of cells
		MAGEA4	SSX	Ki67	FGFR3	MAGEA4	SAGE1	OCT2	MAGEA4	n.s.	missing	MAGEA4	
1-1_C8	Centre	NA	NA	NA	NA	1<br>6 cells	1<br>7 cells	NA	1<br>7 cells			NA	26
						20 µm							

B

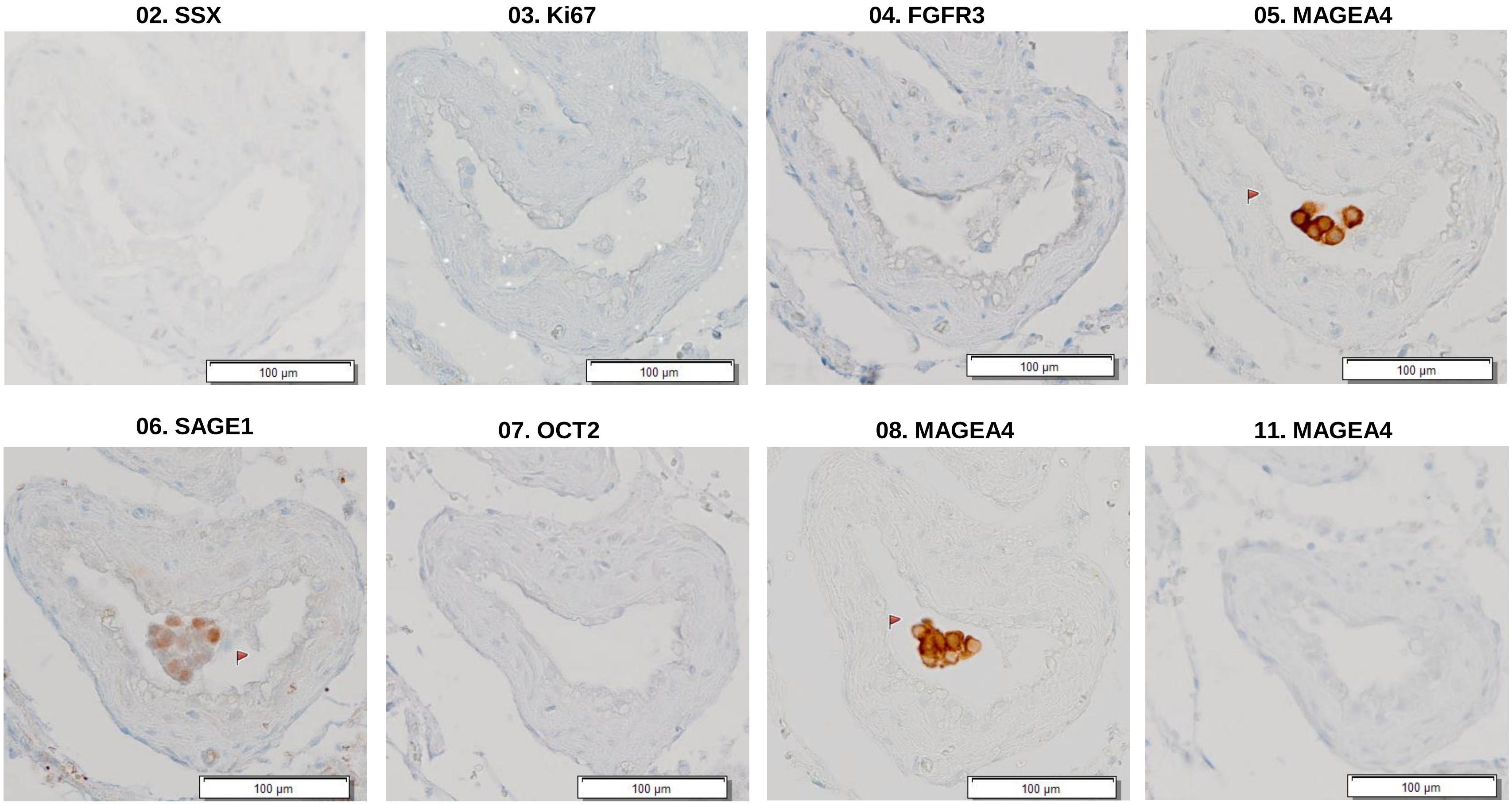

Clone no. 1-1_C9

A

Clone no.	Location	Slide 1	Slide 2	Slide 3	Slide 4	Slide 5	Slide 6	Slide 7	Slide 8	Slide 9	Slide 10	Slide 11	Minimum number of cells
		MAGEA4	SSX	Ki67	FGFR3	MAGEA4	SAGE1	OCT2	MAGEA4	n.s.	missing	MAGEA4	
1-1_C9	Near Periphery	0	0	0	0	1<br>7 cells	0*<br>3 cells	0	1<br>5 cells			0	24
						20 µm							

B

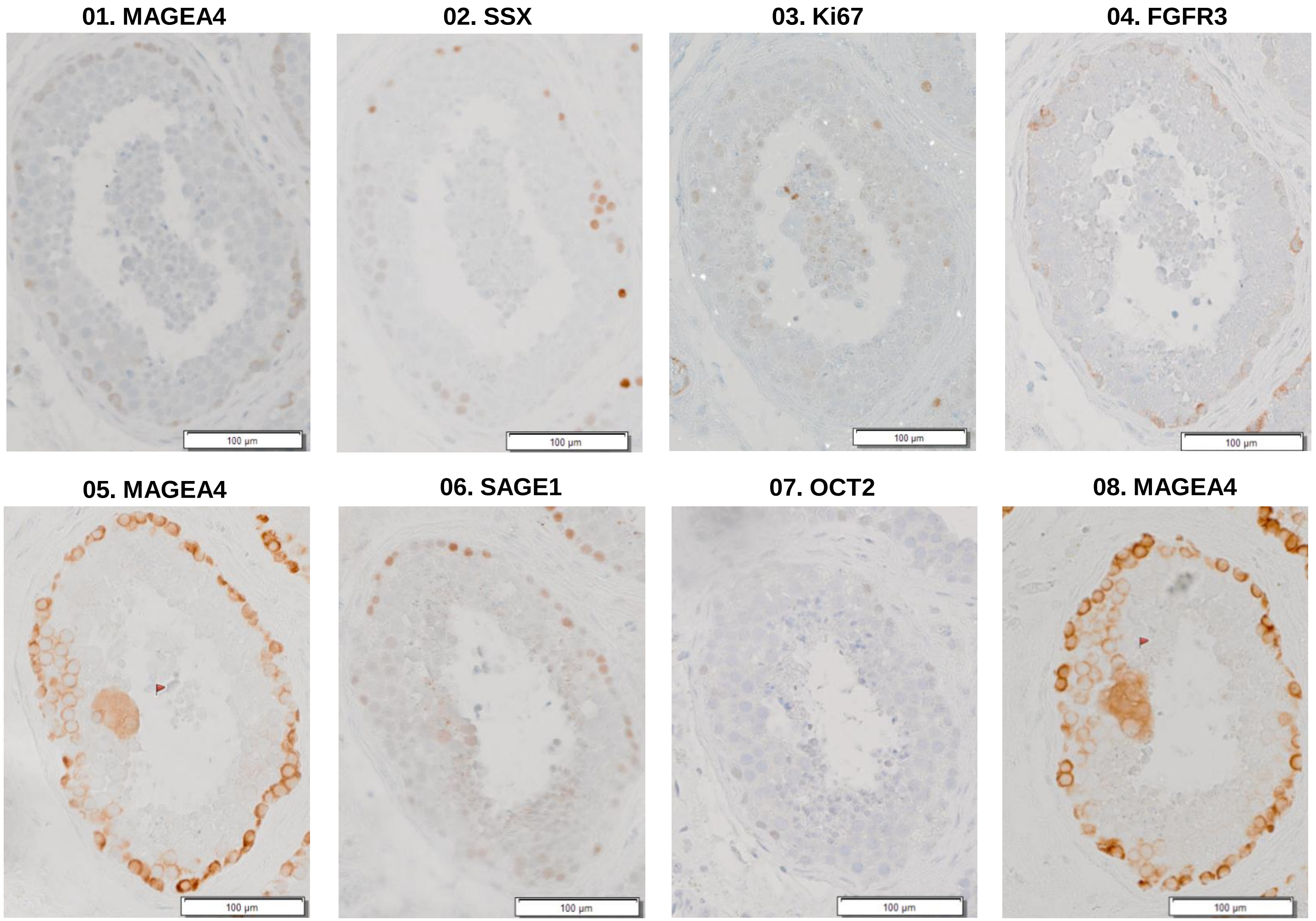

Clone no. 1-1_C10

A

Clone no.	Location	Slide 1	Slide 2	Slide 3	Slide 4	Slide 5	Slide 6	Slide 7	Slide 8	Slide 9	Slide 10	Slide 11	Minimum number of cells
		MAGEA4	SSX	Ki67	FGFR3	MAGEA4	SAGE1	OCT2	MAGEA4	n.s.	missing	MAGEA4	
1-1_C10	Near Periphery	0	0	0	0	1<br>7 cells	0	0	1<br>5 cells			0	24
						20 µm							

B

01. MAGEA4

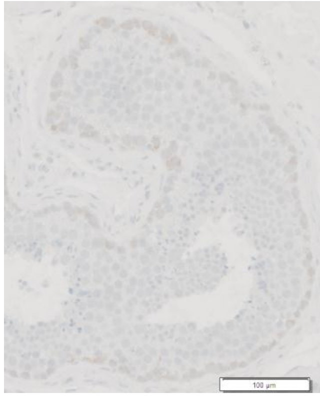

02. SSX

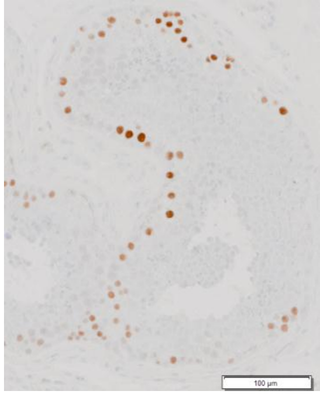

03. Ki67

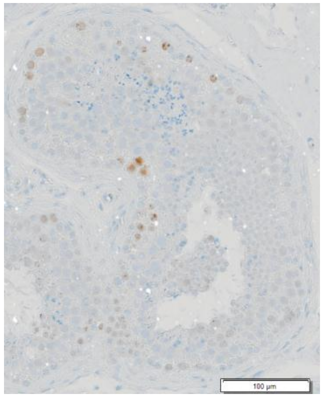

04. FGFR3

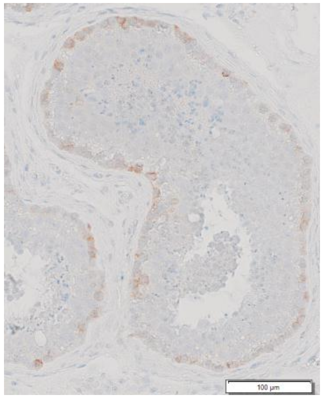

05. MAGEA4

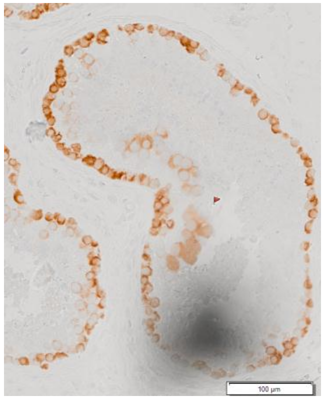

06. SAGE1

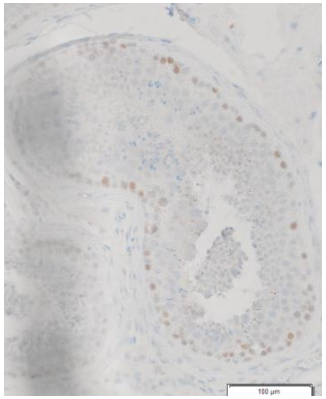

07. OCT2

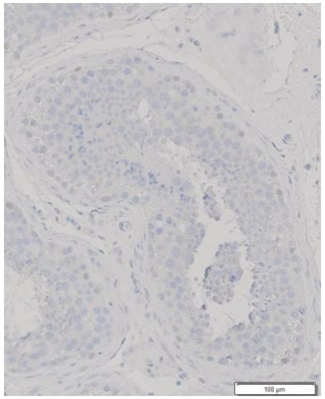

08. MAGEA4

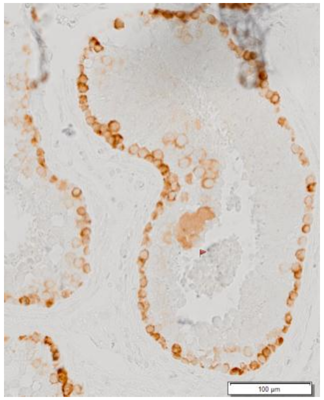

Clone no. 1-1_C11

A

Clone no.	Location	Slide 1	Slide 2	Slide 3	Slide 4	Slide 5	Slide 6	Slide 7	Slide 8	Slide 9	Slide 10	Slide 11	Minimum number of cells
		MAGEA4	SSX	Ki67	FGFR3	MAGEA4	SAGE1	OCT2	MAGEA4	n.s	missing	MAGEA4	
1-1_C11	Periphery	0	0	0	0*	1	0	0	1			0*	57
					5 cells	9 cells			6 cells			8 cells	
					40 µm								

B

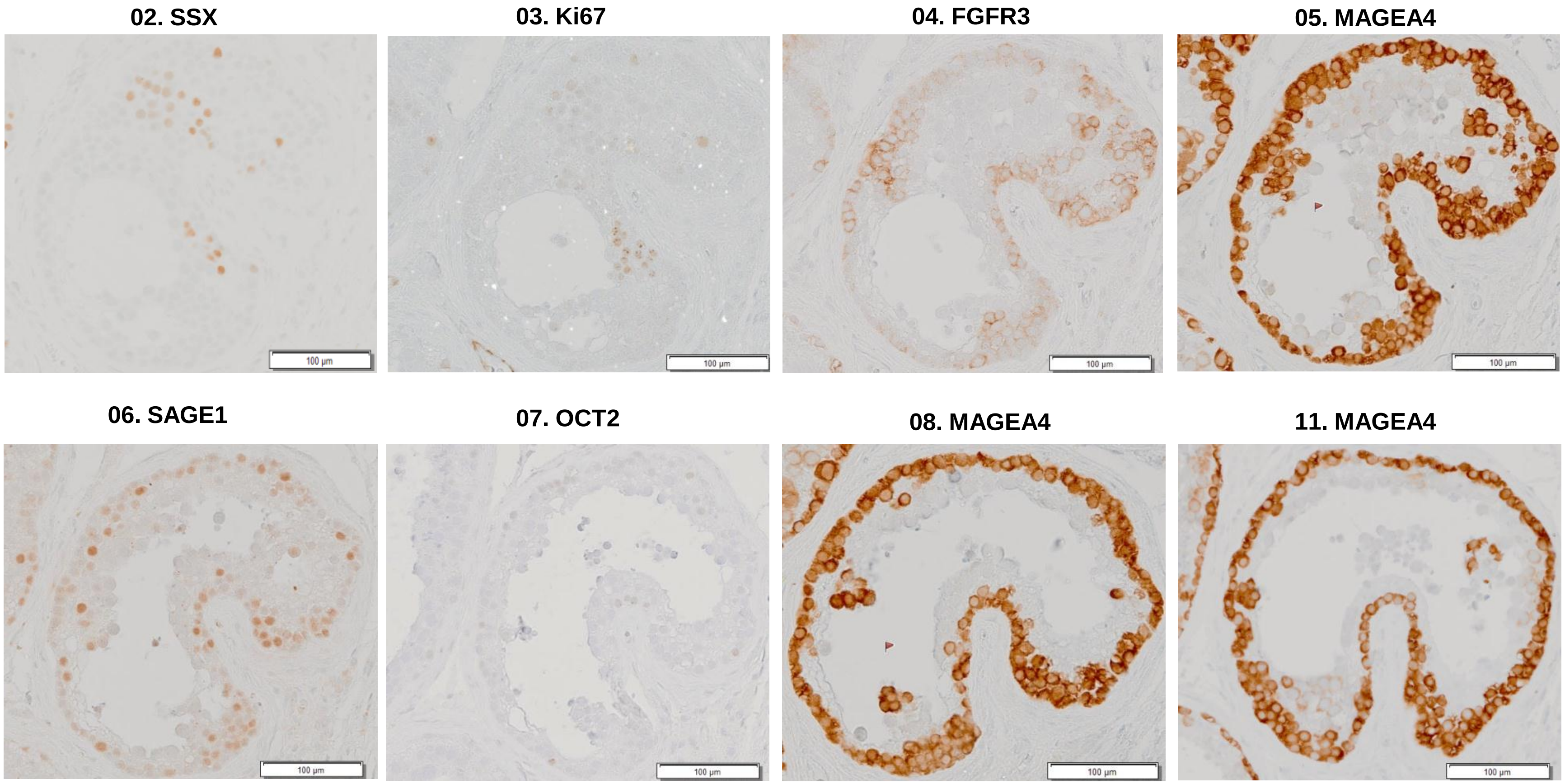

Clone no. 1-1_C12

A

Clone no.	Location	Slide 1	Slide 2	Slide 3	Slide 4	Slide 5	Slide 6	Slide 7	Slide 8	Slide 9	Slide 10	Slide 11	Minimum number of cells
		MAGEA4	SSX	Ki67	FGFR3	MAGEA4	SAGE1	OCT2	MAGEA4	n.s	missing	MAGEA4	
1-1_C12	Centre	0	0	0	0	1<br>6 cells	0	0	1<br>10 cells			0	32
						20 µm							

B

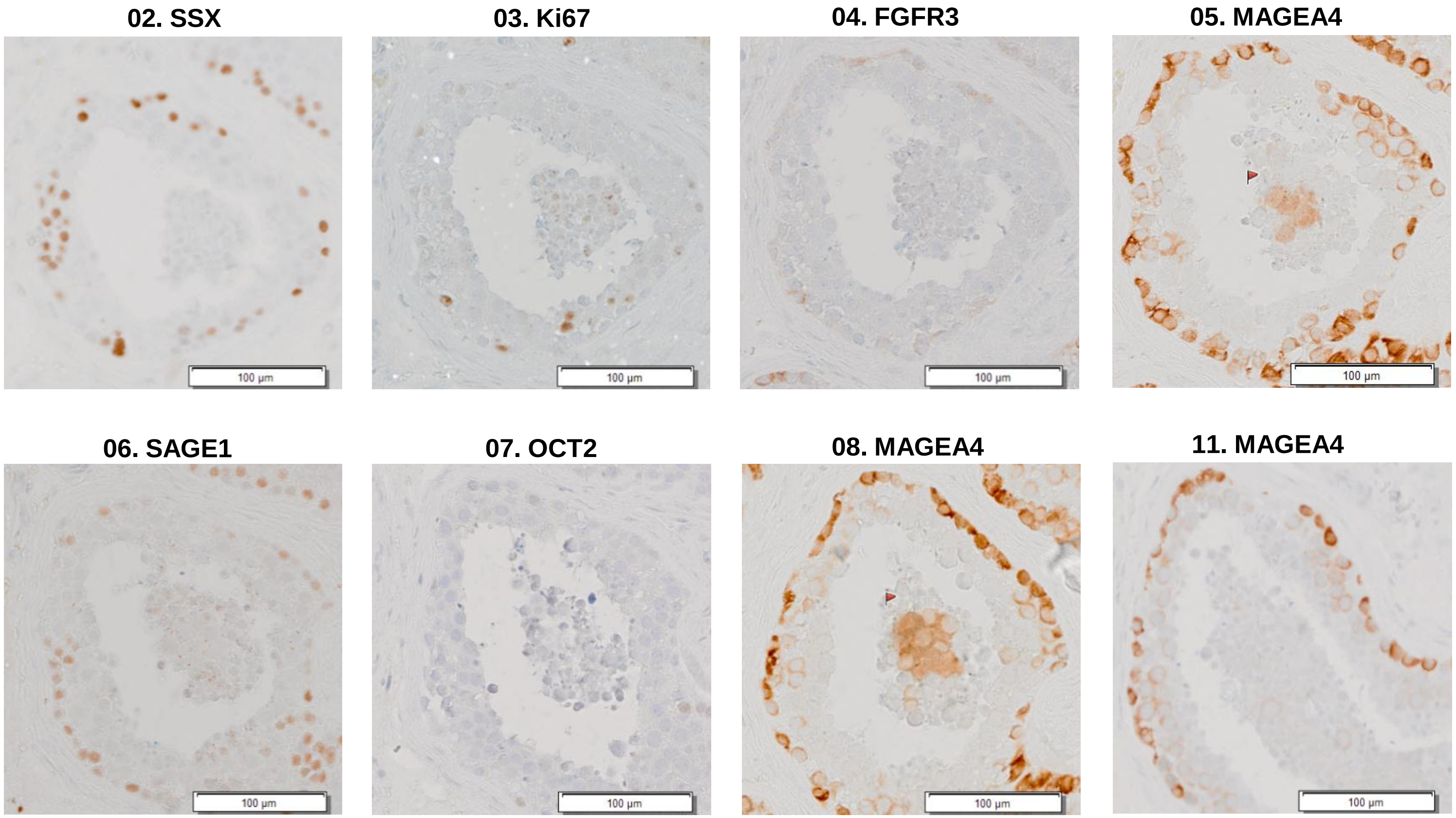

Clone no. 1-1_C13

A

Clone no.	Location	Slide 1	Slide 2	Slide 3	Slide 4	Slide 5	Slide 6	Slide 7	Slide 8	Slide 9	Slide 10	Slide 11	Minimum number of cells
		MAGEA4	SSX	Ki67	FGFR3	MAGEA4	SAGE1	OCT2	MAGEA4	n.s	missing	MAGEA4	
1-1_C13	Near Periphery	0	0	0	0	1<br>7 cells	0*<br>4 cells	0	1<br>7 cells			0	28
						20 µm							

B

01. MAGEA4

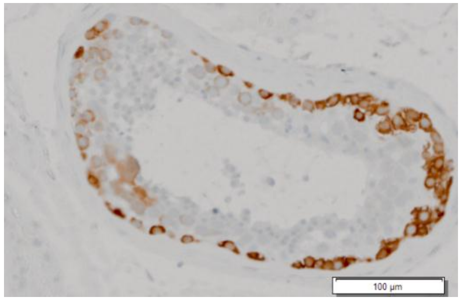

02. SSX

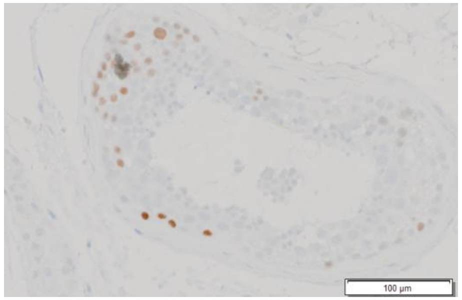

03. Ki67

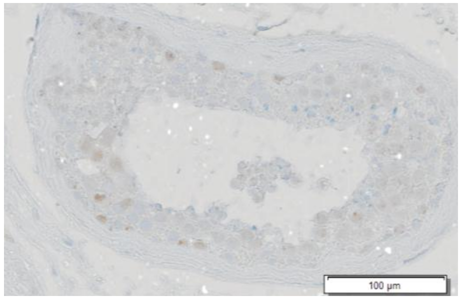

04. FGFR3

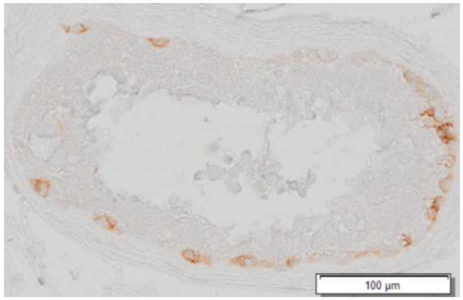

05. MAGEA4

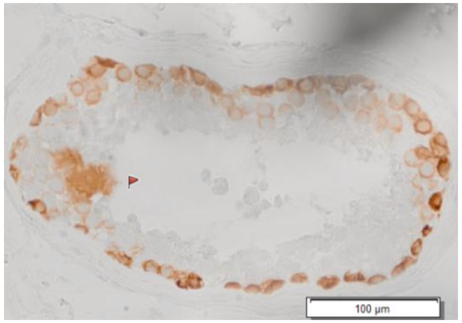

06. SAGE1

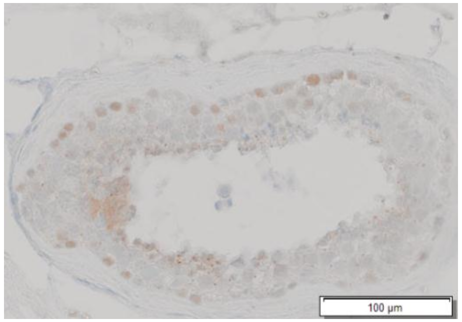

07. OCT2

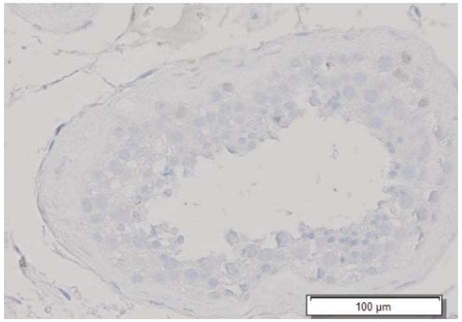

08. MAGEA4

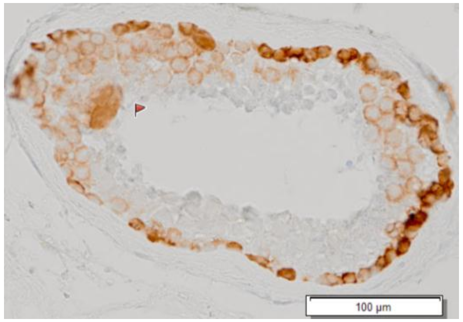

Clone no. 1-1_C14

A

Clone no.	Location	Slide 21	Slide 22	Slide 23	Slide 24	Slide 25	Slide 26	Slide 27	Slide 28	Slide 29	Minimum number of cells
		MAGEA4	SSX	MAGEA4	Ki67	H&E	MAGEA4	OCT2	MAGEA4	FGFR3	
1-1_C14	Centre	NA	0	1	0		1	0	1	NA	72
				13 cells			16 cells		4 cells		
				30 µm							

B

22. SSX

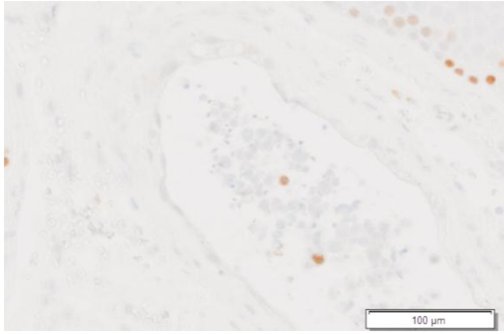

23. MAGEA4

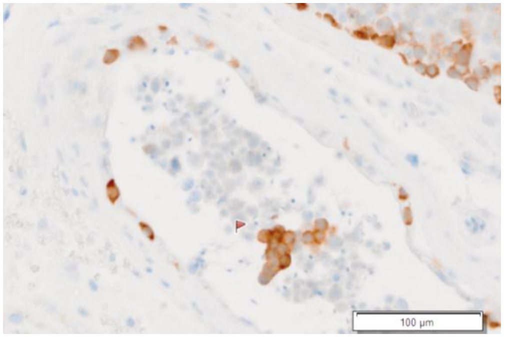

24. Ki67

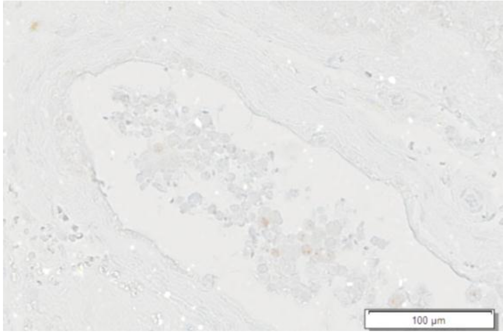

26. MAGEA4

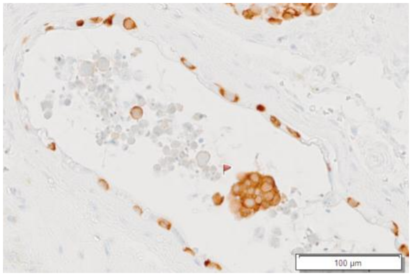

27. OCT2

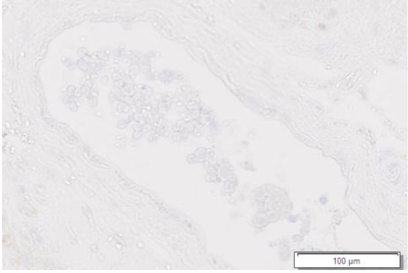

28. MAGEA4

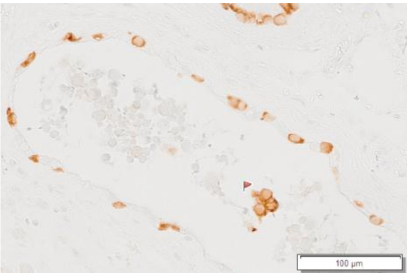

29. FGFR3

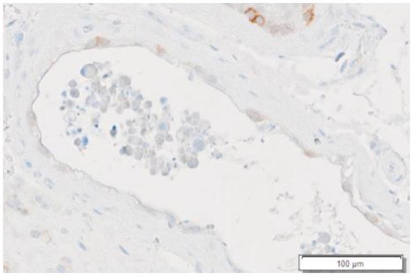

Clone no. 1-1_C15

A

Clone no.	Location	Slide 23	Slide 24	Slide 25	Slide 26	Slide 27	Slide 28	Slide 29	Slide 30	Slide 31	Slide 32	Slide 33	Slide 34	Slide 35	Minimum number of cells
		MAGEA4	Ki67	H&E	MAGEA4	OCT2	MAGEA4	FGFR3	Ki67	MAGEA4	SSX	SAGE1	OCT2	MAGEA4	
1-1_C15	Centre	0*	0		1	0	1	0	0	0*	0	0	0	0*	58
		2 cells			7 cells		8 cells			3 cells				2 cells	
		65 µm													

B

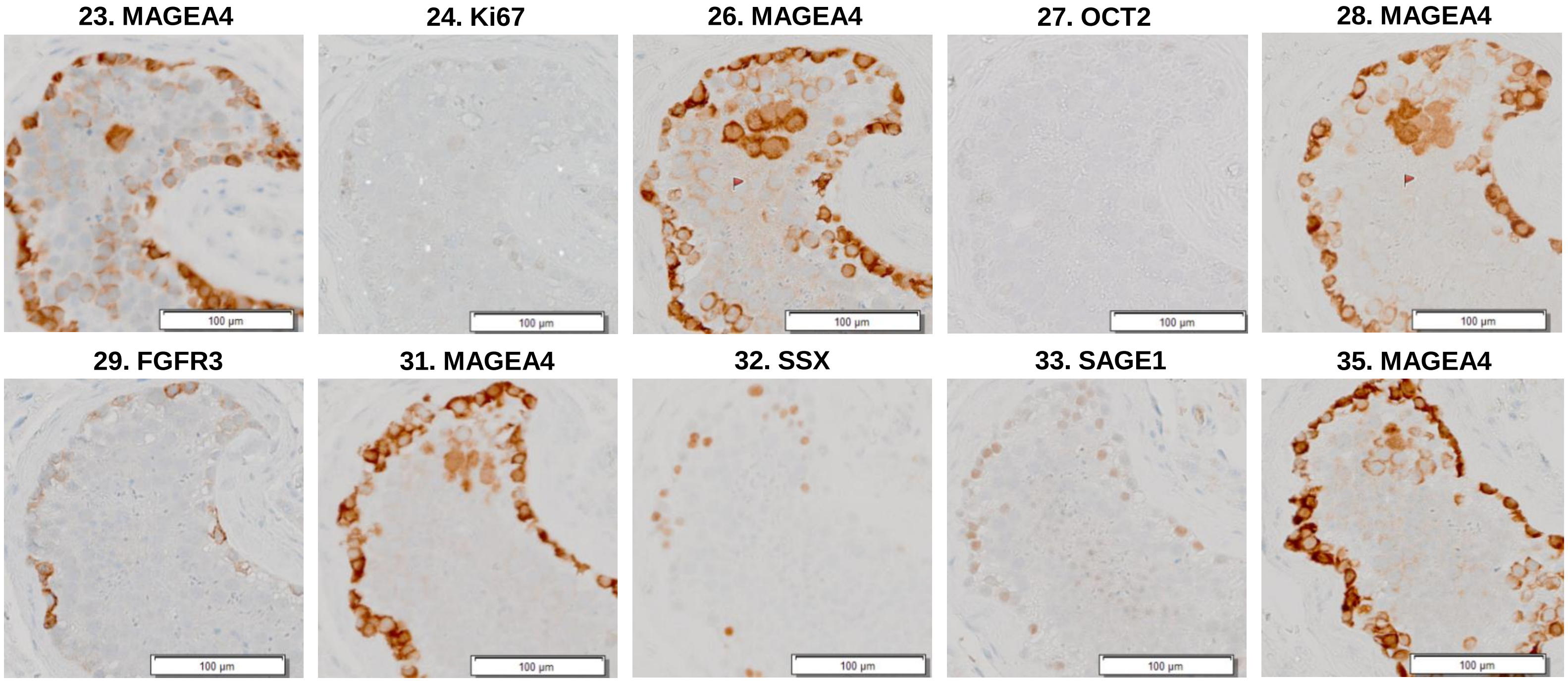

Clone no. 1-1_C16

A

Clone no.	Location	Slide 21	Slide 22	Slide 23	Slide 24	Slide 25	Slide 26	Slide 27	Slide 28	Slide 29	Slide 30	Slide 31	Slide 32	Slide 33	Minimum number of cells
		MAGEA4	SSX	MAGEA4	Ki67	H&E	MAGEA4	OCT2	MAGEA4	FGFR3	Ki67	MAGEA4	SSX	SAGE1	
1-1_C16	Periphery	NA	0	1<br>57 cells	0		1<br>13 cells	0	1<br>15 cells	0	0	0*<br>2 cells	0	0	188
				45 µm	45 µm	45 µm	45 µm	45 µm	45 µm	45 µm	45 µm	45 µm			

B

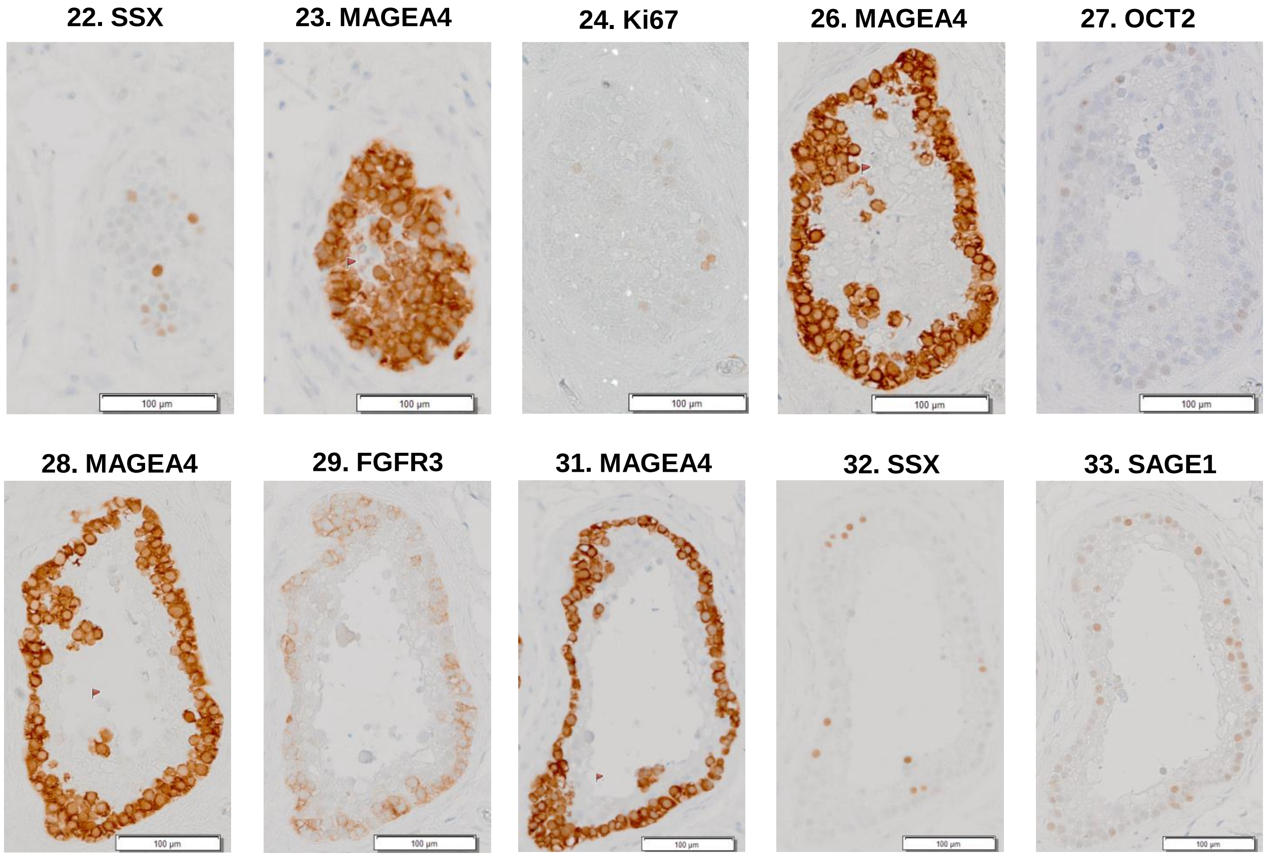

Clone no. 1-1_C17

A

Clone no.	Location	Slide 23	Slide 24	Slide 25	Slide 26	Slide 27	Slide 28	Slide 29	Slide 30	Slide 31	Slide 32	Slide 33	Minimum number of cells
		MAGEA4	Ki67	H&E	MAGEA4	OCT2	MAGEA4	FGFR3	Ki67	MAGEA4	SSX	SAGE1	
1-1_C17	Periphery	0	0		1<br>5 cells	0	1<br>4 cells	0	0	0*<br>2 cells	0	0	22
					30 µm								

B

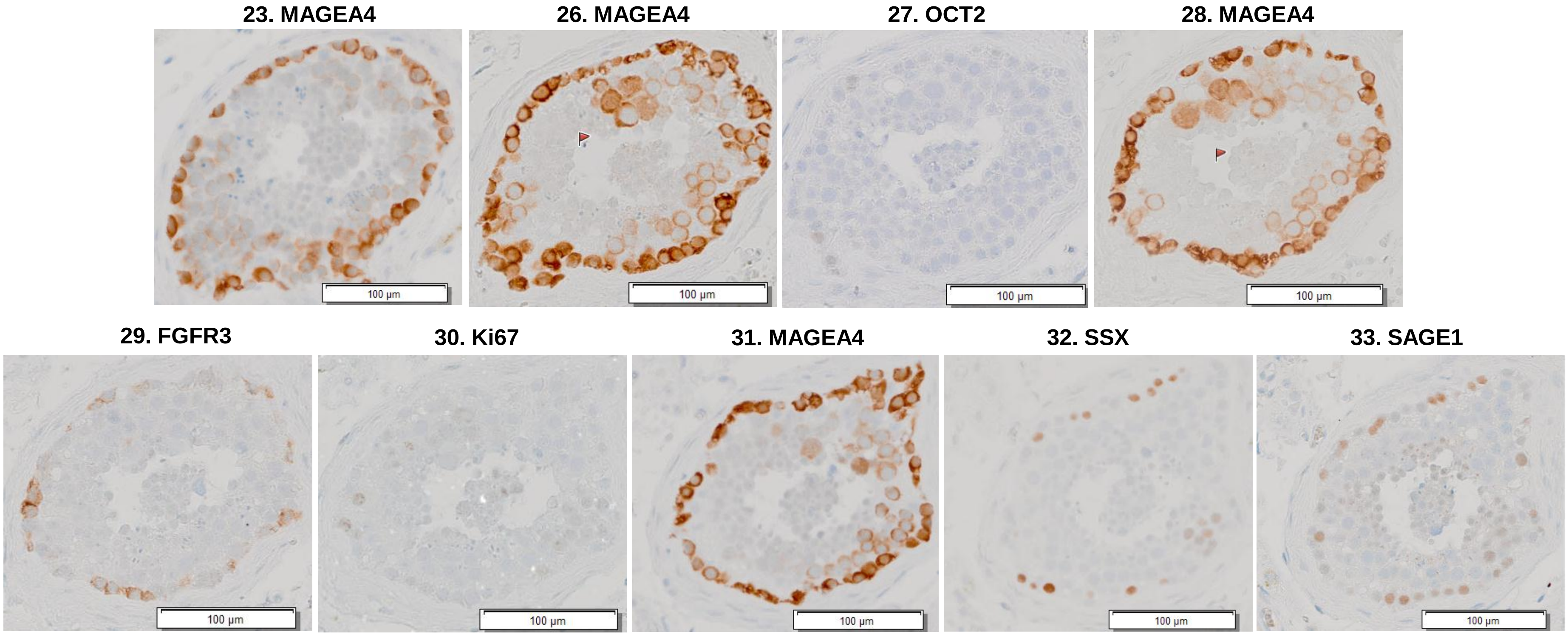

Clone no. 1-1_C18

A

Clone no.	Location	Slide 21	Slide 22	Slide 23	Slide 24	Slide 25	Slide 26	Slide 27	Slide 28	Slide 29	Slide 30	Slide 31	Slide 32	Slide 33	Slide 34	Slide 35	Minimum number of cells
		MAGEA4	SSX	MAGEA4	Ki67	H&E	MAGEA4	OCT2	MAGEA4	FGFR3	Ki67	MAGEA4	SSX	SAGE1	OCT2	MAGEA4	
1-1_C18	Centre	0		0* 4 cells			1 17 cells	0	1 8 cells	0* 7 cells	0	1 9 cells	0	0		0	89
				45 µm													

B

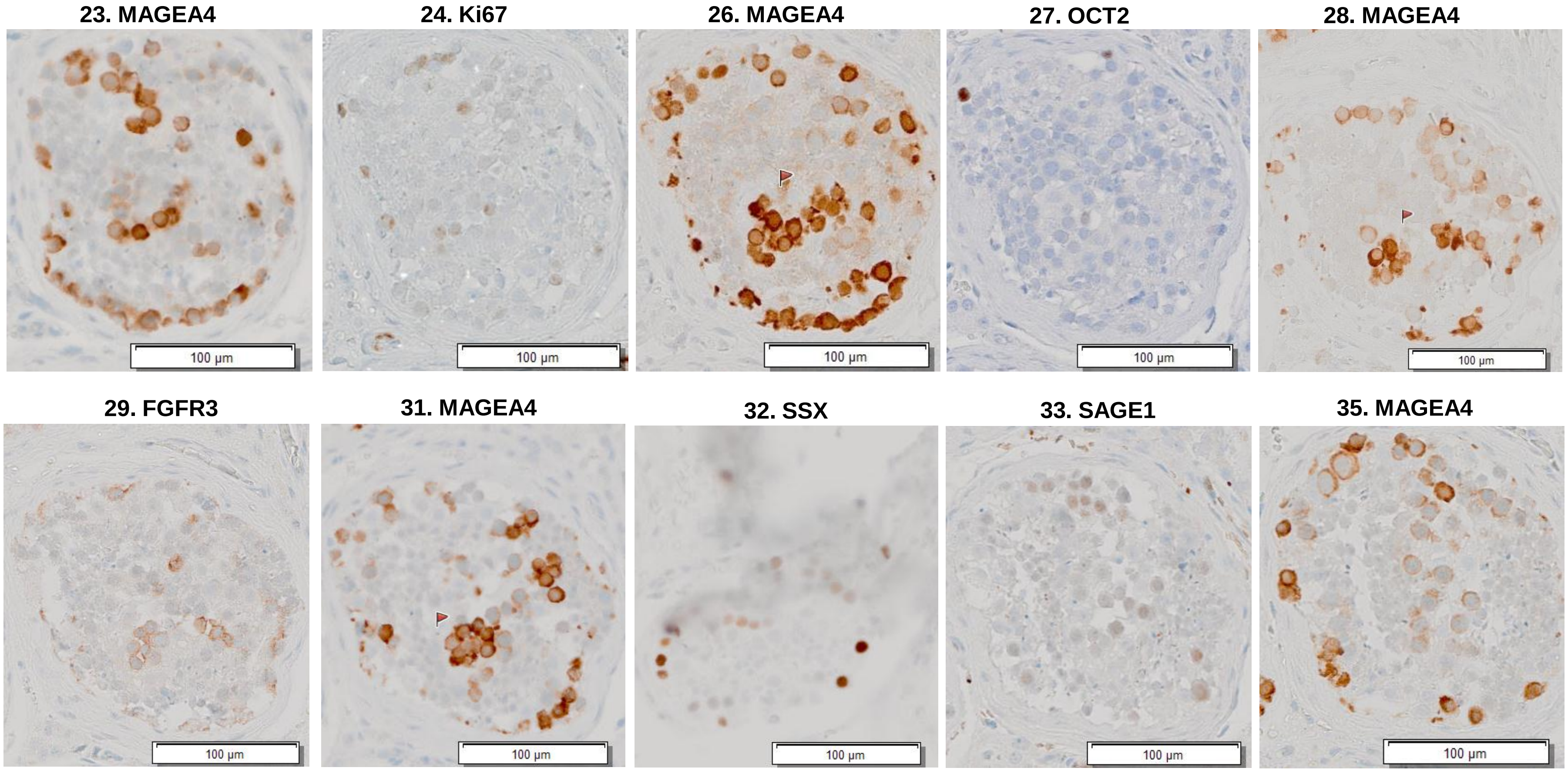

Clone no. 1-1_C19

A

Clone no.	Location	Slide 21	Slide 22	Slide 23	Slide 24	Slide 25	Slide 26	Slide 27	Slide 28	Slide 29	Slide 30	Slide 31	Minimum number of cells
		MAGEA4	SSX	MAGEA4	Ki67	H&E	MAGEA4	OCT2	MAGEA4	FGFR3	Ki67	MAGEA4	
1-1_C19	Centre	0	NA	1<br>4 cells	0		1<br>17 cells	0	1<br>4 cells	0*<br>2 cells	0	0	59
				35 µm (Slides 23–29)									

B

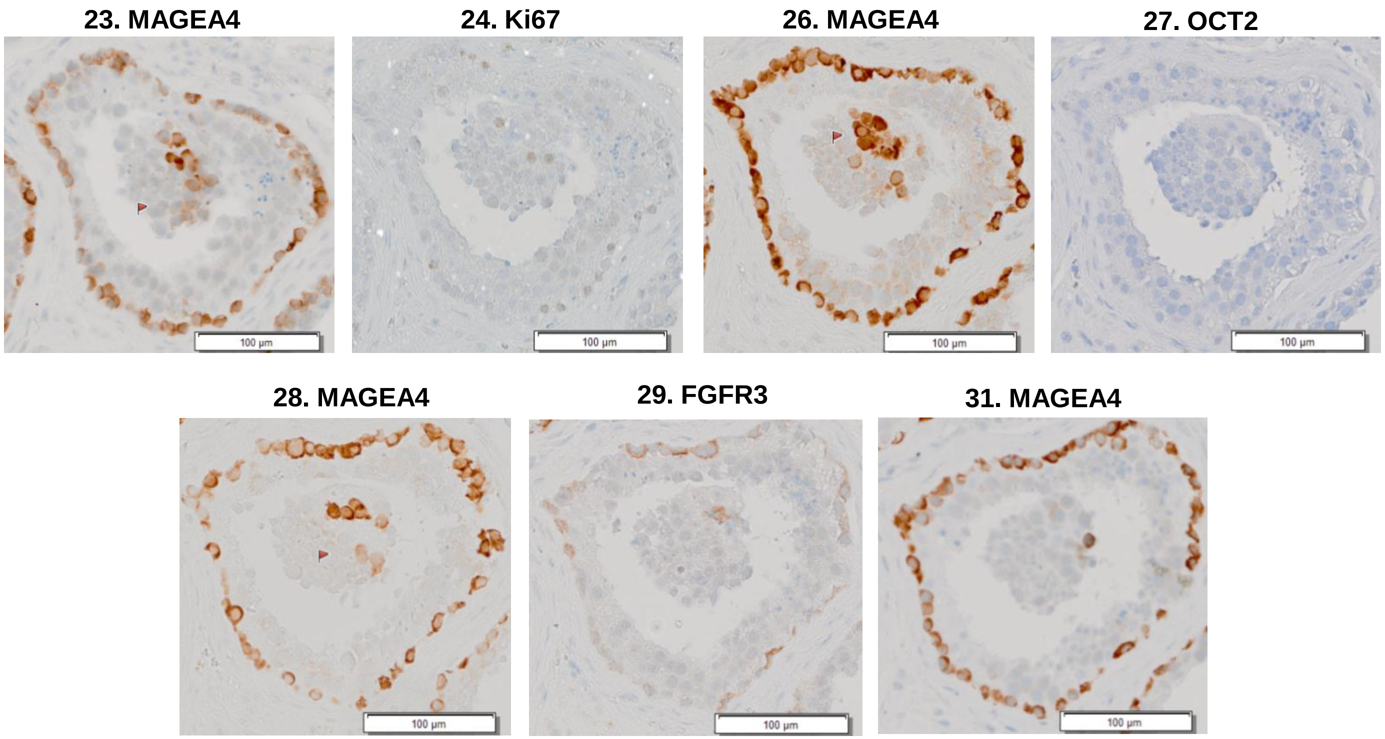

Clone no. 1-1_C20

A

Clone no.	Location	Slide 23	Slide 24	Slide 25	Slide 26	Slide 27	Slide 28	Slide 29	Slide 30	Slide 31	Minimum number of cells
		MAGEA4	Ki67	H&E	MAGEA4	OCT2	MAGEA4	FGFR3	Ki67	MAGEA4	
1-1_C20	Centre	0	0		1<br>4 cells	0	1<br>6 cells	0	0	0	15
					15 µm						

B

23. MAGEA4 **24. Ki67** **26. MAGEA4** **27. OCT2**

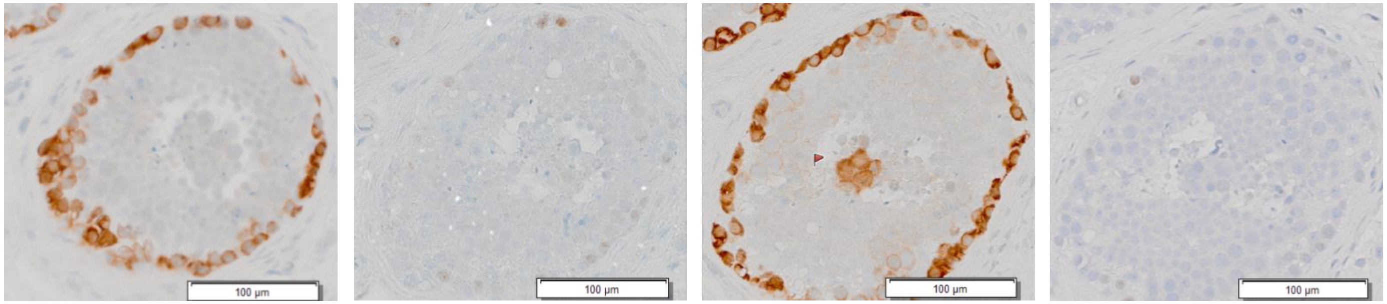

28. MAGEA4 **29. FGFR3** **31. MAGEA4**

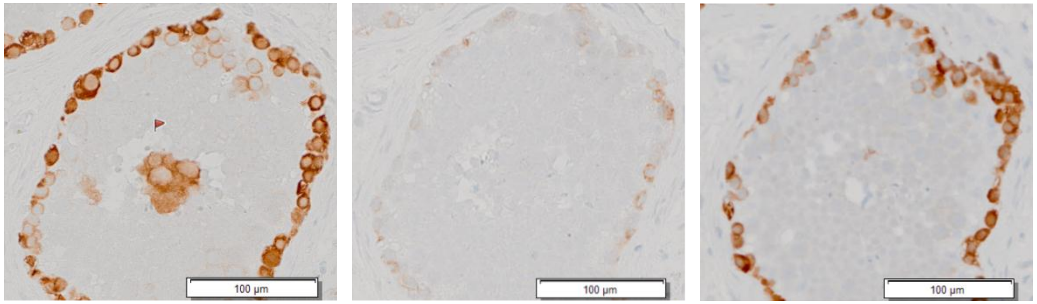

Clone no. 1-1_C21

A

Clone no.	Location	Slide 21 MAGEA4	Slide 22 SSX	Slide 23 MAGEA4	Slide 24 Ki67	Slide 25 H&E	Slide 26 MAGEA4	Slide 27 OCT2	Slide 28 MAGEA4	Slide 29 FGFR3	Slide 30 Ki67	Slide 31 MAGEA4	Minimum number of cells
1-1_C21	Centre	NA		1<br>9 cells	0		1<br>9 cells	0	0*<br>2 cells	0	0	0	44
				30 µm									

B

23. MAGEA4

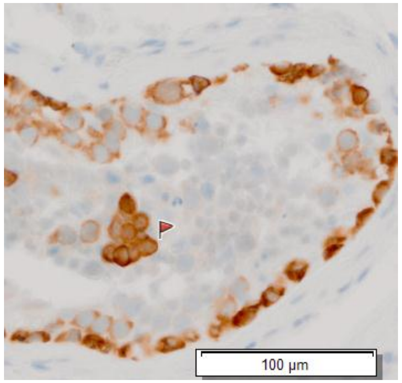

24. Ki67

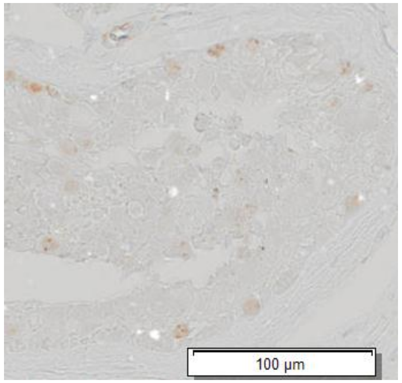

26. MAGEA4

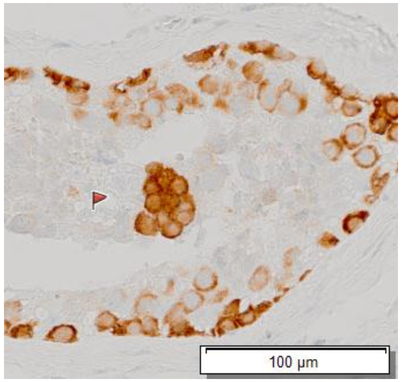

27. OCT2

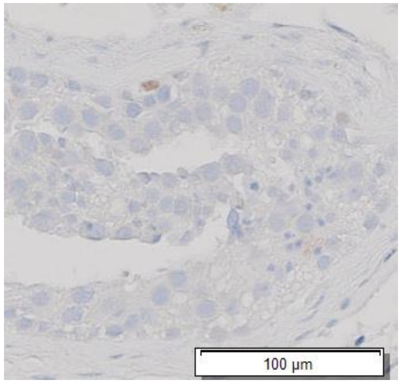

28. MAGEA4

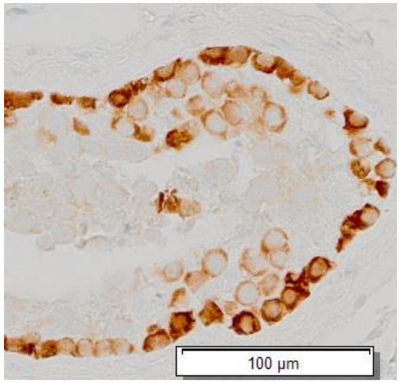

29. FGFR3

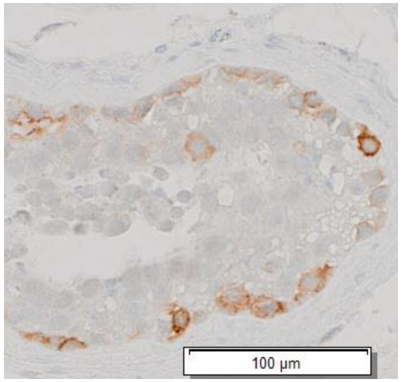

31. MAGEA4

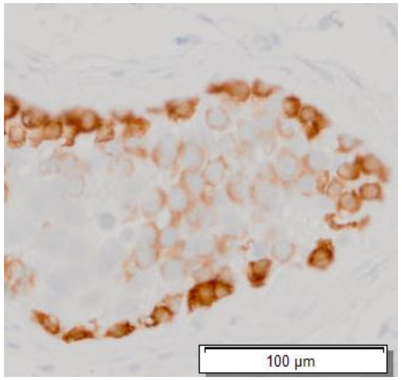

Clone no. 1-1_C22

A

Clone no.	Location	Slide 23	Slide 24	Slide 25	Slide 26	Slide 27	Slide 28	Slide 29	Slide 30	Slide 31	Minimum number of cells
		MAGEA4	Ki67	H&E	MAGEA4	OCT2	MAGEA4	FGFR3	Ki67	MAGEA4	
1-1_C22	Centre	NA	0		1<br>5 cells	0	1<br>5 cells	0	0	0	15
					15 μm						

B

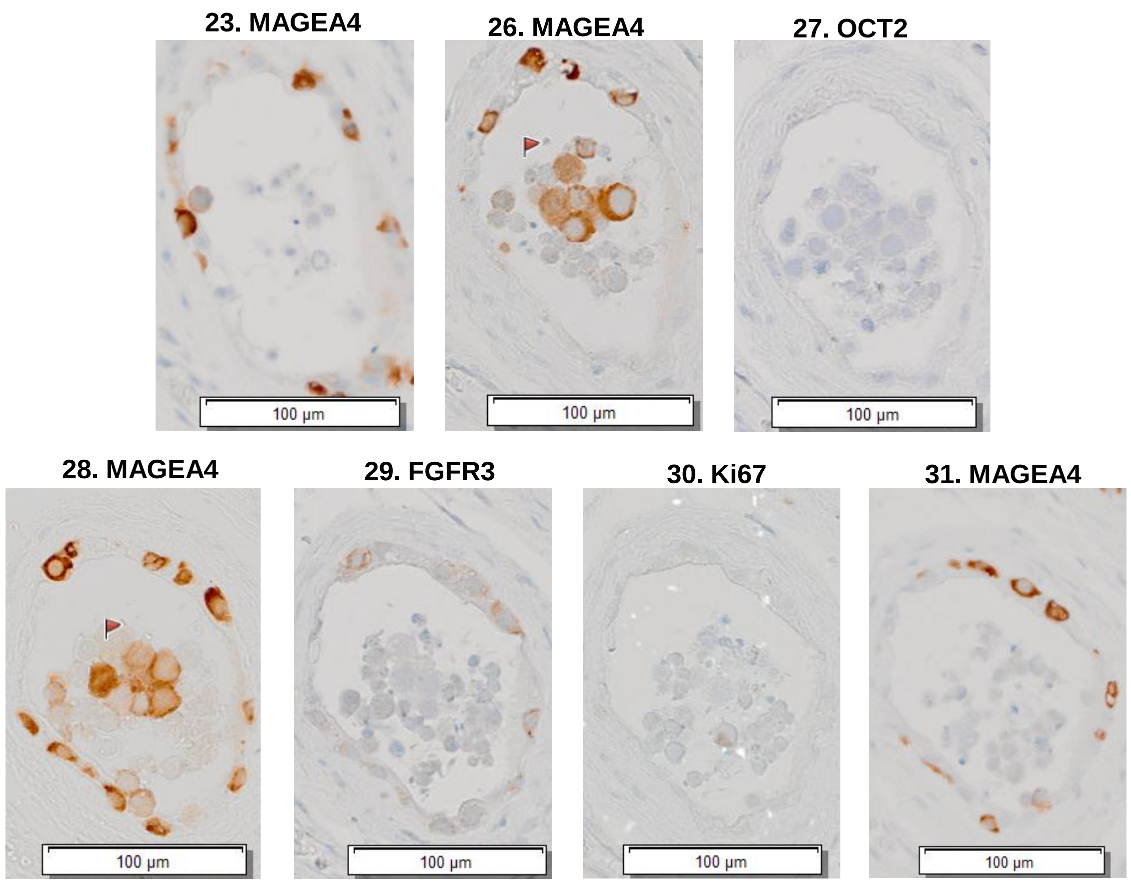

Clone no. 1-1_C23

A

Clone no.	Location	Slide 23 MAGEA4	Slide 24 Ki67	Slide 25 H&E	Slide 26 MAGEA4	Slide 27 OCT2	Slide 28 MAGEA4	Slide 29 FGFR3	Slide 30 Ki67	Slide 31 MAGEA4	Minimum number of cells
1-1_C23	Periphery	0	0		1<br>10 cells	0	1<br>8 cells	0	0	0	27
					15 µm						

B

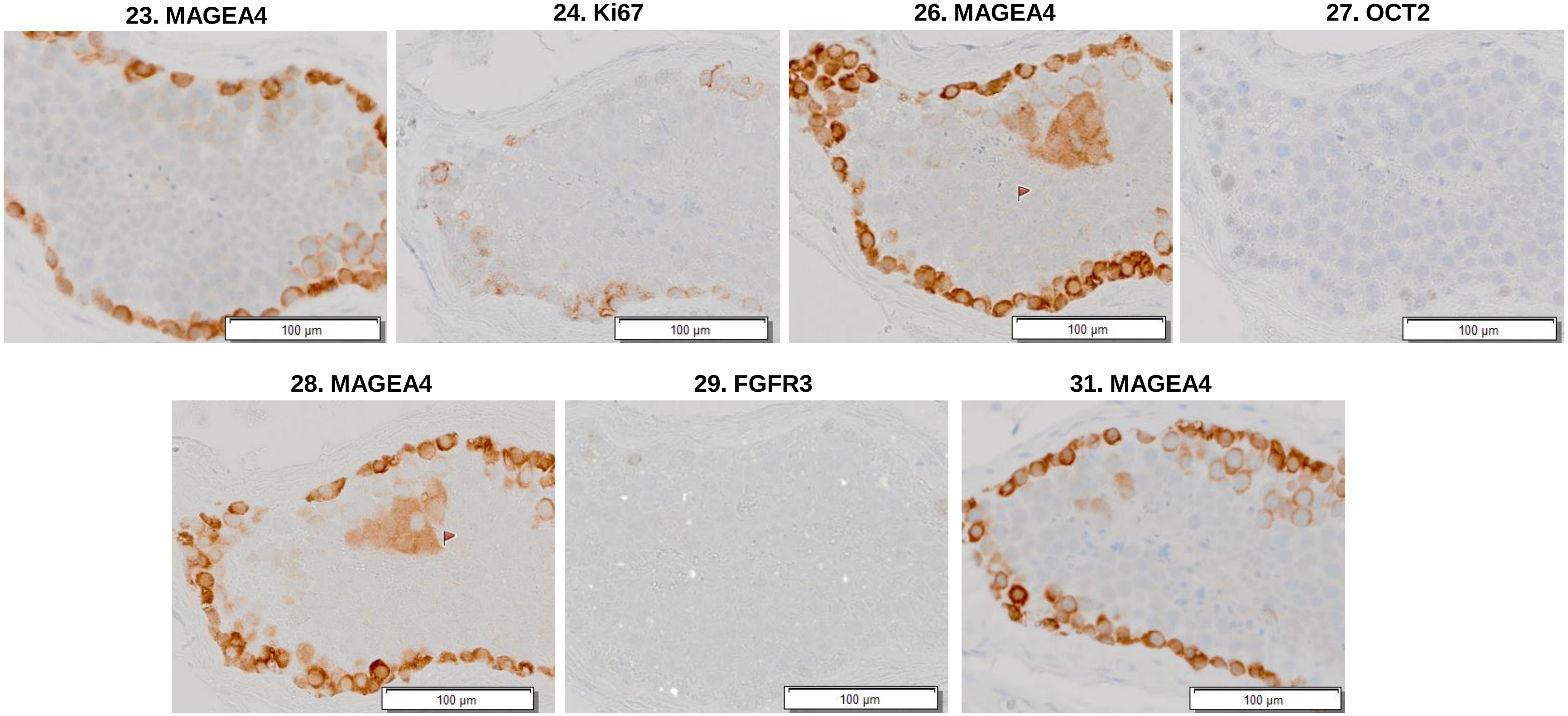

Clone no. 1-1_C24

A

Clone no.	Location	Slide 21	Slide 22	Slide 23	Slide 24	Slide 25	Slide 26	Slide 27	Slide 28	Slide 29	Slide 30	Slide 31	Minimum number of cells
		MAGEA4	SSX	MAGEA4	Ki67	H&E	MAGEA4	OCT2	MAGEA4	FGFR3	Ki67	MAGEA4	
1-1_C24	Periphery	NA	0	1<br>7 cells	0		1<br>15 cells	0	0*<br>4 cells	0	0	0	58
				30 µm									

B

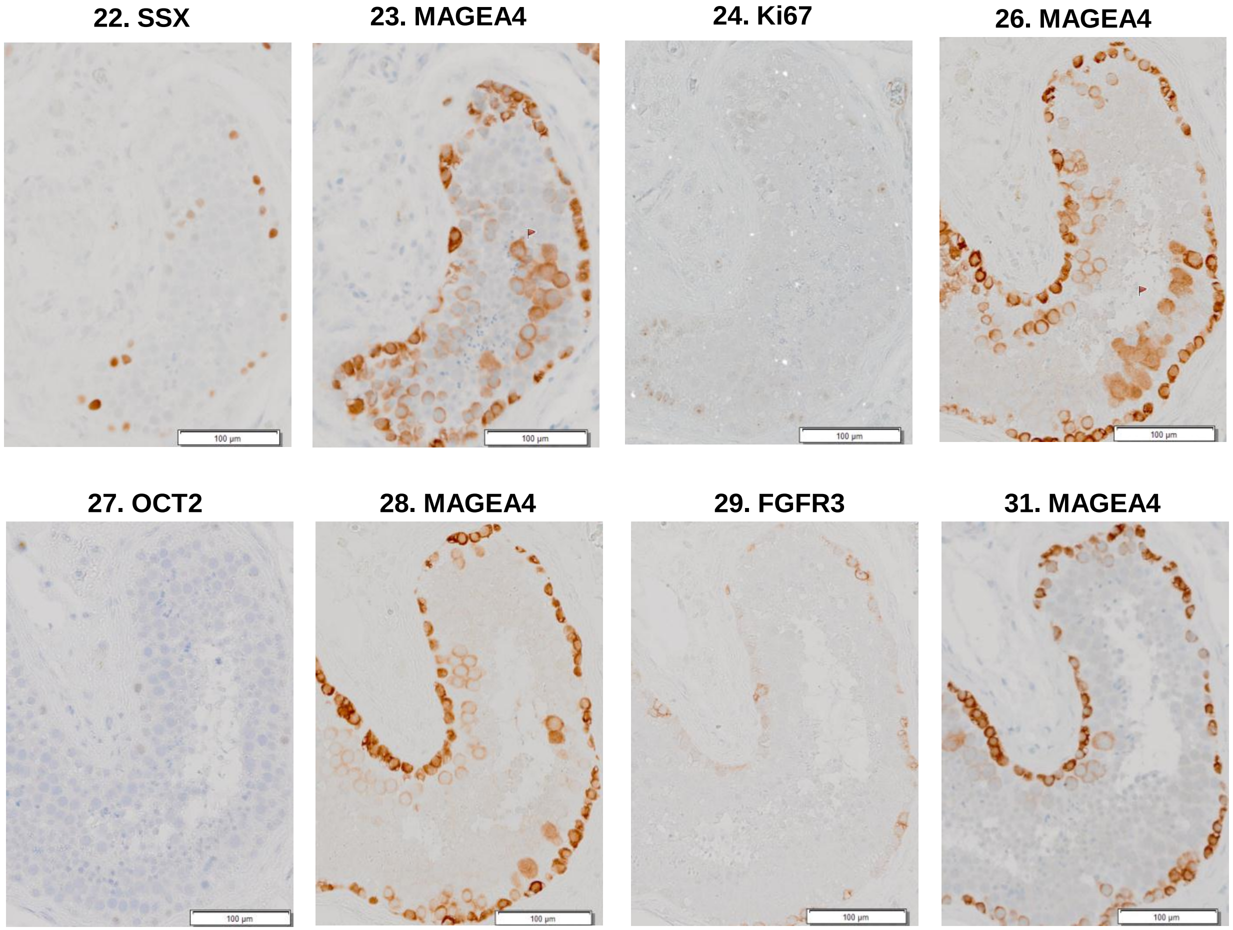

Clone no. 1-1_C25

A

Clone no.	Location	Slide 21	Slide 22	Slide 23	Slide 24	Slide 25	Slide 26	Slide 27	Slide 28	Slide 29	Slide 30	Slide 31	Minimum number of cells
		MAGEA4	SSX	MAGEA4	Ki67	H&E	MAGEA4	OCT2	MAGEA4	FGFR3	Ki67	MAGEA4	
1-1_C25	Near Peripher y		0	0*<br>1 cell	0		1<br>7 cells	0	1<br>4 cells	0	0	0	26
				30 µm									

B

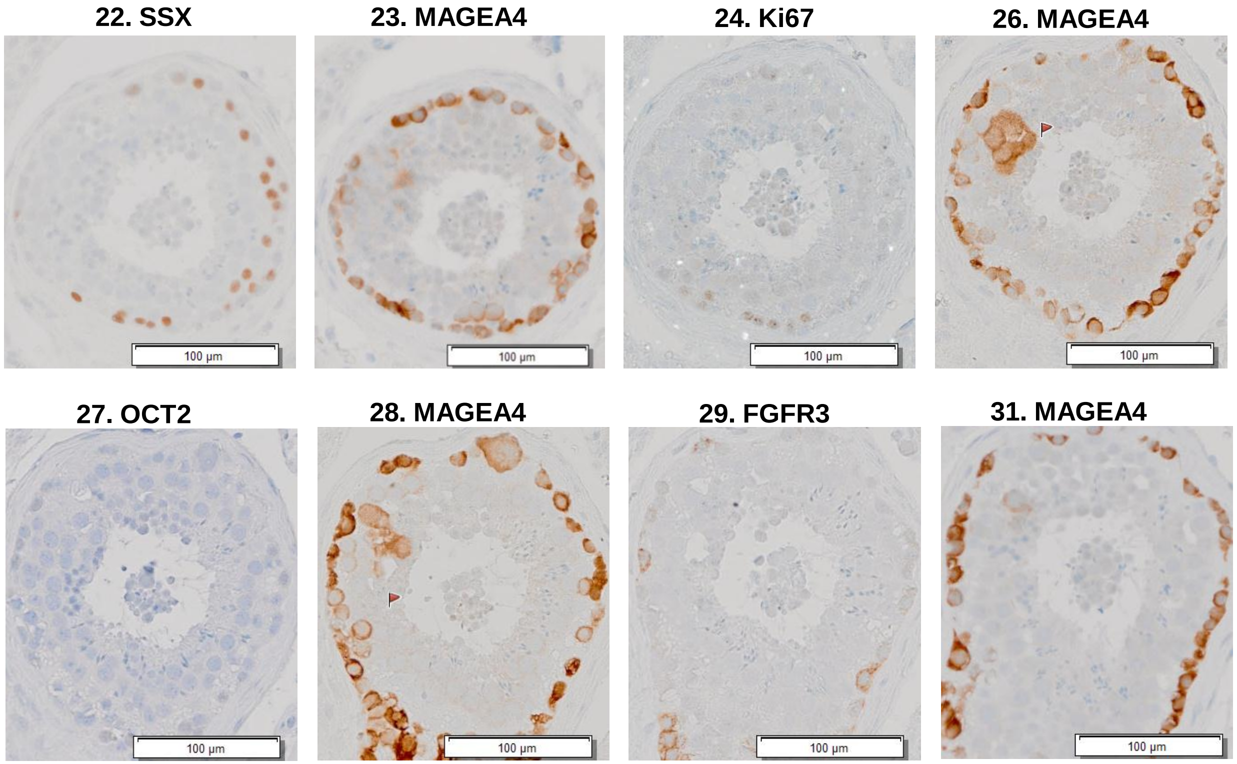

Clone no. 1-1_C26

A

Clone no.	Location	Slide 21	Slide 22	Slide 23	Slide 24	Slide 25	Slide 26	Slide 27	Slide 28	Slide 29	Slide 30	Slide 31	Minimum number of cells
		MAGEA4	SSX	MAGEA4	Ki67	H&E	MAGEA4	OCT2	MAGEA4	FGFR3	Ki67	MAGEA4	
1-1_C26	Near Periphery	0	0	0*<br>7 cells	0		1<br>8 cells	0	1<br>6 cells	0	0	0	43
				30 µm									

B

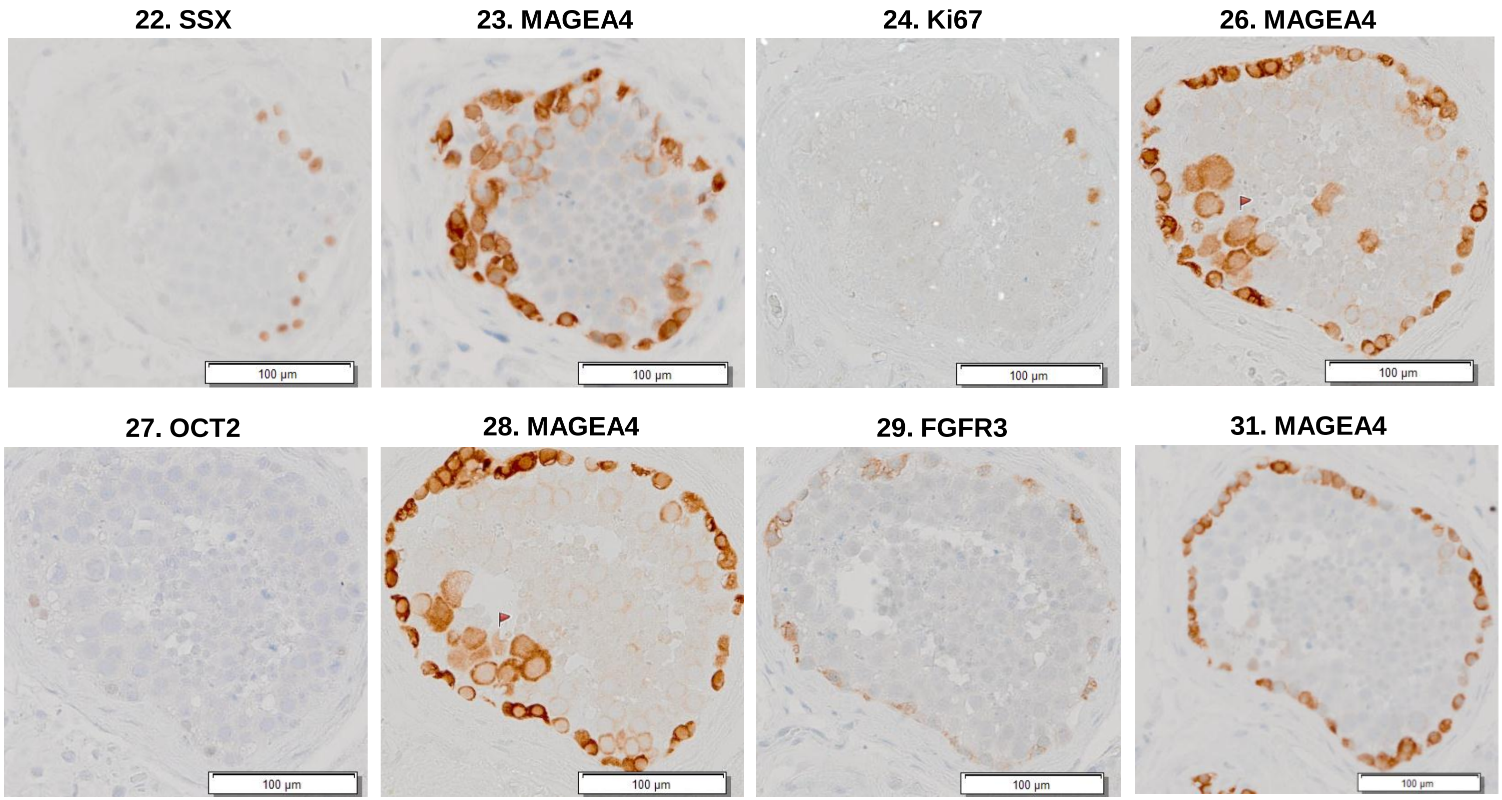

Clone no. 1-1_C27

A

Clone no.	Location	Slide 21	Slide 22	Slide 23	Slide 24	Slide 25	Slide 26	Slide 27	Slide 28	Slide 29	Slide 30	Slide 31	Slide 32	Slide 33	Slide 34	Slide 35	Sldie 36	Sldie 37	Sldie 38	Sldie 39	Sldie 40	Slide 41	Minimum number of cells
		MAGEA4	SSX	MAGEA4	Ki67	H&E	MAGEA4	OCT2	MAGEA4	FGFR3	Ki67	MAGEA4	SSX	SAGE1	OCT2	MAGEA4	n.s	n.s	n.s	n.s	n.s	MAGEA4	
1-1_C27	Centre	NA	0	1<br>4 cells			1<br>20 cells	0	1<br>22 cells	0	0	0*<br>5 cells	0	0	0	1<br>13 cells						0	163
				65 µm																			

B

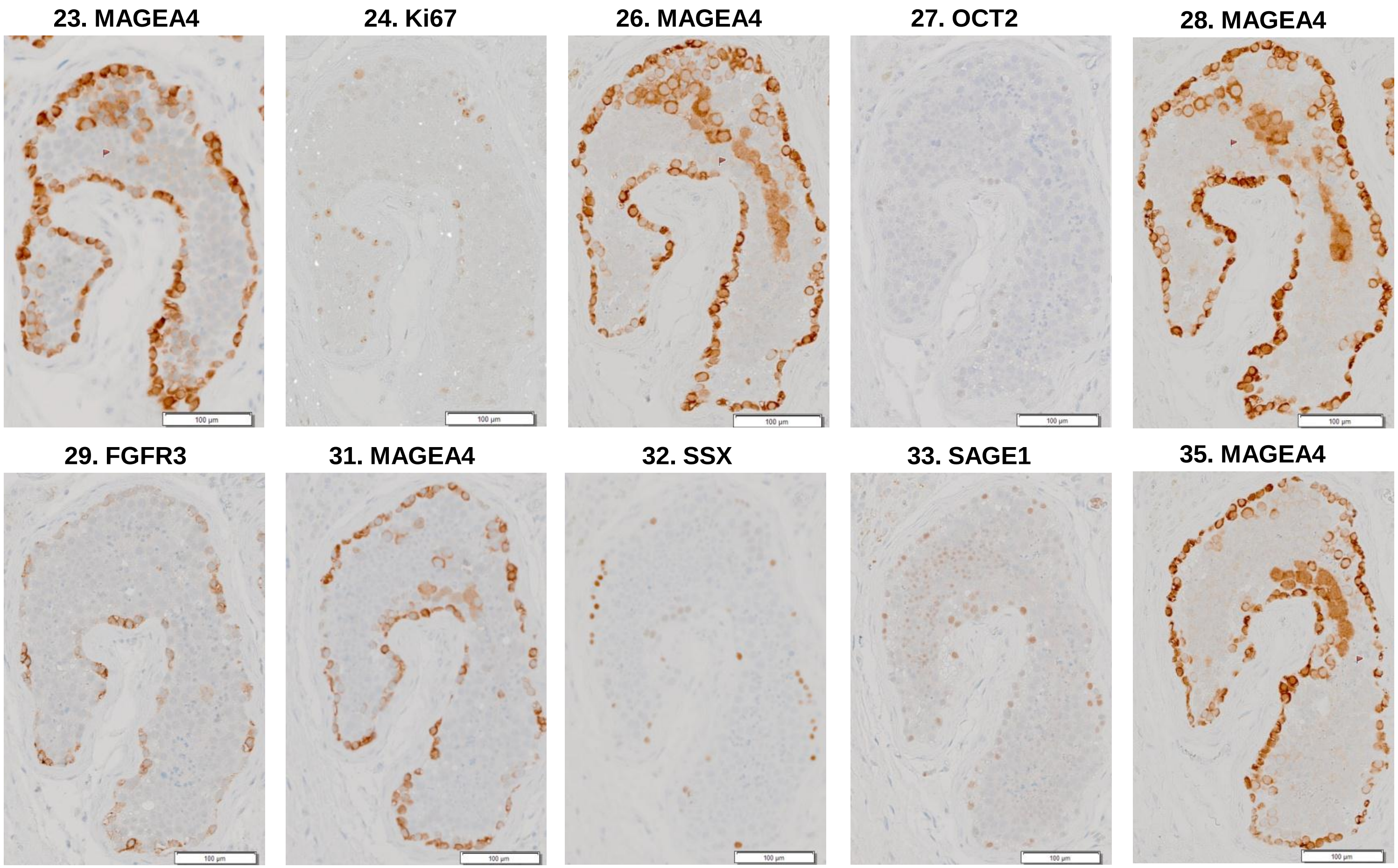

Clone no. 1-1_C28

A

Clone no.	Location	Slide 21	Slide 22	Slide 23	Slide 24	Slide 25	Slide 26	Slide 27	Slide 28	Slide 29	Slide 30	Slide 31	Minimum number of cells
		MAGEA4	SSX	MAGEA4	Ki67	H&E	MAGEA4	OCT2	MAGEA4	FGFR3	Ki67	MAGEA4	
1-1_C28	Centre	0	0	1<br>4 cells	0		1<br>5 cells	0	0*<br>2 cells	0	0	NA	24
				30 µm									

B

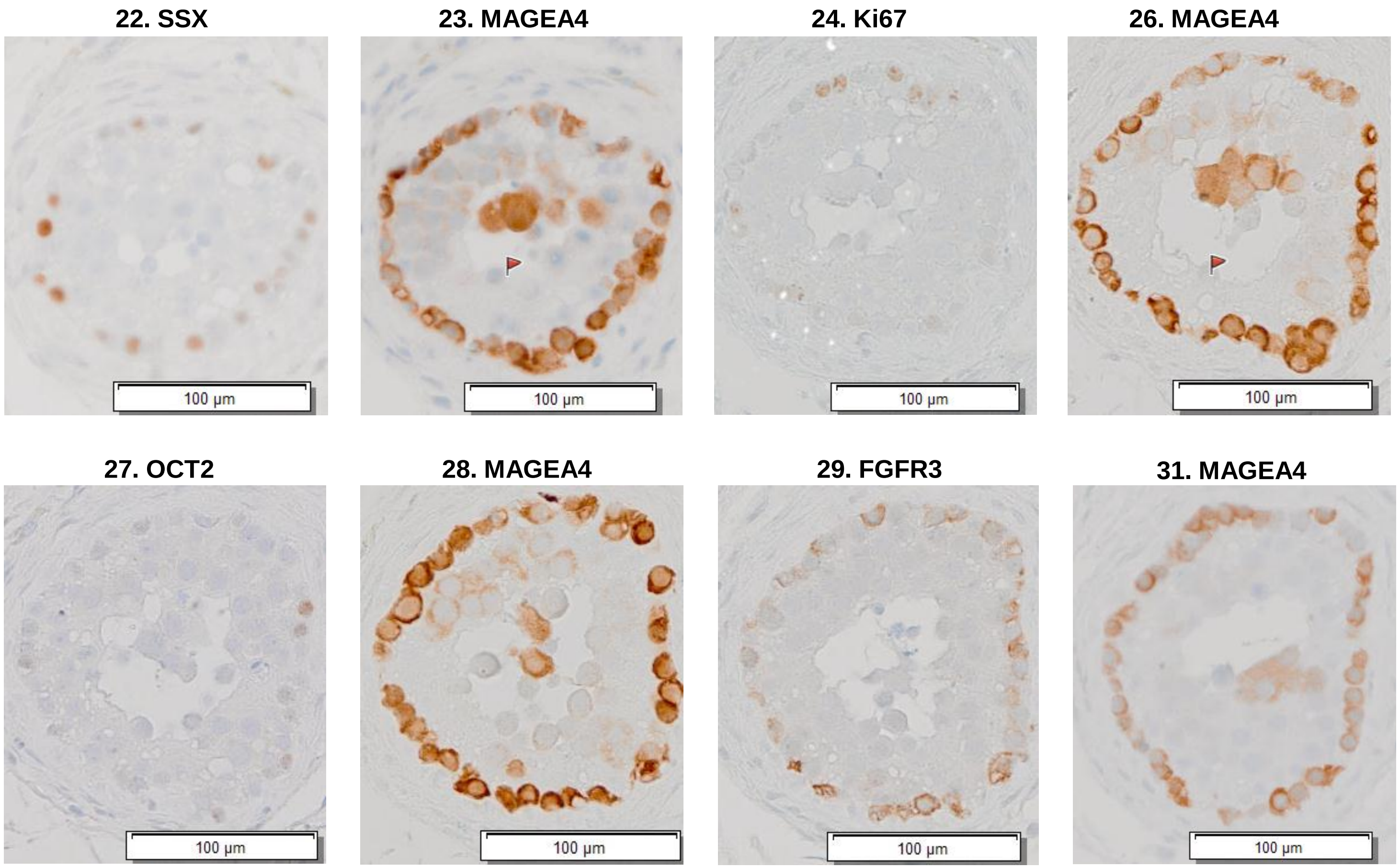

Clone no. 1-1_C29

A

Clone no.	Location	Slide 21	Slide 22	Slide 23	Slide 24	Slide 25	Slide 26	Slide 27	Slide 28	Slide 29	Slide 30	Slide 31	Minimum number of cells
		MAGEA4	SSX	MAGEA4	Ki67	H&E	MAGEA4	OCT2	MAGEA4	FGFR3	Ki67	MAGEA4	
1-1_C29	Centre	0	0	0*	0		1	0	1	0	0	0	42
				5 cells			9 cells		6 cells				
				30 µm									

B

22. SSX

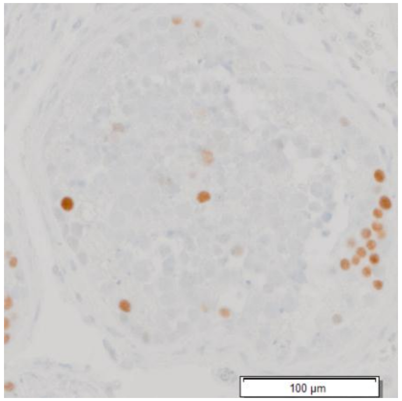

23. MAGEA4

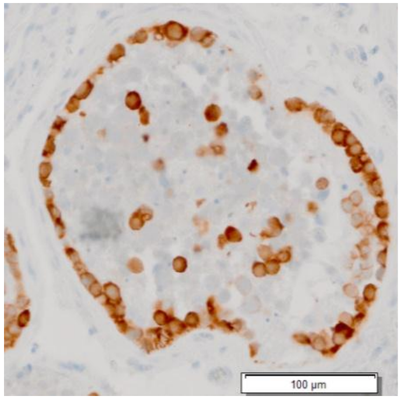

24. Ki67

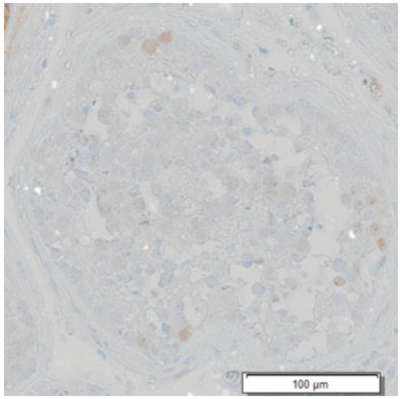

26. MAGEA4

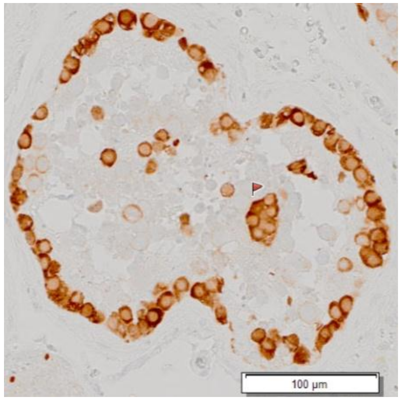

27. OCT2

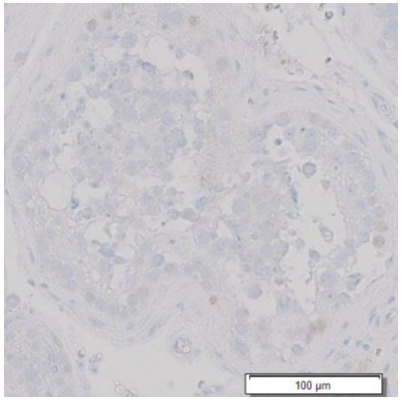

28. MAGEA4

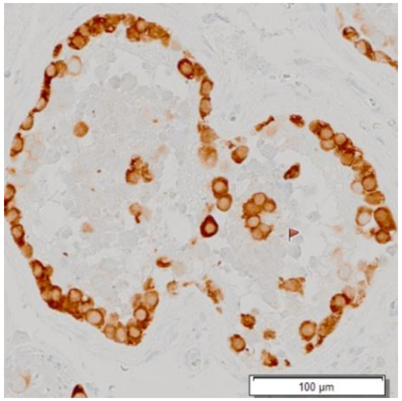

29. FGFR3

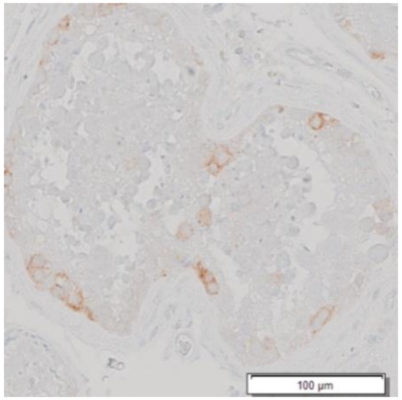

31. MAGEA4

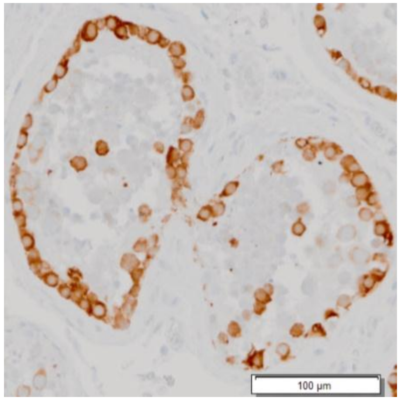

Clone no. 1-1_C30

A

Clone no.	Location	Slide 23	Slide 24	Slide 25	Slide 26	Slide 27	Slide 28	Slide 29	Slide 30	Slide 31	Minimum number of cells
		MAGEA4	Ki67	H&E	MAGEA4	OCT2	MAGEA4	FGFR3	Ki67	MAGEA4	
1-1_C30	Centre	0	0		1	0	1	0	0	0	15
					5 cells		5 cells				
					15 µm						

B

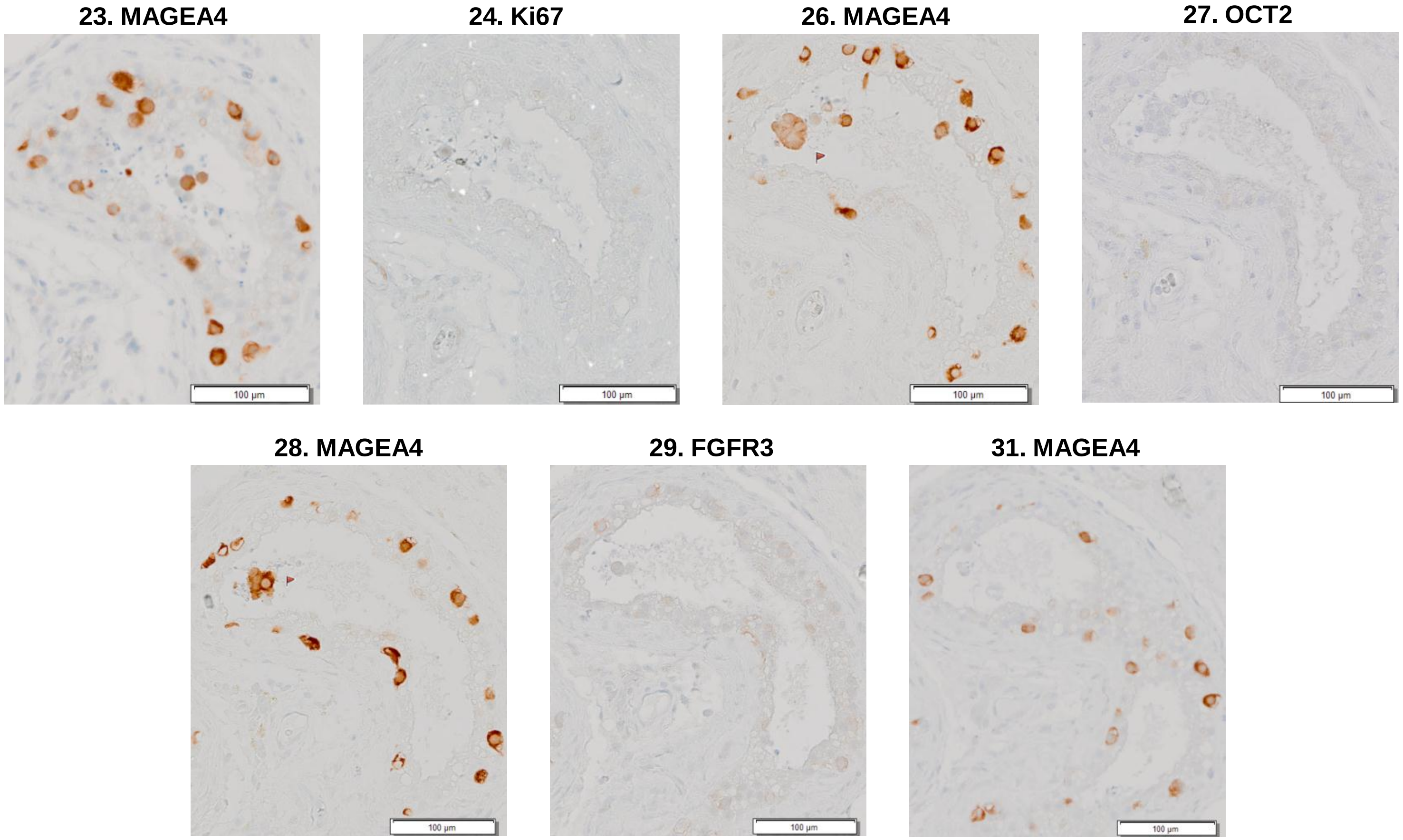

Clone no. 1-1_C31

A

<table>
<tr><th rowspan="2">Clone no.</th><th rowspan="2">Location</th><th>Slide 23</th><th>Slide 24</th><th>Slide 25</th><th>Slide 26</th><th>Slide 27</th><th>Slide 28</th><th>Slide 29</th><th>Slide 30</th><th>Slide 31</th><th>Slide 32</th><th>Slide 33</th><th rowspan="2">Minimum number of cells</th></tr>
<tr><th>MAGEA4</th><th>Ki67</th><th>H&E</th><th>MAGEA4</th><th>OCT2</th><th>MAGEA4</th><th>FGFR3</th><th>Ki67</th><th>MAGEA4</th><th>SSX</th><th>SAGE1</th></tr>
<tr><td rowspan="3">1-1_C31</td><td rowspan="3">Near Periphery</td><td rowspan="3">0</td><td rowspan="3">0</td><td rowspan="3"></td><td>1</td><td rowspan="2">0</td><td>1</td><td rowspan="2">0</td><td rowspan="2">0</td><td>0*</td><td rowspan="3">0</td><td rowspan="3">0</td><td rowspan="3">41</td></tr>
<tr><td>6 cells</td><td>11 cells</td><td>2 cells</td></tr>
<tr><td colspan="6">30 µm</td></tr>
</table>

B

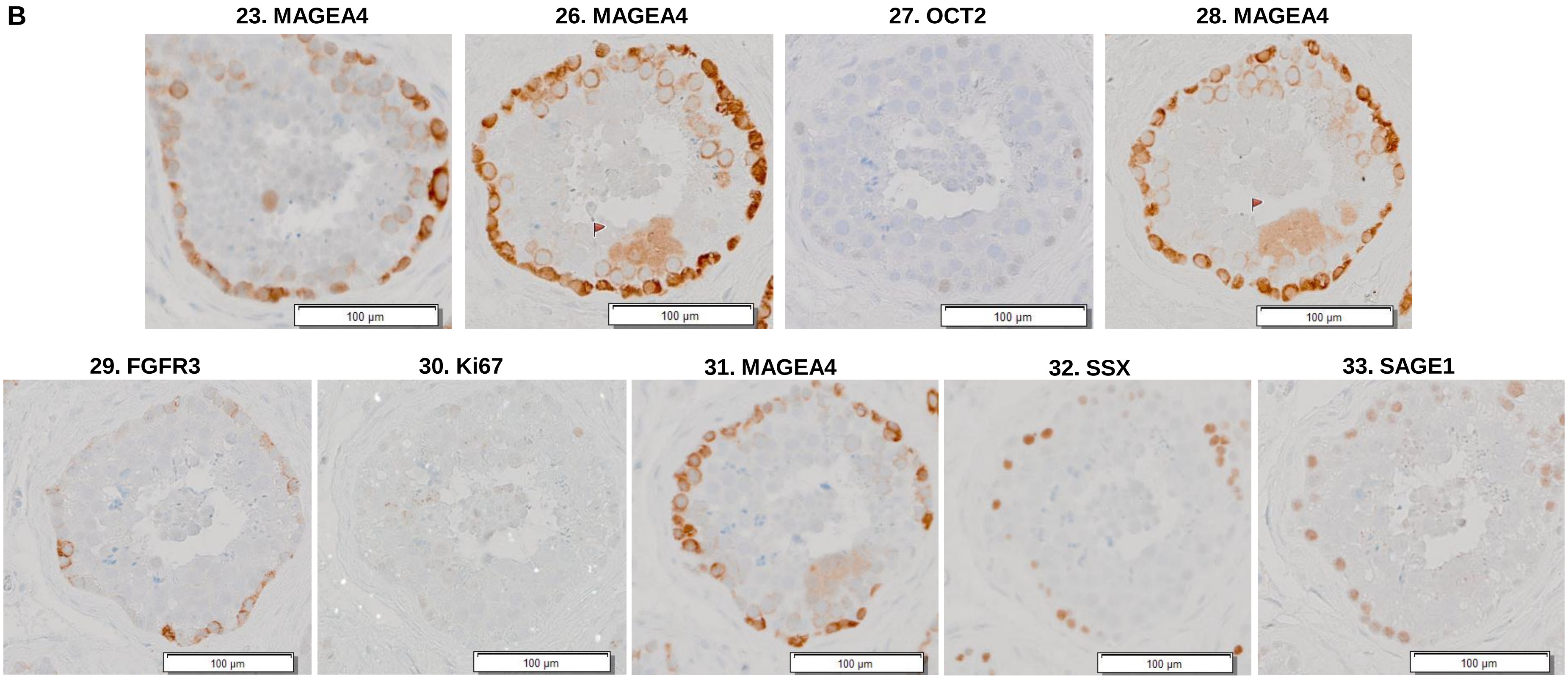

Clone no. 1-1_C32

A

Clone no.	Location	Slide 21	Slide 22	Slide 23	Slide 24	Slide 25	Slide 26	Slide 27	Slide 28	Slide 29	Slide 30	Slide 31	Minimum number of cells
		MAGEA4	SSX	MAGEA4	Ki67	H&E	MAGEA4	OCT2	MAGEA4	FGFR3	Ki67	MAGEA4	
1-1_C32	Near Periphery	0	0	1<br>7 cells			1<br>6 cells	0	1<br>7 cells	0	0	0	40
				30 µm									

B

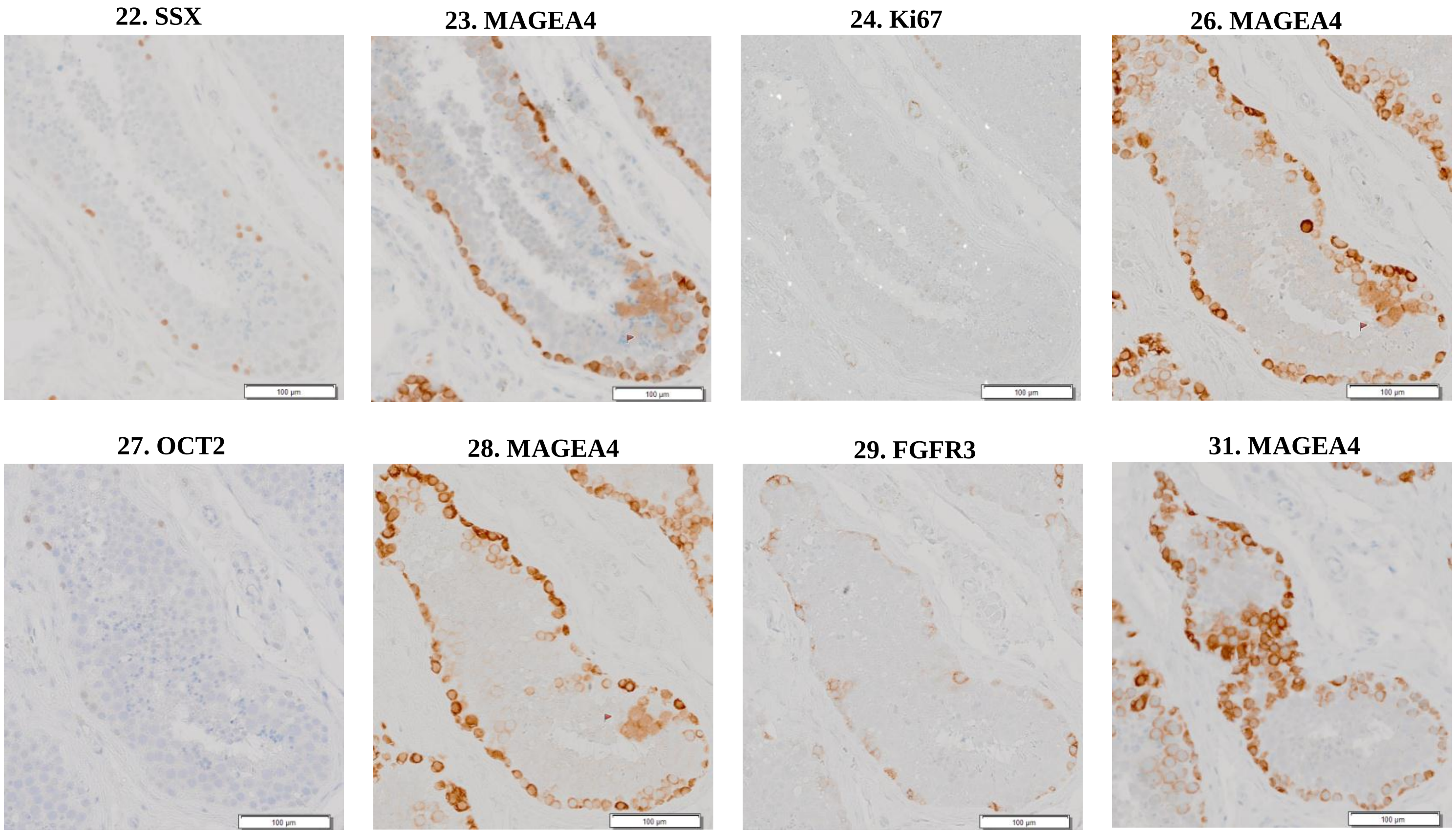

Clone no. 1-1_C33

A

Clone no.	Location	Slide 23	Slide 24	Slide 25	Slide 26	Slide 27	Slide 28	Slide 29	Slide 30	Slide 31	Slide 32	Slide 33	Minimum number of cells
		MAGEA4	Ki67	n.s	MAGEA4	OCT2	MAGEA4	FGFR3	Ki67	MAGEA4	SSX	SAGE1	
1-1_C33	Centre	NA	0		1<br>8 cells	0	1<br>10 cells	1<br>3 cells	0	1<br>8 cells	NA	NA	53
					30 µm								

B

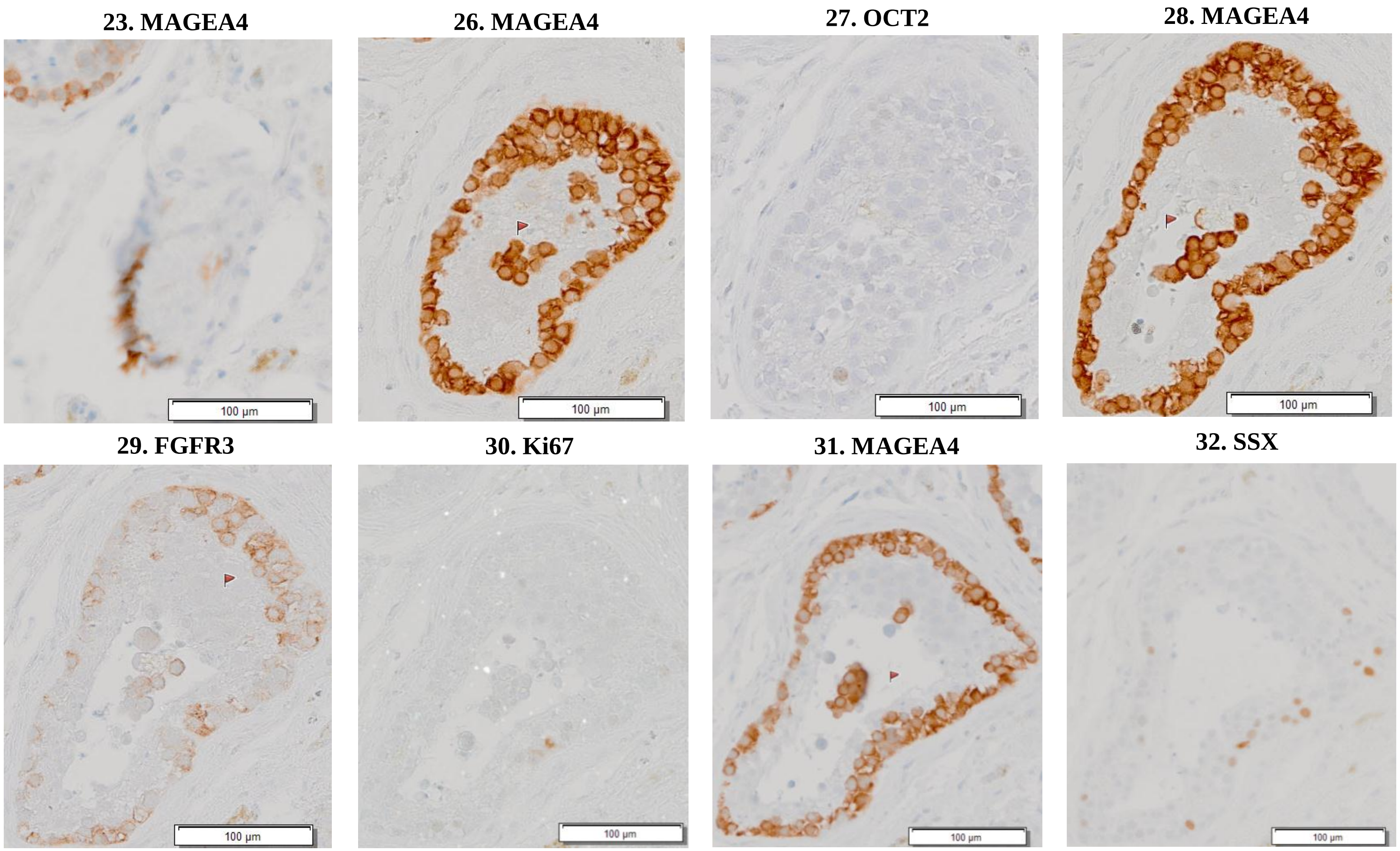

Clone no. 1-1_C34

A

Clone no.	Location	Slide 23	Slide 24	Slide 25	Slide 26	Slide 27	Slide 28	Slide 29	Slide 30	Slide 31	Slide 32	Slide 33	Slide 34	Slide 35	Minimum number of cells
		MAGEA4	Ki67	H&E	MAGEA4	OCT2	MAGEA4	FGFR3	Ki67	MAGEA4	SSX	SAGE1	OCT2	MAGEA4	
1-1_C34	Near Periphery	0			0*<br>3 cells	0	1<br>5 cells	0	0	1<br>8 cells	0	0	0	0*<br>2 cells	51
					50 µm										

B

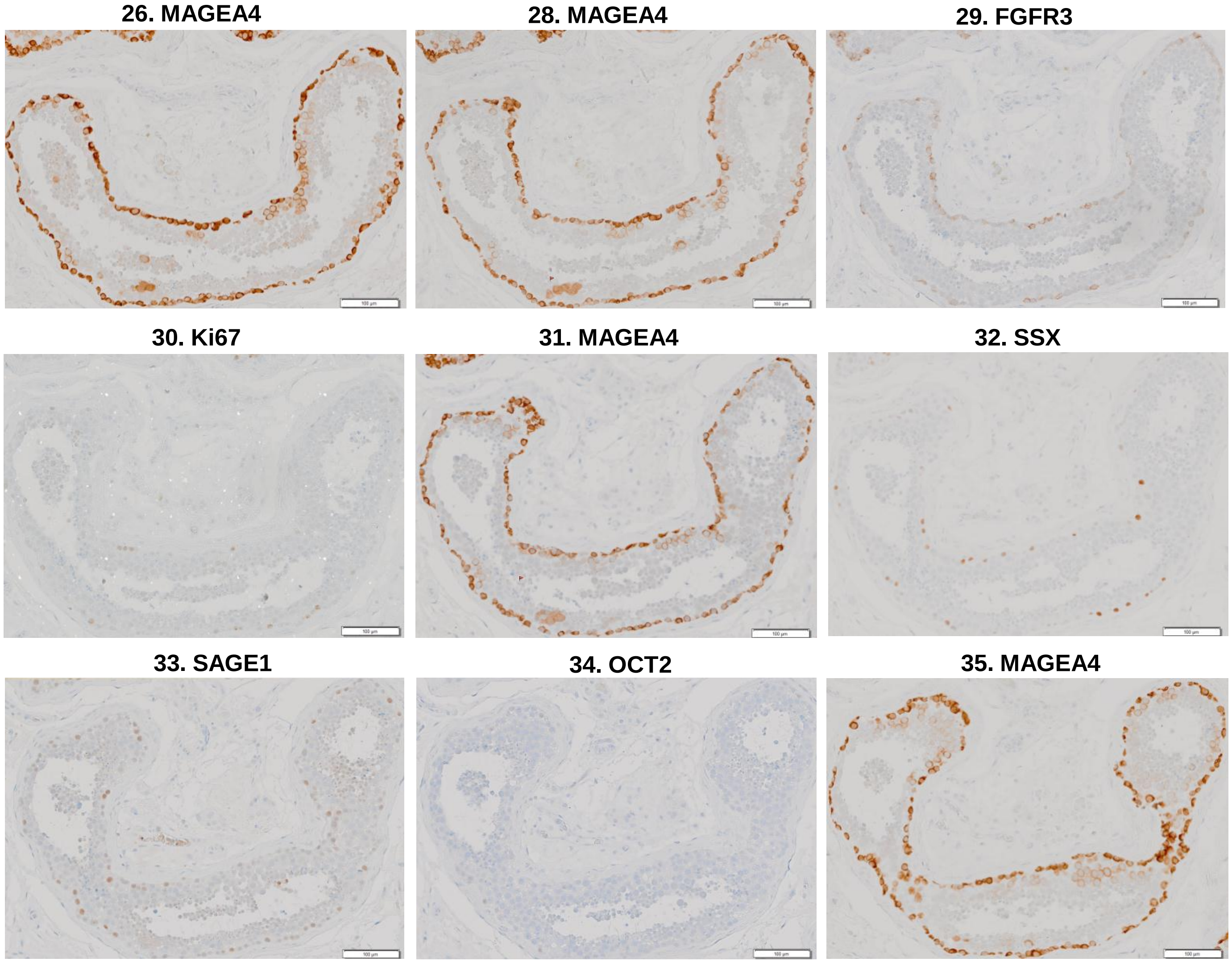

Clone no. 1-1_C35

A

Clone no.	Location	Slide 23	Slide 24	Slide 25	Slide 26	Slide 27	Slide 28	Slide 29	Slide 30	Slide 31	Slide 32	Slide 33	Slide 34	Slide 35	Sldie 36	Sldie 37	Sldie 38	Sldie 39	Sldie 40	Slide 41	Minimum number of cells
		MAGEA4	Ki67	H&E	MAGEA4	OCT2	MAGEA4	FGFR3	Ki67	MAGEA4	SSX	SAGE1	OCT2	MAGEA4	n.s	n.s	n.s	n.s	n.s	MAGEA4	
1-1_C35	Near Periphery	0*			0*	0	0*	0	0	1	0	0	0	1						0	72
		2 cells			3 cells		2 cells			12 cells				5 cells							
		65 µm																			

B

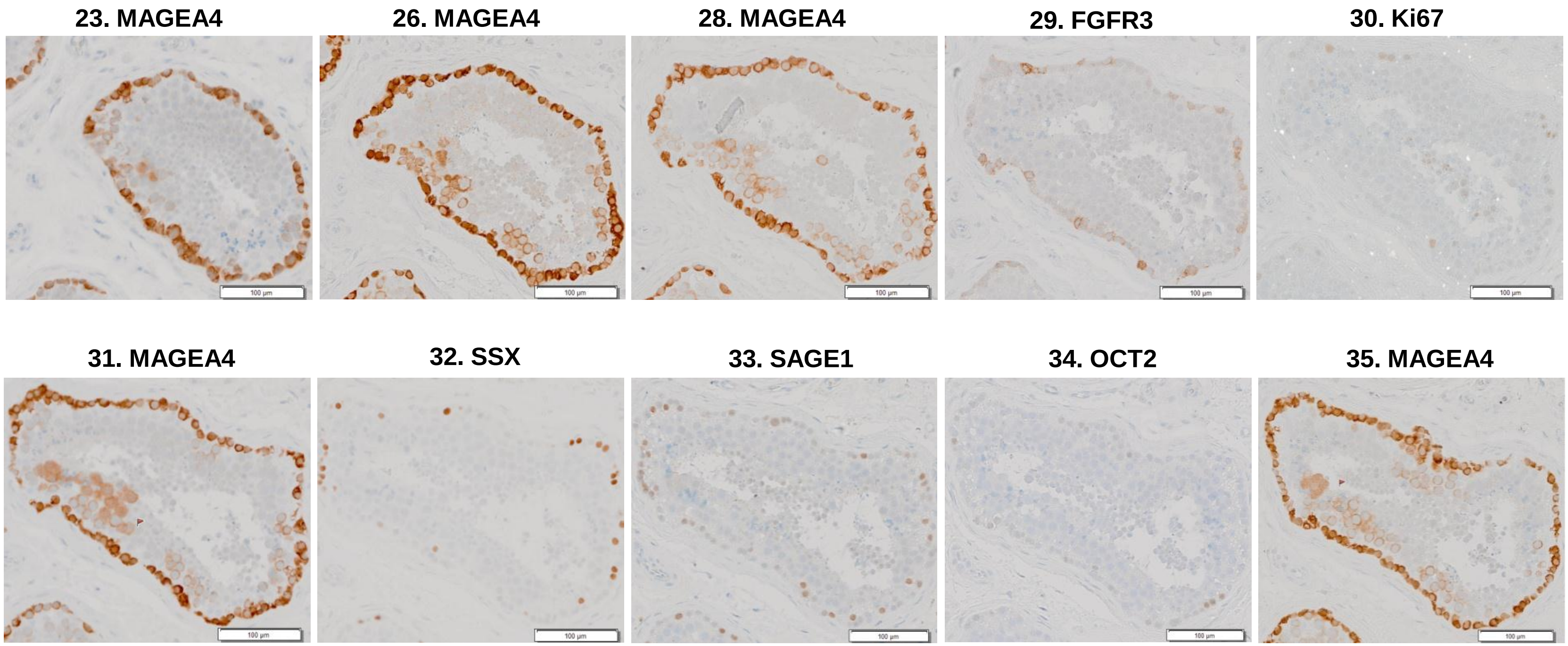

Clone no. 1-1_C36

A

Clone no.	Location	Slide 26	Slide 27	Slide 28	Slide 29	Slide 30	Slide 31	Slide 32	Slide 33	Slide 34	Slide 35	Minimum number of cells
		MAGEA4	OCT2	MAGEA4	FGFR3	Ki67	MAGEA4	SSX	SAGE1	OCT2	MAGEA4	
1-1_C36	Centre	0*<br>4 cells	0	1<br>7 cells	0*<br>1 cell	1<br>9 cells	1<br>21 cells	1<br>17 cells	1<br>15 cells	0	0	98
		40 µm										

B

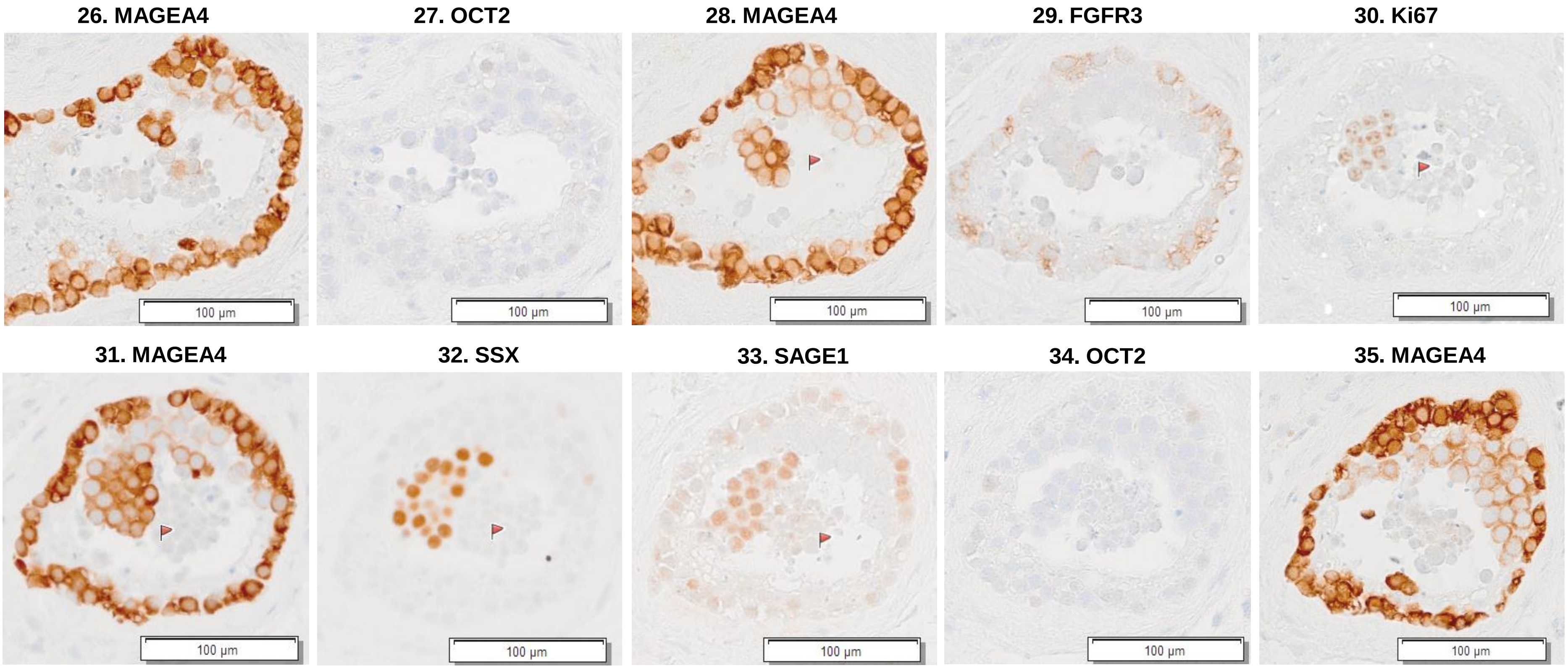

Clone no. 1-1_C37

A

Clone no.	Location	Slide 26	Slide 27	Slide 28	Slide 29	Slide 30	Slide 31	Slide 32	Slide 33	Slide 34	Slide 35	Minimum number of cells
		MAGEA4	OCT2	MAGEA4	FGFR3	Ki67	MAGEA4	SSX	SAGE1	OCT2	MAGEA4	
1-1_C37	Centre	0	0	1	0*	0	1	0	1	0	0	55
				9 cells	2 cells		13 cells		3 cells			
				30 µm								

B

26. MAGEA4 | 27. OCT2 | 28. MAGEA4 | 29. FGFR3 | 30. Ki67

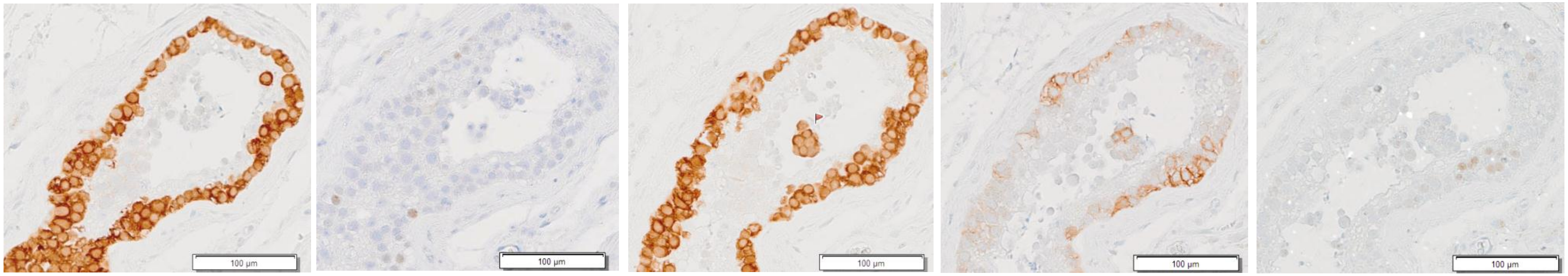

31. MAGEA4 | 32. SSX | 33. SAGE1 | 34. OCT2 | 35. MAGEA4

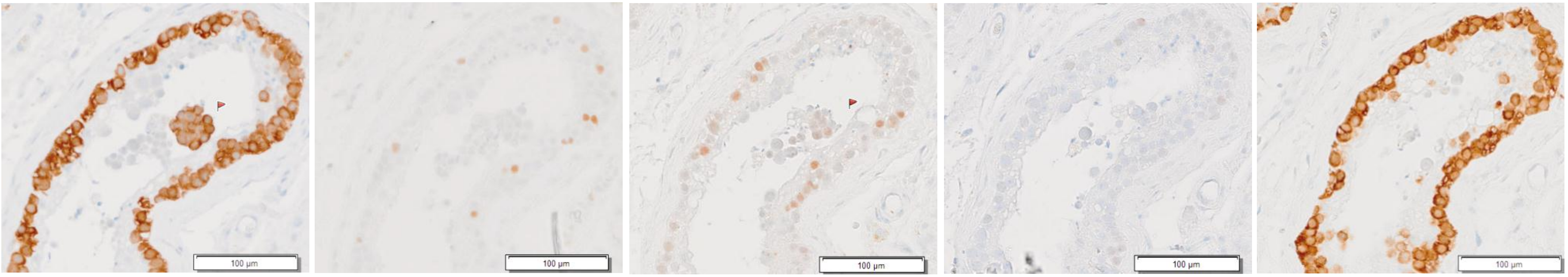

Clone no. 1-1_C38

A

Clone no.	Location	Slide 26	Slide 27	Slide 28	Slide 29	Slide 30	Slide 31	Slide 32	Slide 33	Slide 34	Slide 35	Minimum number of cells
		MAGEA4	OCT2	MAGEA4	FGFR3	Ki67	MAGEA4	SSX	SAGE1	OCT2	MAGEA4	
1-1_C38	Near Periphery	0*<br>2 cells	0	1<br>6 cells	0	0	1<br>5 cells	0	0	0	0	28
		30 µm										

B

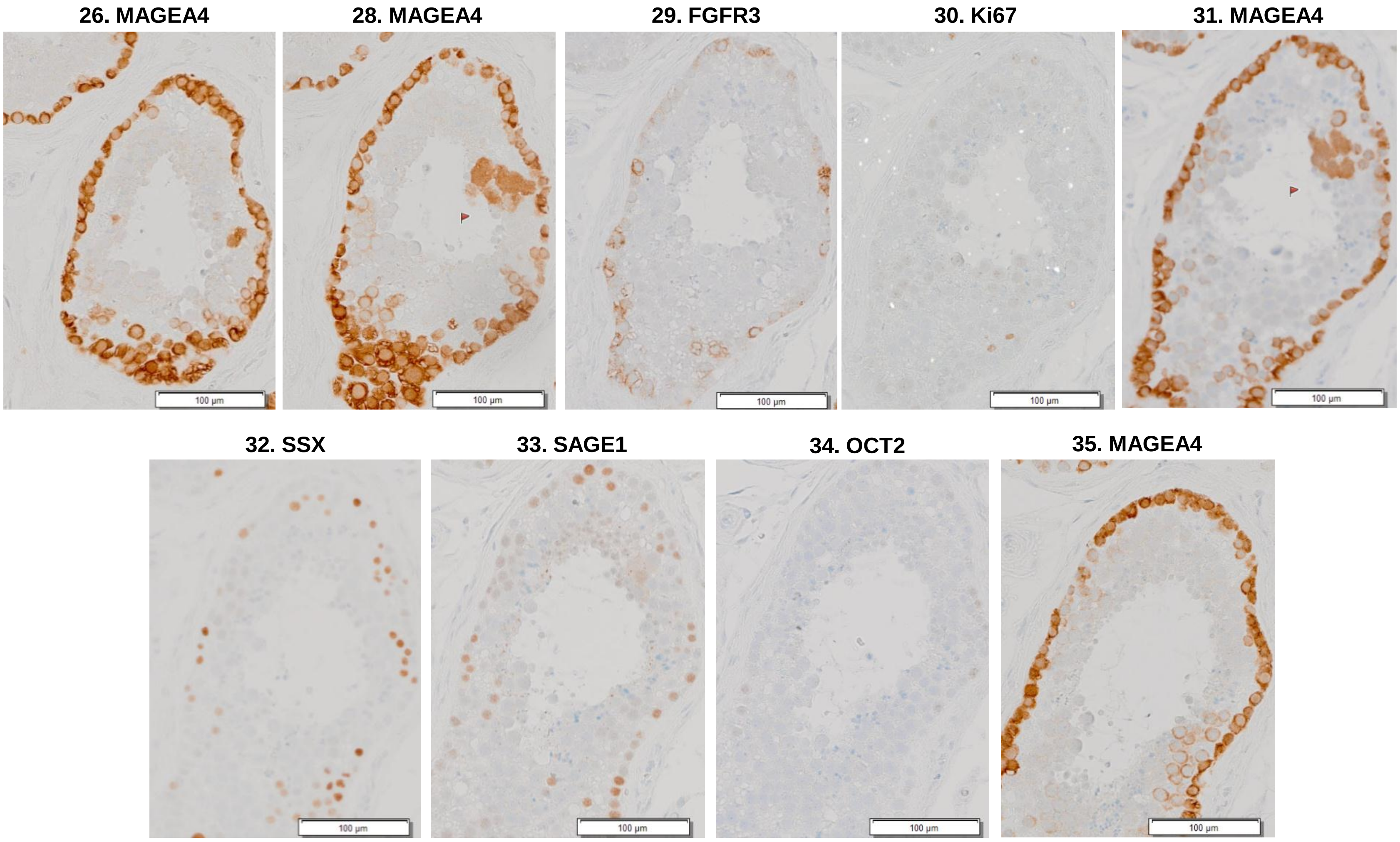

Clone no. 1-1_C39

A

Clone no.	Location	Slide 28	Slide 29	Slide 30	Slide 31	Slide 32	Slide 33	Slide 34	Slide 35	Minimum number of cells
		MAGEA4	FGFR3	Ki67	MAGEA4	SSX	SAGE1	OCT2	MAGEA4	
1-1_C39	Periphery	0	0	0	0	1	1	0	NA	35
						18 cells	17 cells			
						10 µm				

B

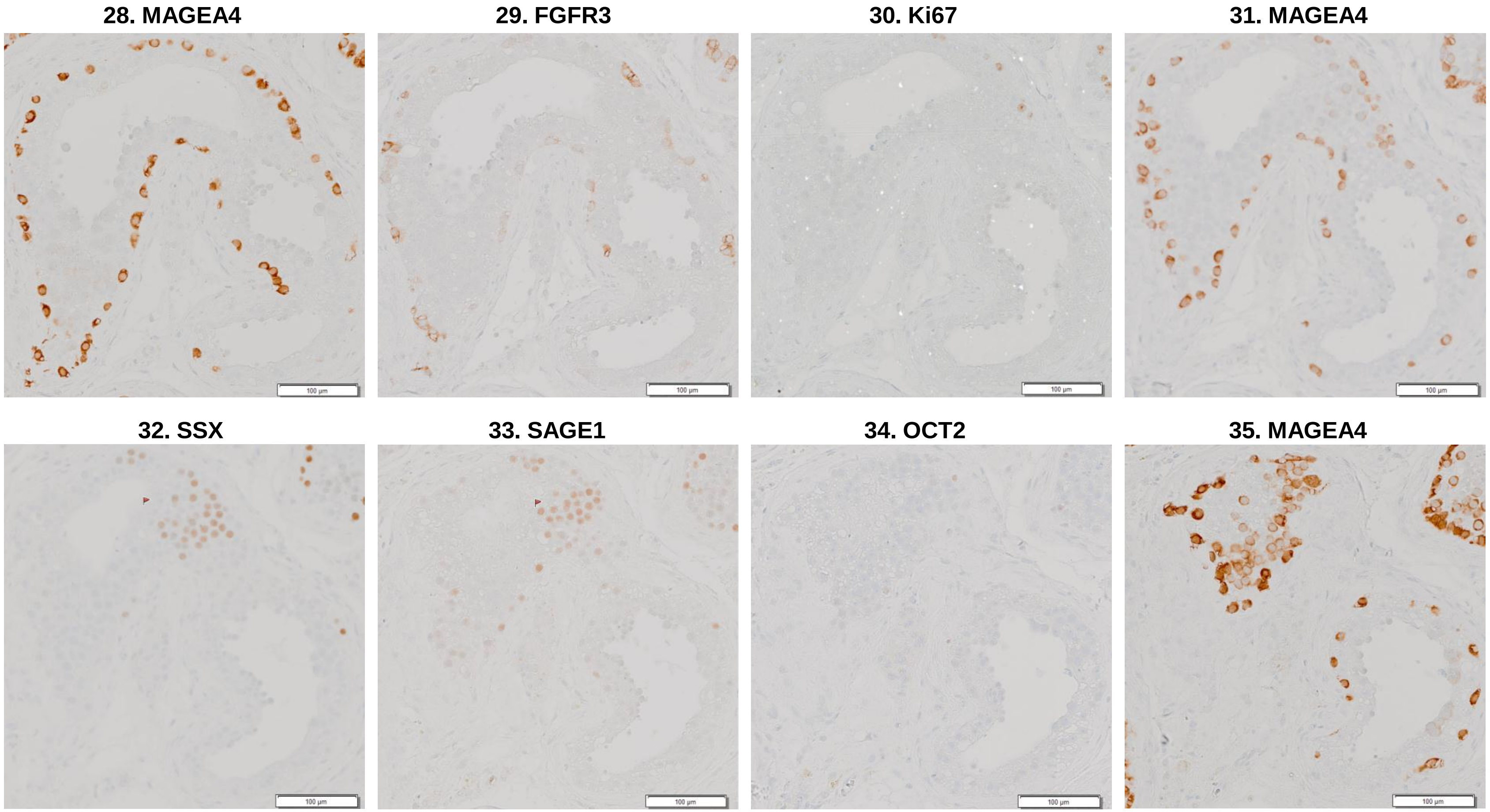

Clone no. 1-1_C40

A

Clone no.	Location	Slide 28	Slide 29	Slide 30	Slide 31	Slide 32	Slide 33	Slide 34	Slide 35	Sldie 36	Sldie 37	Sldie 38	Sldie 39	Sldie 40	Slide 41	Minimum number of cells
		MAGEA4	FGFR3	Ki67	MAGEA4	SSX	SAGE1	OCT2	MAGEA4	n.s	n.s	n.s	n.s	n.s	MAGEA4	
1-1_C40	Periphery	0	0*<br>14 cells	0	0*<br>15 cells	1<br>38 cells	1<br>20 cells	0	1<br>23 cells						0	124
			35 µm													

B

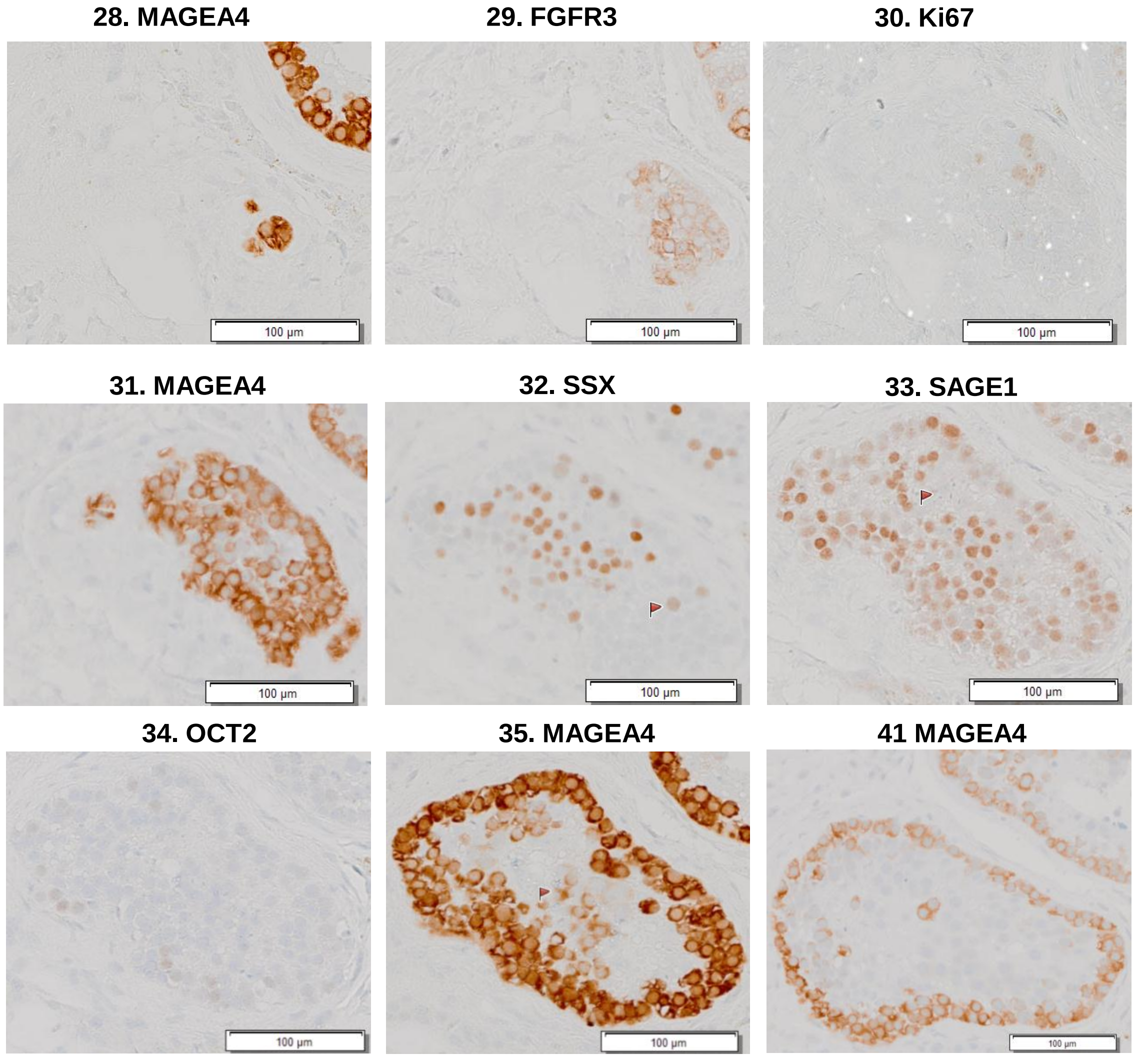

Clone no. 1-1_C41

A

Clone no.	Location	Slide 29	Slide 30	Slide 31	Slide 32	Slide 33	Slide 34	Minimum number of cells
		FGFR3	Ki67	MAGEA4	SSX	SAGE1	OCT2	
1-1_C41	Centre	0	0	0	1<br>10 cells	1<br>14 cells	0	24
					10 µm			

B

29. FGFR3 30. Ki67 31. MAGEA4 32. SSX 33. SAGE1 34. OCT2

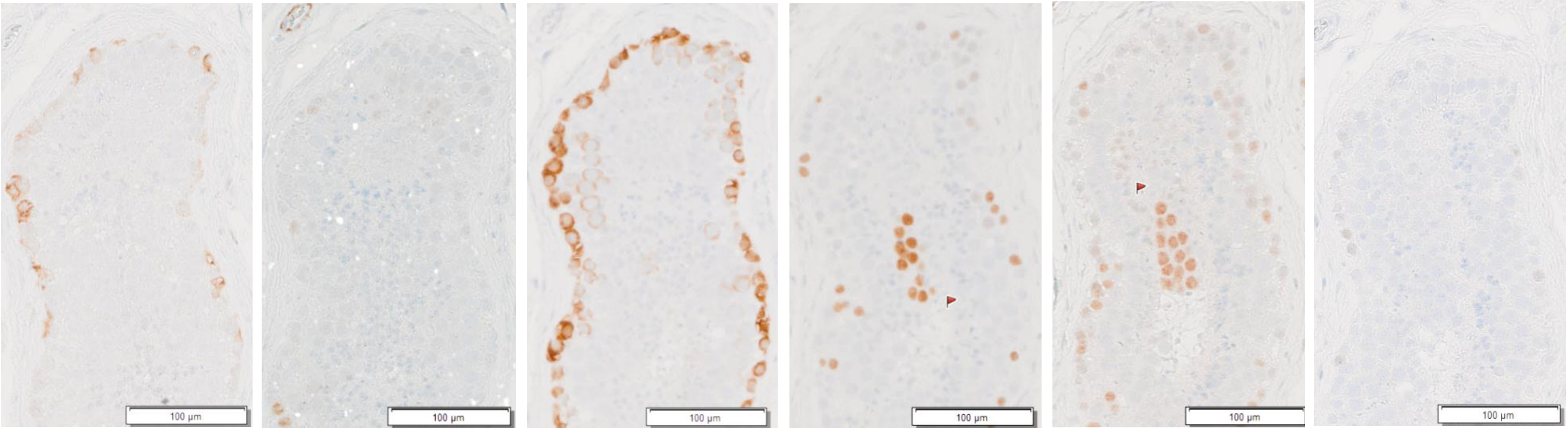

Clone no. 1-1_C42

A

Clone no.	Location	Slide 28	Slide 29	Slide 30	Slide 31	Slide 32	Slide 33	Slide 34	Slide 35	Slide 36	Slide 37	Slide 38	Slide 39	Slide 40	Slide 41	Minimum number of cells
		MAGEA4	FGFR3	Ki67	MAGEA4	SSX	SAGE1	OCT2	MAGEA4	n.s	n.s	n.s	n.s	n.s	MAGEA4	
1-1_C42	Near Periphery	0	0	0	1<br>3 cells	0	0	0	1<br>4 cells						0*<br>6 cells	49
					55 µm											

B

28. MAGEA4

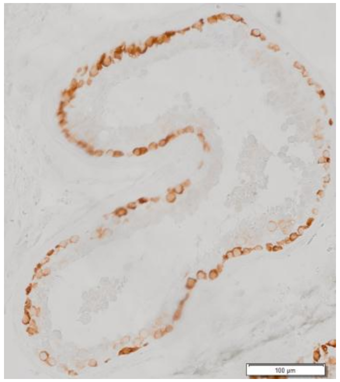

29. FGFR3

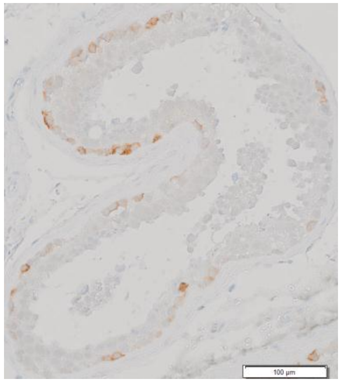

30. Ki67

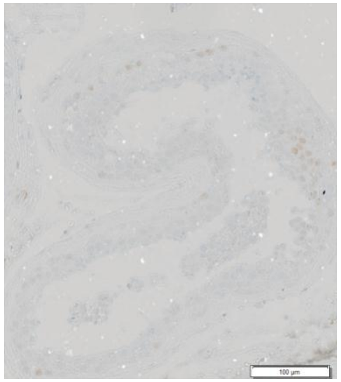

31. MAGEA4

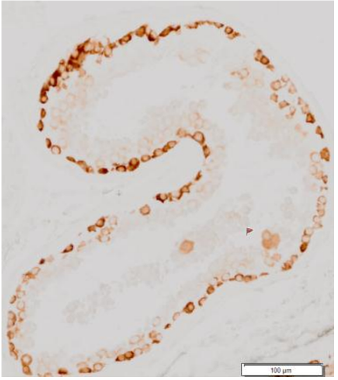

32. SSX

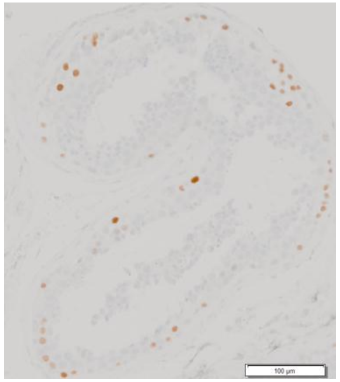

33. SAGE1

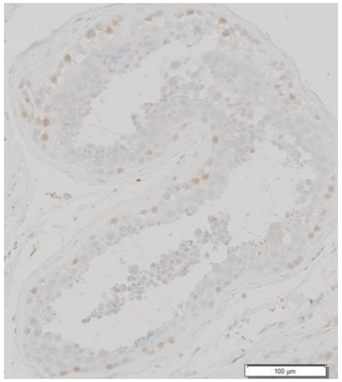

34. OCT2

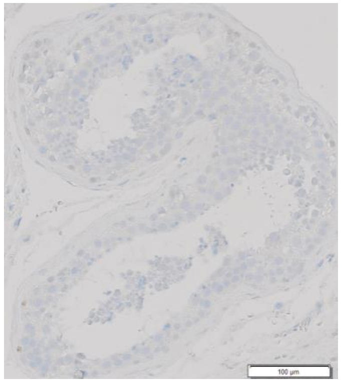

35. MAGEA4

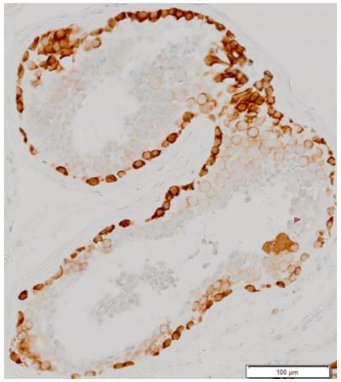

41. MAGEA4

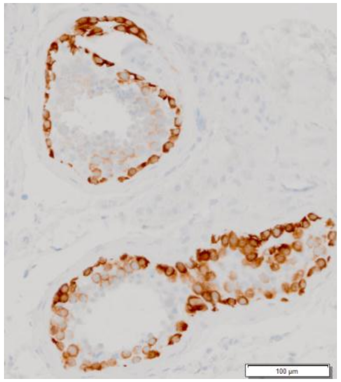

Clone no. 1-1_C43

A

Clone no.	Location	Slide 31	Slide 32	Slide 33	Slide 34	Slide 35	Slide 36	Slide 37	Slide 38	Slide 39	Slide 40	Slide 41	Minimum number of cells
		MAGEA4	SSX	SAGE1	OCT2	MAGEA4	n.s	n.s	n.s	n.s	n.s	MAGEA4	
1-1_C43	Near Periphery	0	0	0	0	1<br>7 cells						1<br>5 cells	42
						35 µm							

B

31. MAGEA4

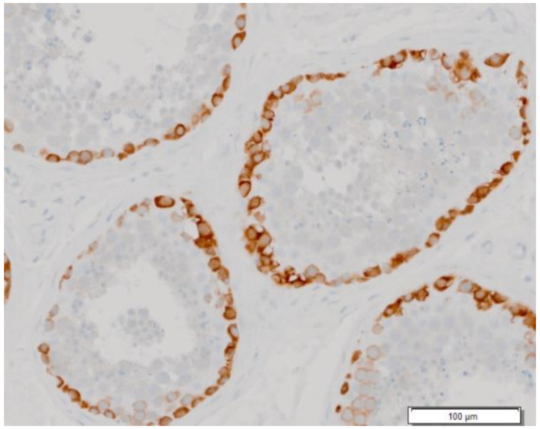

32. SSX

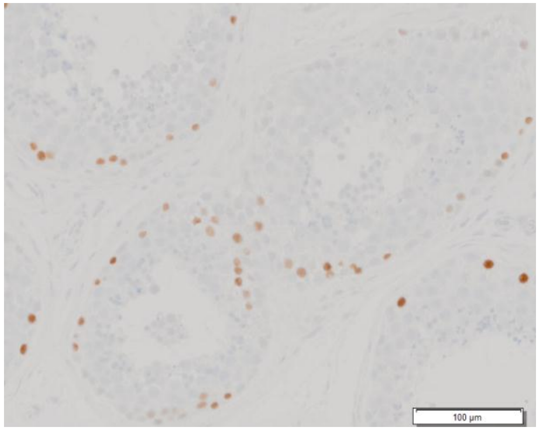

33. SAGE1

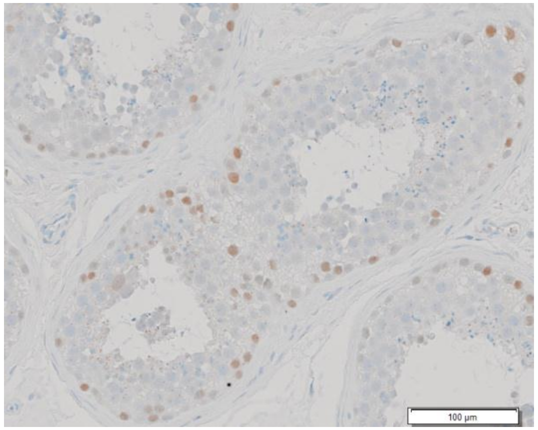

34. OCT2

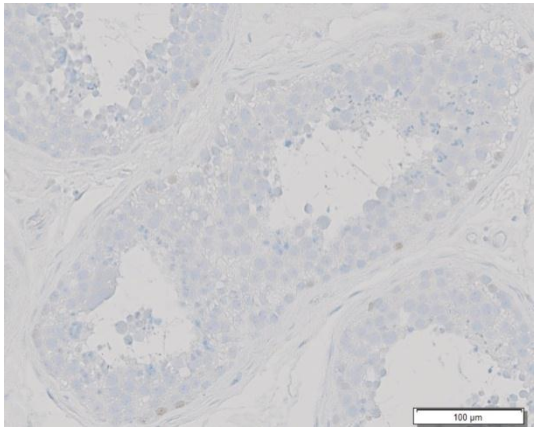

35. MAGEA4

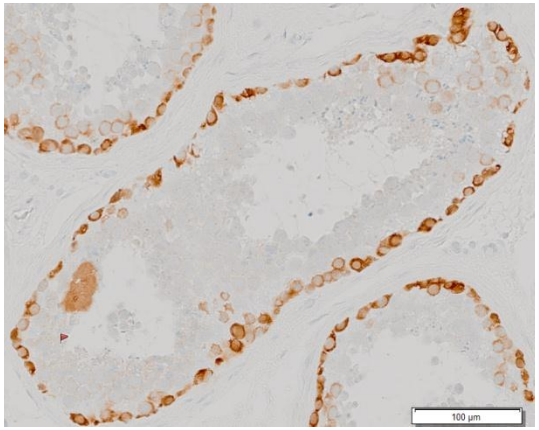

41. MAGEA4

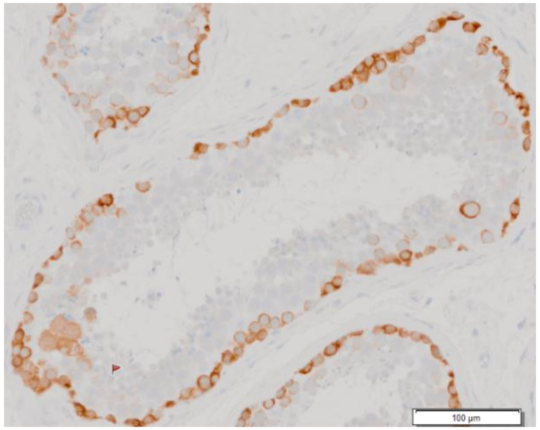

Clone no. 1-1_C44

A

Clone no.	Location	Slide 23	Slide 24	Slide 25	Slide 26	Slide 27	Slide 28	Slide 29	Slide 30	Slide 31	Slide 32	Slide 33	Slide 34	Slide 35	Slide 36	Slide 37	Slide 38	Slide 39	Sldie 40	Slide 41	Minimum number of cells
		MAGEA4	Ki67	H&E	MAGEA4	OCT2	MAGEA4	FGFR3	Ki67	MAGEA4	SSX	SAGE1	OCT2	MAGEA4	n.s	n.s	n.s	n.s	n.s	MAGEA4	
1-1_C44	Near Periphery	NA			0*<br>2 cells	0	0*<br>3 cells	0	0	1<br>10 cells	0	0	0	1<br>21 cells						1<br>10 cells	187
					80 µm																

B

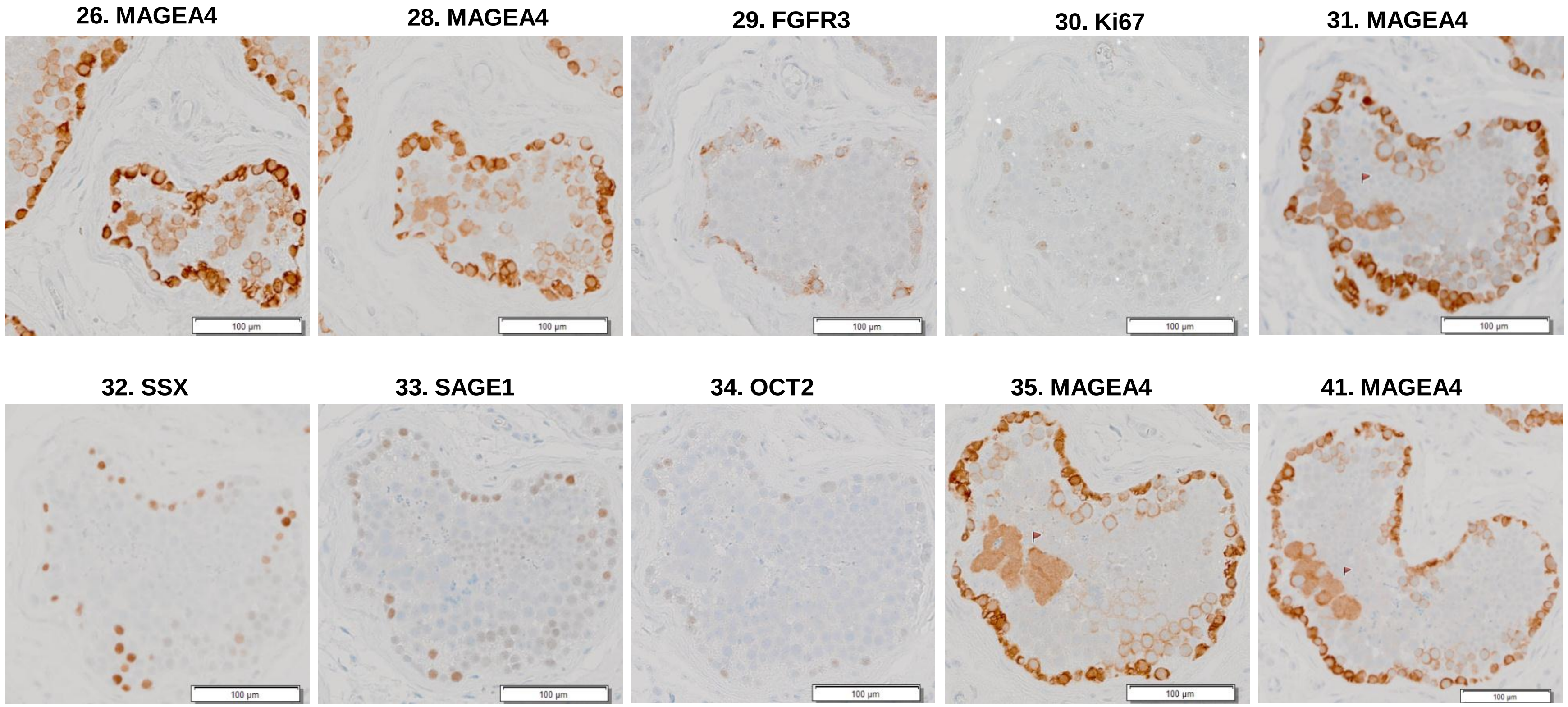

Clone no. 1-1_C45

A

Clone no.	Location	Slide 31	Slide 32	Slide 33	Slide 34	Slide 35	Slide 36	Slide 37	Slide 38	Slide 39	Slide 40	Slide 41	Minimum number of cells
		MAGEA4	SSX	SAGE1	OCT2	MAGEA4	n.s	n.s	n.s	n.s	n.s	MAGEA4	
1-1_C45	Centre	0	0	1<br>4 cells	0	1<br>12 cells						1<br>5 cells	72
				45 µm									

B

31. MAGEA4

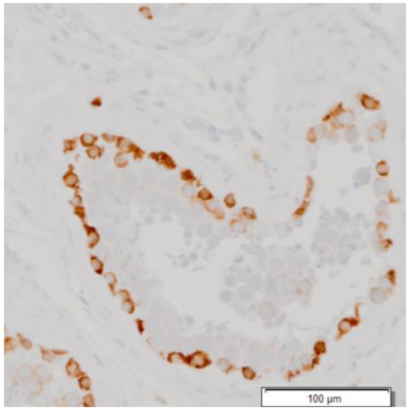

32. SSX

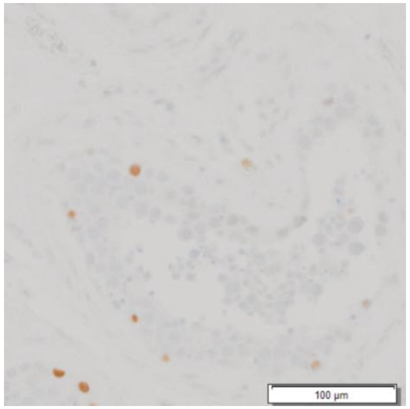

33. SAGE1

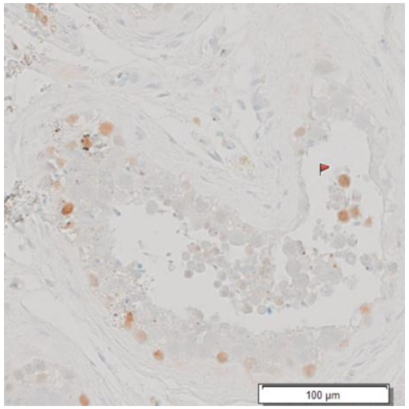

34. OCT2

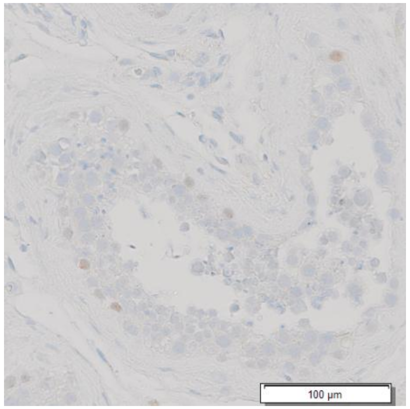

35. MAGEA4

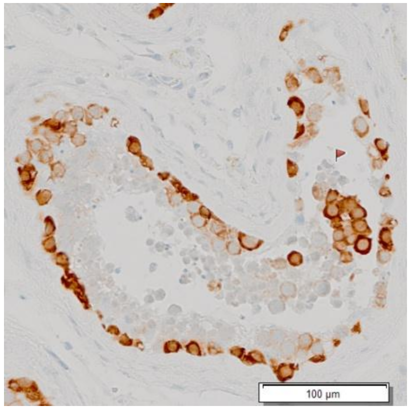

41. MAGEA4

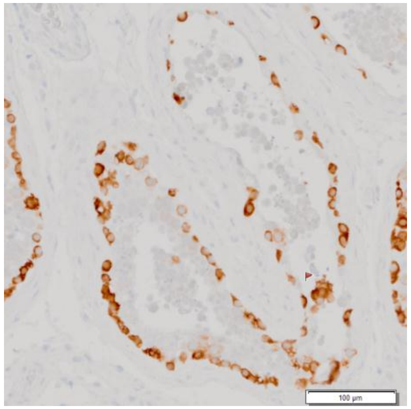

Clone no. 1-2_C1

A

Clone no.	Location	Slide 168	Slide 169	Slide 170	Slide 171	Slide 172	Slide 173	Slide 174	Slide 175	Slide 176	Slide 177	Slide 178	Slide 179	Slide 180	Slide 181	Minimum number of cells
		MAGEA4	FGFR3	Ki67	MAGEA4	SSX	SAGE1	OCT2	MAGEA4	n.s	OCT2	MAGEA4	FGFR3	Ki67	MAGEA4	
1-2_C1	Centre	1	0	0	1	0	0	0	1			0*			0	180
		20 cells			22 cells				15 cells			5 cells				
		55 µm														

B

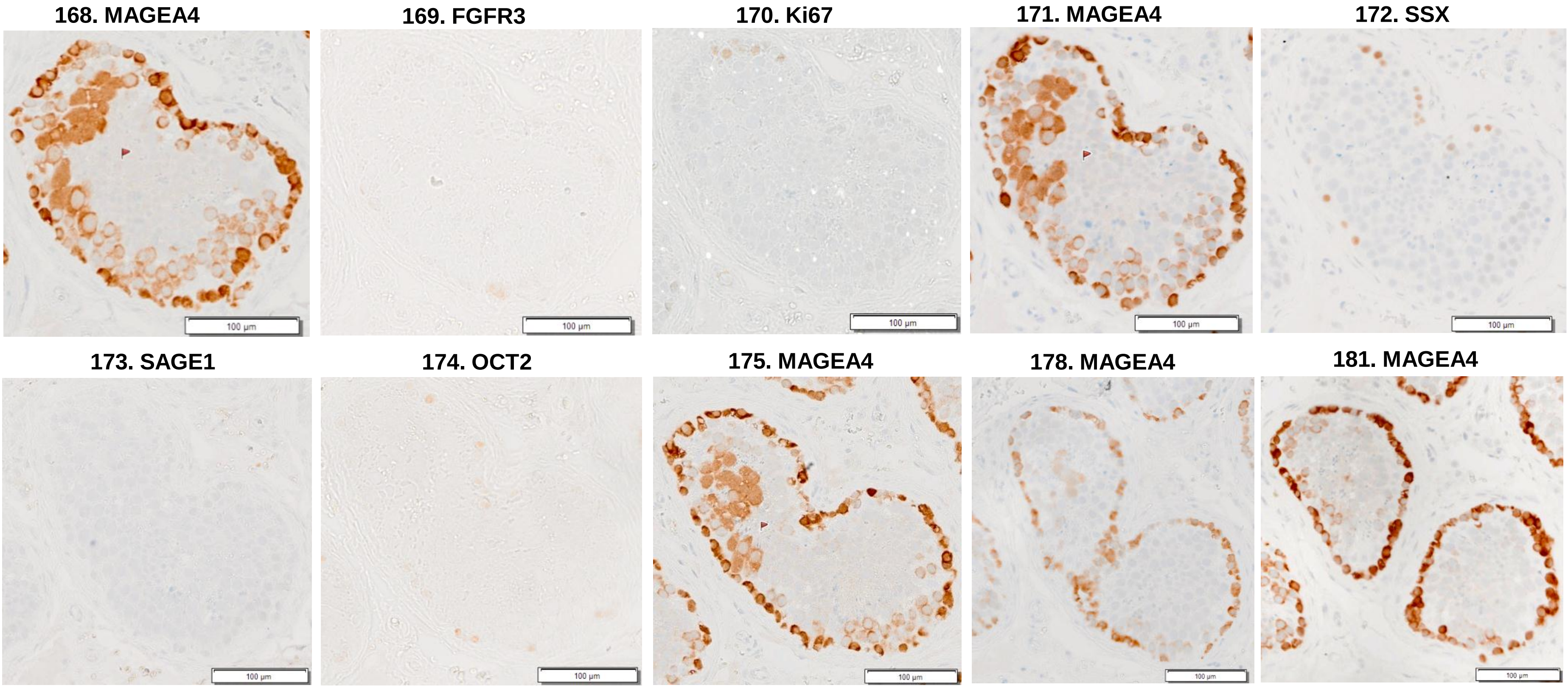

Clone no. 1-2_C2

A

Clone no.	Location	Slide 168	Slide 169	Slide 170	Slide 171	Slide 172	Slide 173	Slide 174	Slide 175	Slide 176	Slide 177	Slide 178	Slide 179	Slide 180	Slide 181	Minimum number of cells
		MAGEA4	FGFR3	Ki67	MAGEA4	SSX	SAGE1	OCT2	MAGEA4	n.s	OCT2	MAGEA4	FGFR3	Ki67	MAGEA4	
1-2_C2	Near Periphery	1<br>9 cells	0	NA	1<br>6 cells	0	0	0	1<br>3 cells			1<br>4 cells			0	58
		55 µm														

B

168. MAGEA4

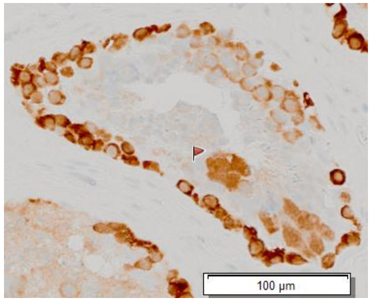

169. FGFR3

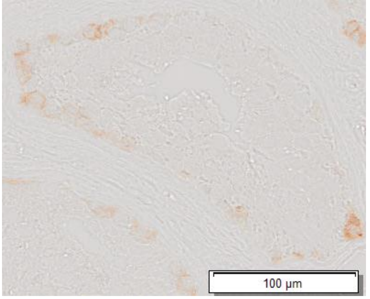

171. MAGEA4

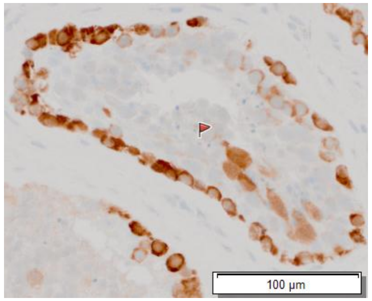

172. SSX

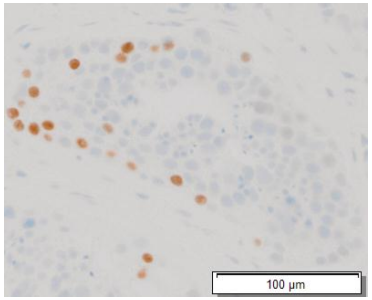

173. SAGE1

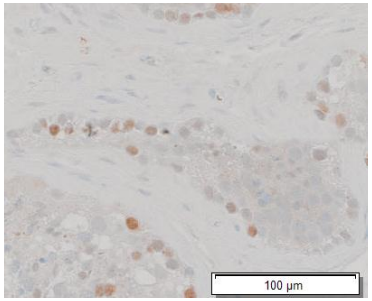

174. OCT2

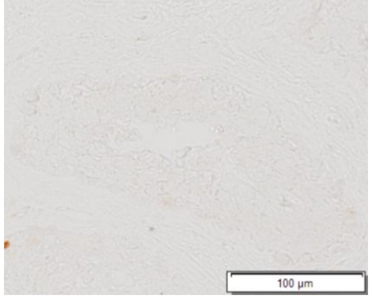

175. MAGEA4

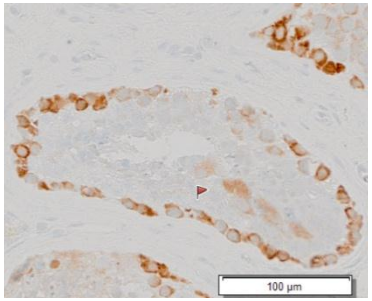

178. MAGEA4

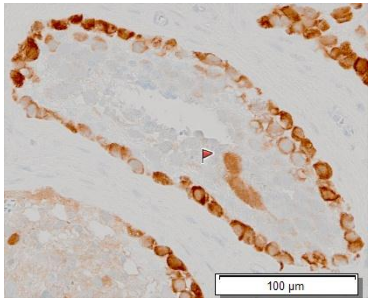

Clone no. 1-2_C3

A

Clone no.	Location	Slide 168	Slide 169	Slide 170	Slide 171	Slide 172	Slide 173	Slide 174	Slide 175	Slide 176	Slide 177	Slide 178	Slide 179	Slide 180	Slide 181	Slide 182	Slide 183	Slide 184	Slide 185	Minimum number of cells
		MAGEA4	FGFR3	Ki67	MAGEA4	SSX	SAGE1	OCT2	MAGEA4	n.s	OCT2	MAGEA4	FGFR3	Ki67	MAGEA4	SSX	FGFR3	n.s	MAGEA4	
1-2_C3	Centre	1	0	0	1	0	0	0	1			1			0*				0	87
		5 cells			7 cells				6 cells			7 cells			5 cells					
		70 µm																		

B

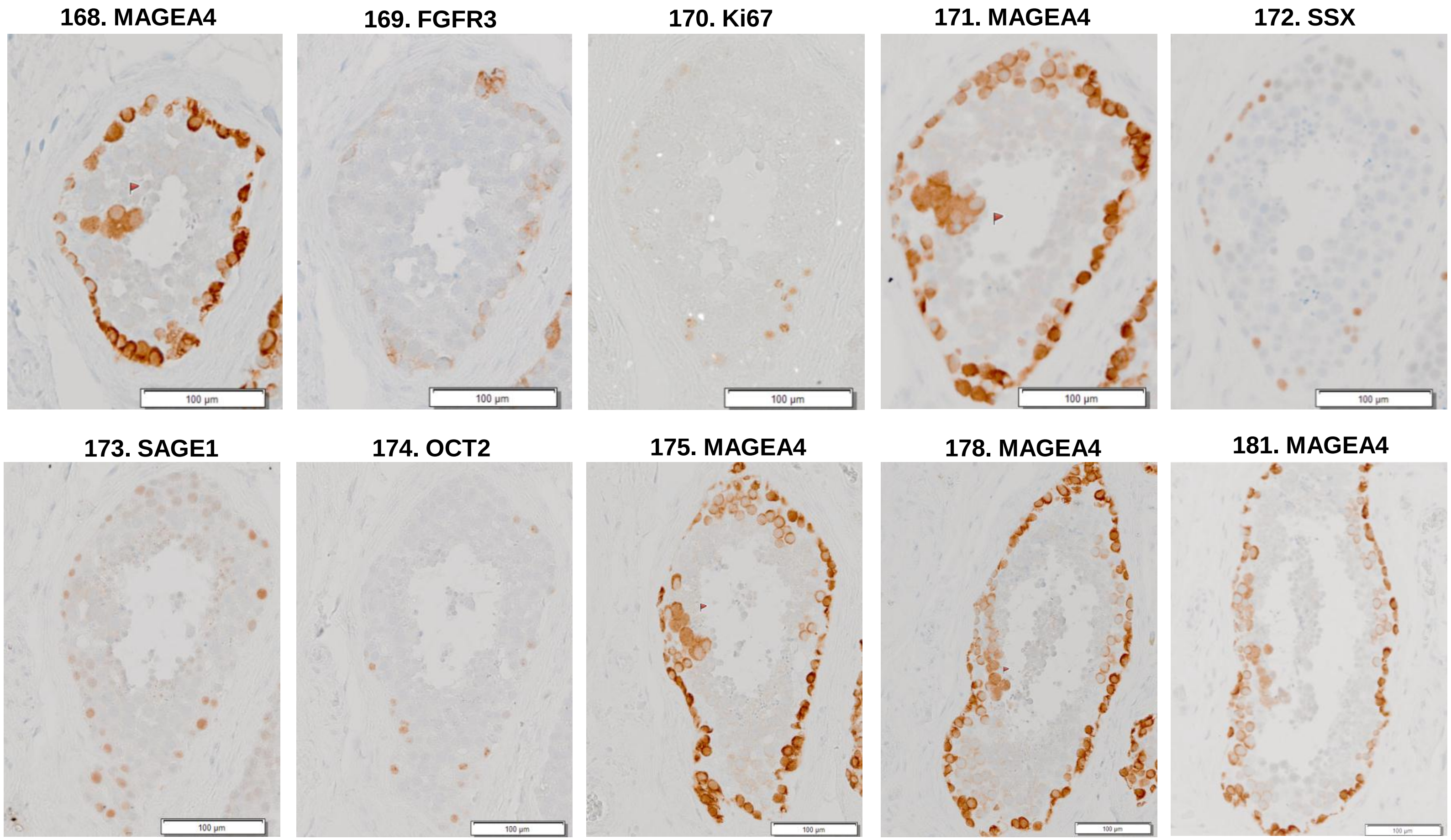

Clone no. 1-2_C4

A

Clone no.	Location	Slide 168	Slide 169	Slide 170	Slide 171	Slide 172	Slide 173	Slide 174	Slide 175	Slide 176	Slide 177	Slide 178	Minimum number of cells
		MAGEA4	FGFR3	Ki67	MAGEA4	SSX	SAGE1	OCT2	MAGEA4	n.s	OCT2	MAGEA4	
1-2_C4	Periphery	1<br>5 cells	0	0	1<br>5 cells	0	0	0	1<br>9 cells			0	50
		40 µm											

B

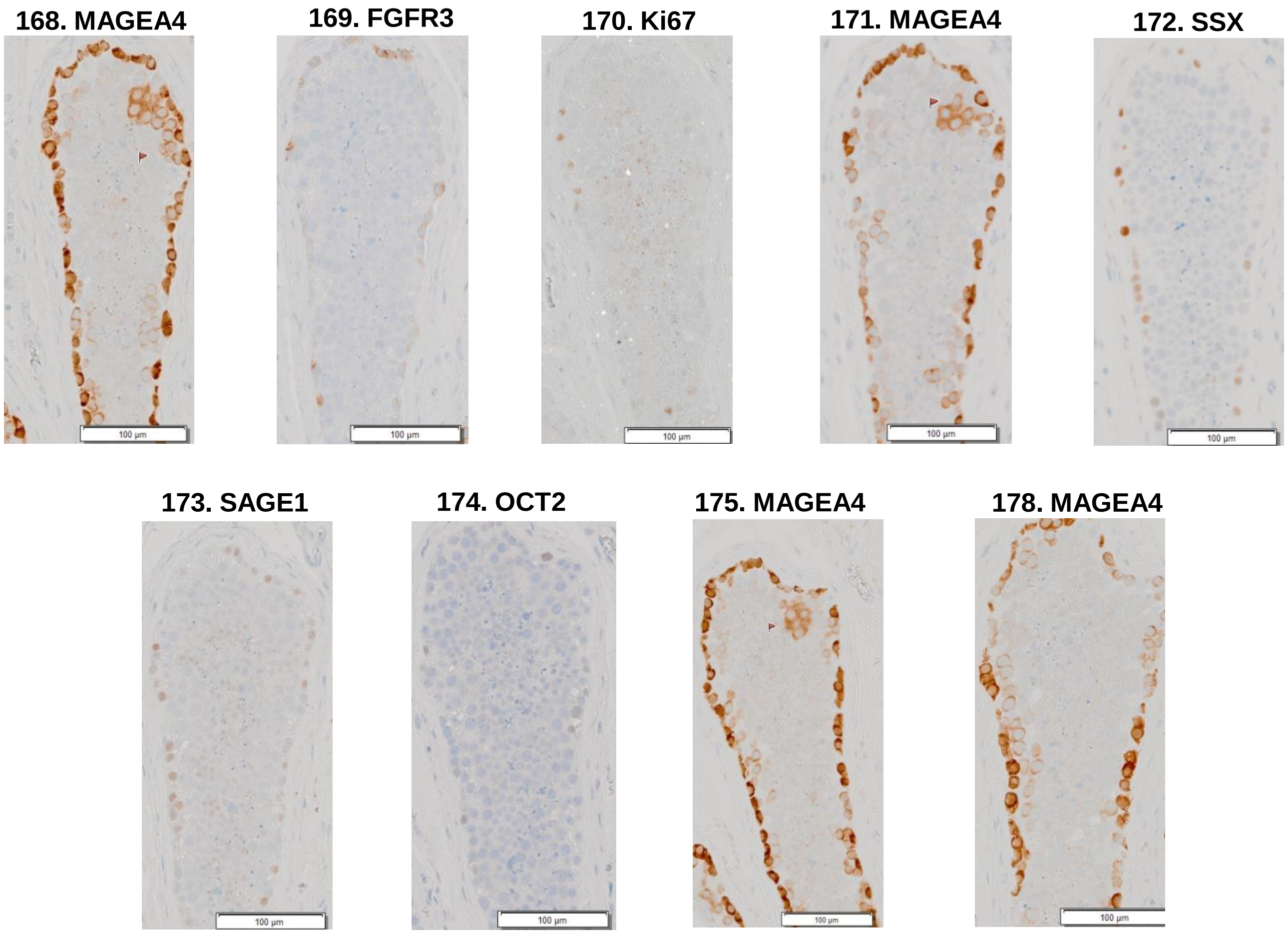

Clone no. 1-2_C5

A

Clone no.	Location	Slide 168	Slide 169	Slide 170	Slide 171	Slide 172	Slide 173	Slide 174	Slide 175	Slide 176	Slide 177	Slide 178	Slide 179	Slide 180	Slide 181	Minimum number of cells
		MAGEA4	FGFR3	Ki67	MAGEA4	SSX	SAGE1	OCT2	MAGEA4	n.s	OCT2	MAGEA4	FGFR3	Ki67	MAGEA4	
1-2_C5	Near Periphery	1<br>3 cells	0	0	1<br>4 cells	0	0	0	1<br>5 cells			1<br>3 cells			0	44
		55 µm														

B

168. MAGEA4

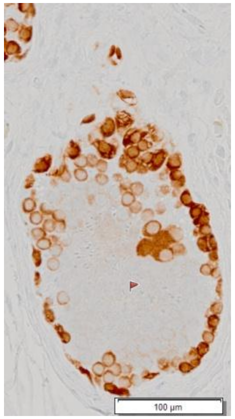

169. FGFR3

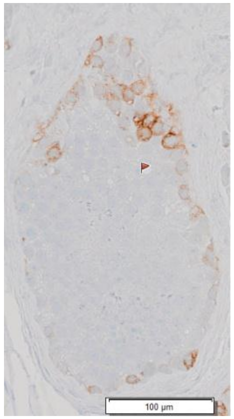

170. Ki67

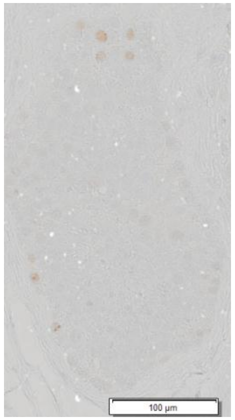

171. MAGEA4

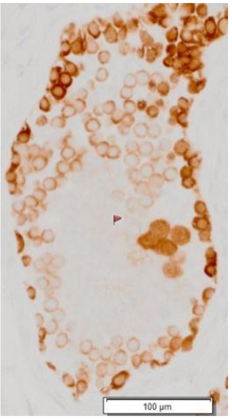

172. SSX

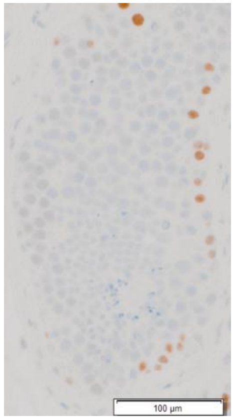

173. SAGE1

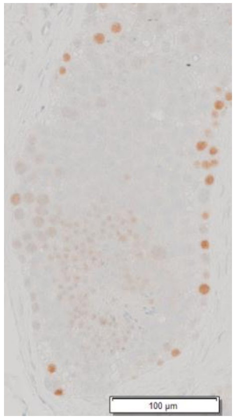

174. OCT2

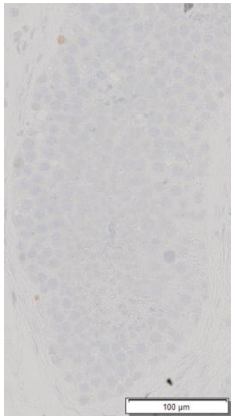

175. MAGEA4

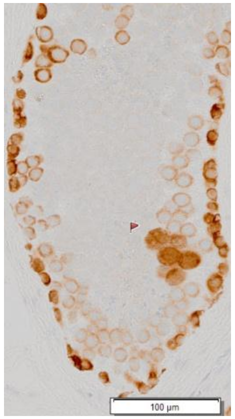

178. MAGEA4

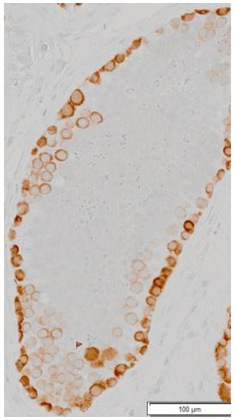

181. MAGEA4

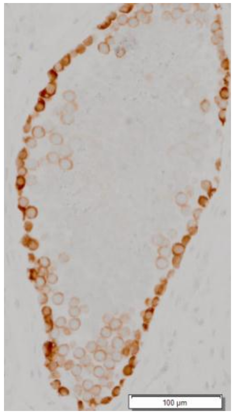

Clone no. 1-2_C6

A

Clone no.	Location	Slide 168	Slide 169	Slide 170	Slide 171	Slide 172	Slide 173	Slide 174	Slide 175	Slide 176	Slide 177	Slide 178	Slide 179	Slide 180	Slide 181	Minimum number of cells
		MAGEA4	FGFR3	Ki67	MAGEA4	SSX	SAGE1	OCT2	MAGEA4	n.s	OCT2	MAGEA4	FGFR3	Ki67	MAGEA4	
1-2_C6	Near Periphery	0*<br>1 cell	0	0	1<br>8 cells	0	0	0	1<br>7 cells			1<br>5 cells			0	65
		55 µm														

B

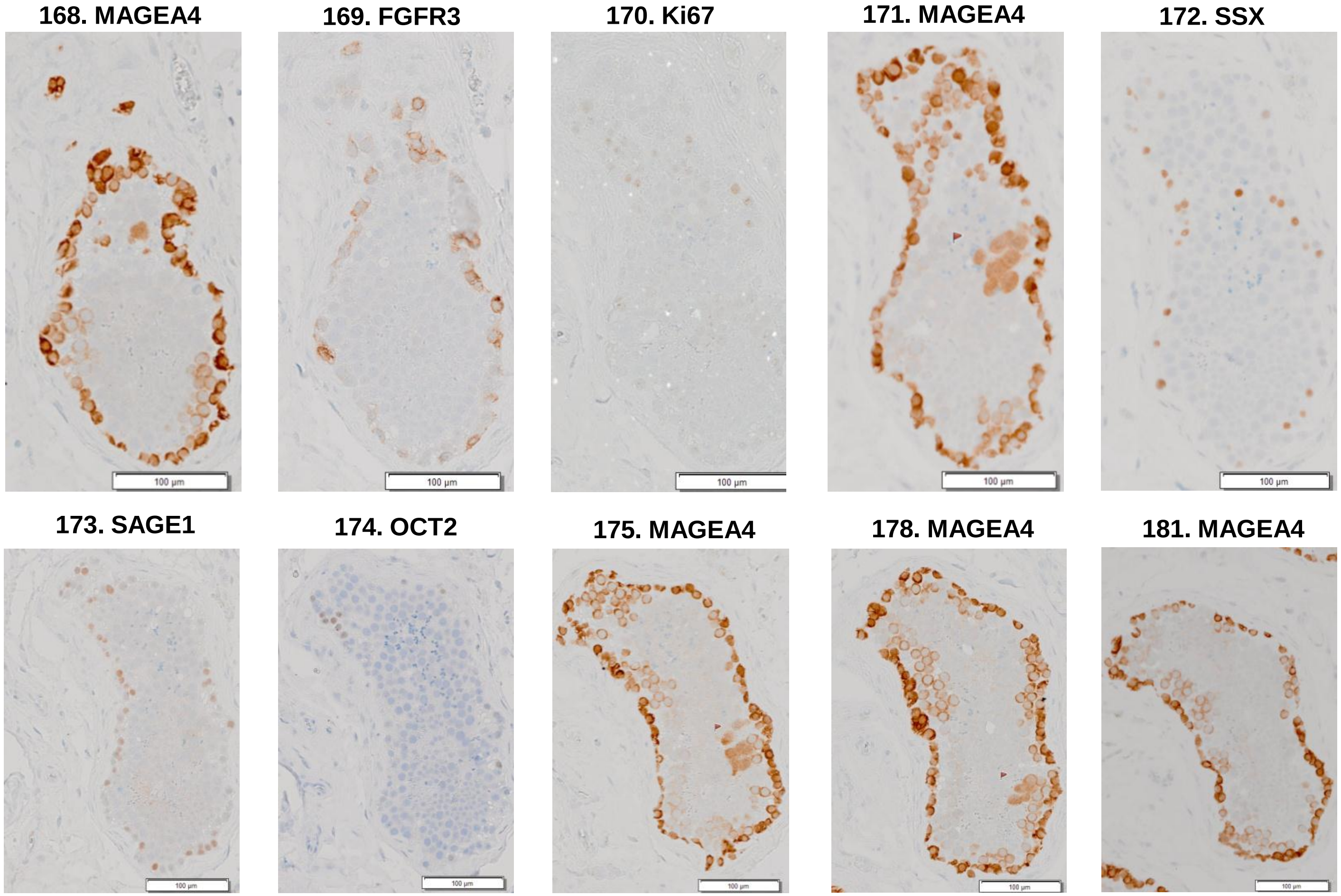

Clone no. 1-2_C7

A

Clone no.	Location	Slide 168	Slide 169	Slide 170	Slide 171	Slide 172	Slide 173	Slide 174	Slide 175	Slide 176	Slide 177	Slide 178	Slide 179	Slide 180	Slide 181	Minimum number of cells
		MAGEA4	FGFR3	Ki67	MAGEA4	SSX	SAGE1	OCT2	MAGEA4	n.s	OCT2	MAGEA4	FGFR3	Ki67	MAGEA4	
1-2_C7	Near Periphery	1<br>13 cells	0	0	1<br>10 cells	0	0	0	1<br>19 cells			1<br>6 cells			1<br>3 cells	152
		70 µm														

B

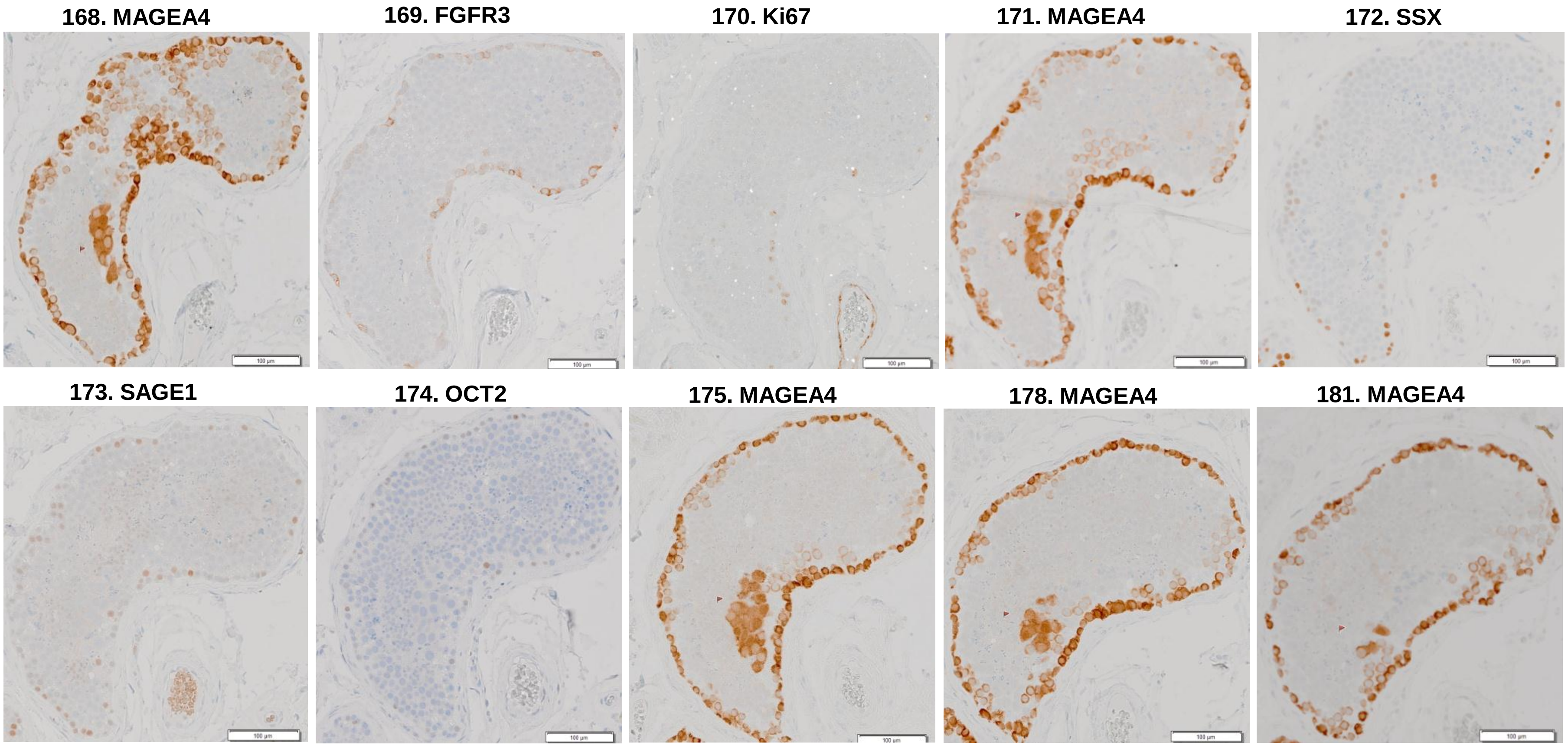

Clone no. 1-2_C8

A

Clone no.	Location	Slide 168	Slide 169	Slide 170	Slide 171	Slide 172	Slide 173	Slide 174	Slide 175	Slide 176	Slide 177	Slide 178	Slide 179	Slide 180	Slide 181	Minimum number of cells
		MAGEA4	FGFR3	Ki67	MAGEA4	SSX	SAGE1	OCT2	MAGEA4	n.s	OCT2	MAGEA4	FGFR3	Ki67	MAGEA4	
1-2_C8	Near Periphery	0*<br>2 cells	0	0	1<br>6 cells	0	0	0	1<br>3 cells			0*<br>2 cells			0	40
		55 µm														

B

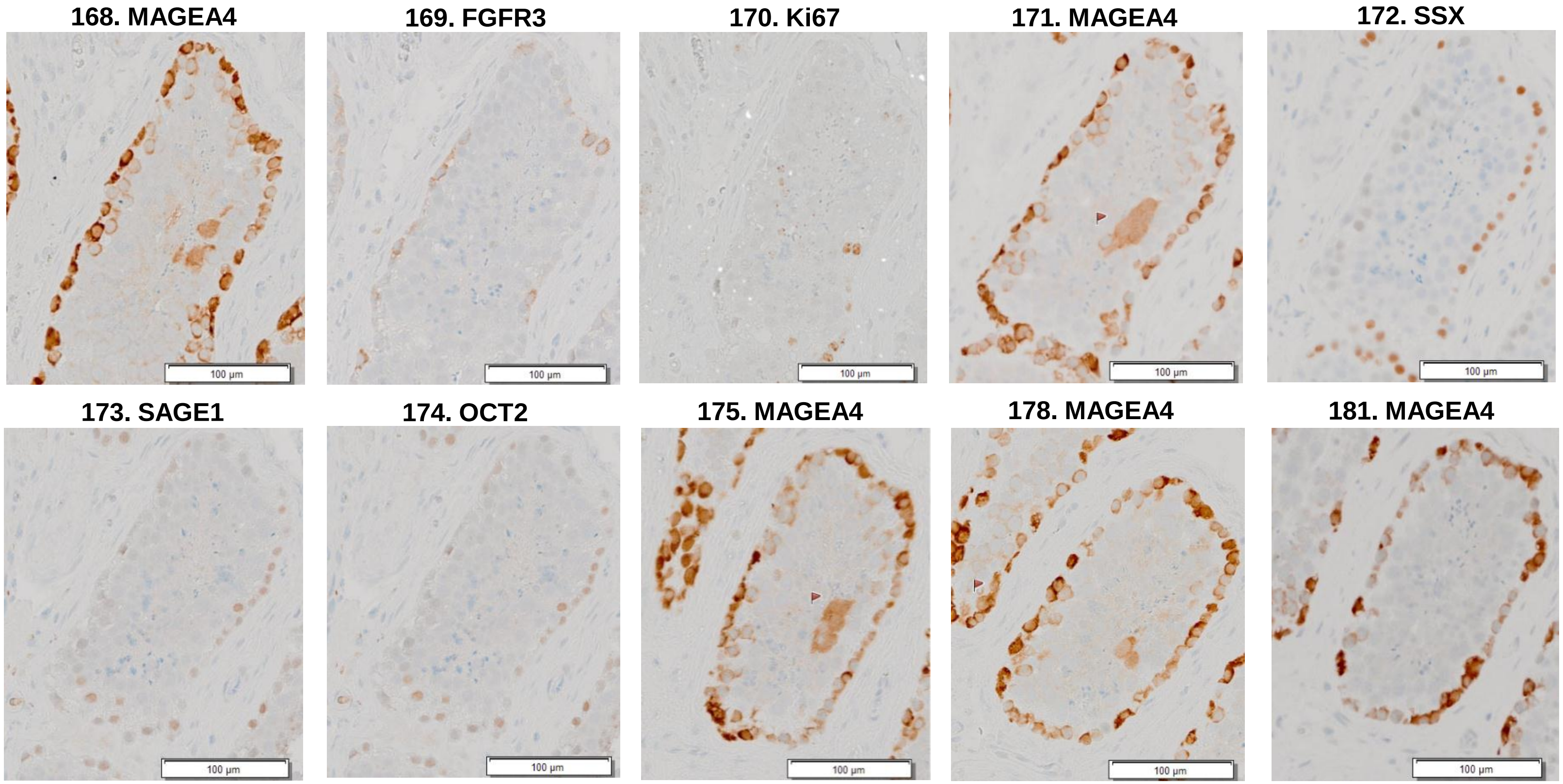

Clone no. 1-2_C9

A

Clone no.	Location	Slide 168	Slide 169	Slide 170	Slide 171	Slide 172	Slide 173	Slide 174	Slide 175	Slide 176	Slide 177	Slide 178	Minimum number of cells
		MAGEA4	FGFR3	Ki67	MAGEA4	SSX	SAGE1	OCT2	MAGEA4	n.s	OCT2	MAGEA4	
1-2_C9	Centre	0*<br>2 cells	0	0	1<br>4 cells	0	0	0	1<br>6 cells			1<br>7 cells	54
		55 µm											

B

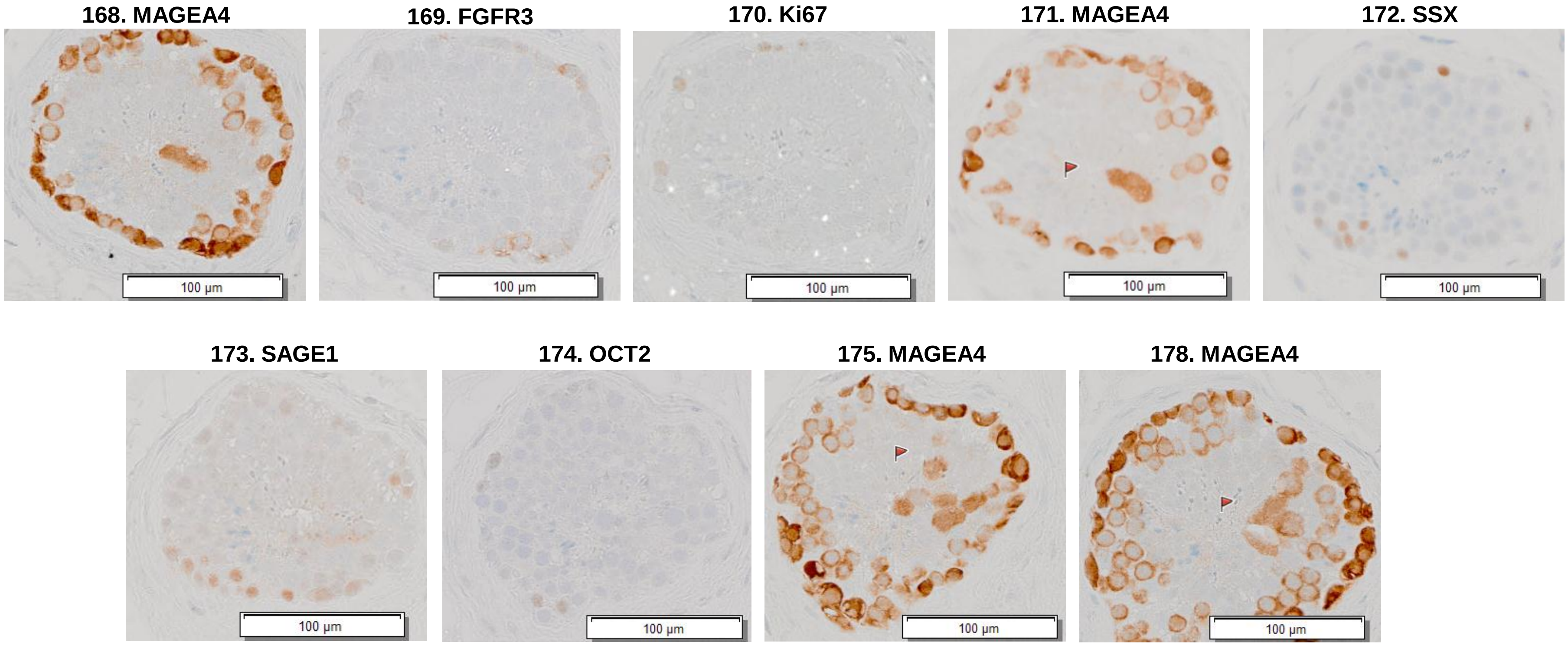

Clone no. 1-2_C10

A

Clone no.	Location	Slide 168	Slide 169	Slide 170	Slide 171	Slide 172	Slide 173	Slide 174	Slide 175	Slide 176	Slide 177	Slide 178	Slide 179	Slide 180	Slide 181	Slide 182	Slide 183	Slide 184	Slide 185	Minimum number of cells
		MAGEA4	FGFR3	Ki67	MAGEA4	SSX	SAGE1	OCT2	MAGEA4	n.s	OCT2	MAGEA4	FGFR3	Ki67	MAGEA4	SSX	FGFR3	n.s	MAGEA4	
1-2_C10	Centre	1<br>5 cells	0	0	1<br>9 cells	0	0	0	1<br>9 cells			1<br>14 cells			1<br>8 cells				0	131
		70 µm																		

B

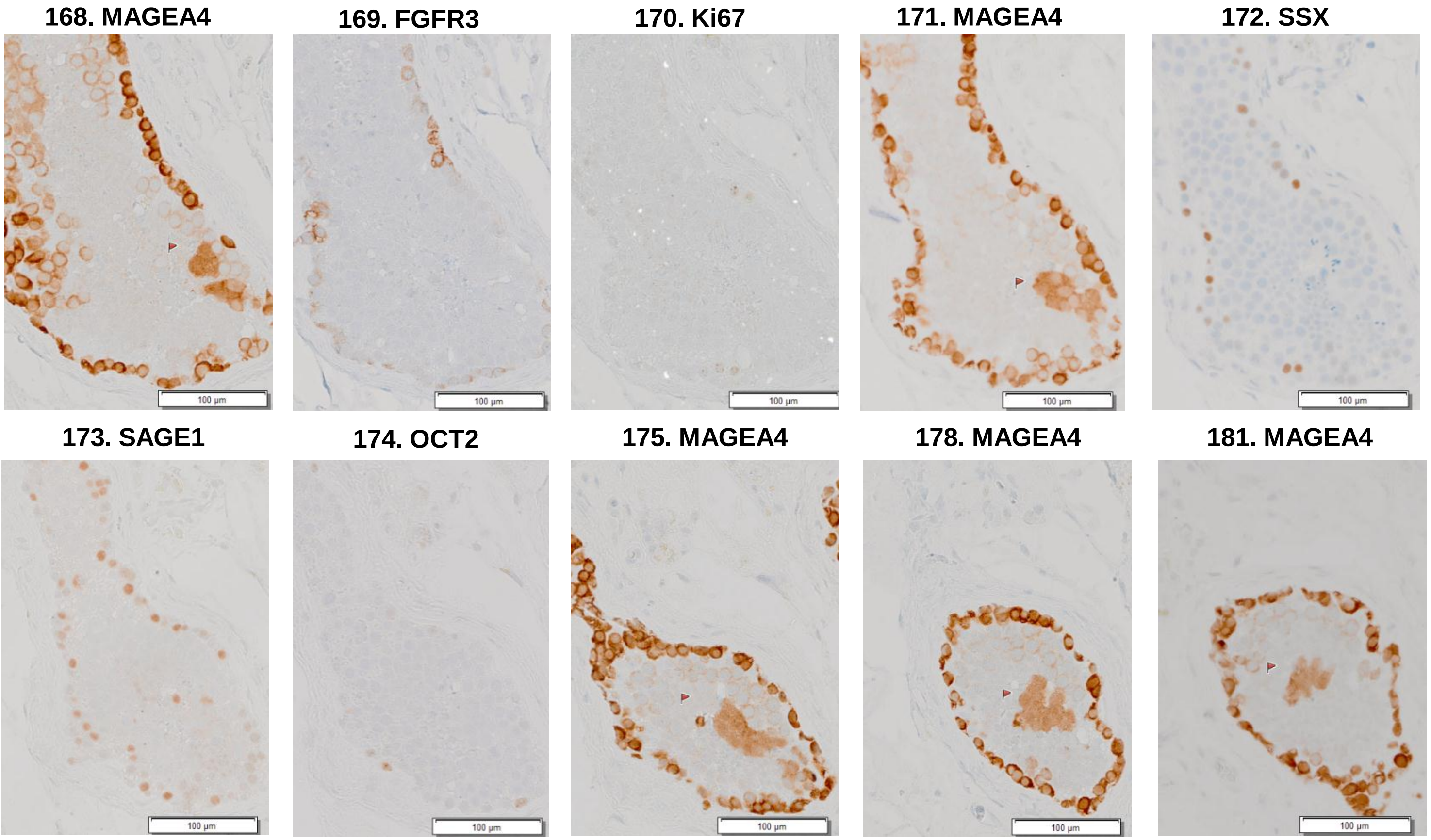

Clone no. 1-2_C11

A

Clone no.	Location	Slide 168	Slide 169	Slide 170	Slide 171	Slide 172	Slide 173	Slide 174	Slide 175	Slide 176	Slide 177	Slide 178	Minimum number of cells
		MAGEA4	FGFR3	Ki67	MAGEA4	SSX	SAGE1	OCT2	MAGEA4	n.s	OCT2	MAGEA4	
1-2_C11	Centre & periphery	0*<br>2 cells	0	0	1<br>11 cells	0	0	0	1<br>6 cells			NA	58
		40 µm											

B

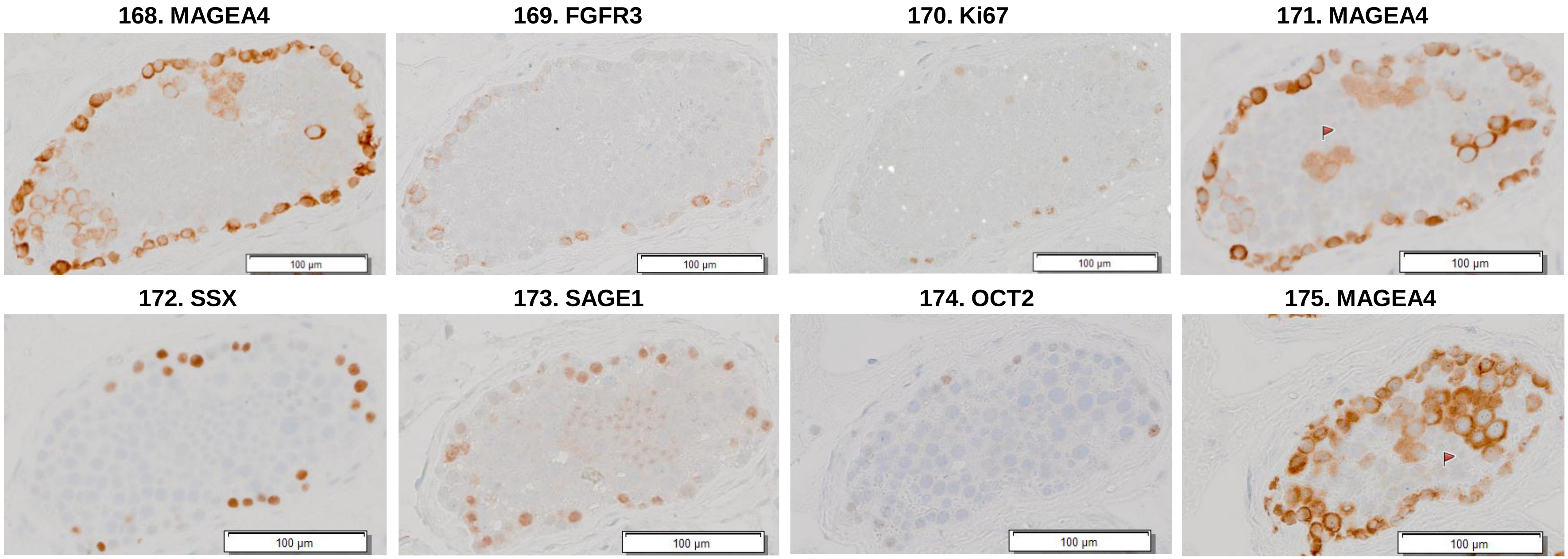

Clone no. 1-2_C12

A

Clone no.	Location	Slide 168	Slide 169	Slide 170	Slide 171	Slide 172	Slide 173	Slide 174	Slide 175	Slide 176	Slide 177	Slide 178	Minimum number of cells
		MAGEA4	FGFR3	Ki67	MAGEA4	SSX	SAGE1	OCT2	MAGEA4	n.s	OCT2	MAGEA4	
1-2_C12	Near Periphery	0*<br>1 cell	0	0	1<br>6 cells	0	0	0	1<br>7 cells			NA	41
		40 µm											

B

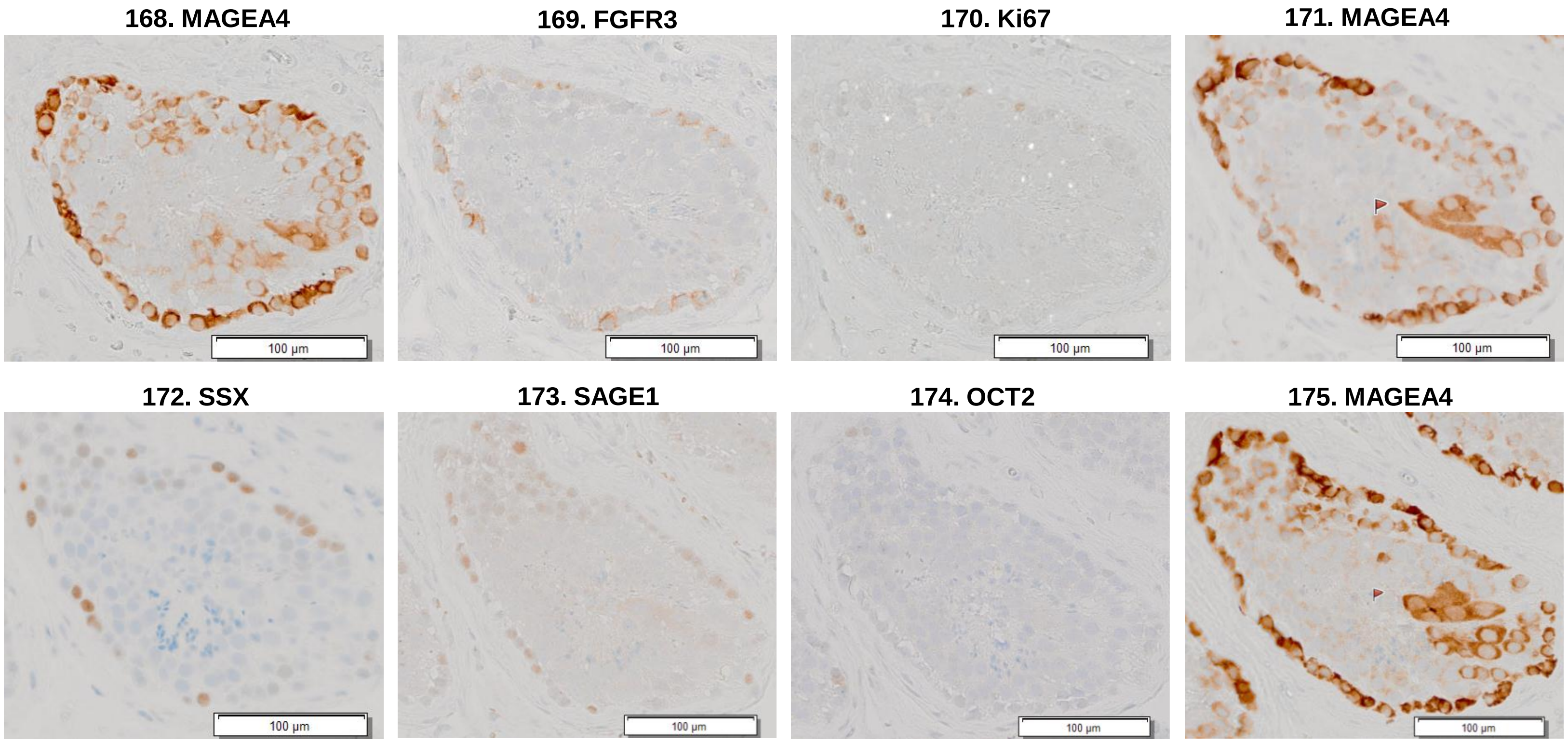

Clone no. 1-2_C13

A

Clone no.	Location	Slide 168	Slide 169	Slide 170	Slide 171	Slide 172	Slide 173	Slide 174	Slide 175	Slide 176	Slide 177	Slide 178	Slide 179	Slide 180	Slide 181	Minimum number of cells
		MAGEA4	FGFR3	Ki67	MAGEA4	SSX	SAGE1	OCT2	MAGEA4	n.s	OCT2	MAGEA4	FGFR3	Ki67	MAGEA4	
1-2_C13	Centre	1<br>8 cells	0	0	1<br>4 cells	0	0	0	1<br>7 cells			1<br>4 cells			0	63
		55 µm														

B

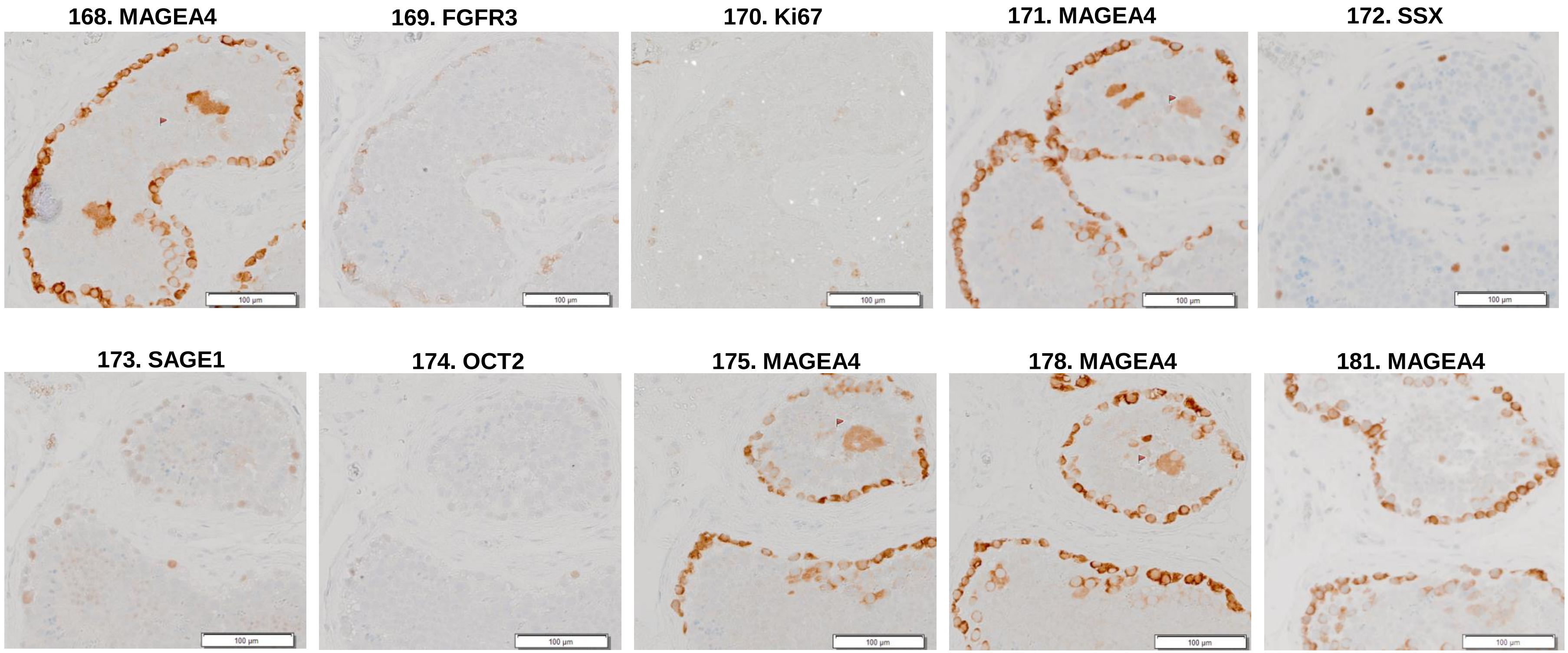

Clone no. 1-2_C14

A

Clone no.	Location	Slide 168	Slide 169	Slide 170	Slide 171	Slide 172	Slide 173	Slide 174	Slide 175	Minimum number of cells
		MAGEA4	FGFR3	Ki67	MAGEA4	SSX	SAGE1	OCT2	MAGEA4	
1-2_C14	Centre	0*<br>7 cells	0	0	1<br>12 cells	1<br>13 cells	0*<br>7 cells	0	NA	58
		30 µm								

B

168. MAGEA4

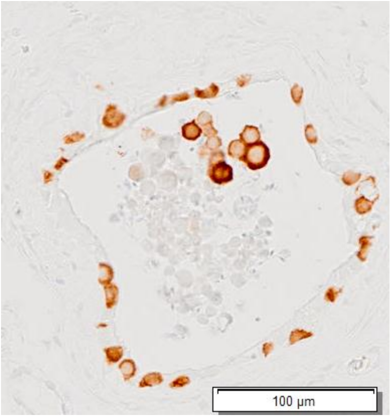

169. FGFR3

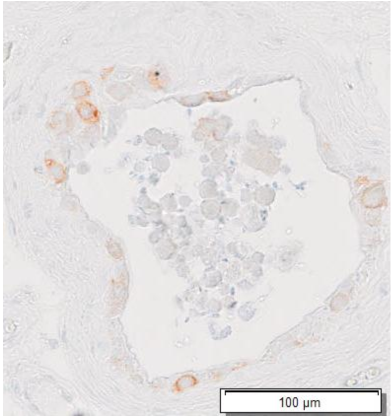

170. Ki67

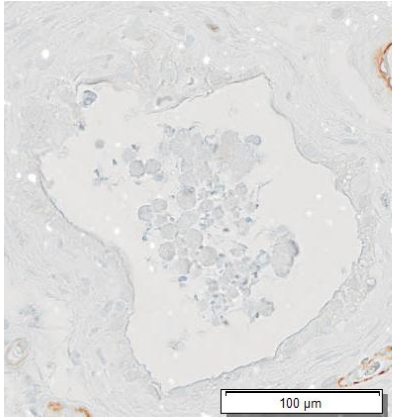

171. MAGEA4

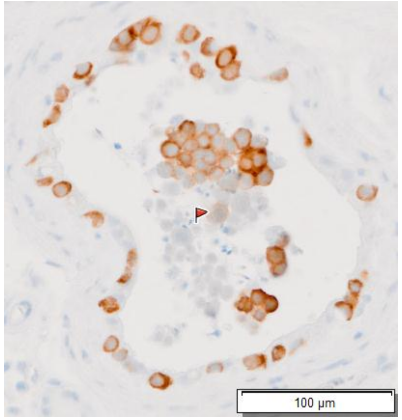

172. SSX

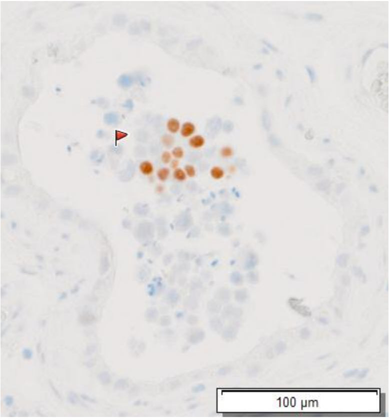

173. SAGE1

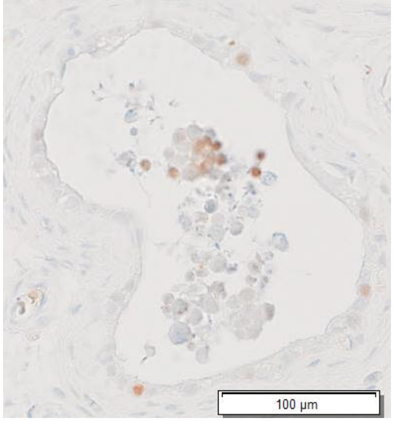

174. OCT2

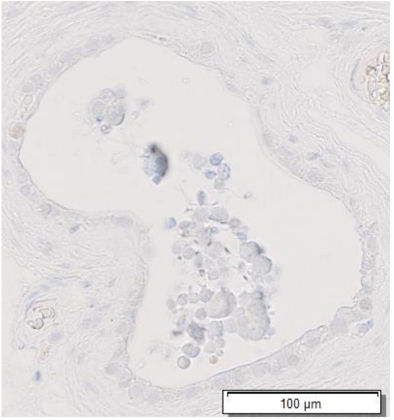

175. MAGEA4

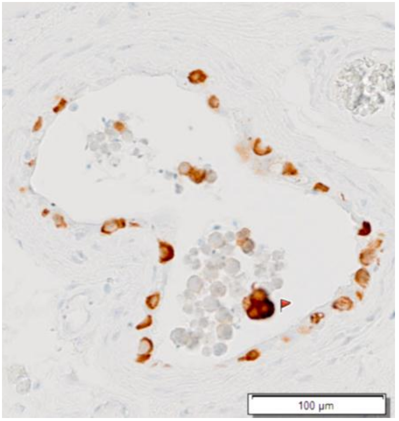

Clone no. 1-2_C15

A

Clone no.	Location	Slide 168	Slide 169	Slide 170	Slide 171	Slide 172	Slide 173	Slide 174	Slide 175	Slide 176	Slide 177	Slide 178	Minimum number of cells
		MAGEA4	FGFR3	Ki67	MAGEA4	SSX	SAGE1	OCT2	MAGEA4	n.s	OCT2	MAGEA4	
1-2_C15	Centre	0* 2 cells	0	0	1 4 cells	0	0	0	1 4 cells			0* 1 cell	34
		55 µm											

B

168. MAGEA4

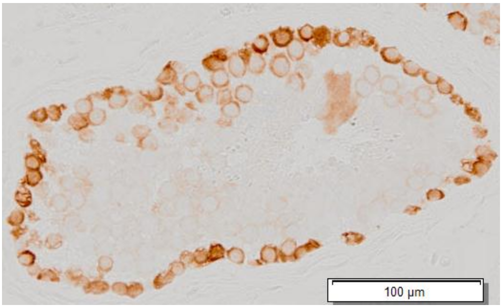

169. FGFR3

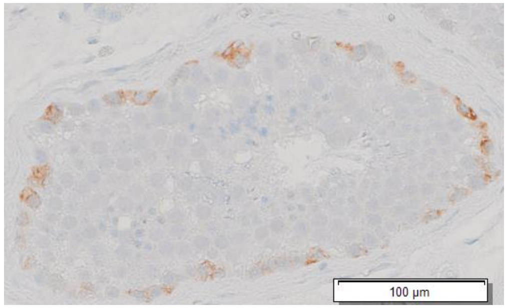

170. Ki67

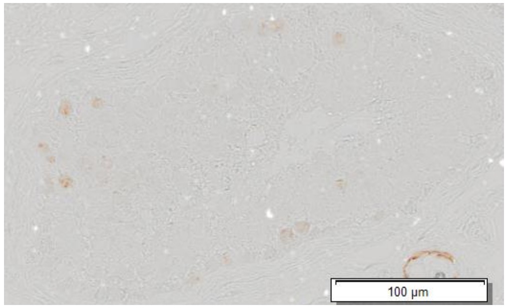

171. MAGEA4

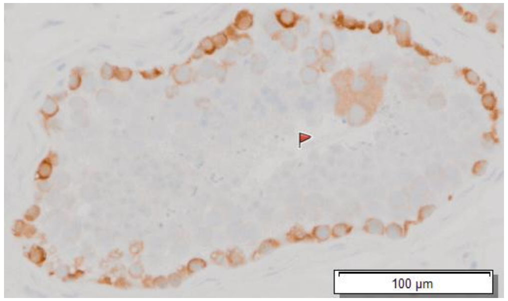

172. SSX

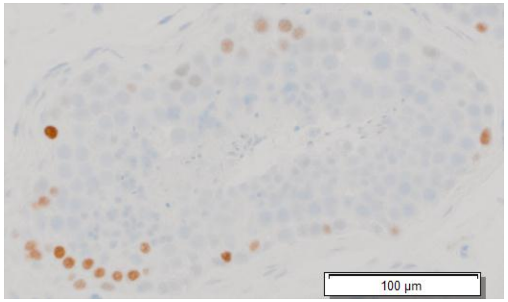

173. SAGE1

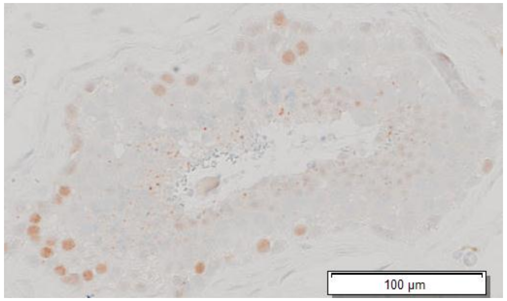

174. OCT2

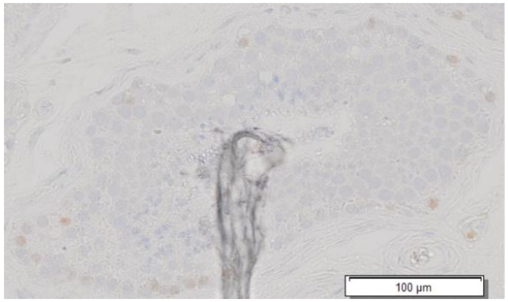

175. MAGEA4

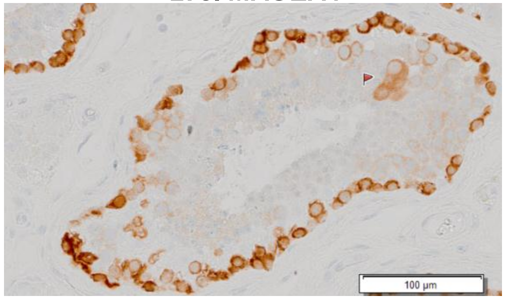

178. MAGEA4

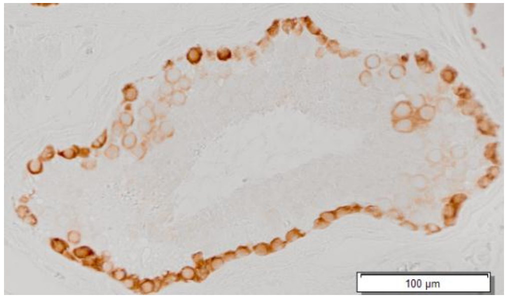

Clone no. 1-2_C16

A

Clone no.	Location	Slide 168	Slide 169	Slide 170	Slide 171	Slide 172	Slide 173	Slide 174	Slide 175	Slide 176	Slide 177	Slide 178	Slide 179	Slide 180	Slide 181	Minimum number of cells
		MAGEA4	FGFR3	Ki67	MAGEA4	SSX	SAGE1	OCT2	MAGEA4	n.s	OCT2	MAGEA4	FGFR3	Ki67	MAGEA4	
1-2_C16	Centre	0	0	0	1	0	0	0	1			0*			0	33
					3 cells				6 cells			2 cells				
					40 µm											

B

168. MAGEA4

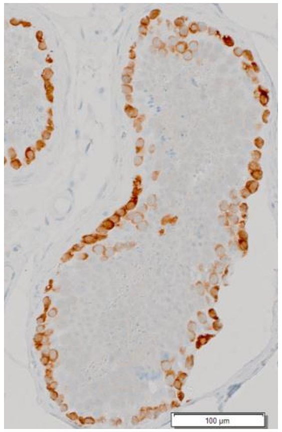

169. FGFR3

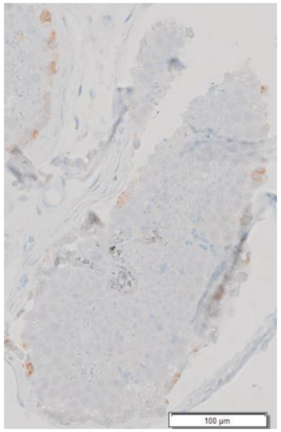

170. Ki67

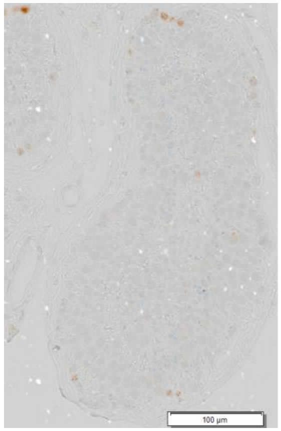

171. MAGEA4

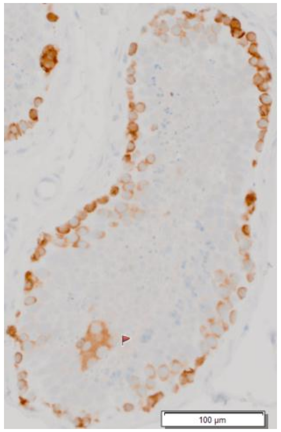

172. SSX

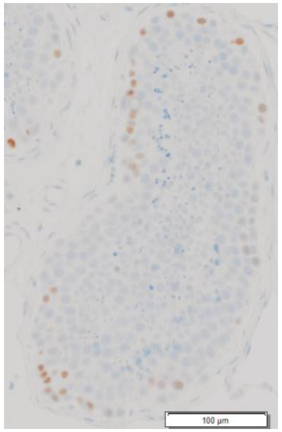

173. SAGE1

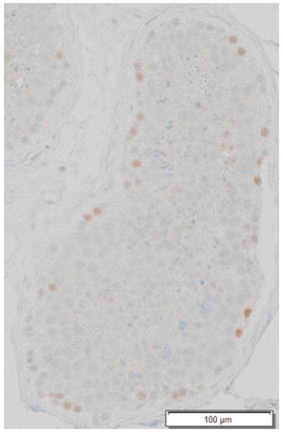

174. OCT2

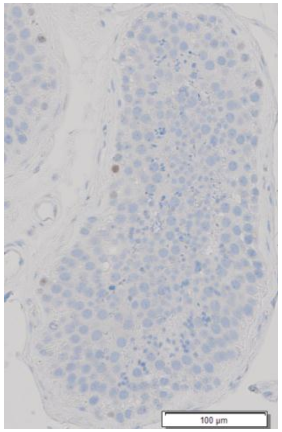

175. MAGEA4

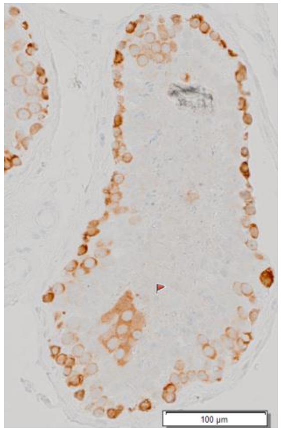

178. MAGEA4

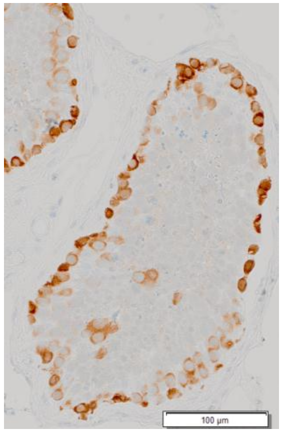

181. MAGEA4

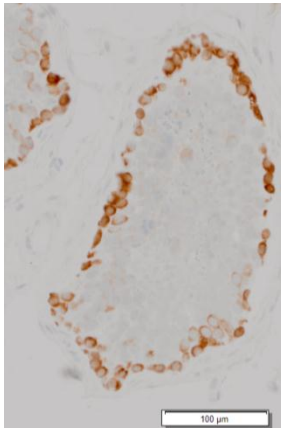

Clone no. 1-2_C17

A

Clone no.	Location	Slide 171	Slide 172	Slide 173	Slide 174	Slide 175	Slide 176	Slide 177	Slide 178	Slide 179	Slide 180	Slide 181	Slide 182	Slide 183	Slide 184	Slide 185	Minimum number of cells
		MAGEA4	SSX	SAGE1	OCT2	MAGEA4	n.s	OCT2	MAGEA4	FGFR3	Ki67	MAGEA4	SSX	FGFR3	n.s	MAGEA4	
1-2_C17	Centre	0	0	0	0	1		0	1	0	0	1	0			0	40
						8 cells			4 cells			6 cells					
						35 µm											

B

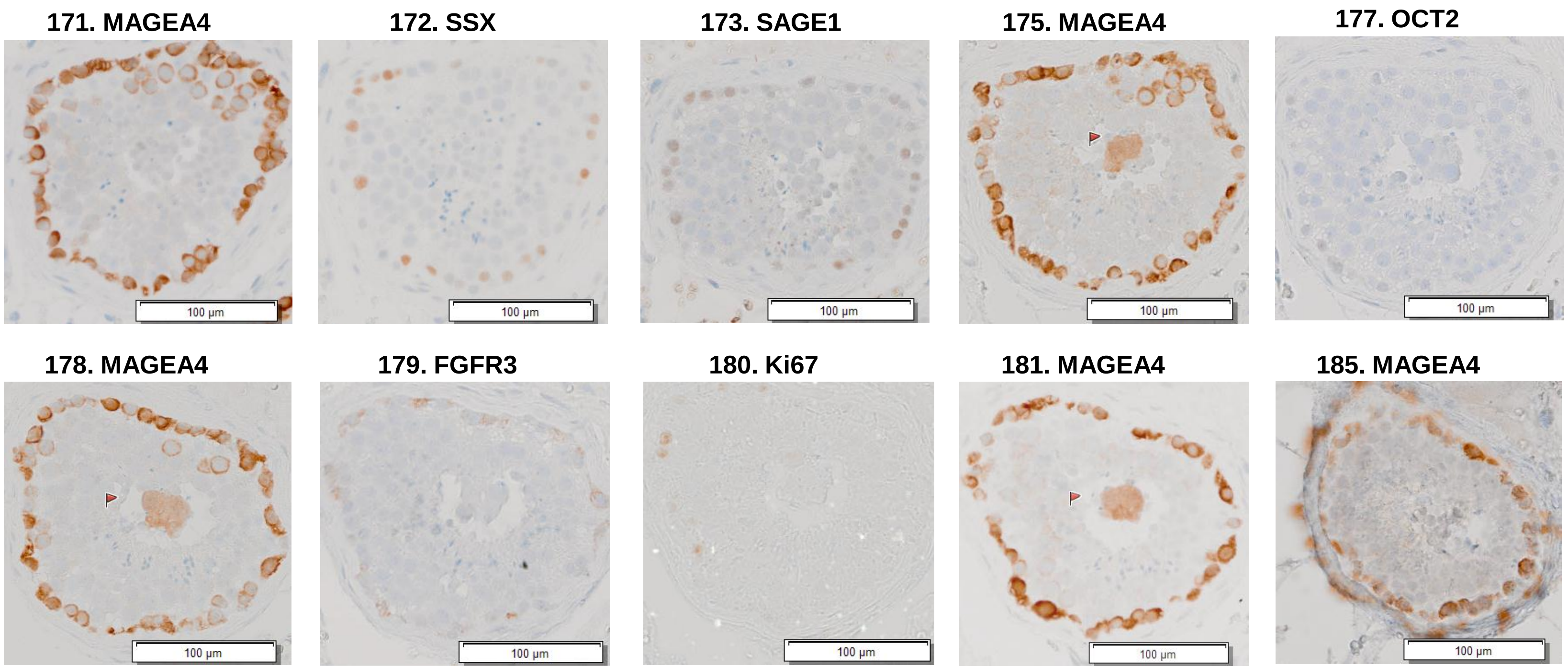

Clone no. 1-2_C18

A

Clone no.	Location	Slide 168	Slide 169	Slide 170	Slide 171	Slide 172	Slide 173	Slide 174	Slide 175	Slide 176	Slide 177	Slide 178	Slide 179	Slide 180	Slide 181	Minimum number of cells
		MAGEA4	FGFR3	Ki67	MAGEA4	SSX	SAGE1	OCT2	MAGEA4	n.s	OCT2	MAGEA4	FGFR3	Ki67	MAGEA4	
1-2_C18	Periphery	0*	NA	0	0*	1	1	0	1		0	1	0		0	112
		1 cell			7 cells	11 cells	11 cells		18 cells			11 cells				
		55 µm														

B

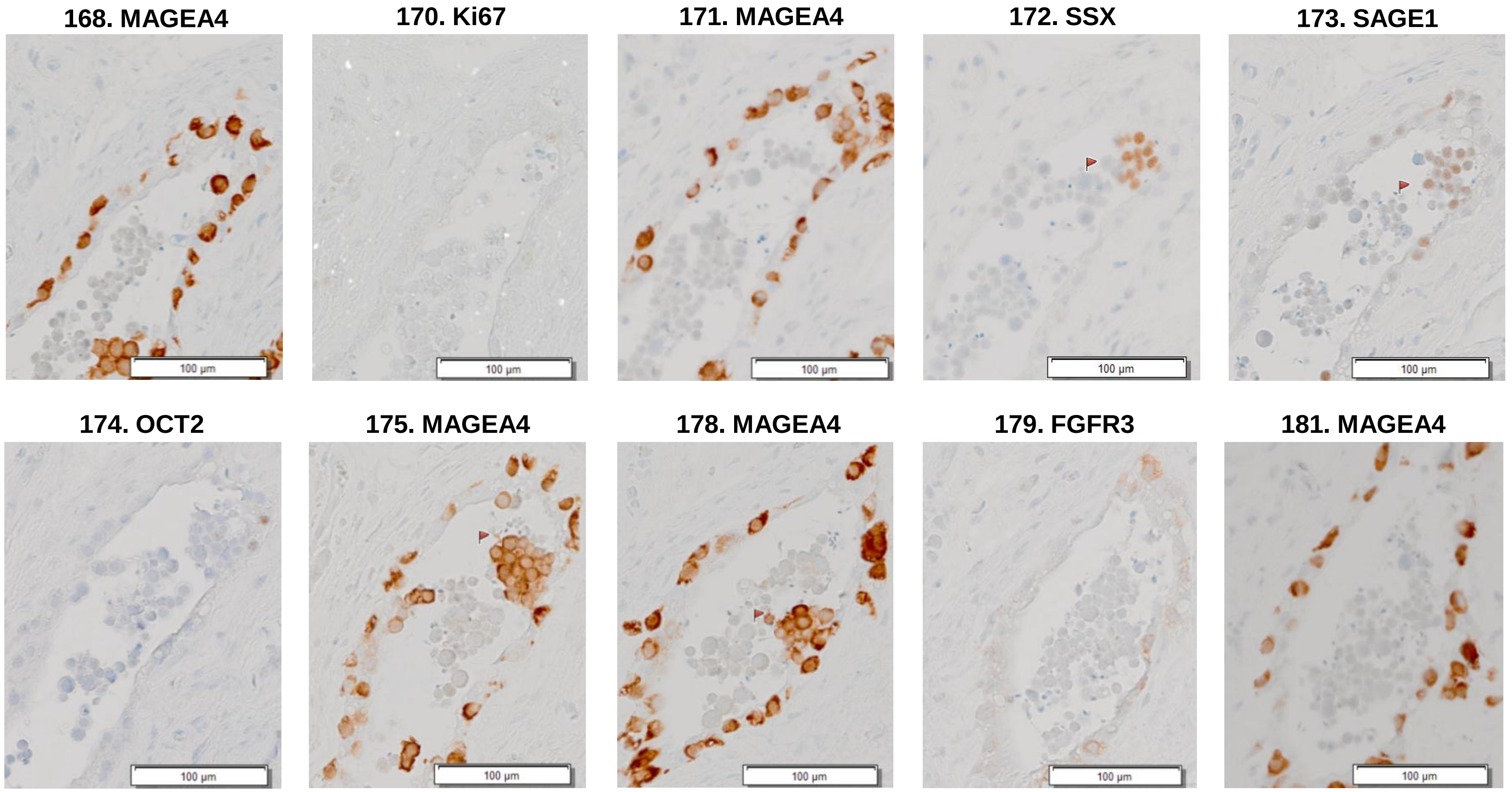

Clone no. 1-2_C19

A

Clone no.	Location	Slide 171	Slide 172	Slide 173	Slide 174	Slide 175	Slide 176	Slide 177	Slide 178	Slide 179	Slide 180	Slide 181	Minimum number of cells
		MAGEA4	SSX	SAGE1	OCT2	MAGEA4	n.s	OCT2	MAGEA4	FGFR3	Ki67	MAGEA4	
1-2_C19	Near Periphery	0	0	0	0	1<br>5 cells		0	1<br>13 cells	0	0	0*<br>2 cells	53
						35 µm							

B

171. MAGEA4

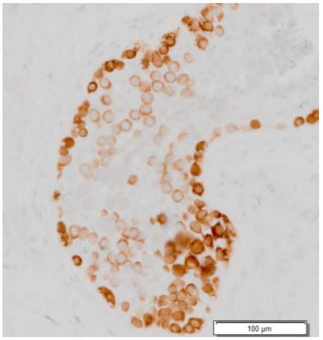

172. SSX

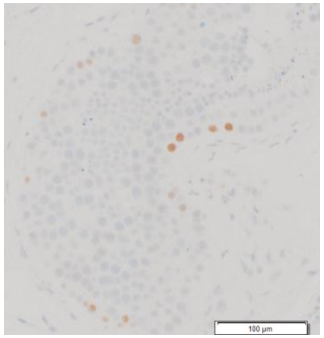

173. SAGE1

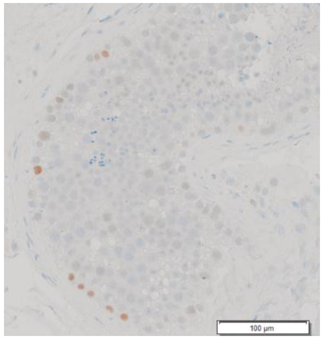

175. MAGEA4

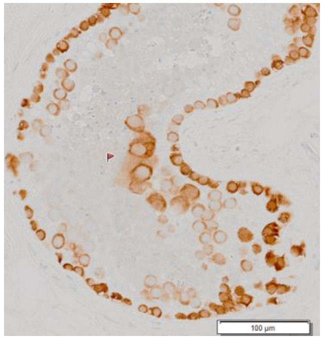

177. OCT2

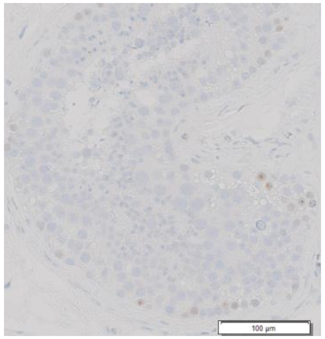

178. MAGEA4

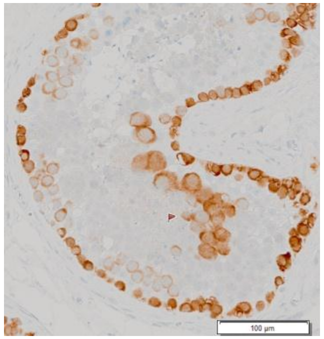

179. FGFR3

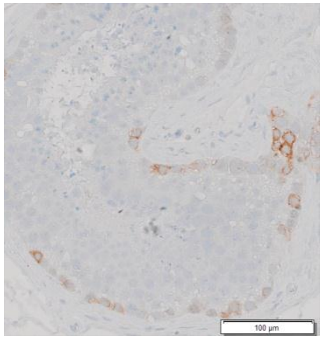

180. Ki67

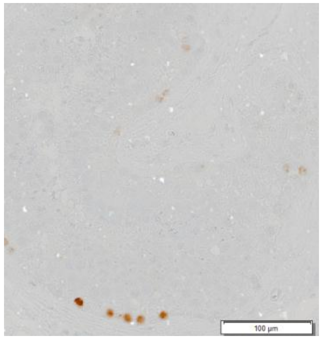

181. MAGEA4

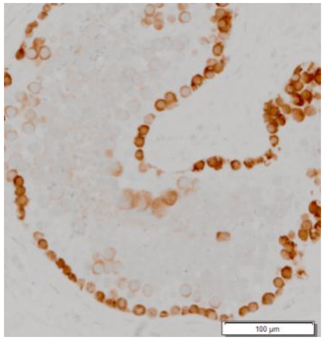

Clone no. 1-2_C20

A

Clone no.	Location	Slide 171	Slide 172	Slide 173	Slide 174	Slide 175	Slide 176	Slide 177	Slide 178	Slide 179	Slide 180	Slide 181	Slide 182	Slide 183	Slide 184	Slide 185	Slide 186	Slide 187	Slide 188	Slide 189	Slide 190	Slide 191	Minimum number of cells
		MAGEA4	SSX	SAGE1	OCT2	MAGEA4	n.s	OCT2	MAGEA4	FGFR3	Ki67	MAGEA4	SSX	FGFR3	n.s	MAGEA4	n.s	n.s	n.s	n.s	n.s	MAGEA4	
1-2_C20	Periphery	0* 3 cells	0	0	0	1 9 cells		0	1 5 cells	0	0	0* 5 cells				0* 6cells						NA	87
		75 µm																					

B

171. MAGEA4 | 172. SSX | 173. SAGE1 | 175. MAGEA4 | 177. OCT2

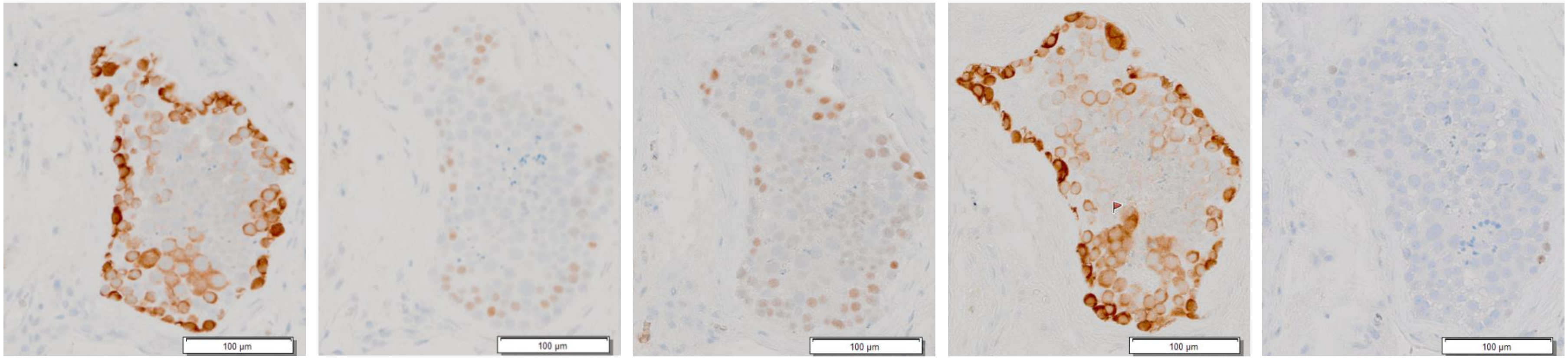

178. MAGEA4 | 179. FGFR3 | 180. Ki67 | 181. MAGEA4 | 185. MAGEA4

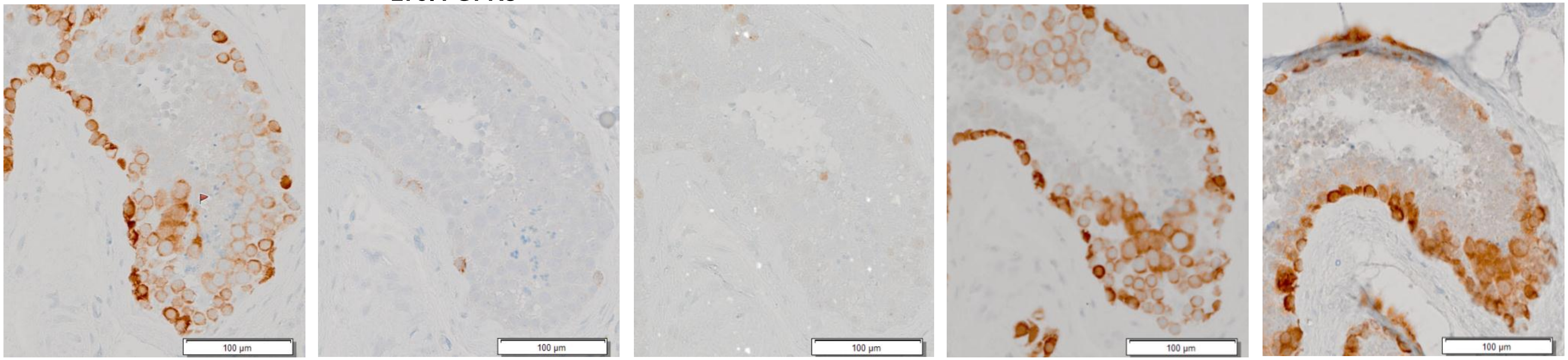

Clone no. 1-2_C21

A

Clone no.	Location	Slide 171	Slide 172	Slide 173	Slide 174	Slide 175	Slide 176	Slide 177	Slide 178	Slide 179	Slide 180	Slide 181	Slide 182	Slide 183	Slide 184	Slide 185	Slide 186	Slide 187	Slide 188	Slide 189	Slide 190	Slide 191	Minimum number of cells
		MAGEA4	SSX	SAGE1	OCT2	MAGEA4	n.s	OCT2	MAGEA4	FGFR3	Ki67	MAGEA4	SSX	FGFR3	n.s	MAGEA4	n.s	n.s	n.s	n.s	n.s	MAGEA4	
1-2_C21	Centre	0*	0	0	0	1		0	0*	0	0	1	0	0		0*						0	48
		1 cell				4 cells			1 cell			4 cells				3 cells							
		75 µm																					

B

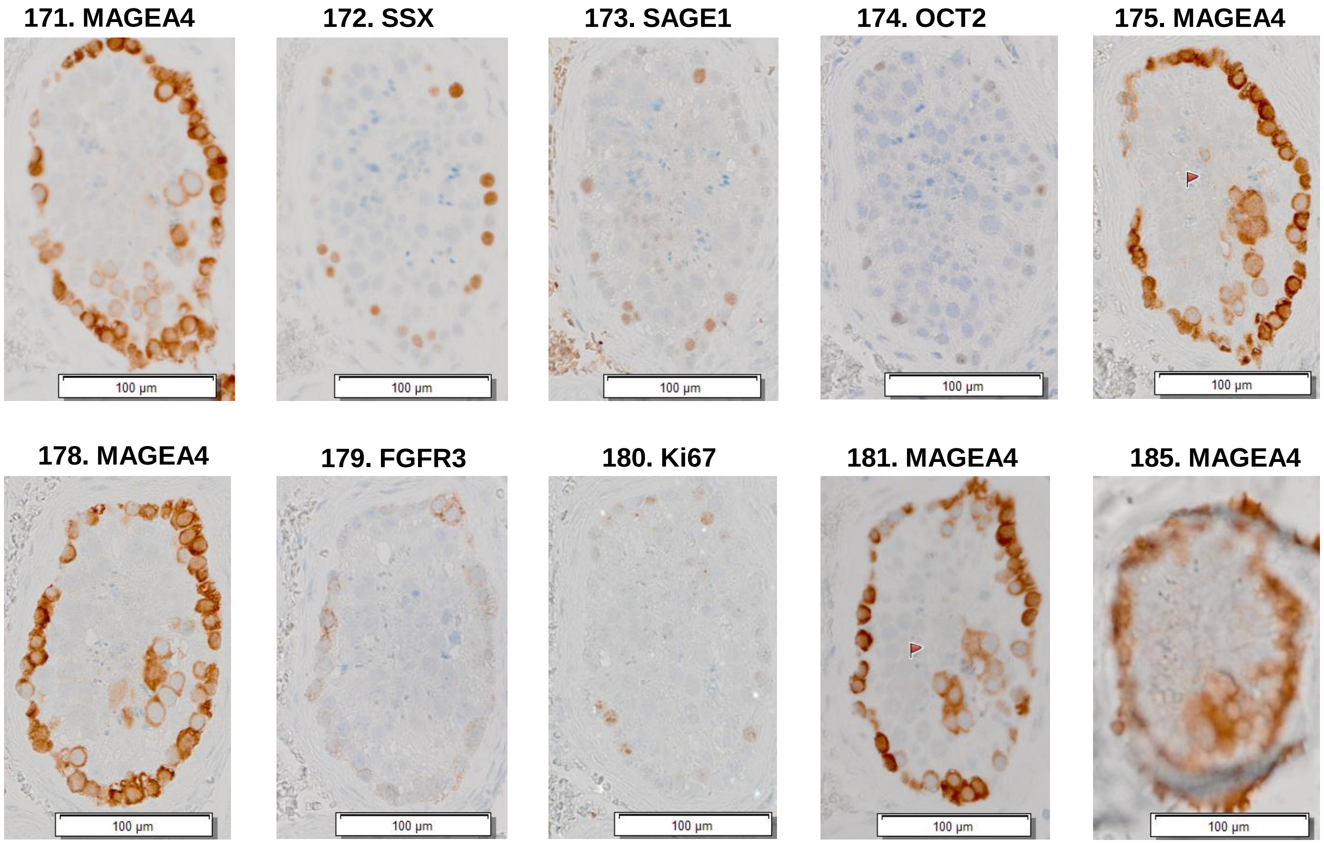

Clone no. 1-2_C22

A

Clone no.	Location	Slide 171	Slide 172	Slide 173	Slide 174	Slide 175	Slide 176	Slide 177	Slide 178	Slide 179	Slide 180	Slide 181	Slide 182	Slide 183	Slide 184	Slide 185	Minimum number of cells
		MAGEA4	SSX	SAGE1	OCT2	MAGEA4	n.s	OCT2	MAGEA4	FGFR3	Ki67	MAGEA4	SSX	FGFR3	n.s	MAGEA4	
1-2_C22	Centre	0	0	0	0	1<br>5 cells		0	1<br>11 cells	0	0	1<br>7 cells	0			NA	57
						35 µm											

B

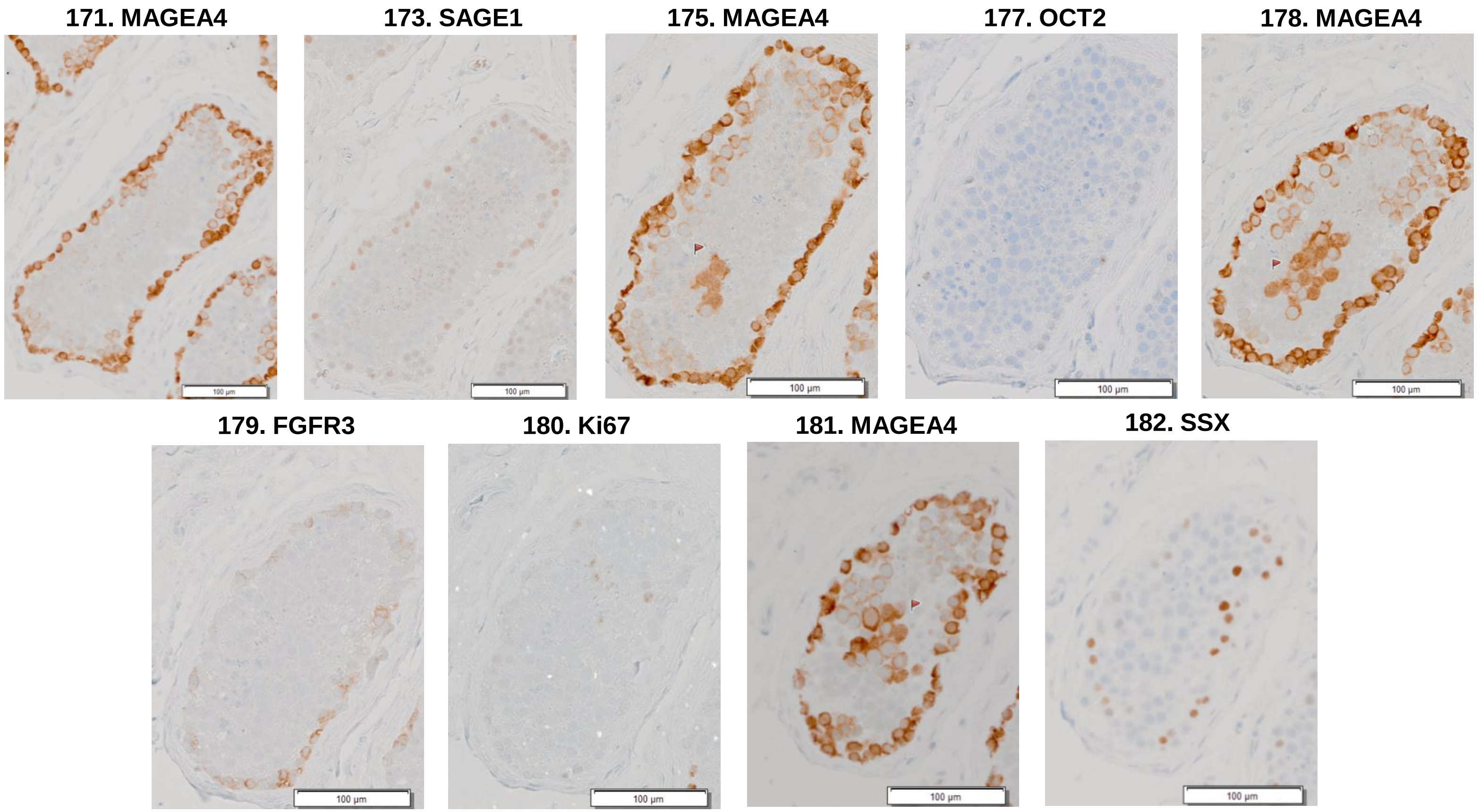

Clone no. 1-2_C23

A

Clone no.	Location	Slide 171	Slide 172	Slide 173	Slide 174	Slide 175	Slide 176	Slide 177	Slide 178	Slide 179	Slide 180	Slide 181	Minimum number of cells
		MAGEA4	SSX	SAGE1	OCT2	MAGEA4	n.s	OCT2	MAGEA4	FGFR3	Ki67	MAGEA4	
1-2_C23	Centre	NA	NA	NA	0	1<br>5 cells		0	1<br>7 cells	0	0	NA	24
						20 µm							

B

173. SAGE1

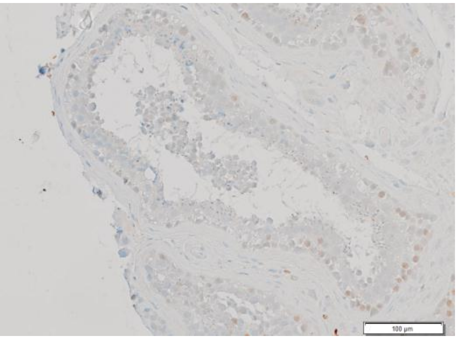

175. MAGEA4

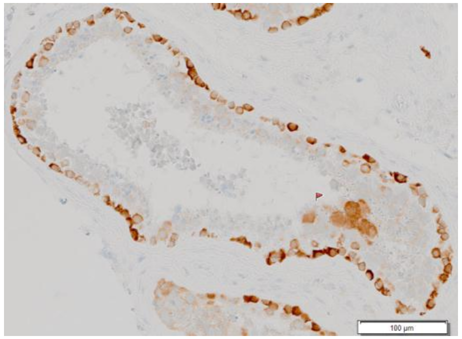

177. OCT2

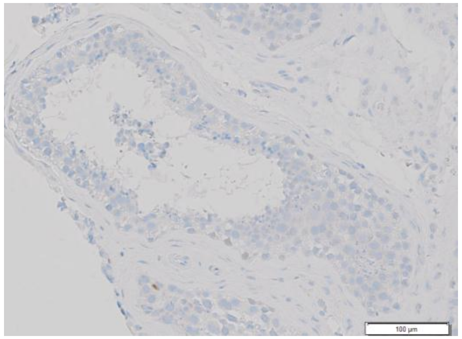

178. MAGEA4

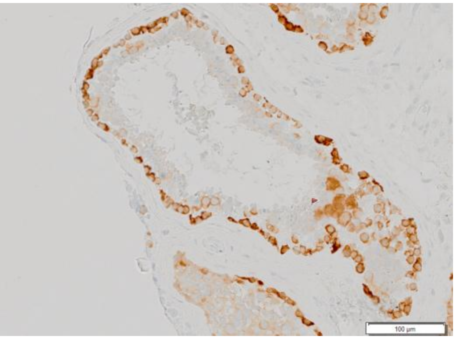

179. FGFR3

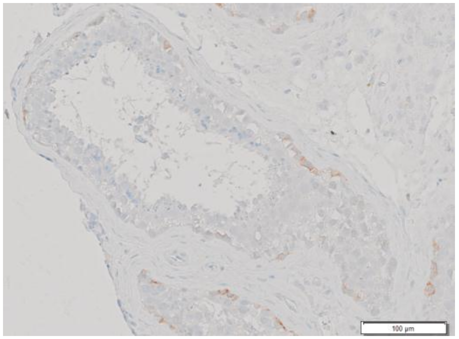

180. Ki67

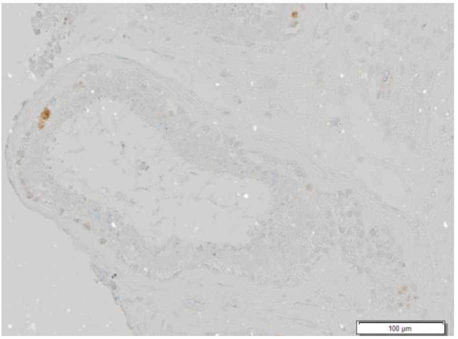

Clone no. 1-2_C24

A

Clone no.	Location	Slide 171	Slide 172	Slide 173	Slide 174	Slide 175	Slide 176	Slide 177	Slide 178	Slide 179	Slide 180	Slide 181	Slide 182	Slide 183	Slide 184	Slide 185	Minimum number of cells
		MAGEA4	SSX	SAGE1	OCT2	MAGEA4	n.s	OCT2	MAGEA4	FGFR3	Ki67	MAGEA4	SSX	FGFR3	n.s	MAGEA4	
1-2_C24	Periphery	0	0	0	0	1<br>8 cells		0	1<br>13 cells	0	0	1<br>14 cells	0			0	83
						35 µm											

B

171. MAGEA4

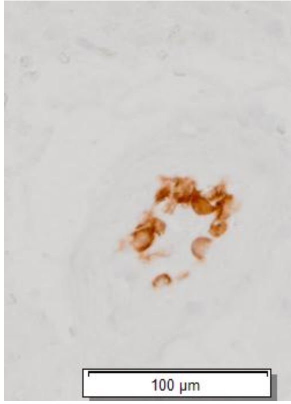

173. SAGE1

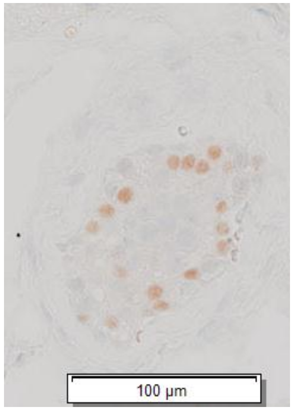

175. MAGEA4

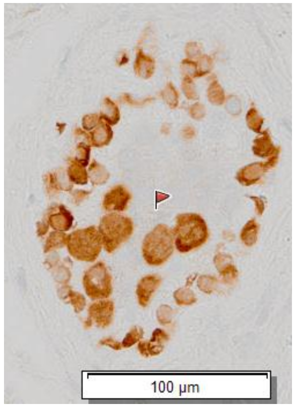

177. OCT2

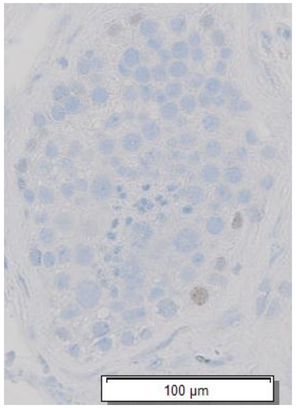

178. MAGEA4

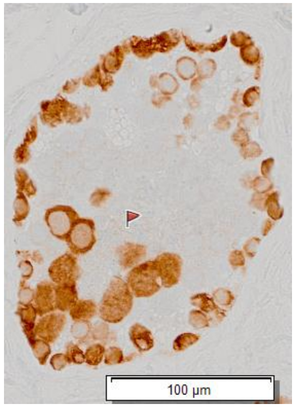

179. FGFR3

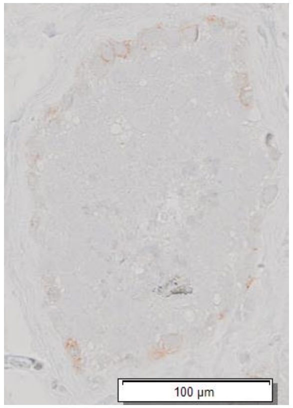

180. Ki67

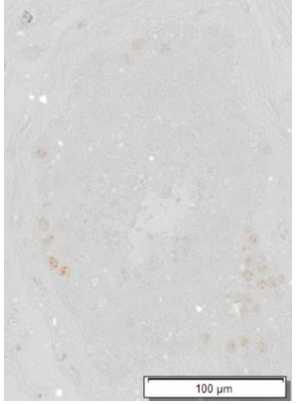

181. MAGEA4

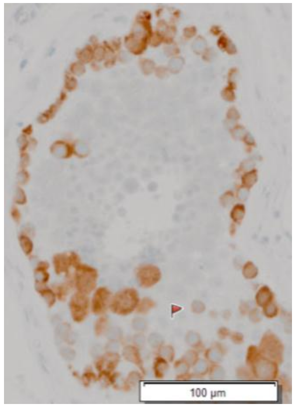

182. SSX

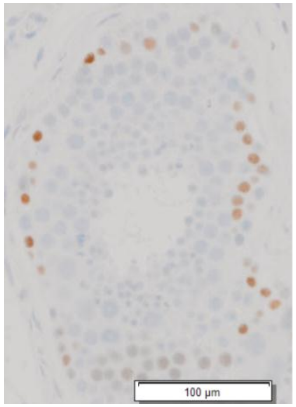

Clone no. 1-2_C25

A

Clone no.	Location	Slide 170	Slide 171	Slide 172	Slide 173	Slide 174	Slide 175	Slide 176	Slide 177	Slide 178	Slide 179	Slide 180	Slide 181	Minimum number of cells
		Ki67	MAGEA4	SSX	SAGE1	OCT2	MAGEA4	n.s	OCT2	MAGEA4	FGFR3	Ki67	MAGEA4	
1-2_C25	Centre	0	0*<br>2 cells	0	0	0	1<br>4 cells		0	1<br>5 cells	0		0	30
			40 µm											

B

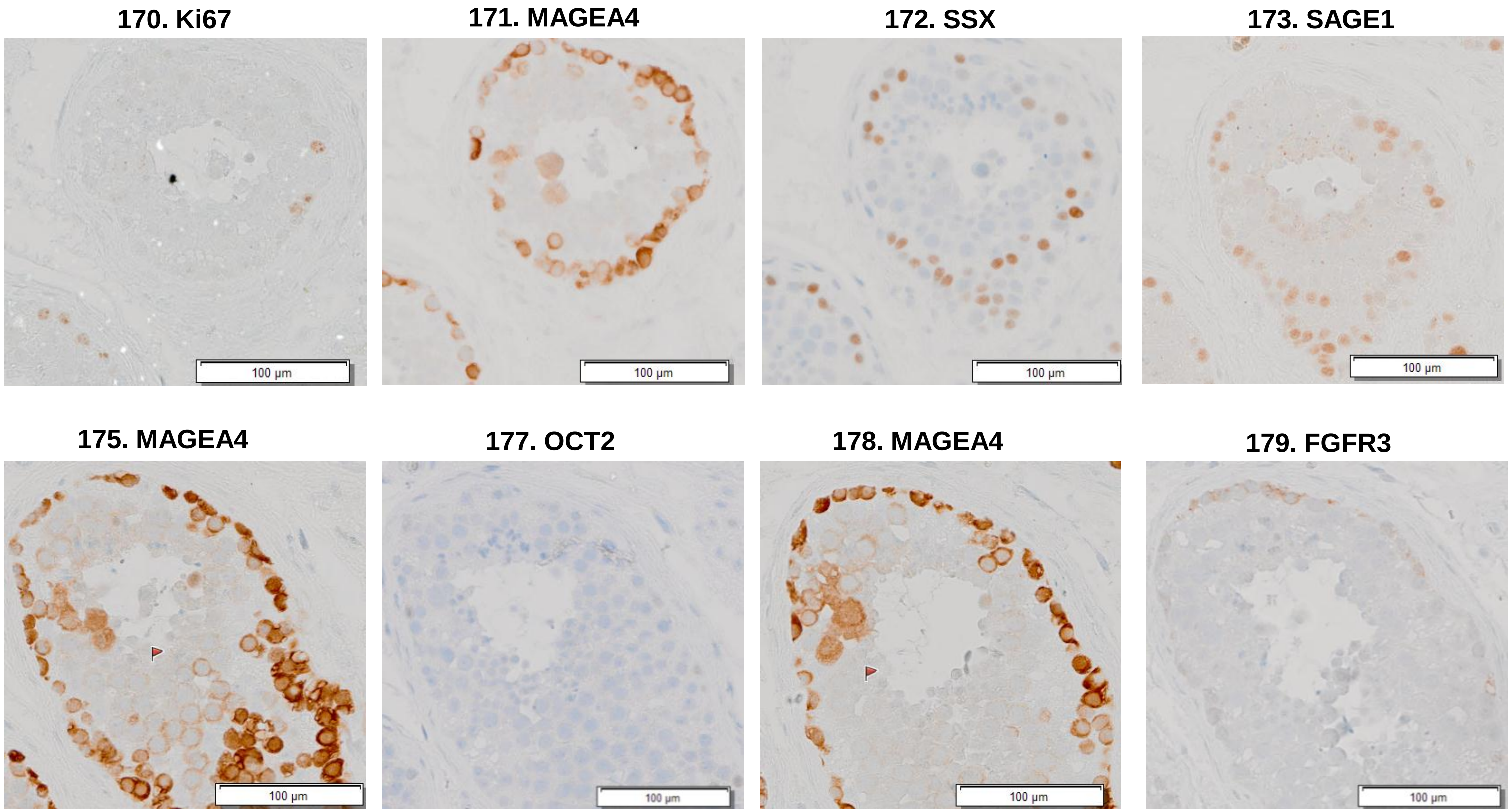

Clone no. 1-2_C26

A

Clone no.	Location	Slide 171	Slide 172	Slide 173	Slide 174	Slide 175	Slide 176	Slide 177	Slide 178	Slide 179	Slide 180	Slide 181	Slide 182	Slide 183	Slide 184	Slide 185	Minimum number of cells
		MAGEA4	SSX	SAGE1	OCT2	MAGEA4	n.s	OCT2	MAGEA4	FGFR3	Ki67	MAGEA4	SSX	FGFR3	n.s	MAGEA4	
1-2_C26	Periphery	0	0	0	0	1<br>5 cells		0	1<br>7 cells	0	0	1<br>15 cells	0			0*<br>2 cells	89
						55 µm											

B

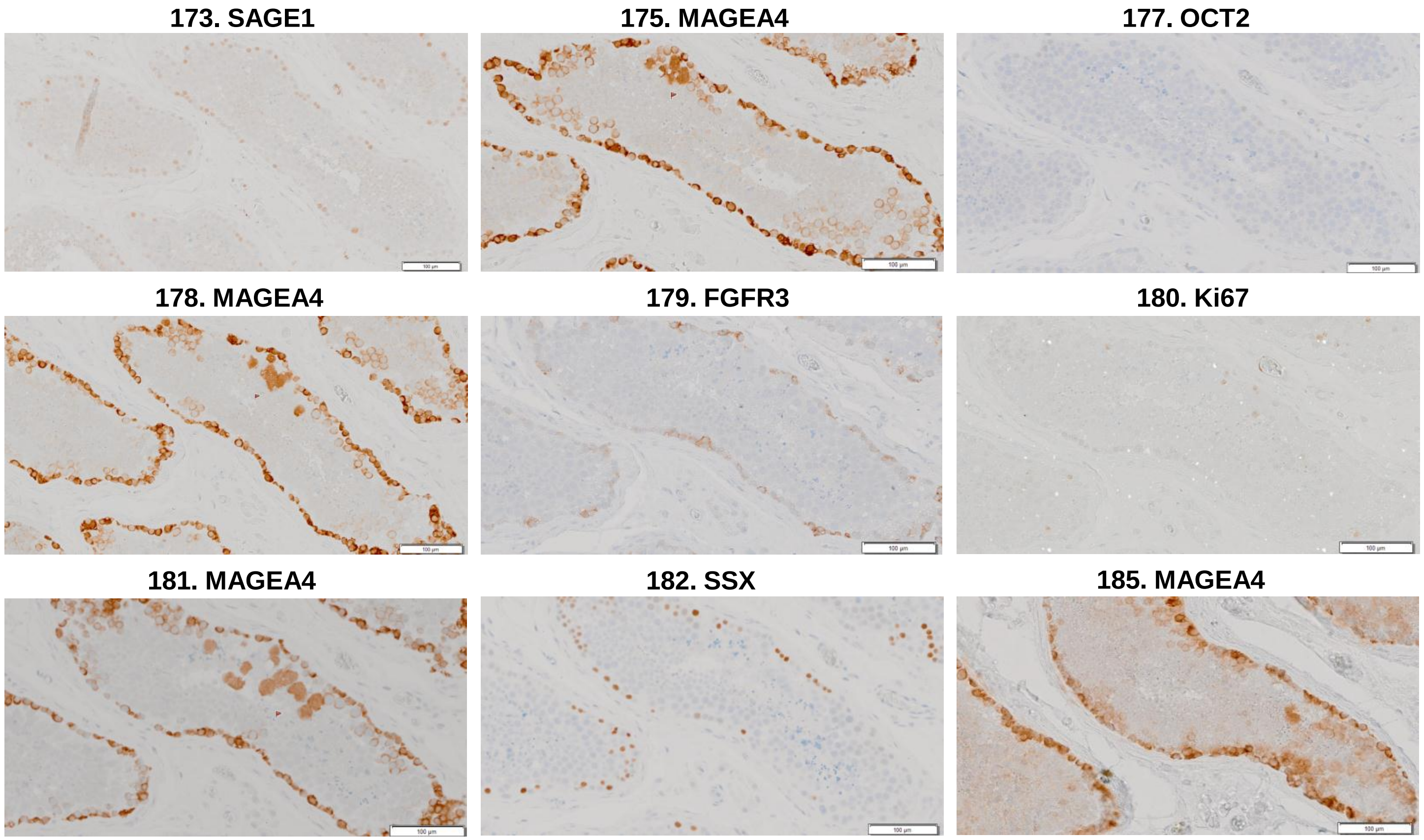

Clone no. 1-2_C27

A

Clone no.	Location	Slide 168	Slide 169	Slide 170	Slide 171	Slide 172	Slide 173	Slide 174	Slide 175	Slide 176	Slide 177	Slide 178	Slide 179	Slide 180	Slide 181	Minimum number of cells
		MAGEA4	FGFR3	Ki67	MAGEA4	SSX	SAGE1	OCT2	MAGEA4	n.s	OCT2	MAGEA4	FGFR3	Ki67	MAGEA4	
1-2_C27	Near Periphery	1	0	0	1	0	0	0	1		0	1	0		0	47
		6 cells			4 cells				3 cells			5 cells				
		55 µm														

B

168. MAGEA4

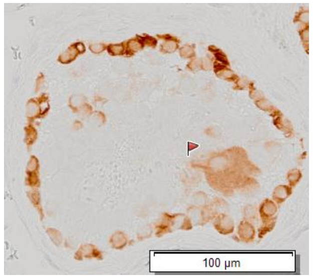

169. FGFR3

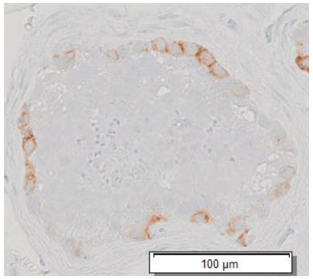

170. Ki67

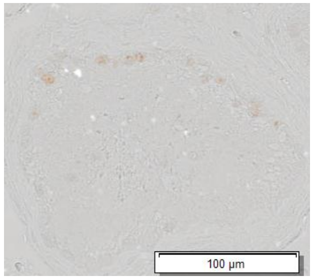

171. MAGEA4

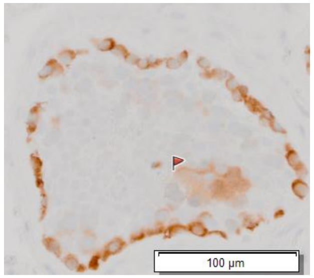

172. SSX

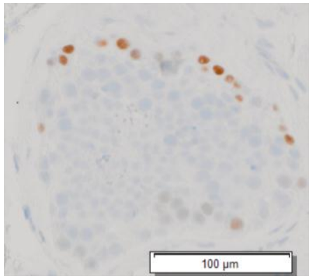

173. SAGE1

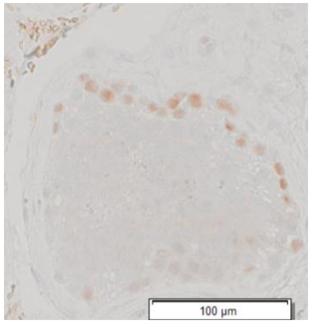

174. OCT2

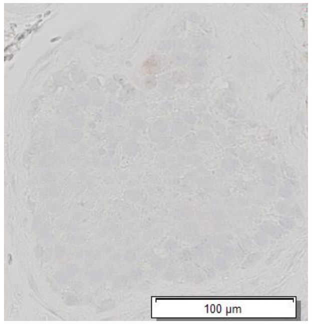

175. MAGEA4

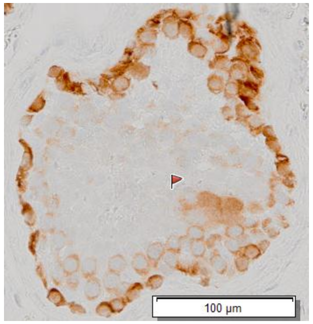

178. MAGEA4

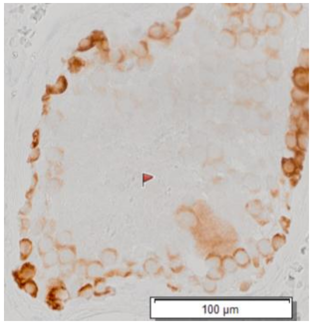

181. MAGEA4

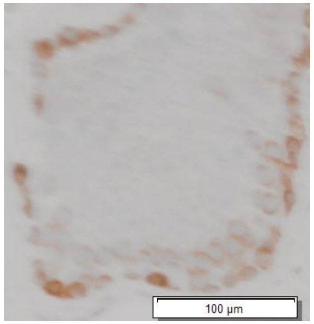

Clone no. 1-2_C28

A

Clone no.	Location	Slide 168	Slide 169	Slide 170	Slide 171	Slide 172	Slide 173	Slide 174	Slide 175	Slide 176	Slide 177	Slide 178	Slide 179	Slide 180	Slide 181	Minimum number of cells
		MAGEA4	FGFR3	Ki67	MAGEA4	SSX	SAGE1	OCT2	MAGEA4	n.s	OCT2	MAGEA4	FGFR3	Ki67	MAGEA4	
1-2_C28	Near Periphery	0		0	1<br>3 cells	0	0	0	1<br>4 cells		0	1<br>3 cells	0		0	28
					40 µm											

B

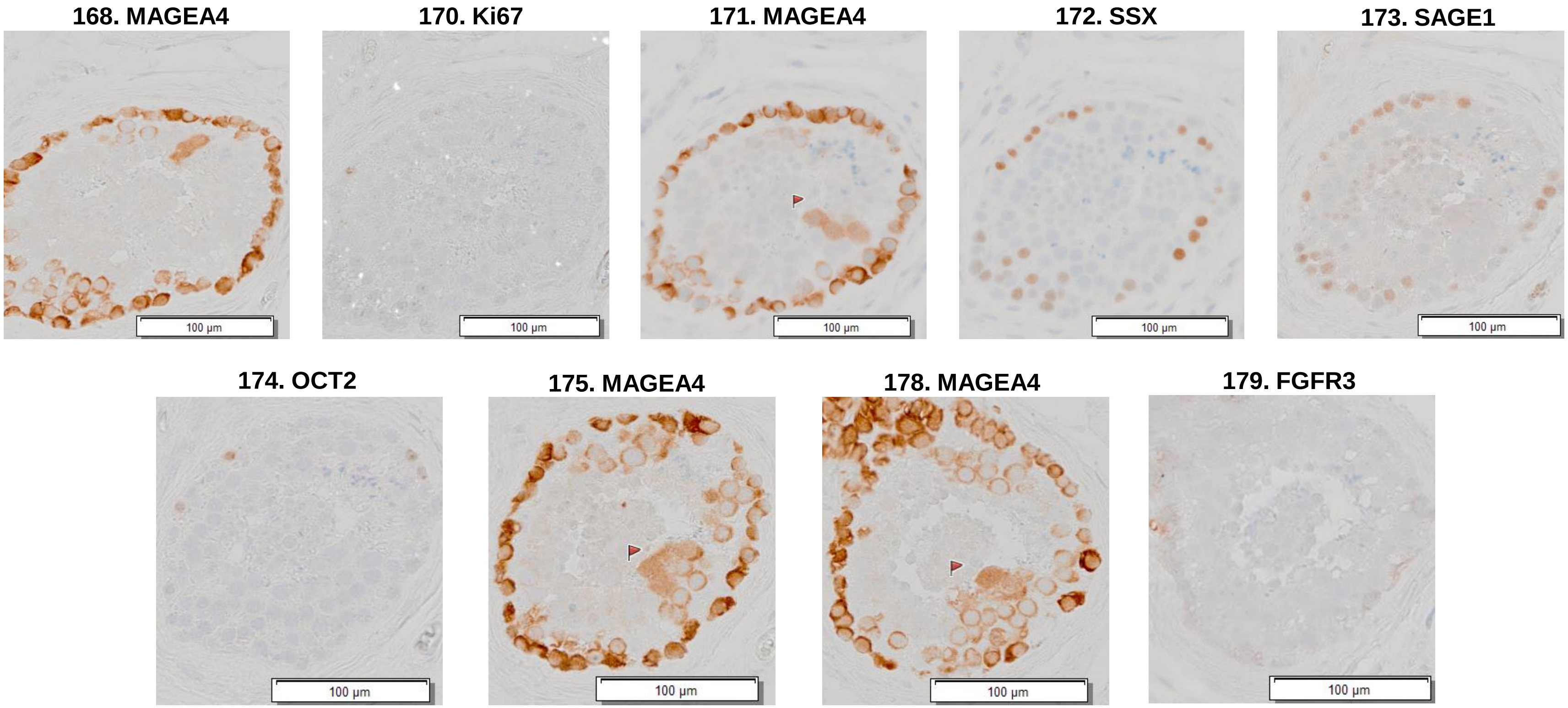

Clone no. 1-2_C29

A

Clone no.	Location	Slide 171	Slide 172	Slide 173	Slide 174	Slide 175	Slide 176	Slide 177	Slide 178	Slide 179	Slide 180	Slide 181	Minimum number of cells
		MAGEA4	SSX	SAGE1	OCT2	MAGEA4	n.s	OCT2	MAGEA4	FGFR3	Ki67	MAGEA4	
1-2_C29	Near Periphery	0	0	0	0	1<br>6 cells		0	1<br>3 cells	0		0	18
						20 µm							

B

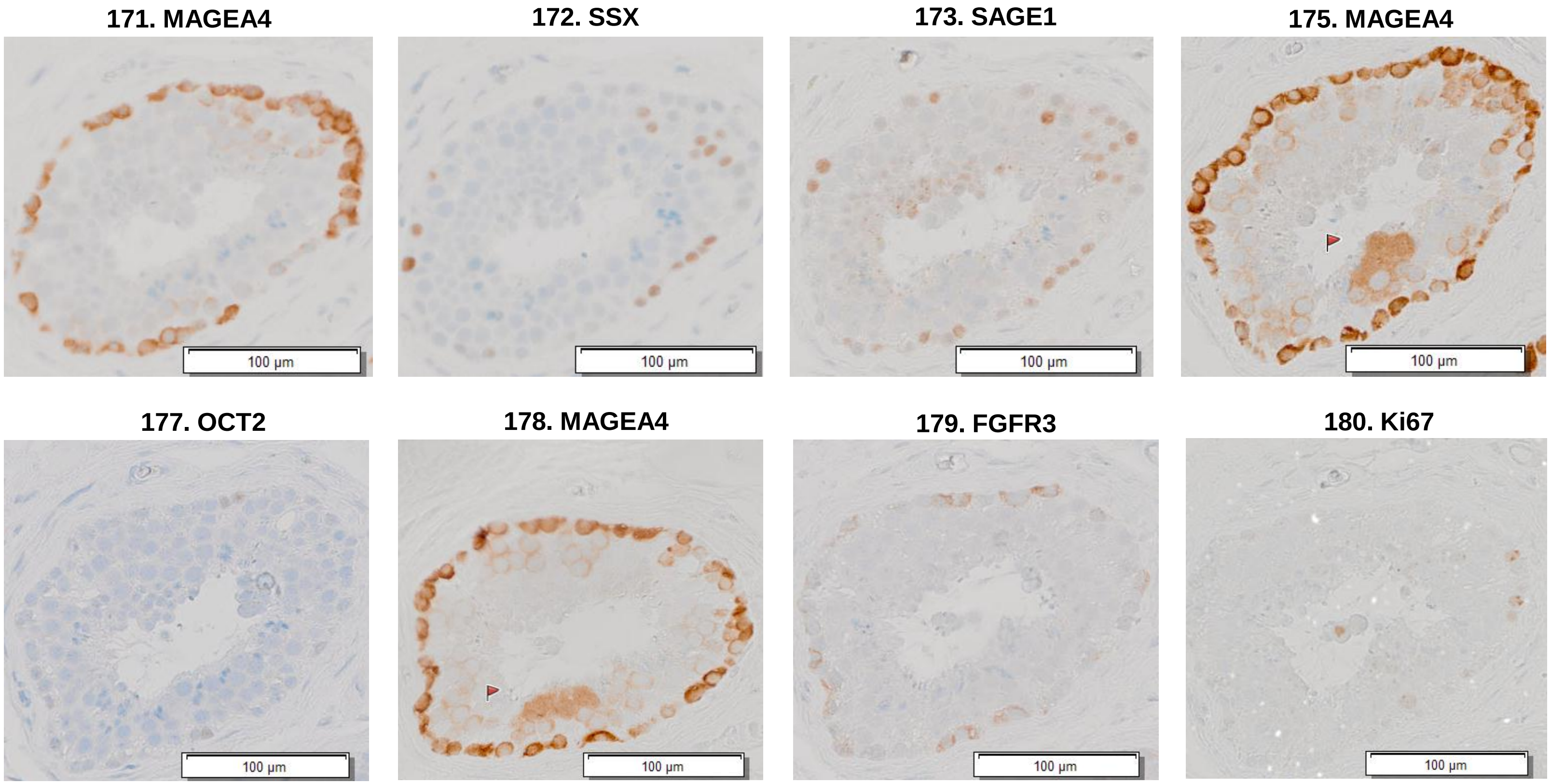

Clone no. 1-2_C30

A

Clone no.	Location	Slide 175	Slide 176	Slide 177	Slide 178	Slide 179	Slide 180	Slide 181	Slide 182	Slide 183	Slide 184	Slide 185	Minimum number of cells
		MAGEA4	n.s	OCT2	MAGEA4	FGFR3	Ki67	MAGEA4	SSX	FGFR3	n.s	MAGEA4	
1-2_C30	Centre	NA		0	1<br>6 cells	0	0	1<br>5 cells	0			NA	22
					20 µm								

B

175. MAGEA4

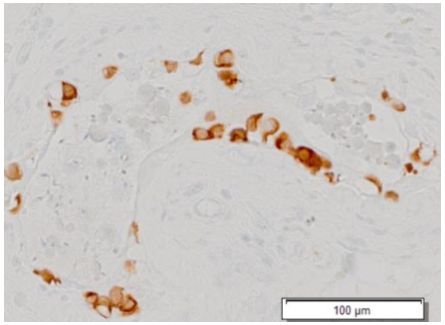

177. OCT2

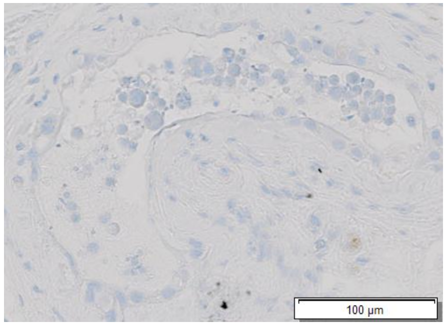

178. MAGEA4

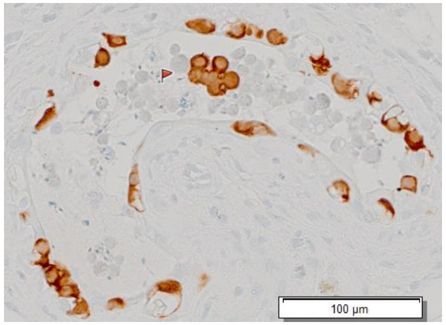

179. FGFR3

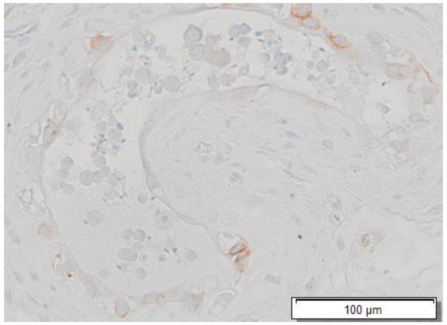

180. Ki67

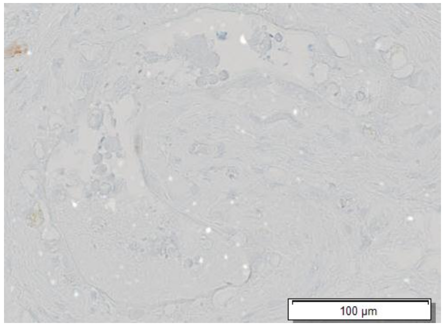

181. MAGEA4

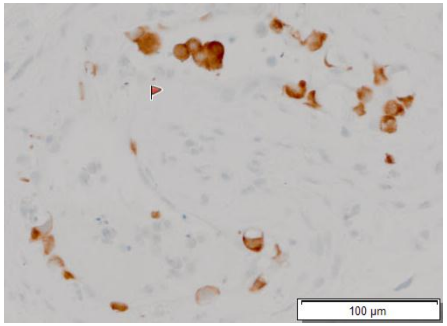

182. SSX

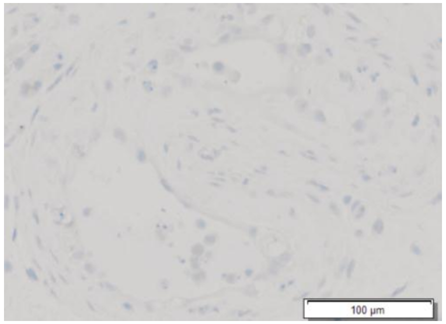

Clone no. 1-2_C31

A

<table>
<tr><th rowspan="2">Clone no.</th><th rowspan="2">Location</th><th>Slide 171</th><th>Slide 172</th><th>Slide 173</th><th>Slide 174</th><th>Slide 175</th><th>Slide 176</th><th>Slide 177</th><th>Slide 178</th><th>Slide 179</th><th>Slide 180</th><th>Slide 181</th><th rowspan="2">Minimum number of cells</th></tr>
<tr><th>MAGEA4</th><th>SSX</th><th>SAGE1</th><th>OCT2</th><th>MAGEA4</th><th>n.s</th><th>OCT2</th><th>MAGEA4</th><th>FGFR3</th><th>Ki67</th><th>MAGEA4</th></tr>
<tr><td rowspan="2">1-2_C31</td><td rowspan="2">Centre</td><td rowspan="2">0</td><td rowspan="2">0</td><td rowspan="2">0</td><td rowspan="2">0</td><td>1
6 cells</td><td></td><td>0</td><td>1
7 cells</td><td rowspan="2">0</td><td rowspan="2">0</td><td rowspan="2">0</td><td rowspan="2">26</td></tr>
<tr><td colspan="4">20 µm</td></tr>
</table>

B

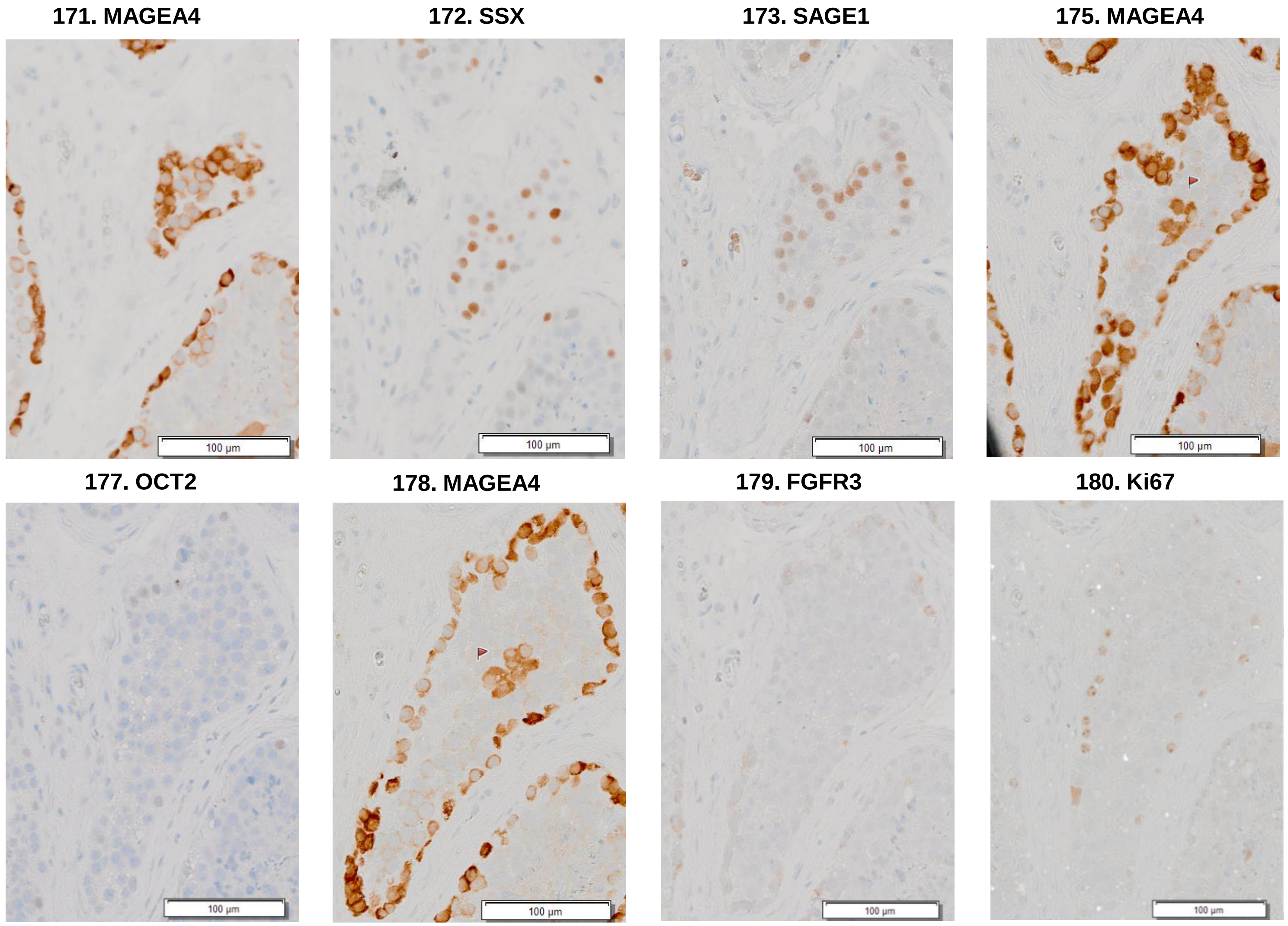

Clone no. 1-2_C32

A

Clone no.	Location	Slide 175	Slide 176	Slide 177	Slide 178	Slide 179	Slide 180	Slide 181	Slide 182	Slide 183	Slide 184	Slide 185	Minimum number of cells
		MAGEA4	n.s	OCT2	MAGEA4	FGFR3	Ki67	MAGEA4	SSX	FGFR3	n.s	MAGEA4	
1-2_C32	Near Periphery	NA		0	1<br>6 cells	0	0	1<br>3 cells	0			NA	18
					20 µm								

B

175. MAGEA4

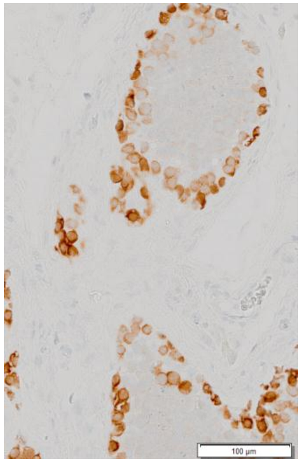

177. OCT2

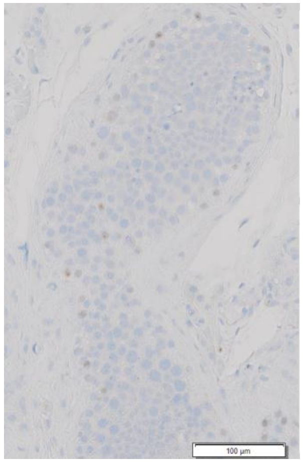

178. MAGEA4

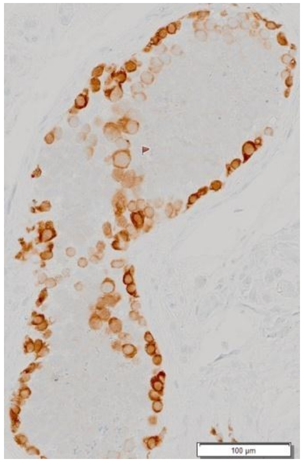

179. FGFR3

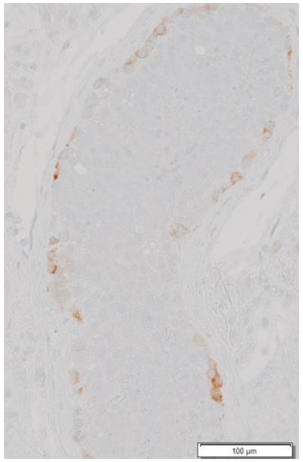

180. Ki67

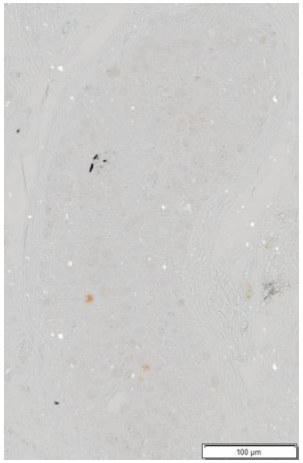

181. MAGEA4

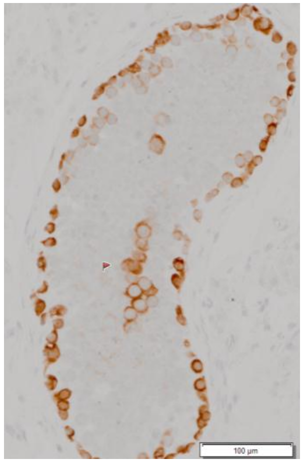

182. SSX

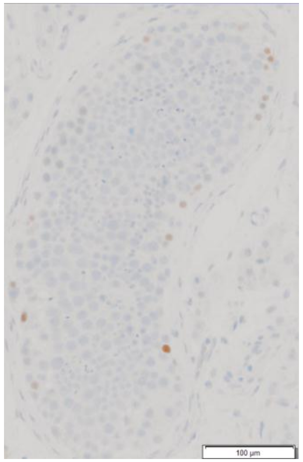

Clone no. 1-2_C33

A

Clone no.	Location	Slide 175	Slide 176	Slide 177	Slide 178	Slide 179	Slide 180	Slide 181	Slide 182	Slide 183	Slide 184	Slide 185	Minimum number of cells
		MAGEA4	n.s	OCT2	MAGEA4	FGFR3	Ki67	MAGEA4	SSX	FGFR3	n.s	MAGEA4	
1-2_C33	Centre	0		0	1<br>5 cells	0	0	0*<br>2 cells	1<br>3 cells			0	17
					25 µm								

B

175. MAGEA4

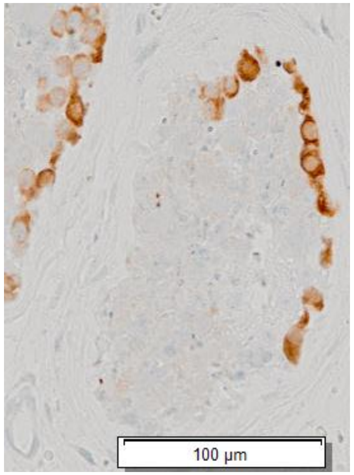

177. OCT2

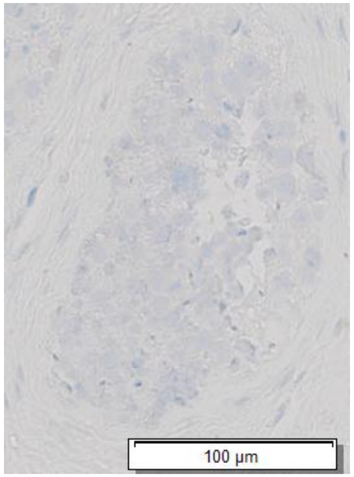

178. MAGEA4

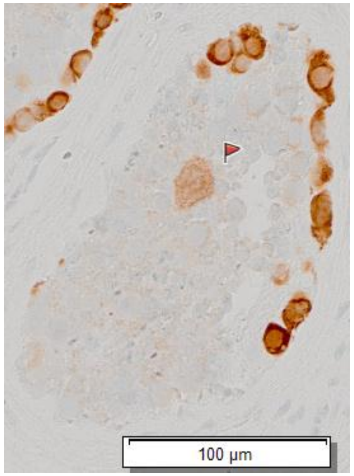

179. FGFR3

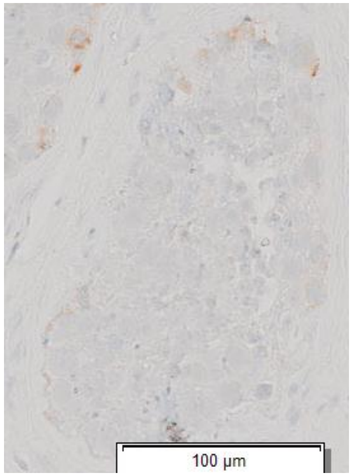

180. Ki67

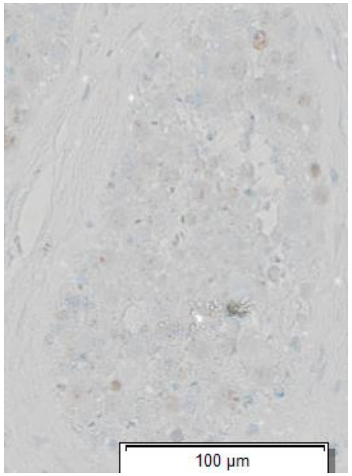

181. MAGEA4

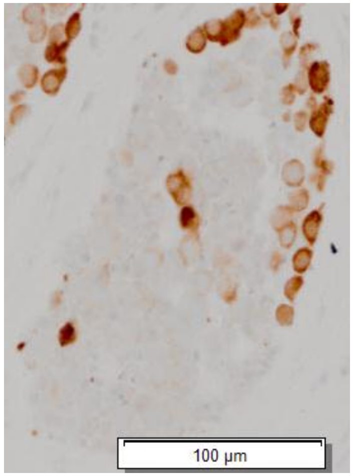

182. SSX

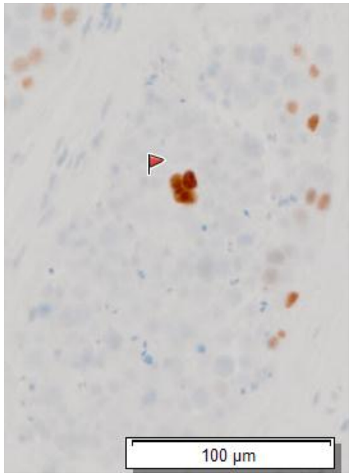

Clone no. 1-2_C34

A

Clone no.	Location	Slide 175	Slide 176	Slide 177	Slide 178	Slide 179	Slide 180	Slide 181	Slide 182	Slide 183	Slide 184	Slide 185	Minimum number of cells
		MAGEA4	n.s	OCT2	MAGEA4	FGFR3	Ki67	MAGEA4	SSX	FGFR3	n.s	MAGEA4	
1-2_C34	Centre	0		0	1<br>13 cells	0	0	1<br>6 cells	0			0	38
					20 µm								

B

175. MAGEA4

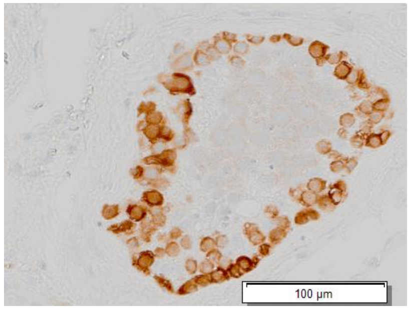

177. OCT2

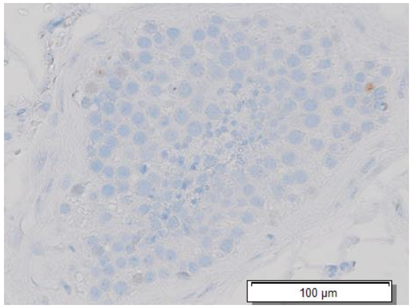

178. MAGEA4

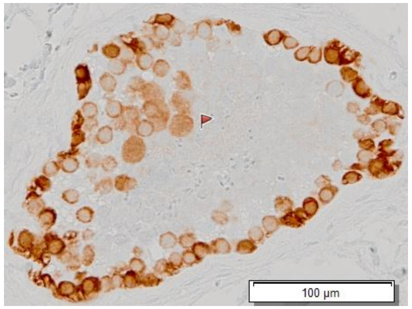

179. FGFR3

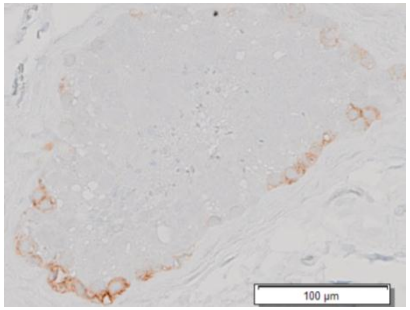

180. Ki67

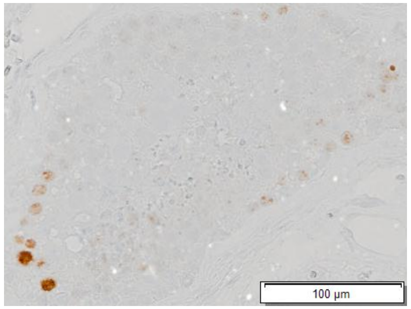

181. MAGEA4

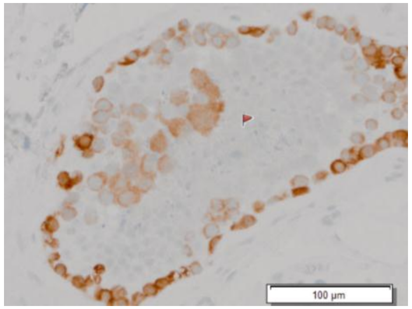

182. SSX

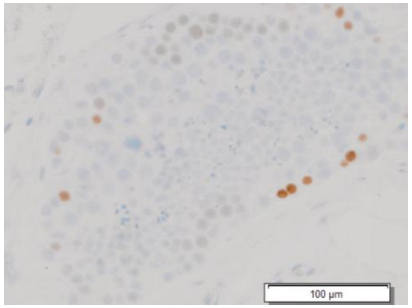

Clone no. 1-2_ C35

A

<table>
<tr><td rowspan="2">Clone no.</td><td rowspan="2">Location</td><td>Slide 178</td><td>Slide 179</td><td>Slide 180</td><td>Slide 181</td><td>Slide 182</td><td>Slide 183</td><td>Slide 184</td><td>Slide 185</td><td rowspan="2">Minimum number of cells</td></tr>
<tr><td>MAGEA4</td><td>FGFR3</td><td>Ki67</td><td>MAGEA4</td><td>SSX</td><td>FGFR3</td><td>n.s</td><td>MAGEA4</td></tr>
<tr><td rowspan="3">1-2_C35</td><td rowspan="3">Centre</td><td rowspan="3">NA</td><td rowspan="3">0</td><td rowspan="3">0</td><td>1</td><td>1</td><td rowspan="3">NA</td><td rowspan="3"></td><td rowspan="3">NA</td><td rowspan="3">20</td></tr>
<tr><td>13 cells</td><td>7 cells</td></tr>
<tr><td colspan="2">10 µm</td></tr>
</table>

B

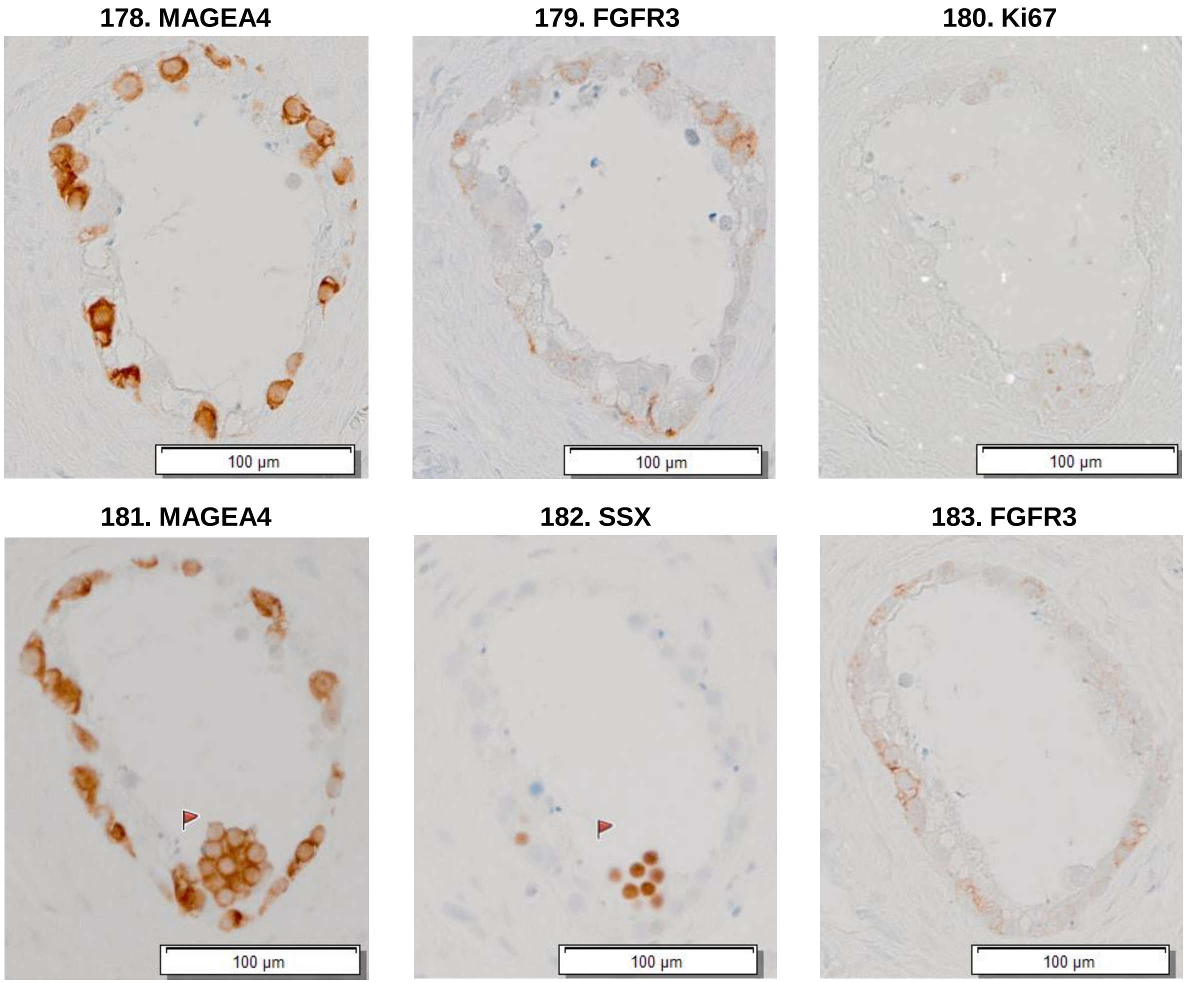

Clone no. 1-2_C36

A

Clone no.	Location	Slide 175	Slide 176	Slide 177	Slide 178	Slide 179	Slide 180	Slide 181	Slide 182	Slide 183	Slide 184	Slide 185	Minimum number of cells
		MAGEA4	n.s	OCT2	MAGEA4	FGFR3	Ki67	MAGEA4	SSX	FGFR3	n.s	MAGEA4	
1-2_C36	Periphery	0*		0	1	0	0	1	0*	0		0	73
		3 cells			14 cells			10 cells	5 cells				
		40 µm											

B

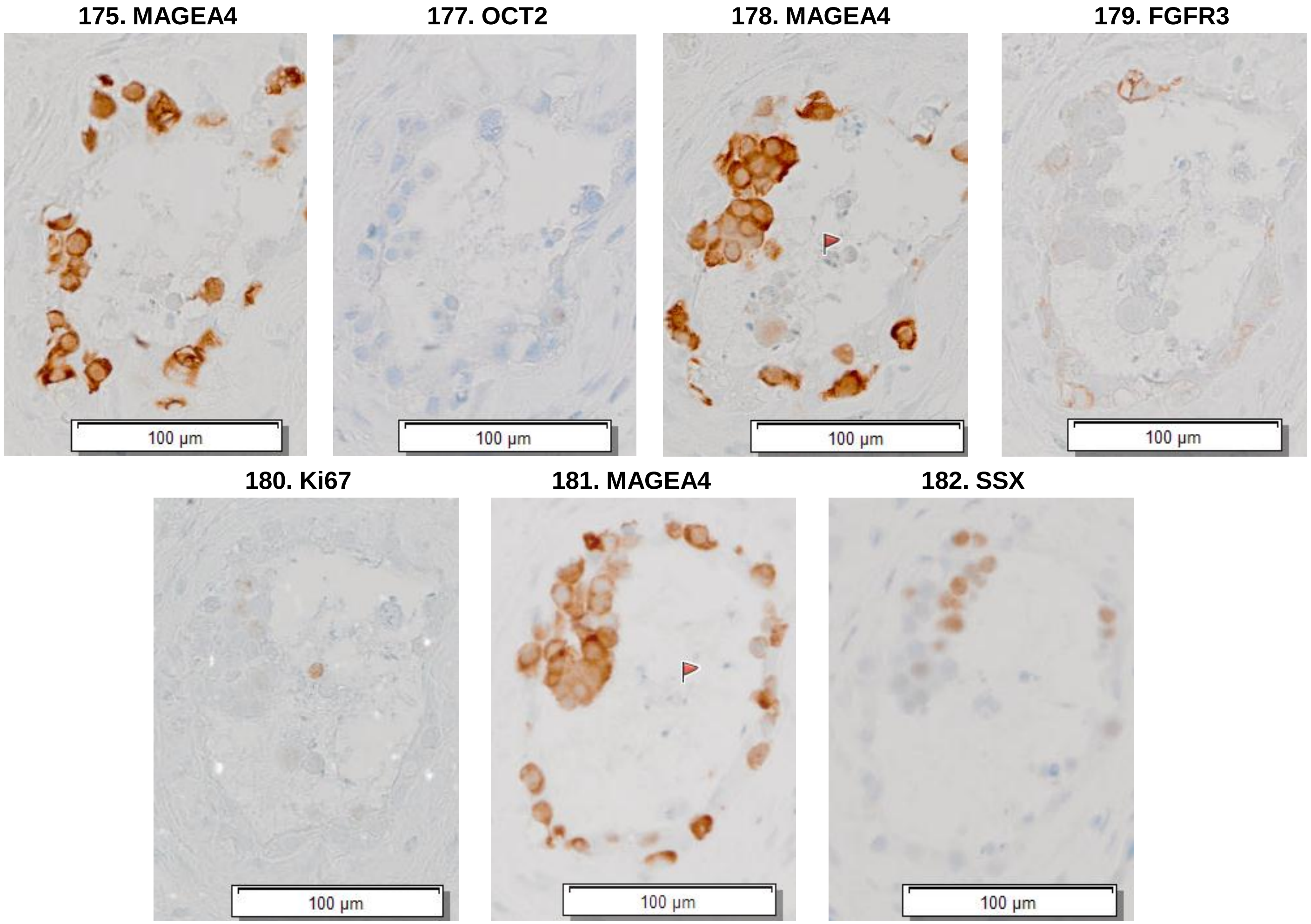

Clone no. 1-2_C37

A

Clone no.	Location	Slide 175	Slide 176	Slide 177	Slide 178	Slide 179	Slide 180	Slide 181	Slide 182	Slide 183	Slide 184	Slide 185	Minimum number of cells
		MAGEA4	n.s	OCT2	MAGEA4	FGFR3	Ki67	MAGEA4	SSX	FGFR3	n.s	MAGEA4	
1-2_C37	Centre	0		0	1<br>7 cells	0	0	1<br>6 cells	0	0		0	26
					20 µm								

B

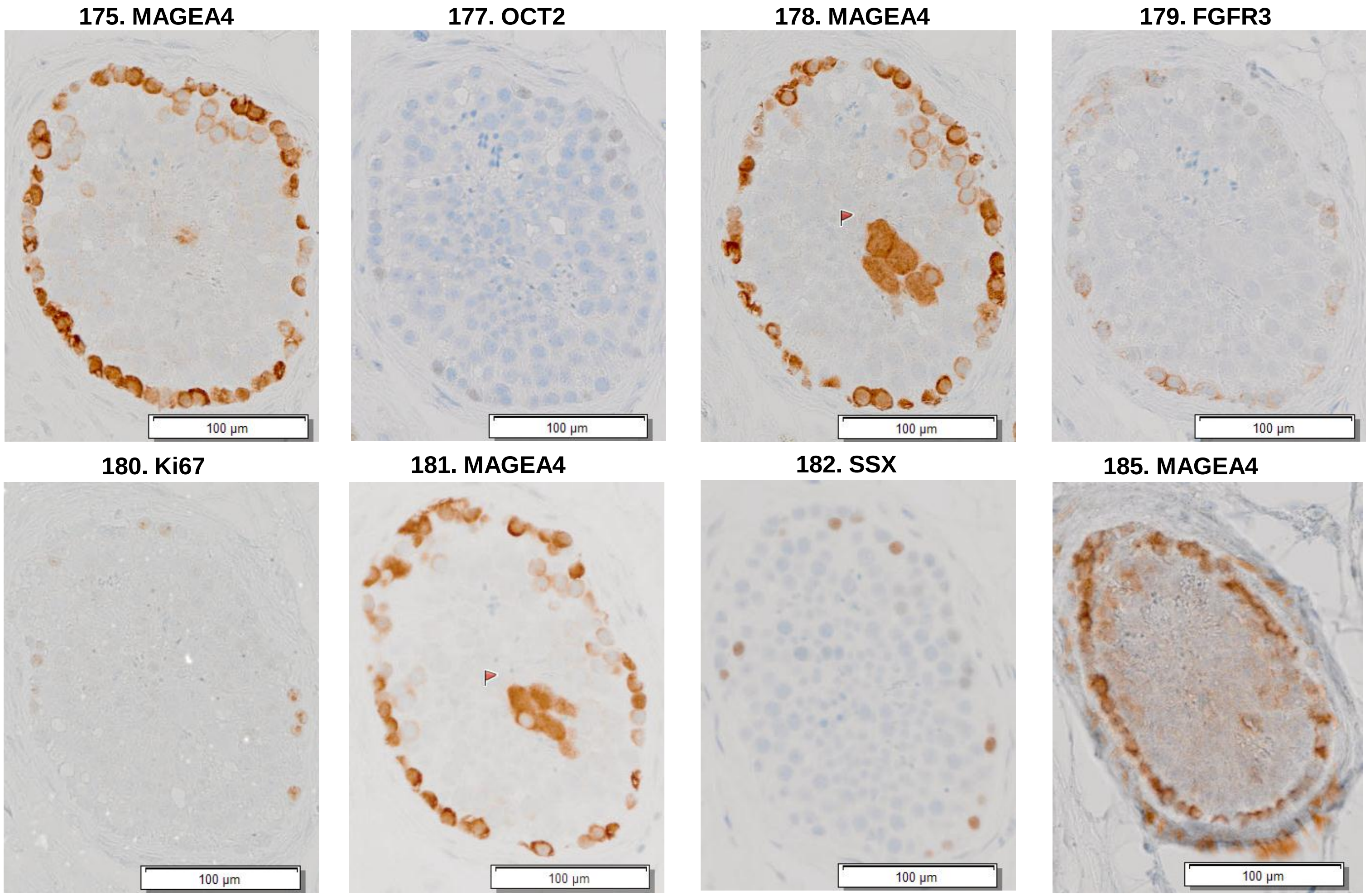

Clone no. 1-2_C38

A

Clone no.	Location	Slide 178	Slide 179	Slide 180	Slide 181	Slide 182	Slide 183	Slide 184	Slide 185	Slide 186	Slide 187	Slide 188	Slide 189	Slide 190	Slide 191	Minimum number of cells
		MAGEA4	FGFR3	Ki67	MAGEA4	SSX	FGFR3	n.s	MAGEA4	n.s	n.s	n.s	n.s	n.s	MAGEA4	
1-2_C38	Near Periphery	0		0	1<br>4 cells	0	0		1<br>9 cells						0	33
					25 µm											

B

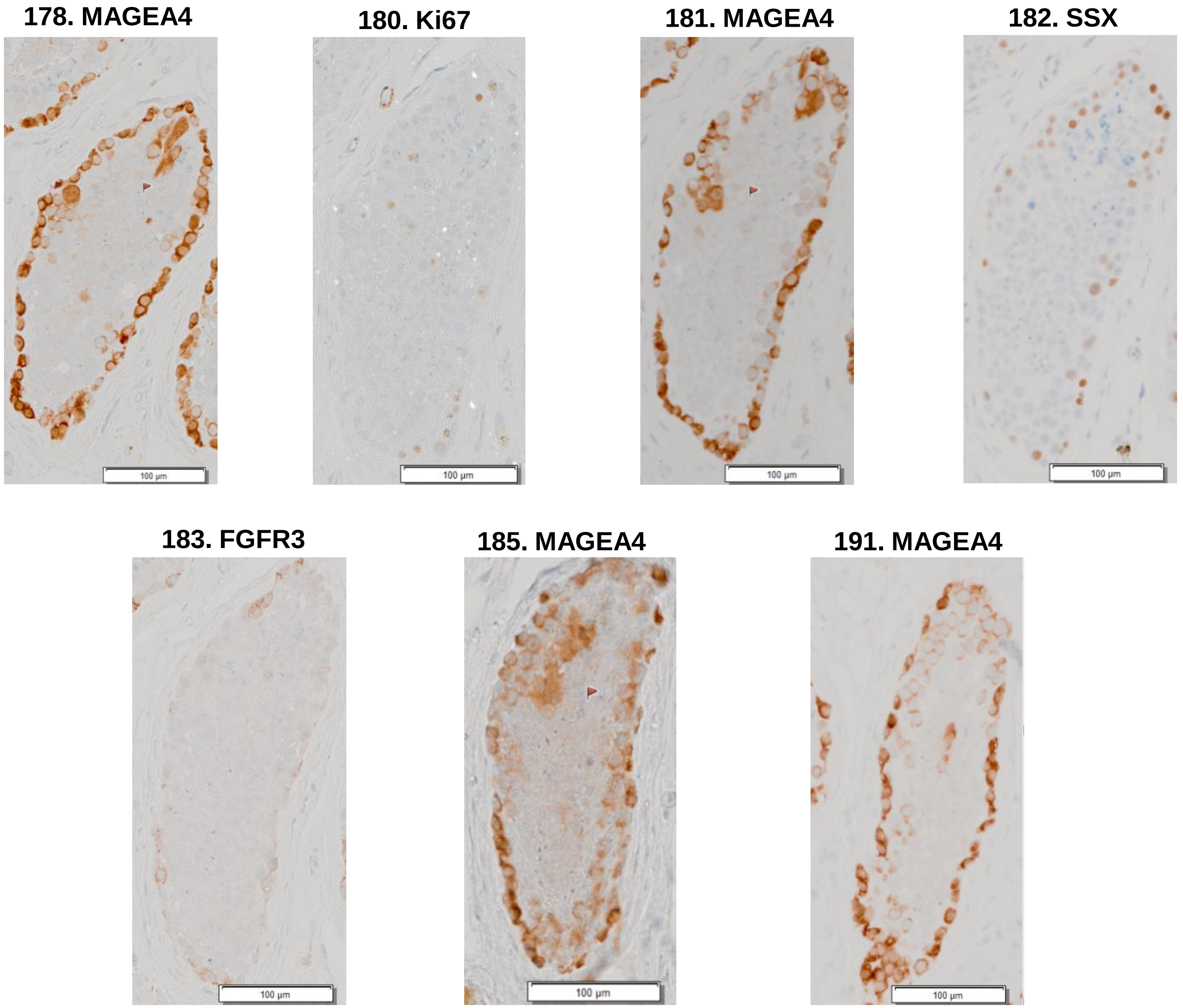

Clone no. 1-2_C39

A

Clone no.	Location	Slide 178	Slide 179	Slide 180	Slide 181	Slide 182	Slide 183	Slide 184	Slide 185	Slide 186	Slide 187	Slide 188	Slide 189	Slide 190	Slide 191	Minimum number of cells
		MAGEA4	FGFR3	Ki67	MAGEA4	SSX	FGFR3	n.s	MAGEA4	n.s	n.s	n.s	n.s	n.s	MAGEA4	
1-2_C39	Near Periphery	0		0	1<br>3 cells	0	0		1<br>4 cells						0*<br>2 cells	35
					55 µm											

B

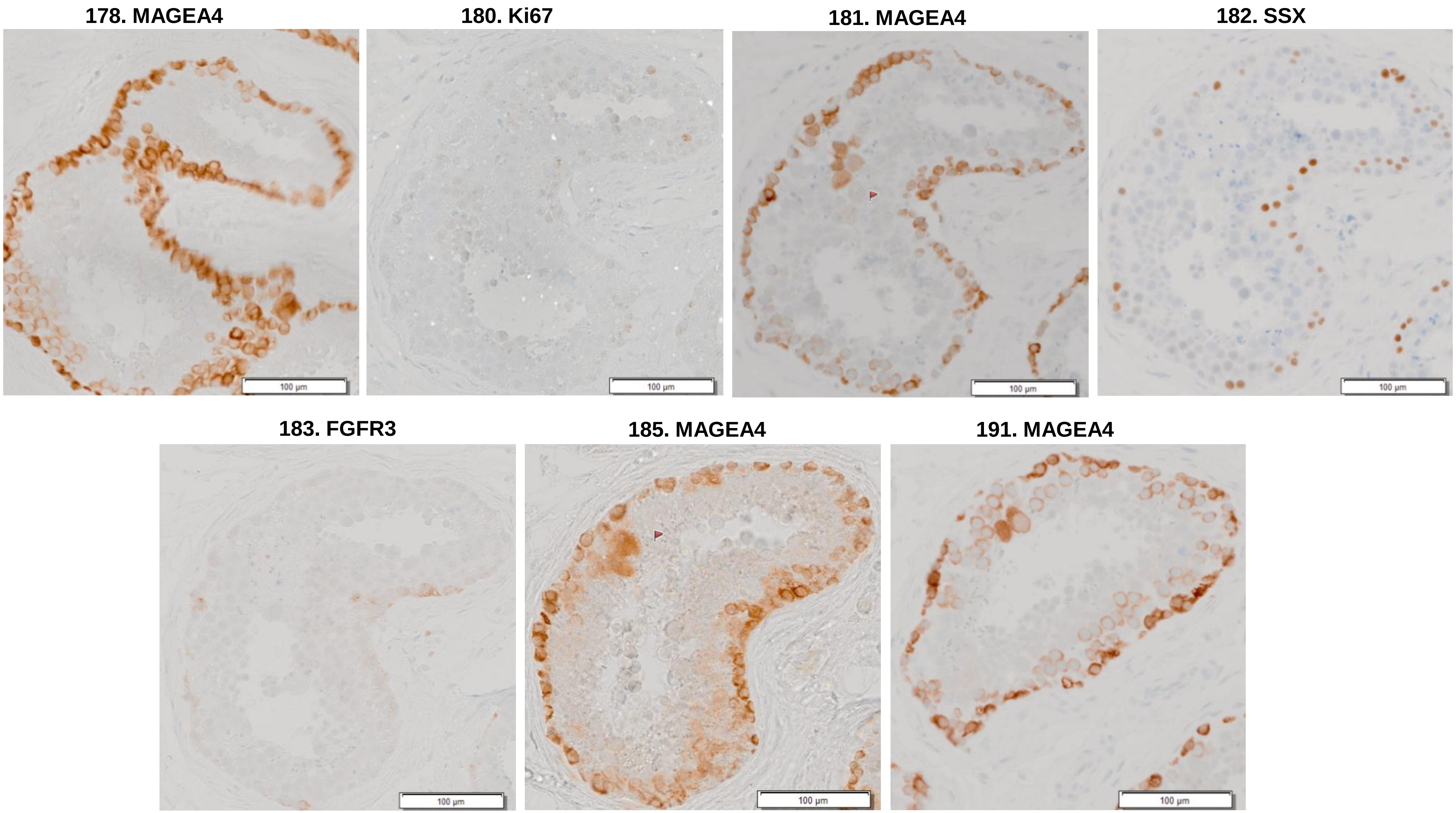

Supplement: Dataset S2 — 84 microclones identified in samples 1–1 and 1–2. (PDF) [file pone.0042382.s006.pdf]
