# Supplementary material for: Selfish Spermatogonial Selection: Evidence from an Immunohistochemical Screen in Testes of Elderly Men
Source: PLoS One. 2012 Aug 6;7(8):e42382. doi: 10.1371/journal.pone.0042382 (PMC3412839; doi:10.1371/journal.pone.0042382)
Supplement: Text S1 — Legend for Video S1. (DOC) [file pone.0042382.s007.doc]

**Text S1. Legend for Video S1.** The movie demonstrates how sections containing immunopositive tubules were aligned in 3D space and how the constructed model fits in the aligned sections. Each of the five immunopositive tubular cross-sections in section 51 (the uppermost section) were colour coded (purple, blue, yellow, green and pink). The reconstruction reveals that the blue, purple, yellow and green immunopositive tubular-cross sections belong to the same tubule. Rotation around the *x*-axis reveals how the structure is positioned between the outer sections and rotation around the *z*-axis reveals how four of the five immunopositive tubular cross-sections are connected. Although they are in close proximity, there is no apparent connection between the pink and green tubules. The model is displayed to scale.
